# Structural and Regulatory Characterization of the Placental Epigenome at Its Maternal Interface

**DOI:** 10.1371/journal.pone.0014723

**Published:** 2011-02-23

**Authors:** Tianjiao Chu, Daniel Handley, Kimberly Bunce, Urvashi Surti, W. Allen Hogge, David G. Peters

**Affiliations:** 1 Department of Obstetrics, Gynecology and Reproductive Sciences, University of Pittsburgh, Pennsylvania, United States of America; 2 Center for Fetal Medicine, Magee-Womens Research Institute, Pittsburgh, Pennsylvania, United States of America; Brunel University, United Kingdom

## Abstract

Epigenetics can be loosely defined as the study of cellular “traits” that influence biological phenotype in a fashion that is not dependent on the underlying primary DNA sequence. One setting in which epigenetics is likely to have a profound influence on biological phenotype is during intrauterine development. In this context there is a defined and critical window during which balanced homeostasis is essential for normal fetal growth and development. We have carried out a detailed structural and functional analysis of the placental epigenome at its maternal interface. Specifically, we performed genome wide analysis of DNA methylation in samples of chorionic villus (CVS) and maternal blood cells (MBC) using both commercially available and custom designed microarrays. We then compared these data with genome wide transcription data for the same tissues. In addition to the discovery that CVS genomes are significantly more hypomethylated than their MBC counterparts, we identified numerous tissue-specific differentially methylated regions (T-DMRs). We further discovered that these T-DMRs are clustered spatially along the genome and are enriched for genes with tissue-specific biological functions. We identified unique patterns of DNA methylation associated with distinct genomic structures such as gene bodies, promoters and CpG islands and identified both direct and inverse relationships between DNA methylation levels and gene expression levels in gene bodies and promoters respectively. Furthermore, we found that these relationships were significantly associated with CpG content. We conclude that the early gestational placental DNA methylome is highly organized and is significantly and globally associated with transcription. These data provide a unique insight into the structural and regulatory characteristics of the placental epigenome at its maternal interface and will drive future analyses of the role of placental dysfunction in gestational disease.

## Introduction

One area of genomics that is attracting intense interest is epigenetics, which can be loosely defined as the study of cellular “traits” that influence biological phenotype in a fashion that is not dependent on the underlying primary DNA sequence[1]. Of particular significance is that epigenetic changes in genome function can result in altered phenotypic states that are not only sustained in the short term but may be heritable in a mitotic and even meiotic fashion [2], [3], [4], [5], [6], [7], [8]. Gene environment interactions are centrally involved in our susceptibility to disease and these influences are likely to be mediated to a large degree via epigenetic regulatory phenomena[9].

An important aspect of epigenetics is DNA methylation. There is abundant evidence to suggest that DNA methylation is intimately involved in the regulation of gene expression[10] and that DNA methylation patterns can be altered as a component of disease pathogenesis[11], [12]. Evidence is also emerging to suggest that DNA methylation is altered during development and by environmental stress[6], [13], [14]. However, the mechanisms by which these epigenetic influences are exerted are by no means clear. There are many gaps in our knowledge regarding the function of DNA methylation in various genomic contexts such as promoters and gene bodies and the mechanisms by which DNA methylation can influence gene expression.

One setting in which epigenetics is likely to have a profound influence on biological phenotype is during intrauterine development. In this context there is a defined and critical window during which balanced homeostasis is essential for normal fetal growth and development. Because of its central role in guiding fetal development and acting as the gatekeeper of maternal environmental exposure, the placenta responds to and is potentially marked in an epigenetic context by environmental insults which suggests that the placental epigenome might serve not only as a record of *in utero* exposure but also as a mediator and/or modulator of disease pathogenesis[15], [16]. This is significant because early gestational placental dysfunction has been implicated in a number of diseases including preeclampsia [17]. Furthermore, it is known that villus-derived apoptotic bodies are a major source of the placentally-derived DNA and RNA species in maternal plasma and it has been demonstrated that the quantitative analysis of these molecules has significant utility for the diagnosis and prognosis of both genetic and complex fetal diseases [18], [19], [20], [21].

In light of the above, we have undertaken a comprehensive analysis of cytosine methylation patterns in chorionic villus samples (CVS) and gestational age-matched maternal blood cells (MBCs) using two distinct microarray based methods. We provide the first detailed analysis of the chorionic villus methylome at the maternal interface in the context of both global gene expression patterns and primary DNA sequence.

## Materials and Methods

### Tissue Handling and DNA Extraction

The collection of tissue samples was approved by the University of Pittsburgh Institutional Review Board (PRO07070298). This project includes no involvement of human subjects according to the federal regulations [§46.102(f)]. That is, no data was obtained through intervention or interaction with the individual, nor was any identifiable private information obtained. All samples used in this study were discarded de-identified tissues. CVS samples were obtained between gestational weeks 11 and 13 from the Magee Womens Hospital Cytogenetic Screening Laboratory. All samples we confirmed to have normal karyotypes using standard cytogenetic techniques. Samples were dissected under a microscope and separated from any decidual tissue or flecks of blood. The culture media was removed and the tissue placed in 1.5–2.0 mL micro centrifuge tubes before freezing at −80°C until DNA was extracted. To extract DNA, one 5 mm stainless steel bead and 180 µL buffer ATL (from Qiagen's DNeasy Blood and Tissue kit) were added to each CVS sample. The samples were placed in the TissueLyser (Qiagen) Adaptor set 2×24, and the TissueLyser was operated for 20 seconds at 30 Hz. The DNA was then purified using the DNeasy Blood and Tissue kit as per the manufacturer's protocol. MBCs were obtained between gestational weeks 11 and 13 from the Magee Women's Hospital Prenatal Screening lab. DNA was extracted from the MBC's using a modified protocol previously described by Iovannisci, *et al*., 2006 [22], using reagents from the MasturePure DNA Purification Kit (Epincentre Technologies, Madison, WI, Cat. No. MCD85201). Briefly, clotted blood (approximately 1 mL) was mixed with an equal volume (1 mL) of 2X Tissue and Cell Lysis Solution, votexed for 10 s and combined with 2 mL Tissue and Cell Lysis Solution (MasturePure kit) containing 25 ng/µL proteinase K. 2 mL of MPC Protein Precipitation Reagent was added to the total volume (4 mL) of the lysed sample and vortex vigorously for 10–15 sec, after which samples were cooled on ice for ≥1 hour. Cell debris were then pelleted by centrifugation (x2) for at least 30 min at ≥2000 g and supernatants transferred to new 50 mL conical tubes. DNA was precipitated in 2 volumes of isopropanol, purified by phenol/chloroform extraction and resuspended in 50 µL DNAse/RNAse free water.

### Target DNA Preparation for Agilent Microarray Analysis

The Agilent data is MIAME compliant has been deposited in the GEO database (http://www.ncbi.nlm.nih.gov/geo/) with the accession number/series record GSE23835. Genomic DNA samples (3 µg) were digested for two hours at 37°C with 50U HpaII (New England Biolabs [NEB]) in 90 µL total reaction volume using NEB buffer 4. A second aliquot of 50U, 1 µL of buffer 4, and 4 µL water were added and digestion continued overnight (total reaction volume was 100 µL). Mock digestion controls were included to monitor digestion efficiency. Following overnight digestion, reactions were digested further with 5 uL (50U) of TspRI (NEB) at 65°C for three hours. Reactions were then incubated further with 75U (0.75 µl) Exonuclease III (NEB) and incubated at 30°C for 1 hour. Enzymatic activity was then nullified by heating at 70°C for 20 min after which 50U of RecJF (NEB) were added to remove single stranded DNA. Reactions were incubated for 30 min at 37°C and inactivated at 65°C for 20 min. Reactions were then phenol-chloroform extracted and the DNA precipitated and resuspended in 21.2 µL nuclease-free de-ionized water. Finally, extracted genomic DNA was quantified and assessed for purity using a NanoDrop ND-1000 UV-VIS Spectrophotometer.

### CGH Target Labeling and Hybridization for Agilent Microarrays

Experimental and reference DNA were labeled with Cy3-dUTP and Cy5-dUTP respectively, and vice versa for dye-swaps, using a BioPrime CGH Genomic Labeling kit per the manufacturer's protocol (Agilent). Hybridization was performed in a mix containing 50 µL of human Cot-1, 52 µL of Agilent 10x blocking agent, 260 µL of Agilent 2x HiRPM hybridization buffer, and 158 µL of the labeled DNA. The hybridization mix was heated to 95°C for 3 minutes, then incubated at 37°C for 30 minutes and applied onto the active array area. Hybridization with gentle agitation was carried out at 65°C for 40 hours. After hybridization, the slides were washed in Oligo aCGH Wash Buffer 1 and Oligo aCGH Wash Buffer 2, followed by acetonitrile and Stabilization and Drying Solution (Agilent) per the manufacturer's protocol. The slides were scanned using an Agilent Scanner and the data was analyzed using Agilent Feature Extraction software 8.1 (Agilent). Visualization and comparison of the datasets were done with CGH-Analytics 3.2 (Agilent).

### Infinium Microarray Analysis

The Infinium data is MIAME compliant has been deposited in the GEO database (http://www.ncbi.nlm.nih.gov/geo/) with the accession number/series record GSE23311. The HumanMethylation27 DNA Analysis BeadChip (Illumina) allows interrogation of 27,578 CpG sites based on the NCBI CCDS database (Genome Build 36) and also targets the promoter regions of 110 miRNA genes. Bisulphite conversion of DNA was carried out using the EZ DNA Methylation™ Kit (Zymo Research Corp., CA) to convert unmethylated cytosine nucleotides to uracil. Following denaturation with 0.1N NaOH, converted DNA samples were amplified by incubation at 37°C for 20 hours in a proprietary amplification reaction mix. Amplified DNA was fragmented using vendor-supplied reagents by incubation for one hour at 37°C. Fragmented DNA sample was precipitated and resuspended in hybridization buffer. Infinium BeadChips were cleaned and activated by washing with ethanol, formamide and vendor supplied pre-hybridization buffers. DNA samples are denatured, applied to the Infinium arrays and hybridized 16–24 hours with rocking at 48°C. The BeadChip is placed into a flow-through chamber, unhybridized and non-specifically hybridized DNA was washed away and single base extension was performed on bound primers with labeled nucleotides. Hybridized DNA sample was removed by washing using proprietary buffers. Staining steps were performed to attach florescent dyes to the labeled nucleotides and the array surface sealed to protect the dyes from atmospheric degradation. The final array was scanned using an Illumina BeadArray Reader and the data analyzed using Bead Studio 2.0.

### Determination of the methylation status of CpG sites using Infinium Array Data

On an Infinium array, each targeted CpG site was interrogated by 2 probes: probe A for unmethylation status, and probe B for methylation status. The A probe signals and B probe signals were normalized separately, using the cyclic loess algorithm (Wu). We then computed the log ratio of probe B to probe A: log(B/A), as well as the beta value, which was defined as approximately B/(A+B+100), assuming A, B≥0. Both beta and log(B/A) can be used as a measurement of the methylation level of a CpG site. In particular, a CpG site was hypomethylated if the log (B/A) value of that site was significantly lower than 0. It was hypermethylated if log(B/A) is significantly higher than 0. Student's t tests were used to test if a CpG site was methylated in a group of samples, or if two groups of samples had identical methylation rates at a given CpG site. Empirical Bayesian method proposed in Smyth (2004) was used to estimate the within group variance. P values were adjusted using Benjamini and Hochberg's method to control the false discovery rate (FDR) at 5%.

### Determination of methylation status at MspI sites using custom Agilent E-Arrays

Each custom Agilent array was hybridized with an HpaII digested sample (HpaII+) against the same sample without HpaII digestion (HpaII−). If the CpG dinucleotide in an MspI site recognition site was hypomethylated, the signal from the HpaII− sample should be stronger than the signal from the corresponding HpaII+ sample. If the CpG dinucleotide in an MspI site recognition site was hypermethylated, the signal from the HpaII+ sample should be the same as the signal from the corresponding HpaII− sample. We used the log signal ratio of HpaII− to HpaII+ to measure the hypomethylation level of the CpG in an MspI site: The MspI site was hypomethylated if the value of log (HpaII−/HpaII+) for that site was significantly above 0. Similarly, we determined that one group of samples was more hypomethylated than another group at a given MspI site if the log (HpaII−/HpaII+) of that site was significantly higher in the first group than in the second group. The statistical analysis of the Agilent E-Array data was similar to the analysis of the Infinium Array data discussed above. The log (HpaII−/HpaII+) signals were normalized using the cyclic loess algorithm. Student's t tests were used to test if a CpG site was hypomethylated in a group of samples, or if two groups of samples had identical hypomethylation status at a given CpG site. Empirical Bayesian method was used to estimate the within group variance. P values are adjusted with FDR controlled at 5%.

### Determination of the Spatial Pattern of Hypomethylation in Chromosomes 13, 18, and 21

We used the sliding window approach to visualize the hypomethylation patterns of the MspI sites over the whole chromosomes, and identify regions with significantly higher methylation/hypomethylation levels. After identifying all MspI sites that hypomethylated with FDR controlled at 5%, we computed, 1) for both CVS and MBC samples, the moving average of the hypomethylation rate for each MspI site, which was defined as the percentage of hypomethyated MspI sites among the 50 MspI sites closest to that MspI site, and 2) the moving average for the difference in hypothmethylation between CVS and MBC, which was defined as, among the 50 MspI sites closest to that MspI site, the difference in the number of hypomethylated sites between CVS and MBC divided by 50. The moving averages were plotted along the whole chromosomes for the visualization of the hypomethylation pattern in CVS and MBC, and the difference in hypomethylation between CVS and MBC.

To identify the regions with distinct hypomethylation pattern, for each type of moving averages, we first ran simulations to get its empirical distribution of the moving averages under the null hypothesis that the methylation of the MspI sites is uniform over the whole chromosomes, and that whether an MspI site is methylated in CVS is totally independent of whether it is methylated in MBC. Using the estimated empirical distribution, we computed the p values for the moving average of hypomethylation level or difference in hypomethylation level at each MspI site. The p values were adjusted to control FDR at 5%. For each type of moving averages, any two MspI sites with adjusted p values ≤0.05 were merged provided there were fewer than 50 MspI sites between them (recall that the moving average value at each MspI site represents the average hypomethylation rate or the average difference in hypomethylation rate over a 50-site long region). By this approach we were able to identify, 1) the regions where the MspI sites were significantly hypomethylated than other regions of in the same chromosome, in either CVS or MBC, and 2) the regions where the MspI sites were significantly more hypomethylated in CVS vs. MBC, or significantly more hypomethylated in MBC vs. CVS, than other regions of the same chromosome.

### RNA Extraction from Tissues

Each tissue sample was combined with 1 ml Trizol (Invitrogen, Carlsbad, CA) and a 5 mm steel bead and homogenized on a TissueLyser (Qiagen, Valencia. CA) for 4 min at 30 Hz, rotating the assembly halfway through the time. The volume was transferred to a fresh tube and the cellular debris was pelleted by centrifuging at 12000 g for 10 minutes at 4°C. The homogenate was transferred to a fresh tube and incubated at room temperature for 5 min. 200 ul of chloroform was added to the sample, and then the samples were vortexed and allowed to incubate at room temperature for 5 min. The samples were then centrifuged at 12000 g for 15 min at 4°C. The aqueous phase was transferred to a fresh tube, 500 ul isopropyl alcohol was added and the samples were mixed and then incubated at room temperature for 10 min. RNA was purified using Qiagen's RNeasy Mini kit. In brief, the samples were transferred to spin columns and centrifuged at 8000 g for 15 seconds. The columns were washed once with 700 ul buffer RW1 and twice with 500 ul RPE with centrifugations for 15 seconds at 8000 g for each wash. The columns were then placed in 1.5 ml centrifuge tubes, and the RNA was eluted by adding 30 ul RNAse-free water and centrifuging for 1 min at 8000 g.

### Real Time Method

Each RNA sample was converted to cDNA using the High Capacity RNA-to-cDNA kit (Applied Biosystems, Foster City, CA) per the manufacturer's protocol. TaqManGene expression assays for the following genes: COL15A1 (Hs0026632_m1), GJA1 (Hs00748445_s1), LAMB1 (Hs01055971_m1), LUM (Hs00158940_m1), PITX2 (Hs00165626_m1), SLC16A4 (Hs00190794_m1), TFPI2 (Hs00197918_m1) and VGLL3 (Hs01013372_m1), as well as for the endogenous control GusB (Hs00939627_m1) were purchased from Applied Biosystems. For each real time PCR reaction, 1 ul cDNA, 1 ul gene expression assay, and 10 ul TaqMan gene expression master mix were combined with water in a well on the reaction plate for a total volume of 20 ul. Each reaction was run in triplicate, and each sample was also run against the endogenous control on the same reaction plate. This eliminated any differences in input DNA variation and allowed the data to be read as a relative quantity. All samples were normalized against one of the MBC samples. The real time PCR reactions were read and analyzed using the 7900HT Sequence Detection System (Applied Biosystems).

### Analysis of the Association Between the Patterns of Hypomethylation and the Pattern of Gene Expression

To analyze the relation between the methylation status of a certain type of structural components of the genome, e.g., the promoters, and the expression levels of the genes corresponding to those regions, we applied a nonparametric regression algorithm – the cubic spline regression – to the data, with the gene expression level as the dependent variable and the measurement of methylation/hypomethylation, such as the average log(B/A) ratio (for Infinium Array data) over each region, as the independent variable. F tests were used to determine if the independent variable was a significant predictor for the dependent variable, with the trace of the smoother matrix used as the degrees of freedom of the independent variable [23].

## Results

We performed a genome wide analysis of DNA methylation in first trimester CVS samples and gestational age matched MBCs. Data were generated using two high-throughput approaches: the Infinium “humanmethylation27” platform marketed by Illumina and a custom Agilent-based platform. The former is targeted towards 27,578 CpGs mostly contained within CpG islands and well characterized promoter sequences that are spread throughout the genome. Using this method we analyzed DNA samples obtained from 12 CVS samples and 12 MBC samples. Using the Agilent platform we carried out an unbiased high-throughput analysis of DNA methylation on chromosomes 13, 18 and 21. This approach was carried out on each of two pools of CVS and two pools of MBC samples as previously described[24]. This custom array contains 215,060 informative probes. Among them, 78,548 probes target 42,978 MspI/HpaII sites on chr18, with 35,570 sites targeted by a matching pair of probes. Also, 46,675 probes target 25,878 MspI/HpaII sites in chr21, with 20,797 sites targeted by a matching pair of probes. Furthermore, 89,837 probes target 49285 MspI/HpaII sites in chr13, with 40,552 sites targeted by a matching pair of probes.

Of the 27,578 CpG sites targeted by the Infinium array, we identified 563 that were hypermethylated in MBC and hypomethylated in CVS versus 155 sites that were hypomethylated in MBC and hypermethylated in CVS. These can be considered to be tissue-specific differentially methylated regions (T-DMRs). Similar analysis of the custom/Agilent microarray data identified 6311 T-DMRs across chromosomes 13, 18 and 21[24]. A significant number of these differentially methylated loci have been verified and these data reported elsewhere[24].

### CVS Genomes are Generally “More Hypomethylated” than MBC Genomes

**Figure 1:**
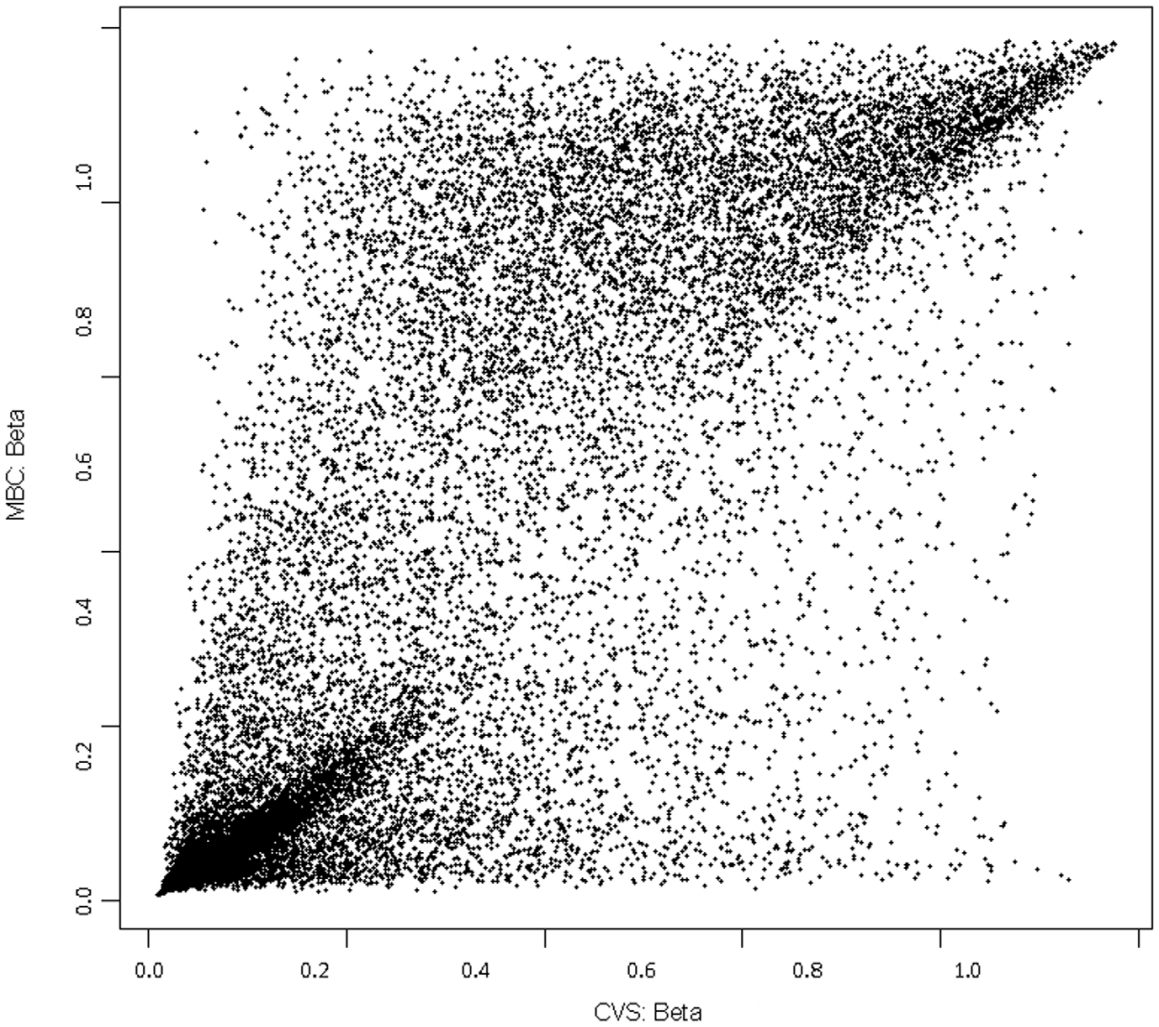
Scatter plot of genome-wide DNA methylation levels (Beta) of CVS and MBC genomes based on the Infinium data.

**Figure 2:**
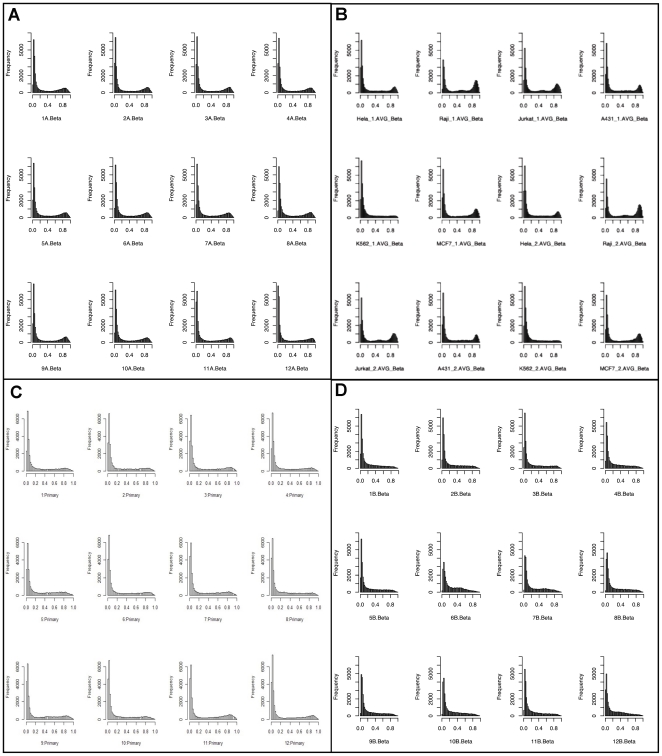
Histograms of genome wide methylation levels (Beta) in (A) 12 MBC samples (B) 12 cell lines (C) Primary ovarian tumors and (D) 12 CVS samples based on the Illumina Infinium data. MBCs, cell lines and primary ovarian tumors display a bimodal distribution of hyper and hypomethylation, CVS genomes have a dramatic reduction in the number of loci that are hypermethylated.

We identified five fold more T-DMRs that were hypomethylated in CVS versus MBC compared to those that were hypomethylated in MBC versus CVS (Figure 1). This does not appear to be an artifact since we observed the same phenomenon in both the Agilent and Illumina data sets and each of these approaches relies upon significantly different library preparation methods. Furthermore, when we plotted the frequency of methylation at specific CpG sites in MBCs using the Illumina data we found there to be a clear bimodal distribution, with large numbers of CpG sites that are either completely hypermethylated or completely hypomethylated (Figure 2A). This bimodal pattern was also evident for a variety of cell lines (Figure 2B) and primary ovarian tumor samples (Figure 2C). These samples were used for comparison because they serve as examples of both cultured and uncultured transformed cell tissue types respectively. We chose neoplastic samples for this purpose because of the previously suggested similarities between the molecular phenotype of placental tissues and tumors [25]. Data from cell lines were obtained directly from Illumina whereas the primary ovarian tumor data were obtained in our own lab as part of a separate experiment. The bimodal distribution was, however, not evident in CVS genomes, which displayed significantly fewer fully hypermethylated sites and significantly more partially methylated sites (Figure 2D).

### CVS vs. MBC T-DMRs Display Spatial Associations Within Chromosomes 13, 18 and 21

We next asked whether there were significant differences in the spatial location of T-DMRs that distinguish CVS and MBC genomes. A primary goal of this analysis was to determine whether T-DMRs are clustered together or dispersed randomly throughout the genome. This required that our analysis focused on broad differentially methylated genomic regions rather than individual T-DMRs.

**Figure 3:**
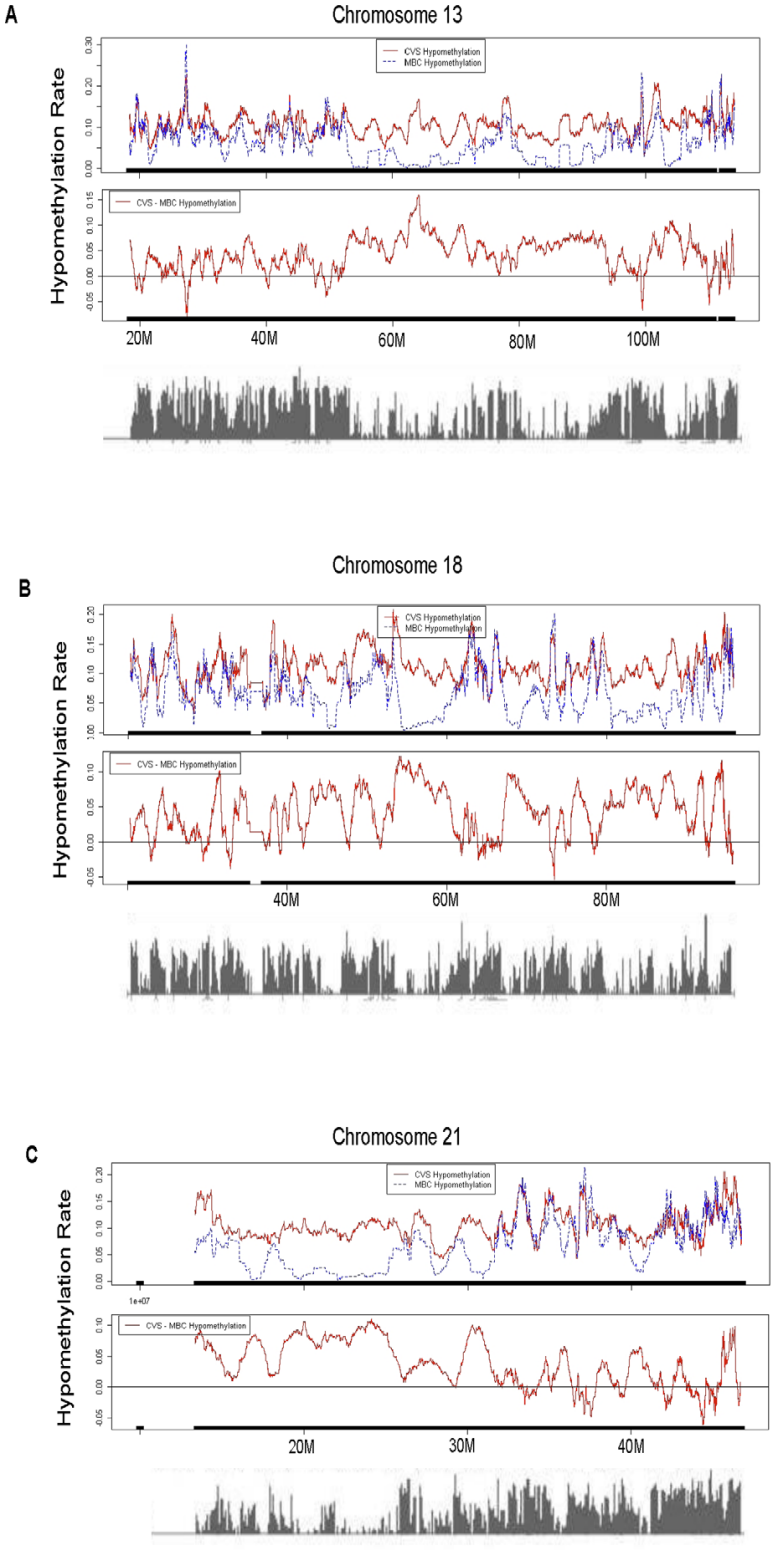
Top: Moving average of the hypomethylation levels of the MspI sites levels in CVS (solid line) and MBC (dashed line). Middle: Moving average of the difference in the hypomethylation levels of the MspI sites between CVS and MBC. The short dense vertical lines above the X axis (appearing as a solid horizontal line) in both the top and bottom panels represent locations of the MspI sites in each chromosome. Bottom: Histogram of the EST and mRNAs aligned to the chromosome generated using NCBI genome Map Viewer. (**A**) Chromosome 13. (**B**) Chromosome 18. (**C**) Chromosome 21.

**Figure 4:**
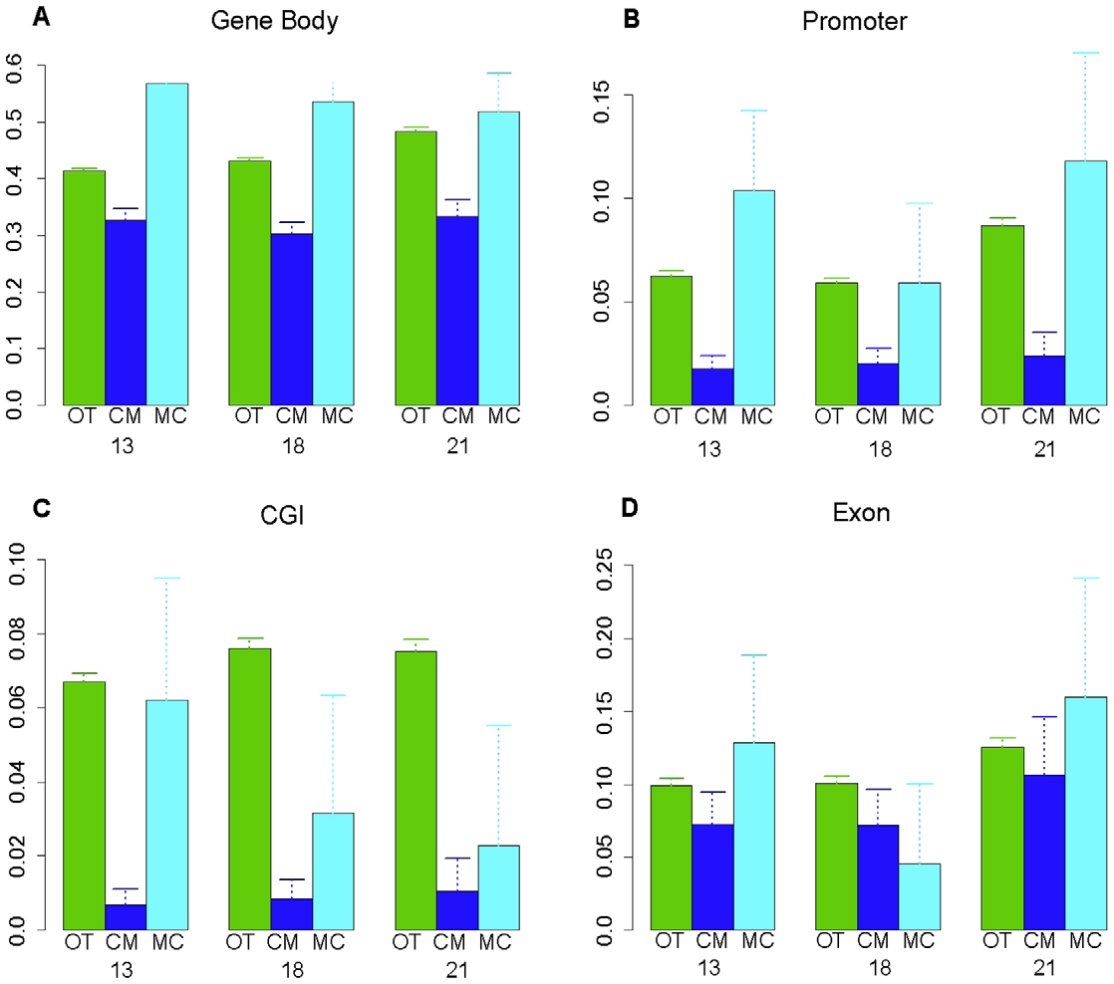
Distributions of differentially methylated and non-differentially methylated MspI sites in various structural components of the genome based on custom Agilent microarray data. OT: MspI sites not differentially methylated in CVS vs. MBC. CM: MspI sites more hypomethylated in CVS than in MBC. MC: MspI sites more hypomethylated in MBC than in CVS. Data are presented for each chromosome (13, 18, 21), and each type of MspI sites (OT, CM, MC), as the proportions of sites that are located inside (A) gene bodies (**B**) promoter regions (**C**) CGIs and (**D**) exons.

In order to identify broad regions of interest we employed the “sliding windows” approach described in Materials and Methods. Using both the Infinium and Agilent data, we observed that the distribution of broadly hypomethylated regions was very similar for both CVS and MBC genomes. However, we found that T-DMRs tend to cluster together in distinct chromosomal locations. This phenomenon was apparent in both the Infinium (not shown) and Agilent data sets, although it was more obvious in the latter (Figure 3), in which the probes were more closely spaced, thereby providing higher resolution. It is notable that regions dense in T-DMRs were also those that encode the fewest numbers of expressed sequence tags and mRNAs (compare the top and middle panels of Figures 3A, 3B and 3C with the corresponding bottom panel). This suggests that T-DMRs are more likely to be found outside coding regions of the genome, a finding that is corroborated by the data presented in Figure 4 (see below).

### T-DMR Clusters are enriched for Developmentally Significant Transcription Factors

We next explored the possibility that there was a functional basis for the spatial clustering of T-DMRs. We approached this by looking for correlations between these regions and their physical relationship to known genes. Specifically we wanted to determine whether regions where T-DMRs are clustered contained an over-representation of genes involved in particular networks or GO (gene ontology) functions. Sliding windows (see above) that showed statistically significant differences between CVS and MBCs were mapped to gene bodies and promoters. Specifically, we identified regions that were a) hypomethylated in CVS versus MBC or b) hypomethylated in MBC versus CVS.

We found that gene bodies and promoters that overlap broadly hypomethylated regions in MBCs (relative to CVS) were highly enriched for genes whose expressions are associated with the regulation of gene expression. Specifically, we identified regions hypomethylated in MBCs relative to CVS that overlapped with a total of twenty-one gene bodies. Furthermore, the functions of only sixteen of these have been previously characterized and a total of thirteen (81%) were identified as encoding transcription factors. These are listed in Table 1. Notably, a significant number of these genes encode transcription factors that have functional significance in the context of development and many of these are aberrantly methylated at the DNA level in a variety of tumors[26], [27], [28], [29], [30]. To put this finding in context, we identified all the genes in Table 1 that include a gene ontology designation (GO) of “Development”. Of 20 GO annotated genes, only 9 have “development” in their GO terms. These genes are identified in Table 1 by an asterisk. Strikingly we discovered that there are a total of 786 genes on chromosome 13, 18, and 21 that have GO annotations, and only 81 of them have “development” in their GO terms. We used a two sided Fisher exact test against the null hypothesis that the proportion of genes related to development among the hypermethylated MBC genes is the same as among all the other genes on chromosomes 13, 18 and 21. We discovered that there was a highly significant over-representation of genes involved in development amongst spatially clustered T-DMRs that are hypomethylated in MBCs versus CVS (p = 0.00005574).

**Table 1:** Genes Identified Within Broadly Hypomethylated Regions of MBC Versus CVS.

*Gene Names*	*Symbol*	*Type*	*Active Location*
Chromosome 18 open reading frame 18	C18ORF18	Other	Unknown
Caudal type homeobox 2	CDX2*	Transcription regulator	Nucleus
Collagen, type IV, alpha 2	COL4A2	Other	Extracellular Space
Deleted in lymphocytic leukemia 2 (non-protein coding)	DLEU2	Other	Unknown
GATA binding protein 6	GATA6*	Transcription regulator	Nucleus
GS homeobox 1	GSX1	Transcription regulator	Nucleus
Neurocanthocytosis	NA	Other	Unknown
Neurocanthocytosis	NA	Other	Unknown
One cut homeobox 2	ONECUT2*	Transcription regulator	Nucleus
Protocadherin 17	PCDH17	Other	Unknown
Pancreatic and duodenal homeobox 1	PDX1	Transcription regulator	Nucleus
POU class 4 homeobox 1	POU4F1	Transcription regulator	Nucleus
RAB20, member RAS oncogene family	RAB20	Enzyme	Cytoplasm
Receptor-interacting serine-threonine kinase 4	RIPK4	Kinase	Nucleus
Ring finger protein 219	RNF219	Other	Unknown
Sal-like 3 (Drosophila)	SALL3*	Other	Nucleus
Single-minded homolog 2 (Drosophila)	SIM2	Transcription regulator	Nucleus
SRY (sex determining region Y)-box 1	SOX1*	Transcription regulator	Nucleus
SRY (sex determining region Y)-box 21	SOX21	Transcription regulator	Nucleus
Zinc finger protein 161 homolog (mouse)	ZFP161	Other	Nucleus
Zic family member 2 (odd-paired homolog, Drosophila)	ZIC2*	Transcription regulator	Nucleus

### T-DMRs Are More Likely to be Located Outside CpG Islands, Promoters and Gene Bodies

Given the bias towards less methylation in CVS versus MBC genomes, we explored the relationship between T-DMRs and their genomic locations in more detail. Because the Infinium array generally only targets CpG sites within known CGIs and/or promoter sequences we focused on data generated using the custom Agilent oligonucleotide microarray that is targeted towards every HpaII/MspI recognition sequence (CCGG) on chromosomes 13, 18 and 21 [31]. This platform has the advantage that probes are not specifically targeted towards known promoters and/or CGIs but instead are distributed in an unbiased fashion. This allows the identification of methylated CpG sites and T-DMRs that occur in other genomic regions such as gene bodies and regions that are not known to encompass functional genes.

First, we compared the locations of T-DMRs where CVS was hypomethylated relative to MBC with the locations of T-DMRs where the methylation patterns of the two tissues do not show any significant difference. We found that, using the Fisher's exact test, for all three chromosomes, these T-DMRs were significantly more likely to be outside a gene body than inside a gene body (Figure 4A). Furthermore, these T-DMRs were significantly more likely to be outside a promoter than inside a promoter (Figure 4B) and more likely to be outside than inside an exon (Figure 4D). In addition, we found that, for all three chromosomes, T-DMRs within which CVS was hypomethylated compared to MBC were up to 10 times less likely to be inside a CGI (Figure 4C). This is significant because it suggests that tissue specific methylation is more likely to occur in regions where CpG sites are not required to be strictly hypermethylated or hypomethylated. This can be explained by the fact that CGIs are, by definition, regions where CpGs are hypomethylated [32]. Therefore, they are unlikely to be common sites of tissue specific methylation.

We also carried out the opposite analysis to that described above. Specifically we compared the locations of T-DMRs in which MBCs were hypomethylated (relative to CVS) with the genomic regions in which the methylation patterns of the two tissues did not show significant difference. We found that, generally, these T-DMRs were less likely to exist inside CGIs (Figure 4C) and exons (Figure 4D), and slightly more likely to exist inside gene bodies and promoter regions (Figure 4A and B respectively). However, we note that the results are much less significant than those discussed above, mainly because we found much fewer (812) sites where MBC was hypomethylated compared to CVS than the 5,499 sites where CVS was hypomethylated compared to MBC[24].

### Hypomethylated Regions of the CVS and MBC Are More Likely to be in CGIs, Promoters and Exons

**Figure 5:**
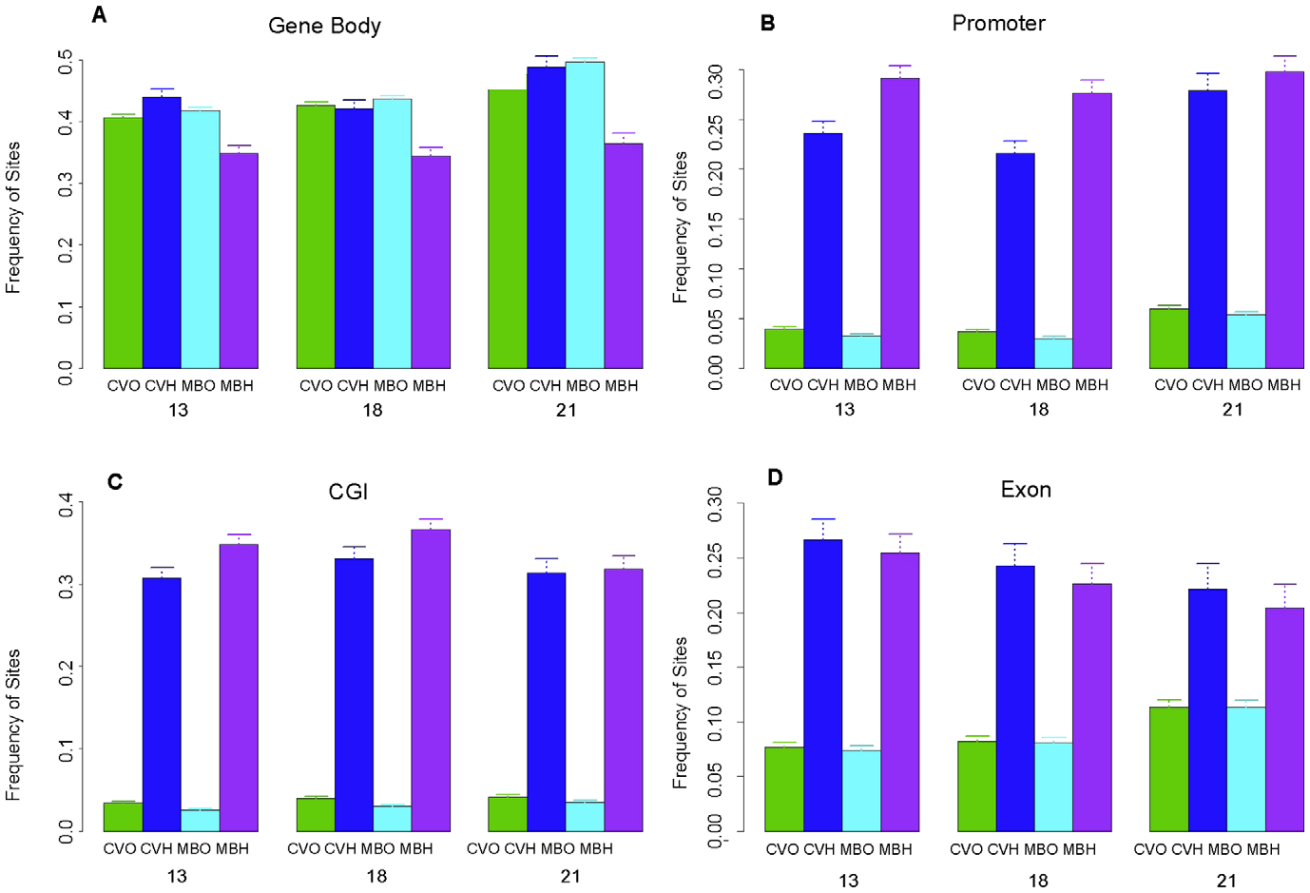
Distributions of hypomethylated and non-hypomethylated MspI sites in CVS and MBC tissues in various types of genomic regions based on custom Agilent microarray data. CVO: MspI sites not hypomethylated in CVS. CVH: MspI sites hypomethylated in CVS. MBO: MspI sites not hypomethylated in MBC. MBH: MspI sites hypomethylated in MBC. Data are presented for each chromosome (13, 18, 21) and each type of MspI site (CVO, CVH, MBO, MBH), as the proportion that are located inside (**A**) gene bodies (**B**) promoter regions (**C**) CGIs (**D**) exons.

We next considered CpGs that were hypomethylated in CVS (relative to the rest of the CVS genome) and/or hypomethylated in MBCs (relative to the rest of the MBC genome). Those sites in gene bodies showed no clear pattern in CVS whereas such sites in MBC were slightly more likely to be outside (than inside) gene bodies (Figure 5A). The pattern for promoters was very clear. Using the Fisher's exact test, we found that hypomethylated sites were much more likely to be inside promoters than other sites. This was true for both CVS and MBC (Figure 5B). The patterns for CGIs and exons were similar to the pattern for promoters. Specifically, hypomethylated sites were much more like to be inside CGIs or exons, as oppose to outside CGIs or inside introns, than the non-hypomethylated sites. This was true for both CVS and MBC (Figure 5C and D). We also found that sites hypomethylated in MBC were even more likely than in CVS to be inside (as oppose to outside) a promoter or CGI, which partly explains why sites more hypomethylated in CVS than in MBC were more like to be outside of CGI and promoter than sites more hypomethylated in MBC than in CVS.

### Pathway Analysis Reveals that T-DMRs are Enriched for Distinct Functional Groups

**Figure 6:**
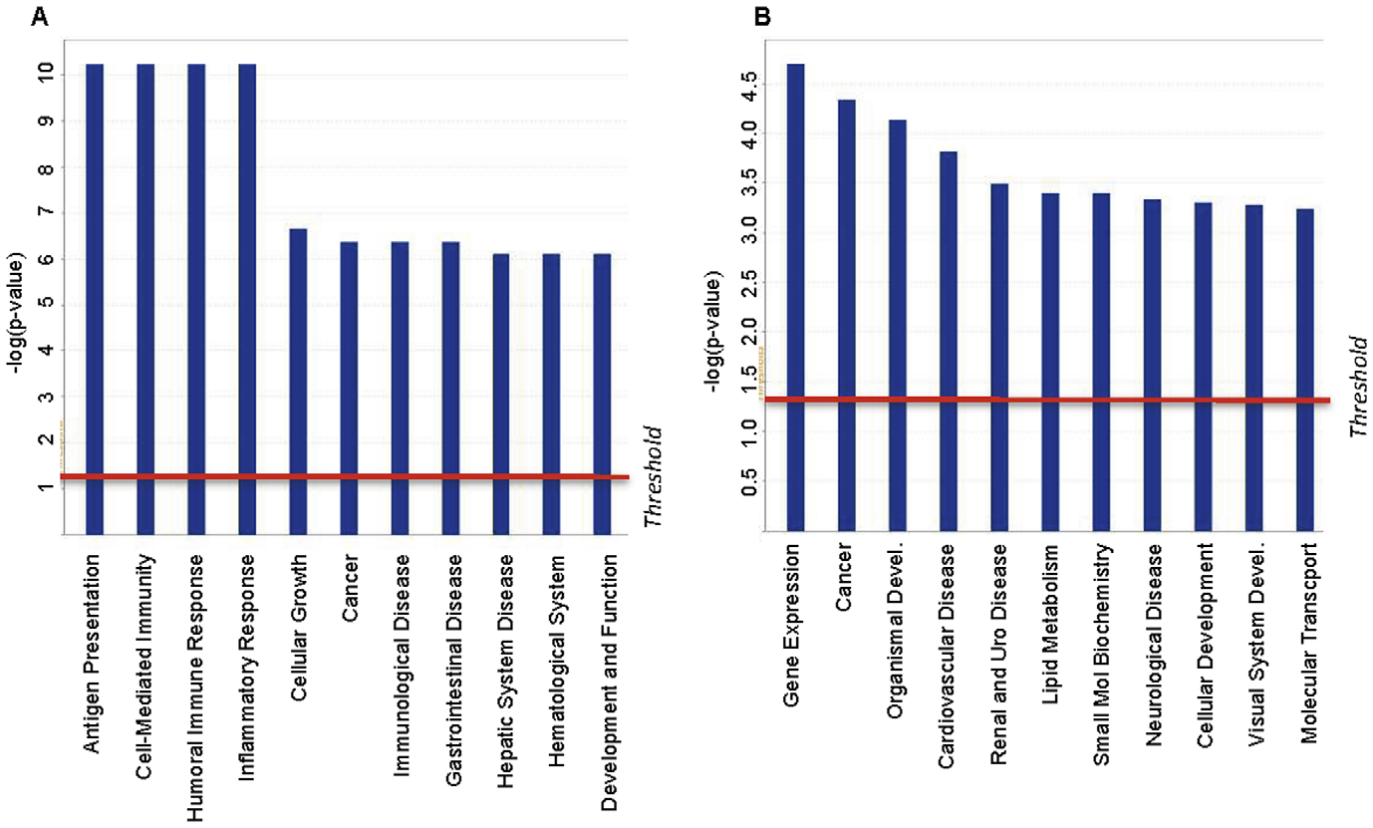
Biological Functions of genes hypomethylated in MBC versus CVS (A) and vice versa (B) Identified Using Ingenuity Pathways Analysis Software.

To further explore the relationship between T-DMRs and gene function we analyzed the Illumina data using Ingenuity Pathways Analysis (IPA) software. Specifically, we performed a gene ontology analysis of T-DMRs where CpGs are hypomethylated in MBC versus CVS and vice versa. We found that T-DMRs hypomethylated in MBC versus CVS were significantly biased towards genes that are involved in tissue specific leukocyte function. For example, the top networks identified in IPA were heavily biased towards immune function. These IPA-designated enriched networks are listed in Table S1. Similarly, the top IPA-identified biological functions (Figure 6A and Table S2) were “Antigen Presentation”, “Cell Mediated Immune Response” and “Humoral Immune Response”. These findings suggest that T-DMRs that are hypomethylated in the promoters of the MBC genome (compared to CVS) have strong functional significance.

This apparent relationship between tissue function and T-DMR profile was not so clear when we performed the same analysis on T-DMRs that are hypomethylated in the promoters of the CVS genome (compared to MBC). It should be noted, however, that we identified more than three times as many high scoring networks amongst the hypomethylated CVS T-DMRs than their hypomethylated MBC counterparts. This may reflect both the broad range of biological functions performed in/by CVS and the fact that it contains multiple distinct cell types. These factors likely conspire to complicate the task of identifying distinct pathways and biological functions. IPA-designated enriched networks for these data are listed in Table S3. High scoring IPA-designated biological functions (Figure 6B and Table S4) include “Gene Expression”, “Cancer” and Organismal Development”.

### Functional Groups Enriched in T-DMRs are also Enriched in Differentially Transcribed Genes

**Figure 7:**
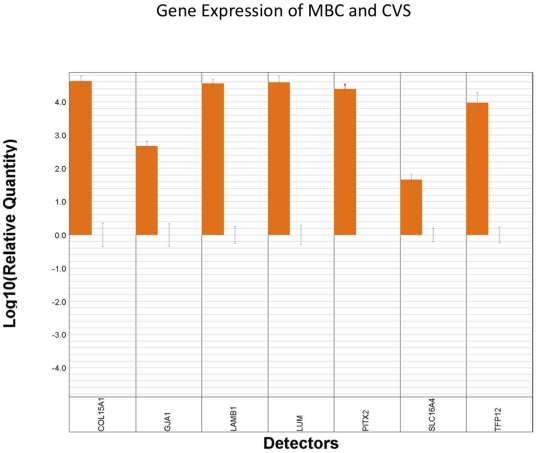
Real time PCR analysis was performed using total RNA samples from MBC (n = 6) and CVS (n = 6) to detect transcript levels encoded by the following genes: COL15A1, GJA1, LAMB1, LUM, PITX2, SLC16A4, TFPI2 and VGLL3. Each reaction was run in triplicate against an endogenous control (GUSB) and normalized against one of the MBC samples. The median logRQ was plotted for each gene.

Given the potential for DNA methylation patterns to be intimately associated with gene expression, we sought to determine whether T-DMRs identified in the Illumina data were also present as tissue specific differentially transcribed genes (TDTs). Thus, we analyzed Affymetrix gene expression microarray data to identify CVS- and MBC-specific TDTs and then analyzed these data using IPA. A sub-set of TDTs was validated using quantitative real time PCR. These data, which demonstrate tissue specific gene expression in CVS, are presented in Figure 7. We found very little overlap between T-DMRs and TDTs. Specifically, we found only 6 genes (of a total of 207) that were both more highly expressed AND contained CpGs that were hypomethylated in MBC versus CVS. These were CD48, CD52, CMTM2, CST7, LYZ and NFE2. No overlap was found between genes that were more highly expressed AND hypermethylated in MBC versus CVS. Similarly, we found 14 genes (of a total of 643) that were both more highly expressed AND hypomethylated in CVS versus MBC. These were ANGPT2, CDH1, COL3A1, CRIM1, CSH2, ENPEP, GCM1, H19, INSL4, KRT8, LGALS14, PGM3, SLC16A4 and STS. No overlap was found between genes that were more highly expressed AND hypermethylated in CVS versus MBC. The fact that such minimal overlap was seen between data sets was not the result of minimal overlap between the DNA methylation and gene expression array platforms, which contain 11,337 common genes. It may, however, be a consequence of our rather stringent approach to selecting TDTs (see Materials and Methods), which resulted in a relatively short list of candidate genes. However, when we compared pathway analysis data obtained using IPA for genes that were both more highly expressed AND hypermethylated in MBC versus CVS, we found a striking overlap in enriched networks and biological functions, despite the minimal overlap in specific genes. As shown in Table S5, the most significant IPA-designated networks identified amongst genes whose expressions were elevated in MBC relative to CVS overlap closely with those identified among genes hypomethylated in MBC versus CVS (Table S1). Similarly overlapping were the IPA-designated biological functions, which can be seen by comparison between Tables S2 and S6. Such overlap was present but not so obvious when networks (Table S7) and biological pathways (Table S8) derived from lists of genes whose expressions were elevated AND hypomethylated in CVS versus MBC were observed (compare to Tables S3 and S4 respectively).

### T-DMRs are Correlated with Levels of Gene Transcription

**Figure 8:**
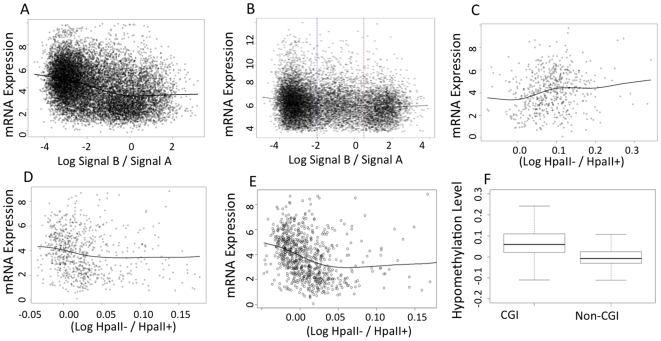
Genome wide relationship between gene expression and promoter methylation for (A) CVS and (B) MBC. The X axis is the average log ratio of signal B to signal A of probes targeting each probe on the Infinium array. Negative values indicate hypomethylation and positive values hypermethylation. The Y axis is the log2 gene expression level. The solid line represents the fitted values of the nonparametric regression of the gene expression level against the methylation level. The two dashed vertical lines mark clusters of hypomethylated and hypermethylated genes. **C–E.** Relation between CVS gene expression and hypomethylation level of various types of genomic regions in chromosomes 13, 18, and 21. The X axis is the hypomethylation level of various genomic regions, measured as the log ratio of the signals from the control samples to the signal of the HapII digested samples averaged over the probes targeting the same genomic region. The Y axis is the log2 gene expression level. The solid line represents the fitted values of the nonparametric regression of the gene expression level against the hypomethylation level of various types of genomic regions. (**C**) Relationship between CVS gene expression and promoter hypomethylation in chromosomes 13, 18, and 21. (**D**) Relationship between CVS gene expression and gene body hypomethylation in chromosomes 13, 18, and 21. (**E**) Relationship between CVS gene expression and the non-CGI gene body hypomethylation in Chromosomes 13, 18, and 21. (**F**) Box plots of the hypomethylation level of the MspI sites inside gene bodies in Chromosomes 13, 18, and 21, as determined by custom Agilent arrays. Left: MspI sites inside gene bodies and CGIs. Right: MspI sites inside gene bodies but outside CGIs.

To explore the global relationship between gene expression and DNA methylation patterns we downloaded gene expression microarray data obtained using mRNA derived from CVS tissue [33] and MBCs from NCBI Gene Expression Omnibus (GEO) (accession number GSE14771). Notably, and in keeping with the observation that CVS genomes are more hypomethylated than MBC genomes, we found there to be twice as many mRNAs over-expressed in CVS versus MBC than vice versa (Table S9). We compared the CVS mRNA transcription profile derived from these Affymetrix data with the DNA methylation profile derived from the Infinium data and found a significant negative correlation between the degree of promoter methylation and the expression level of the corresponding gene (Figure 8A). For these purposes, the promoter region was defined as 1500 upstream to 1500 downstream of the transcriptional start site (TSS), as in Rakyan et al (2008) [34]. All CpG sites targeted by the Illumina array were located within this range. Specifically, we estimated expression levels and promoter methylation rate for 13,847 genes. The expression level of a gene was obtained by averaging the log signal intensity of the probe sets targeting that gene over the 8 normal CVS samples. The promoter methylation rate was estimated by averaging the log ratio of B probe to A probe–log(B/A)–over the 12 CVS samples. The log(B/A) is an indicator of the level of methylation: the higher the methylation level, the higher the value of log(B/A). Using the Infinium methylation data, we found that the correlation between the expression level and log(B/A) is −0.35 (p value of t test<2.2×10^−16^). Moreover, the relationship between the expression level and methylation level is nonlinear. We ran a nonparametric regression and found that, when the methylation level is less than 50% (log B/A<0), there is a linear negative relationship between methylation and expression. When methylation level is greater than 50% (logB/A>0) however, methylation and expression is uncorrelated. In Figure 8A, the solid line shows the relationship between gene expression levels and DNA methylation in CVS based on the Infinium methylation data. The above trends were similarly present in the relationship between DNA methylation and mRNA transcription in MBCs (Figure 8B), though not as strong as in CVS.

To gain further insight into the relationship between gene expression and methylation, we compared the CVS gene expression profile with the DNA methylation profile for the 3 chromosomes 13, 18, and 21 based on the custom Agilent arrays. We estimated the hypomethylation level of each MspI/HpaII site in the three chromosomes by determining the log ratio of the signal of the control samples to the signal of samples digested with HpaII: log(HpaII−/HpaII+). Unlike the Infinium arrays, which targeted only CpG sites inside the promoter regions, the custom Agilent arrays targeted CpG sites at high density all over the three chromosomes. This allowed us to determine how the methylation patterns of the different structural components of the genome were related to the gene expression profile.

For genes in chromosomes 13, 18, and 21, we found a positive correlation of 0.194 (p value of the t test = 2.126×10^−06^) between the hypomethylation level of their promoter regions and their expression level (Figure 8C), where the promoter region was defined as 1500 upstream and 1000 downstream of the TSS. This finding agrees with the previous analysis of the relationship between DNA methylation and gene expression using the Illumina data. In Figure 8C, the solid line shows how the expression level relates to the hypomethylation level of the promoter regions chromosomes 13, 18, and 21. Furthermore, we found a negative correlation of −0.111 (p value of t test = 0.0018) between the hypomethylation level of the gene body and the expression level, where “gene body” includes both exons and introns (Figure 8D). The relation between hypomethylation and expression is nonlinear. When the genes are moderately or highly hypomethylated (log(HpaII−/HpaII+)>0.03), hypomethylation level and expression level are uncorrelated. When the genes are only weakly or not hypomethylated (log(HpaII−/HpaII+)<0.03), there is a significant negative correlation between hypomethylation level and expression. In Figure 8D, the solid line shows how the expression level relates to the hypomethylation level of the gene bodies in chromosomes 13, 18, and 21. This relationship is more pronounced when gene bodies containing CGIs are removed from the analysis (Figure 8E). Finally, it can be seen in Figure 8F that among the MspI sites inside gene bodies, the hypomethylation levels are higher for those inside CGIs, compared to those outside CGIs.

### The Relationship Between DNA Methylation Levels and Gene Expression are Dependent on Promoter CpG Frequency

**Figure 9:**
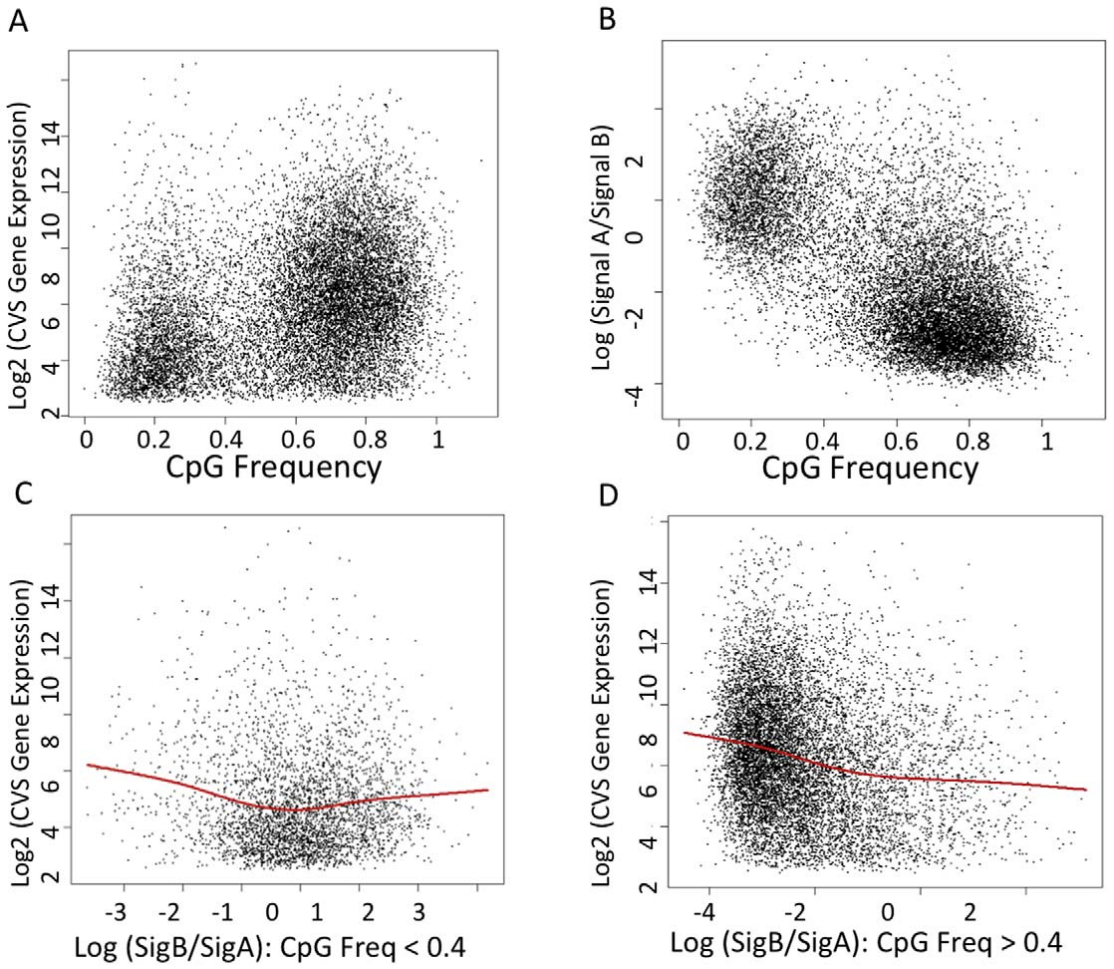
Relationships between promoter methylation and expression in CVS samples in the context of promoter CpG frequency (A) X axis: CpG frequency (defined as the observed CpG/expected CpG) of the promoter region of each gene. Y axis: Log2 gene expression levels in CVS (**B**) X axis: CpG frequency of the promoter region of each gene. Y axis: Log ratio of signal B to signal A averaged over probes targeting the promoter region of each gene in CVS. **C–D**: Relationship between transcription level and promoter region methylation level. X axis: Log ratio of signal B to signal A averaged over probes targeting the promoter region of each gene. Y axis: CpG frequency of the promoter region of each gene. Solid lines represent the fitted values of the nonparametric regression of the transcription level against methylation level. (**C**) Plot of genes whose promoter regions have a CpG frequency<0.4. (**D**) Plot of genes whose promoter regions have a CpG frequency>0.4.

Figure 9 illustrates the relationships between promoter methylation and expression in CVS samples in the context of promoter CpG frequency. As shown in Figure 9A the genes form two clusters based on their promoter CpG frequency (observed CpG/expected CpG) and genes with high CpG frequency promoters tend to be more highly expressed. Figure 9B shows that the genes also form two clusters based on their promoter CpG frequency with respect to promoter methylation levels. Genes in the low CpG frequency cluster tend to be hypermethylated, whereas the genes in the high CpG frequency cluster tend to be hypomethylated. Figures 9C and D show the nonlinear relationship between expression and methylation in each of the two clusters identified in Figure 9B. When CpG frequency is ≤0.4 (Figure 9C), the relationship between methylation and expression shows no clear pattern, whereas when CpG frequency is > 0.4, for genes with hypomethylated promoters, gene expression is positively correlated with hypomethylation level, or equivalently, negatively correlated with methylation level (Figure 9D). The data presented in Figure 9D are consistent with our previous observations of the relationship between promoter methylation and gene expression (without considering CpG frequency) based on data obtained using both the Agilent array and Infinium arrays (Figure 8A,B,C). That is, promoter methylation level is negatively correlated with gene expression, especially for genes with relatively hypomethylated promoter.

## Discussion

We present a comprehensive epigenetic analysis of the placental chorionic villus (CVS) and gestational age matched maternal blood cells (MBC) at the level of DNA methylation. In addition to providing detailed insight into the structure and organization of the CVS and MBC methylomes in the context of promoters, CpG islands and gene bodies, we present novel findings relating the methylation levels of these genetic elements to gene expression levels, biological function and primary DNA sequence.

One fundamental difference between the CVS and MBC genomes is the bias towards a hypomethylated state in the former. Related to this is that fact that, unlike the differentiated adult tissues and tumor samples we investigated, CVS genomes do not have a bimodal distribution of hyper- and hypomethylated sites. We found these to be global phenomena, for which the biological basis is unclear. These observations may however be related to the fact that very early gestational trophoblast stem cells display a hypomethylated genomic state that is consistent with a semi-pluripotent phenotype[35]. This is supported by the fact that trophoblast lineages are thought to retain pluripotency for some time after implantation[36] and the observation that relative hypomethylation in CVS versus MBC in the first trimester is lost by the third trimester[37]. The relative hypomethylation of CVS versus MBC may also be related to the highly proliferative and invasive nature of this tissue and its requirement for a highly active and complex transcriptional state. Interestingly, Papageorgiou et al., (2009)[37] recently reported similar findings using an immunoprecipitation-based approach although this was not as pronounced as in our data and was not the case for all chromosomes.

The spatial association of broadly hypomethylated regions observed in CVS and MBC genomes is intriguing. It is conceivable that such differentially methylated regions play a role in the regulation of expression of functionally related genes such as those identified in Table 1. The fact that some of these genes are aberrantly methylated at the DNA level in a variety of tumors[37] is notable given the previously noted link between the “molecular phenotype” of tumors and early mammalian development[25].

The discovery that T-DMRs are more likely to be located outside promoters and gene bodies suggests that tissue specific differences in DNA methylation are not limited to regions of the genome that have traditionally been associated with tissue specific gene expression. This raises a number of possibilities. For example, it may be that genomic regions outside gene bodies and promoters are important for the regulation of tissue-specific gene expression. It is also possible that tissue specific differences in DNA methylation are of minimal functional significance in the context of tissue specific gene expression. The observation that T-DMRs are highly unlikely to be found in CpG islands can be explained by the fact that CpG islands are, by definition, regions where CpGs are hypomethylated[19]. Therefore, they are unlikely to be common sites of tissue specific methylation.

The fact that hypomethylated regions of the CVS and MBC genomes are highly likely to be located inside (compared to outside) promoters and CpG islands (Figure 4B and 4C) is consistent with previous analyses in other tissues[34]. It is interesting, however, that this phenomenon is also apparent in exons, particularly given the fact that we also found a positive correlation between gene body methylation and gene expression. It would be interesting therefore to specifically identify those exons that do NOT appear to be hypomethylated and determine if these are strongly associated with gene expression.

The notion that there is an organized functional relationship between DNA methylation and tissue-specific biological function is supported by the data presented in Figure 6A in which we identified a clear correlation between tissue specific methylation and cell type specific biological function in MBCs. The fact that this relationship was less clear for CVS is likely to be related to the fact that CVS is both more hypomethylated and also more transcriptionally active. Similarly, it is interesting that, despite almost no overlap in gene specific DNA methylation and transcription, we found “functional” overlap identified by IPA analysis (Table S1). This suggests that DNA methylation patterns in CVS and MBC may provide a permissive framework within which the potential for gene-specific expression is enabled but not necessarily actualized at the time of sample collection. Such a relationship might further explain why we found it more difficult to identify a clear correlation between tissue specific methylation and cell type specific biological function in CVS. Specifically, it may be that the dynamic complexity of CVS transcription throughout early gestation requires a broadly hypomethylated permissive DNA methylome to enable appropriate gene expression.

Our discovery that the DNA methylome is broadly related to gene expression patterns is consistent with previous observations in other tissues but novel in the context of global analysis in CVS[10], [38], [39]. Unlike previous studies, however, we found that the negative correlation between the degree of promoter methylation and the expression level of the corresponding gene was non-linear, only being evident when the methylation level is less than 50%. This was true for both CVS and MBC. There was also a negative correlation between the hypomethylation level of the gene body and expression level and again we found this to be nonlinear. Specifically, when the gene bodies are moderately or highly hypomethylated, hypomethylation level and expression level are uncorrelated, whereas when the gene bodies are only weakly or not hypomethylated there is a significant negative correlation between hypomethylation level and expression. These data extend previous observations by providing preliminary insight into the subtleties of the relationship between DNA methylation and gene expression.

The finding that CVS and MBC promoters are naturally clustered into two groups according to CpG frequency is highly significant. Specifically, for the promoters in the high CpG frequency group, methylation level is negatively correlated to the gene expression level, especially for the hypomethylated promoters, whereas no clear relation between methylation and expression could be derived for the promoters in the low CpG frequency. This contrasts previous observations by Beck et al., (2008) who found that the relationship between DNA methylation and gene expression was largely independent of CpG frequency[38]. It may be that these differences can be explained by the use of different molecular approaches. However, it is important that these observations are further explored.

In summary, we have performed the first comprehensive structural and functional analysis of the early gestational human placental epigenome at its maternal interface. Our data provide detailed insight into global DNA methylation patterns and their relationship to gene expression in the human chorionic villi. Our data also provide a foundation for the molecular characterization of gestational diseases such as preeclampsia, which have a placental component, and the development of non-invasive biomarkers for their minimally invasive detection and management.

## Supporting Information

Table S1 IPA biological network analysis of genes hypomethylated in MBC versus CVS.
(0.04 MB PDF)

Table S2 IPA biological pathway analysis of genes hypomethylated in MBC versus CVS.
(0.36 MB PDF)

Table S3 IPA biological network analysis of genes hypomethylated in CVS versus MBC.
(0.07 MB PDF)

Table S4 IPA biological pathway analysis of genes hypomethylated in CVS versus MBC.
(0.31 MB PDF)

Table S5 IPA biological network analysis of genes over-expressed in MBC versus CVS.
(0.05 MB PDF)

Table S6 IPA biological pathway analysis of genes over-expressed in MBC versus CVS.
(0.37 MB PDF)

Table S7 IPA biological network analysis of genes over-expressed in CVS versus MBC.
(0.05 MB PDF)

Table S8 IPA biological pathway analysis of genes over-expressed in CVS versus MBC.
(0.33 MB PDF)

Table S9 List of genes that are differentially expressed between CVS and MBC.
(0.04 MB PDF)
