# Supplementary material for: Structural and Regulatory Characterization of the Placental Epigenome at Its Maternal Interface
Source: PLoS One. 2011 Feb 23;6(2):e14723. doi: 10.1371/journal.pone.0014723 (PMC3044138; doi:10.1371/journal.pone.0014723)
Supplement: Table S1 — IPA biological network analysis of genes hypomethylated in MBC versus CVS. (0.04 MB PDF) [file pone.0014723.s001.pdf]

Hypomethylated in MBC versus CVS **Table S1**

¬© 2000-2009 Ingenuity Systems, Inc. All rights reserved.

ID	Top Functions	Molecules in Network	Score	Focus Molecules
1	Antigen Presentation, Cell-mediated Immune Response, Humoral Immune Response	BST2,CST7,CTSZ (includes EG:1522),DNMT1,DUSP2,Erm,FUT7,ICAM3,IFN Beta,Ifn gamma,IFN TYPE 1,IL10,IL27,IL12 (complex),IL18BP,IL1R2,Interferon alpha,JAK,KLRG1,LGALS7,LSP1,NALP,NFkB (complex),NLRP3,NLRP12,OSM,PKP1,PTGDR,PYCARD,SELPLG,SPN,STAT,STAT5A,STAT5a/b,TLR9	49	24
2	Cell Signaling, Immunological Disease, Infectious Disease	ALOX15B,Ap1,BCR,CD3,CD6,CD22,CD52,CD93,CD79B,DOK2,ERK,Fcer1,HOXA7,Jnk,LAT,NCF1,NCF4,Nfat (family),P38 MAPK,PADI4,Pkc(s),PLC gamma,PTPN7,PTPRCAP,Ras,RASSF1,SH2D3C,Sod,SOD3,SPI1,TCR,TNFSF8,TNK1,VAV,VAV1	37	21
3	Cancer, Hematological Disease, Immunological Disease	AKT1,AOC2 (includes EG:314),ARHGAP10,beta-estradiol,BLVRB,C6ORF25,CAMKK1,CD84,CD300A,CLIC1,DOK3,EPO,FCRL4,FXYD5,GTP,GUCY1B2,GUCY1B3,IGKV1-117,INPP5D,KLRA1 (includes EG:10748),LAPTM5,MMP2,NUAK1,PGLYRP1,PIK3IP1,PITPNM2,PTK2B,PTPN11,RASIP1,RERG,SPOCK1,TBC1D10C,TRAF3IP3,WFIKKN2,YWHAZ	24	14
4	Infectious Disease, Respiratory Disease, Cell Morphology	C3ORF15,C8ORF4,Caspase,CEBPE,Collagen(s),DAB2IP,ERK1/2,Gngt2-Transducin beta (cone),Histone h3,Hsp90,IGK,IL1,Insulin,ITGAM,KCNC2,KCNJ16,LYZ,Mapk,MYBPC2,NFE2,noladin ether,NR1I2,OXT,PI3K,Pka,PLC,POR,PRKAR1B,RAB32,RNA polymerase II,S100A4,S1PR4,SMOC2,TNP2,TRAF1	22	13
5	Dermatological Diseases and Conditions, Genetic Disorder, Neurological Disease	ATG4C,ATP10A,C14ORF106,CASP8,CBARA1,CHCHD2,E2F1,FOXA2,GIP2,GOLT1B,GPX2,HNF4A,HPN,HTT,IFNA2,KCNC3,ME1,NOX4,ONECUT2,PPP1R16B,RFFL,SAMHD1,SH3BGRL2,SLC15A3,SLC22A18AS,THRB,TMC6,TMC8,TMEM126B,TNFAIP8L2,TP53,ZFYVE19,ZNF22,ZNF175,ZNHIT3	21	13
6	Cancer, Cellular Movement, Tumor Morphology	AFF3,BAMBI,CD37,CD53,CES2 (includes EG:234671),CHAD (includes EG:1101),CLEC4A,COL7A1,CRYBB1,CXCR6 (includes EG:10663),EMILIN1,FERMT1,GPR21,GPRC5A,H2AFY,HAT1,ICAM4 (includes EG:3386),IL13,IL15,IRS1,ITGB1,KLF16,KRT8,KRT81 (includes EG:3887),LMO2,LTC4S,MGEA5,MN1,MPHOSPH9,NCR3,NHLH1,NPTXR,PDGF-CC,SP1,TGFB1	20	12
7	Cell Cycle, Cancer, Cell-To-Cell Signaling and Interaction	1-oleoyl-lysophosphatidic acid,5-HTR3,ACAP1,ADA,ADARB1,AIF1,ARFRP1,BRCA1,C3,CCNB1,CD48,COX6A1,DGKA,DL glyceraldehyde,GAMT,GCDH,GPR77,GRB2,HRH1,lysophospholipids,MGAT1,MSH3,MYC,MYO3B,NAPEPLD,PERP,Pld,PLD3,PLD4,PTPRO,RPS15A,SAR1B,SLIT1,SOD2,UBR2	15	10
8	Amino Acid Metabolism, Drug Metabolism, Lipid Metabolism	ALB,FAM113B	2	1
9	Digestive System Development and Function, Drug Metabolism, Small Molecule Biochemistry	GFI1,U2AF1L4	2	1
10	Cellular Development, Reproductive System Development and Function	GSG1,PAPOLB	2	1
11	Embryonic Development, Tissue Morphology, Cell-To-Cell Signaling and Interaction	DOCK8,SMAD2	2	1
12	Cancer	LIMD2,MIRN151	2	1
13	Carbohydrate Metabolism, Digestive System Development and Function, Hair and Skin Development and Function	IL17RE,TRAF6	2	1
14	Skeletal and Muscular Disorders, Cell-To-Cell Signaling and Interaction, Cellular Movement	heparin,HSPG2 (includes EG:3339),VWA1	2	1
15	Cancer, Gastrointestinal Disease, Cell Cycle	CDC20,CDC27,SPATC1,TUBG1	2	1
16	Cardiac Arrythmia, Cardiovascular Disease, Molecular Transport	ADRB3,KCNA5,KCNE1,KCNH2,KCNQ1,KCNQ4,MIR133A	2	1
