# Supplementary material for: Structural and Regulatory Characterization of the Placental Epigenome at Its Maternal Interface
Source: PLoS One. 2011 Feb 23;6(2):e14723. doi: 10.1371/journal.pone.0014723 (PMC3044138; doi:10.1371/journal.pone.0014723)
Supplement: Table S2 — IPA biological pathway analysis of genes hypomethylated in MBC versus CVS. (0.36 MB PDF) [file pone.0014723.s002.pdf]

¬© 2000-2009 Ingenuity Systems, Inc. All rights reserved.

Category	Biological Function	Function Annotation	P-Value	Molecules	# Molecules
Antigen Presentation	immune response	immune response	5.67E-11	ADA, CD22, CD48, CD93, CD79B, CEBPE, CST7, DOK3, ICAM3, IL10, IL27, IL1R2, ITGAM, LAT, LSP1, NCF1, NCF4, NLRP3, OSM, PGLYRP1, PTPRCAP, S1PR4, SELPLG, SPN, STAT5A, TLR9, VAV1	27
Antigen Presentation	immune response	immune response of organism	8.25E-06	CD48, IL10, LSP1, NCF1, NLRP3, OSM, PTPRCAP, SELPLG, SPN, TLR9, VAV1	11
Antigen Presentation	inflammatory response	inflammatory response	9.14E-10	ADA, AIF1, CEBPE, GPX2, IL10, ITGAM, KLRG1, LAT, LSP1, LYZ, NCF1, NLRP3, NOX4, OSM, PTGDR, PYCARD, SELPLG, SPI1, STAT5A, VAV1	20
Antigen Presentation	inflammatory response	inflammatory response of organism	3.09E-04	ADA, IL10, OSM, SELPLG, STAT5A	5
Antigen Presentation	and	antibacterial response of organism	1.27E-06	CEBPE, IL10, LYZ, NCF1, PGLYRP1, SPN, TLR9	7
Antigen Presentation	phagocytosis	phagocytosis	1.35E-06	CD93, CEBPE, IL10, ITGAM, LAT, PGLYRP1, SPI1, TLR9, VAV1	9
Antigen Presentation	phagocytosis	phagocytosis of macrophages	5.46E-03	PGLYRP1, SPI1	2
Antigen Presentation	adhesion	adhesion of antigen presenting cells	7.96E-05	CD48, ICAM3, ITGAM, SPI1	4
Antigen Presentation	adhesion	adhesion of dendritic cells	1.93E-03	ICAM3, ITGAM	2
Antigen Presentation	inflammation	inflammation	1.10E-04	ADA, IL10, IL27, IL1R2, NLRP3, NR1I2, OSM, PRKAR1B, PYCARD, TLR9	10
Antigen Presentation	antibody response	antibody response	1.87E-04	BST2, DOK3, IL10, TLR9, VAV1	5
Antigen Presentation	proliferation	proliferation of macrophages	6.46E-04	IL10, SPI1, STAT5A	3
Antigen Presentation	activation	activation of plasmacytoid dendritic cells	8.15E-04	IL10, TLR9	2
Antigen Presentation	activation	activation of peritoneal macrophages	4.76E-03	CEBPE, IL10	2
Antigen Presentation	quantity	quantity of macrophages	1.29E-03	ADA, LSP1, LTC4S, SELPLG, SPI1	5
Antigen Presentation	priming	priming of antigen presenting cells	1.51E-03	IL10, TLR9	2
Antigen Presentation	conversion	conversion of dendritic cells	7.48E-03	IL10	1
Antigen Presentation	cross-priming	cross-priming of antigen presenting cells	7.48E-03	TLR9	1
Cell-mediated Immune Response	immune response	immune response	5.67E-11	ADA, CD22, CD48, CD93, CD79B, CEBPE, CST7, DOK3, ICAM3, IL10, IL27, IL1R2, ITGAM, LAT, LSP1, NCF1, NCF4, NLRP3, OSM, PGLYRP1, PTPRCAP, S1PR4, SELPLG, SPN, STAT5A, TLR9, VAV1	27
Cell-mediated Immune Response	immune response	immune response of organism	8.25E-06	CD48, IL10, LSP1, NCF1, NLRP3, OSM, PTPRCAP, SELPLG, SPN, TLR9, VAV1	11
Cell-mediated Immune Response	inflammatory response	inflammatory response	9.14E-10	ADA, AIF1, CEBPE, GPX2, IL10, ITGAM, KLRG1, LAT, LSP1, LYZ, NCF1, NLRP3, NOX4, OSM, PTGDR, PYCARD, SELPLG, SPI1, STAT5A, VAV1	20
Cell-mediated Immune Response	inflammatory response	inflammatory response of organism	3.09E-04	ADA, IL10, OSM, SELPLG, STAT5A	5
Cell-mediated Immune Response	antibacterial response	antibacterial response of organism	1.27E-06	CEBPE, IL10, LYZ, NCF1, PGLYRP1, SPN, TLR9	7
Cell-mediated Immune Response	phagocytosis	phagocytosis	1.35E-06	CD93, CEBPE, IL10, ITGAM, LAT, PGLYRP1, SPI1, TLR9, VAV1	9
Cell-mediated Immune Response	development	development of T lymphocytes	8.74E-06	ADA, CD6, IL10, IL27, LAT, OSM, SPI1, STAT5A, VAV1	9
Cell-mediated Immune Response	development	development of helper T lymphocytes	4.89E-03	IL10, IL27, STAT5A	3
Cell-mediated Immune Response	development	development of Th2 cells	1.37E-02	IL10, STAT5A	2
Cell-mediated Immune Response	activation	activation of T lymphocytes	1.10E-05	ADA, CD48, ICAM3, IL10, IL27, LAT, SPN, STAT5A, TLR9, VAV1	10
Cell-mediated Immune Response	activation	activation of helper T lymphocytes	7.34E-04	IL10, IL27, TLR9	3
Cell-mediated Immune Response	activation	activation of Th1 cells	2.40E-03	IL10, IL27	2
Cell-mediated Immune Response	proliferation	proliferation of T lymphocytes	2.38E-05	CD6, CD37, CD48, DGKA, DOK2, IL10, IL27, ITGAM, PTPRCAP, SPN, STAT5A, TRAF1, VAV1	13
Cell-mediated Immune Response	inflammation	inflammation	1.10E-04	ADA, IL10, IL27, IL1R2, NLRP3, NR1I2, OSM, PRKAR1B, PYCARD, TLR9	10
Cell-mediated Immune Response	cytotoxic reaction	cytotoxic reaction of leukocytes	1.73E-04	CD48, ITGAM, VAV1	3
Cell-mediated Immune Response	cytotoxic reaction	cytotoxic reaction of natural killer cells	1.51E-03	CD48, VAV1	2
Cell-mediated Immune Response	cytotoxic reaction	cytotoxic reaction of eosinophils	7.48E-03	ITGAM	1
Cell-mediated Immune Response	antibody response	antibody response	1.87E-04	BST2, DOK3, IL10, TLR9, VAV1	5
Cell-mediated Immune Response	cell movement	cell movement of Th1 cells	2.12E-04	FUT7, IL10, SELPLG	3
Cell-mediated Immune Response	cell movement	cell movement of Th2 cells	3.48E-03	IL10, SELPLG	2
Cell-mediated Immune Response	function	function of T lymphocytes	2.91E-04	IL10, LAT, STAT5A, VAV1	4
Cell-mediated Immune Response	function	function of cytotoxic T cells	7.48E-03	IL10	1
Cell-mediated Immune Response	generation	generation of Tr1 cells	3.29E-04	IL10, IL27	2
Cell-mediated Immune Response	generation	generation of naive T lymphocytes	8.15E-04	IL10, IL27	2
Cell-mediated Immune Response	adhesion	adhesion of Th1 cells	8.15E-04	FUT7, SELPLG	2
Cell-mediated Immune Response	adhesion	adhesion of T lymphocytes	2.76E-03	CD48, FUT7, SELPLG, SPN	4
Cell-mediated Immune Response	quantity	quantity of T lymphocytes	9.18E-04	DNMT1, IL10, ITGAM, LAT, OSM, SELPLG, STAT5A, VAV1	8
Cell-mediated Immune Response	binding	binding of helper T lymphocytes	1.51E-03	FUT7, SELPLG	2
Cell-mediated Immune Response	binding	binding of T lymphocytes	6.53E-03	CD6, FUT7, SELPLG	3
Cell-mediated Immune Response	differentiation	differentiation of regulatory T lymphocytes	1.93E-03	IL10, IL27	2
Cell-mediated Immune Response	differentiation	differentiation of natural killer cells	7.00E-03	IL10, STAT5A	2
Cell-mediated Immune Response	differentiation	differentiation of Tr1 cells	7.48E-03	IL27	1
Cell-mediated Immune Response	polarization	polarization of T lymphocytes	3.29E-03	IL27, SPN, VAV1	3
Cell-mediated Immune Response	growth	growth of thymocytes	6.21E-03	IL10, SPI1	2
Cell-mediated Immune Response	growth	growth of T lymphocytes	7.17E-03	IL10, IL27, SPI1, STAT5A	4
Cell-mediated Immune Response	aggregation	aggregation of T lymphocytes	7.00E-03	ICAM3, SPN	2
Cell-mediated Immune Response	antibody-dependent cell-mediated cytotoxic reaction	antibody-dependent cell-mediated cytotoxic reaction of natural killer T lymphocytes	7.48E-03	VAV1	1
Cell-mediated Immune Response	cell division	entry into cell division of T lymphocytes	7.48E-03	CD37	1
Cell-mediated Immune Response	frequency	frequency of cytotoxic T cells	7.48E-03	IL10	1
Cell-mediated Immune Response	induction	induction of Tr1 cells	7.48E-03	IL10	1
Cell-mediated Immune Response	outgrowth	outgrowth of cytotoxic T cells	7.48E-03	IL10	1
Cell-mediated Immune Response	repopulation	repopulation of thymocytes	7.48E-03	DOK2	1
Cell-mediated Immune Response	homeostasis	homeostasis of T lymphocytes	7.84E-03	IL10, STAT5A	2
Cell-mediated Immune Response	infiltration	infiltration of T lymphocytes	8.46E-03	IL10, IL18BP, SELPLG	3
Cell-mediated Immune Response	TH1 immune response	TH1 immune response of memory T lymphocytes	1.49E-02	IL10	1
Humoral Immune Response	immune response	immune response	5.67E-11	ADA, CD22, CD48, CD93, CD79B, CEBPE, CST7, DOK3, ICAM3, IL10, IL27, IL1R2, ITGAM, LAT, LSP1, NCF1, NCF4, NLRP3, OSM, PGLYRP1, PTPRCAP, S1PR4, SELPLG, SPN, STAT5A, TLR9, VAV1	27

				¬© 2000-2009 Ingenuity Systems, Inc. All rights reserved.	
**Category**	**Biological Function**	**Function Annotation**	**P-Value**	**Molecules**	**# Molecules**
Humoral Immune Response	immune response	immune response of organism	8.25E-06	CD48, IL10, LSP1, NCF1, NLRP3, OSM, PTPRCAP, SELPLG, SPN, TLR9, VAV1	11
Humoral Immune Response	inflammatory response	inflammatory response	9.14E-10	ADA, AIF1, CEBPE, GPX2, IL10, ITGAM, KLRG1, LAT, LSP1, LYZ, NCF1, NLRP3, NOX4, OSM, PTGDR, PYCARD, SELPLG, SPI1, STAT5A, VAV1	20
Humoral Immune Response	inflammatory response	inflammatory response of organism	3.09E-04	ADA, IL10, OSM, SELPLG, STAT5A	5
Humoral Immune Response	antibacterial response	antibacterial response of organism	1.27E-06	CEBPE, IL10, LYZ, NCF1, PGLYRP1, SPN, TLR9	7
Humoral Immune Response	phagocytosis	phagocytosis	1.35E-06	CD93, CEBPE, IL10, ITGAM, LAT, PGLYRP1, SPI1, TLR9, VAV1	9
Humoral Immune Response	proliferation	proliferation of B lymphocytes	6.28E-05	ADA, CD22, IL10, PTPRCAP, SPI1, STAT5A, TLR9, VAV1	8
Humoral Immune Response	proliferation	proliferation of pre-B lymphocytes	1.37E-02	IL10, SPI1	2
Humoral Immune Response	inflammation	inflammation	1.10E-04	ADA, IL10, IL27, IL1R2, NLRP3, NR1I2, OSM, PRKAR1B, PYCARD, TLR9	10
Humoral Immune Response	differentiation	differentiation of B lymphocytes	1.41E-04	CD79B, IL10, LAT, STAT5A, TLR9, TNFSF8	6
Humoral Immune Response	differentiation	differentiation of pre-B lymphocytes	1.27E-02	CD79B, LAT	2
Humoral Immune Response	antibody response	antibody response	1.87E-04	BST2, DOK3, IL10, TLR9, VAV1	5
Humoral Immune Response	antibody response	antibody response of mice	7.34E-04	DOK3, IL10, VAV1	3
Humoral Immune Response	activation	activation of B lymphocytes	2.27E-04	ADA, CD22, DOK3, IL10, VAV1	5
Humoral Immune Response	growth	growth of B lymphocytes	3.53E-03	CD22, IL10, STAT5A	3
Humoral Immune Response	quantity	quantity of B lymphocytes	7.17E-03	CD22, IL10, SPN, STAT5A, VAV1	5
Humoral Immune Response	cell rolling	cell rolling of pre-B lymphocytes	7.48E-03	SELPLG	1
Humoral Immune Response	mobility	mobility of B lymphocytes	7.48E-03	IL10	1
Humoral Immune Response	morphology	morphology of germinal center	7.48E-03	ADA	1
Humoral Immune Response	progression	progression of pro-B lymphocytes	7.48E-03	CD79B	1
Inflammatory Response	immune response	immune response	5.67E-11	ADA, CD22, CD48, CD93, CD79B, CEBPE, CST7, DOK3, ICAM3, IL10, IL27, IL1R2, ITGAM, LAT, LSP1, NCF1, NCF4, NLRP3, OSM, PGLYRP1, PTPRCAP, S1PR4, SELPLG, SPN, STAT5A, TLR9, VAV1	27
Inflammatory Response	immune response	immune response of organism	8.25E-06	CD48, IL10, LSP1, NCF1, NLRP3, OSM, PTPRCAP, SELPLG, SPN, TLR9, VAV1	11
Inflammatory Response	inflammatory response	inflammatory response	9.14E-10	ADA, AIF1, CEBPE, GPX2, IL10, ITGAM, KLRG1, LAT, LSP1, LYZ, NCF1, NLRP3, NOX4, OSM, PTGDR, PYCARD, SELPLG, SPI1, STAT5A, VAV1	20
Inflammatory Response	inflammatory response	inflammatory response of organism	3.09E-04	ADA, IL10, OSM, SELPLG, STAT5A	5
Inflammatory Response	antibacterial response	antibacterial response of organism	1.27E-06	CEBPE, IL10, LYZ, NCF1, PGLYRP1, SPN, TLR9	7
Inflammatory Response	phagocytosis	phagocytosis	1.35E-06	CD93, CEBPE, IL10, ITGAM, LAT, PGLYRP1, SPI1, TLR9, VAV1	9
Inflammatory Response	phagocytosis	phagocytosis of eukaryotic cells	5.98E-06	CD93, ITGAM, LAT, PGLYRP1, SPI1, TLR9, VAV1	7
Inflammatory Response	phagocytosis	phagocytosis of normal cells	1.87E-04	CD93, ITGAM, PGLYRP1, SPI1, TLR9	5
Inflammatory Response	phagocytosis	phagocytosis of leukocytes	2.42E-04	CD93, ITGAM, PGLYRP1, SPI1	4
Inflammatory Response	phagocytosis	phagocytosis of fibroblast cell lines	3.29E-04	LAT, VAV1	2
Inflammatory Response	phagocytosis	phagocytosis of kidney cell lines	5.46E-04	LAT, VAV1	2
Inflammatory Response	phagocytosis	phagocytosis of cell lines	2.24E-03	LAT, SPI1, VAV1	3
Inflammatory Response	binding	binding of phagocytes	6.00E-05	FUT7, IL10, ITGAM, LSP1, SELPLG	5
Inflammatory Response	binding	binding of neutrophils	6.97E-05	FUT7, ITGAM, LSP1, SELPLG	4
Inflammatory Response	binding	binding of blood platelets	4.31E-03	ICAM4 (includes EG:3386), NFE2, SELPLG	3
Inflammatory Response	inflammation	inflammation	1.10E-04	ADA, IL10, IL27, IL1R2, NLRP3, NR1I2, OSM, PRKAR1B, PYCARD, TLR9	10
Inflammatory Response	inflammation	inflammation of intestine	4.22E-04	IL10, NR1I2, TLR9	3
Inflammatory Response	inflammation	inflammation of mice	2.06E-03	IL10, IL1R2, NLRP3, PYCARD	4
Inflammatory Response	inflammation	inflammation of hindlimb	7.48E-03	PRKAR1B	1
Inflammatory Response	inflammation	inflammation of small intestine	7.48E-03	NR1I2	1
Inflammatory Response	antibody response	antibody response	1.87E-04	BST2, DOK3, IL10, TLR9, VAV1	5
Inflammatory Response	quantity	quantity of phagocytes	1.95E-04	ADA, IL10, IL18BP, LSP1, LTC4S, SELPLG, SPI1	7
Inflammatory Response	quantity	quantity of macrophages	1.29E-03	ADA, LSP1, LTC4S, SELPLG, SPI1	5
Inflammatory Response	quantity	quantity of neutrophils	2.89E-03	ADA, IL10, IL18BP, SELPLG	4
Inflammatory Response	recruitment	recruitment of neutrophils	2.91E-04	IL10, ITGAM, LSP1, LYZ, PYCARD	5
Inflammatory Response	cell movement	cell movement of neutrophils	4.43E-04	FUT7, IL10, ITGAM, LSP1, SELPLG, SPI1, SPN	7
Inflammatory Response	proliferation	proliferation of macrophages	6.46E-04	IL10, SPI1, STAT5A	3
Inflammatory Response	activation	activation of plasmacytoid dendritic cells	8.15E-04	IL10, TLR9	2
Inflammatory Response	activation	activation of phagocytes	2.91E-03	CEBPE, IL10, ITGAM, TLR9, VAV1	5
Inflammatory Response	activation	activation of peritoneal macrophages	4.76E-03	CEBPE, IL10	2
Inflammatory Response	activation	activation of neutrophils	1.07E-02	IL10, ITGAM, VAV1	3
Inflammatory Response	adhesion	adhesion of phagocytes	1.49E-03	ITGAM, SELPLG, SPI1, VAV1	4
Inflammatory Response	adhesion	adhesion of dendritic cells	1.93E-03	ICAM3, ITGAM	2
Inflammatory Response	adhesion	adhesion of neutrophils	6.53E-03	ITGAM, SELPLG, VAV1	3
Inflammatory Response	aggregation	aggregation of neutrophils	1.51E-03	ITGAM, SELPLG	2
Inflammatory Response	response	response of macrophages	1.51E-03	IL10, ITGAM	2
Inflammatory Response	migration	migration of Langerhans cells	5.46E-03	IL10, PTGDR	2
Inflammatory Response	cell rolling	cell rolling of blood platelets	7.48E-03	SELPLG	1
Cellular Growth and Proliferation	proliferation	proliferation of blood cells	2.10E-07	ADA, CD6, CD22, CD37, CD48, DGKA, DOK2, IL10, IL27, ITGAM, NFE2, PTPRCAP, PTPRO, SPI1, SPN, STAT5A, TLR9, TRAF1, VAV1	19
Cellular Growth and Proliferation	proliferation	proliferation of lymphocytes	8.01E-07	ADA, CD6, CD22, CD37, CD48, DGKA, DOK2, IL10, IL27, ITGAM, PTPRCAP, SPI1, SPN, STAT5A, TLR9, TRAF1, VAV1	17
Cellular Growth and Proliferation	proliferation	proliferation of T lymphocytes	2.38E-05	CD6, CD37, CD48, DGKA, DOK2, IL10, IL27, ITGAM, PTPRCAP, SPN, STAT5A, TRAF1, VAV1	13
Cellular Growth and Proliferation	proliferation	proliferation of normal cells	2.76E-05	ADA, AIF1, CD6, CD22, CD37, CD48, DGKA, DOK2, IL10, IL27, ITGAM, NCF1, NFE2, NOX4, OSM, OXT, PTPRCAP, PTPRO, SPI1, SPN, STAT5A, TLR9, TRAF1, VAV1	24
Cellular Growth and Proliferation	proliferation	proliferation of B lymphocytes	6.28E-05	ADA, CD22, IL10, PTPRCAP, SPI1, STAT5A, TLR9, VAV1	8
Cellular Growth and Proliferation	proliferation	proliferation of cells	2.68E-04	ADA, AIF1, ALOX15B, BST2, CD6, CD22, CD37, CD48, CEBPE, DGKA, DNMT1, DOK2, HOXA7, IL10, IL27, ITGAM, LGALS7, NCF1, NFE2, NOX4, OSM, OXT, PRKAR1B, PTPRCAP, PTPRO, SPI1, SPN, STAT5A, TLR9, TNFSF8, TRAF1, VAV1	32

				¬© 2000-2009 Ingenuity Systems, Inc. All rights reserved.	
**Category**	**Biological Function**	**Function Annotation**	**P-Value**	**Molecules**	**# Molecules**
Cellular Growth and Proliferation	proliferation	proliferation of hematopoietic cells	3.09E-04	CD22, NFE2, PTPRO, SPI1, STAT5A	5
Cellular Growth and Proliferation	proliferation	proliferation of eukaryotic cells	3.50E-04	ADA, AIF1, CD6, CD22, CD37, CD48, CEBPE, DGKA, DOK2, HOXA7, IL10, IL27, ITGAM, NCF1, NFE2, NOX4, OSM, OXT, PTPRCAP, PTPRO, SPI1, SPN, STAT5A, TLR9, TNFSF8, TRAF1, VAV1	27
Cellular Growth and Proliferation	proliferation	proliferation of macrophages	6.46E-04	IL10, SPI1, STAT5A	3
Cellular Growth and Proliferation	proliferation	proliferation of hematopoietic progenitor cells	1.49E-03	CD22, NFE2, PTPRO, STAT5A	4
Cellular Growth and Proliferation	proliferation	proliferation of PASMC cells	1.93E-03	IL10, NOX4	2
Cellular Growth and Proliferation	proliferation	proliferation of smooth muscle cells	4.77E-03	AIF1, IL10, NCF1, NOX4, OXT	5
Cellular Growth and Proliferation	proliferation	proliferation of myoepithelial cells	7.48E-03	OXT	1
Cellular Growth and Proliferation	proliferation	proliferation of synovial cells	7.84E-03	AIF1, OSM	2
Cellular Growth and Proliferation	proliferation	proliferation of pre-B lymphocytes	1.37E-02	IL10, SPI1	2
Cellular Growth and Proliferation	proliferation	proliferation of bone marrow cells	1.43E-02	NFE2, SPI1, STAT5A	3
Cellular Growth and Proliferation	generation	generation of Tr1 cells	3.29E-04	IL10, IL27	2
Cellular Growth and Proliferation	generation	generation of naive T lymphocytes	8.15E-04	IL10, IL27	2
Cellular Growth and Proliferation	generation	generation of dendritic cells	6.21E-03	IL10, SPI1	2
Cellular Growth and Proliferation	growth	growth of cell lines	7.55E-04	AIF1, ALOX15B, CEBPE, HOXA7, HPN, IL10, LGALS7, NOX4, OSM, OXT, POR, PTPRO, RASSF1, S100A4, SPI1, STAT5A, TNK1	17
Cellular Growth and Proliferation	growth	growth of eukaryotic cells	7.60E-04	AIF1, ALOX15B, CD22, CEBPE, HOXA7, HPN, IL10, IL27, LGALS7, NOX4, OSM, OXT, POR, PTPRO, RASSF1, S100A4, SPI1, STAT5A, TNK1, VAV1	20
Cellular Growth and Proliferation	growth	growth of tumor cell lines	9.46E-04	CEBPE, HOXA7, HPN, IL10, LGALS7, OSM, OXT, POR, PTPRO, RASSF1, S100A4, SPI1, STAT5A, TNK1	14
Cellular Growth and Proliferation	growth	growth of cells	2.71E-03	AIF1, ALOX15B, CD22, CEBPE, DGKA, HOXA7, HPN, ICAM3, IL10, IL27, LGALS7, MGAT1, NOX4, OSM, OXT, PKP1, POR, PTPRO, RASSF1, S100A4, SPI1, STAT5A, TNK1, VAV1	24
Cellular Growth and Proliferation	growth	growth of lymphocytes	3.42E-03	CD22, IL10, IL27, SPI1, STAT5A	5
Cellular Growth and Proliferation	growth	growth of B lymphocytes	3.53E-03	CD22, IL10, STAT5A	3
Cellular Growth and Proliferation	growth	growth of thymocytes	6.21E-03	IL10, SPI1	2
Cellular Growth and Proliferation	growth	growth of T lymphocytes	7.17E-03	IL10, IL27, SPI1, STAT5A	4
Cellular Growth and Proliferation	growth	arrest in growth of liver cancer cells	7.48E-03	HPN	1
Cellular Growth and Proliferation	growth	growth of bone marrow cells	7.65E-03	IL10, SPI1, VAV1	3
Cellular Growth and Proliferation	inhibition	inhibition of T lymphocytes	1.04E-03	IL10, IL27, TLR9	3
Cellular Growth and Proliferation	inhibition	inhibition of helper T lymphocytes	1.51E-03	IL10, IL27	2
Cellular Growth and Proliferation	expansion	expansion of lymphocytes	7.93E-03	IL10, IL27, SPI1, STAT5A	4
Cellular Growth and Proliferation	colony formation	colony formation of carcinoma cell lines	1.06E-02	RASSF1, RFFL	2
Cellular Growth and Proliferation	colony formation	colony formation of cells	1.26E-02	DOK2, HPN, IL10, LGALS7, NFE2, RASSF1, RFFL, SPI1	8
Cellular Growth and Proliferation	formation	formation of blood cells	1.27E-02	IL10, NFE2, SPI1	3
Cancer	infection	infection of lymphoma cell lines	4.07E-07	FUT7, ITGAM, SELPLG	3
Cancer	lymphoid cancer	lymphoid cancer	9.42E-06	ADA, CD22, CD52, IL10, LGALS7, POR, RASSF1, SPI1, STAT5A, TLR9, TNFSF8	11
Cancer	cell death	cell death of tumor cell lines	4.02E-05	CD48, CD79B, GPX2, HOXA7, HPN, IL10, ITGAM, LGALS7, LSP1, NLRP3, NR1I2, NUAK1, PADI4, PTPRO, PYCARD, RASSF1, S100A4, SOD3, SPI1, SPN, TRAF1, VAV1	22
Cancer	cell death	cell death of lymphoma cell lines	7.36E-03	CD48, CD79B, HOXA7, IL10, LSP1	5
Cancer	lymphoma	lymphoma	2.48E-04	ADA, CD22, CD52, IL10, LGALS7, POR, RASSF1, SPI1, TLR9	9
Cancer	cell rolling	cell rolling of leukemia cell lines	5.46E-04	FUT7, SELPLG	2
Cancer	necrosis	necrosis of lymphoma cell lines	5.46E-04	HOXA7, IL10	2
Cancer	necrosis	necrosis of tumor tissue	7.48E-03	NUAK1	1
Cancer	binding	binding of leukemia cell lines	7.52E-04	FUT7, ITGAM, SELPLG, SPN	4
Cancer	binding	binding of tumor cell lines	7.36E-03	FUT7, ITGAM, OSM, SELPLG, SPN	5
Cancer	growth	growth of tumor cell lines	9.46E-04	CEBPE, HOXA7, HPN, IL10, LGALS7, OSM, OXT, POR, PTPRO, RASSF1, S100A4, SPI1, STAT5A, TNK1	14
Cancer	growth	arrest in growth of liver cancer cells	7.48E-03	HPN	1
Cancer	growth	growth of prostatic carcinoma	7.48E-03	DAB2IP	1
Cancer	adhesion	adhesion of tumor cell lines	1.00E-03	ALOX15B, FUT7, ITGAM, LAT, RASSF1, SELPLG, SPN	7
Cancer	adhesion	adhesion of leukemia cell lines	7.65E-03	ITGAM, LAT, SELPLG	3
Cancer	apoptosis	apoptosis of tumor cell lines	1.10E-03	CD48, CD79B, GPX2, HPN, ITGAM, LGALS7, LSP1, NR1I2, PADI4, PTPRO, PYCARD, RASSF1, S100A4, SOD3, SPN, TRAF1, VAV1	17
Cancer	morphology	morphology of cancer cells	1.14E-03	HPN, LSP1	2
Cancer	morphology	morphology of liver cancer cells	7.48E-03	HPN	1
Cancer	developmental process	developmental process of genital tumor	1.93E-03	DAB2IP, DNMT1	2
Cancer	developmental process	developmental process of tumor cell lines	3.89E-03	CEBPE, HOXA7, HPN, IL10, ITGAM, LGALS7, OSM, OXT, POR, PTPRO, RASSF1, S100A4, SPI1, STAT5A, TNK1	15
Cancer	developmental process	developmental process of adenoma	4.10E-03	DNMT1, RASSF1	2
Cancer	developmental process	developmental process of lymphoma cell lines	1.32E-02	HOXA7, IL10, ITGAM	3
Cancer	leukemia	leukemia	2.39E-03	ADA, CD22, CD52, DOK2, LMO2, POR, SPI1, TLR9	8
Cancer	leukemia	leukemia of mammalia	9.76E-03	DOK2, LMO2, SPI1	3
Cancer	hyperproliferation	hyperproliferation of hematopoietic progenitor cells	2.40E-03	DOK2, IL10	2
Cancer	hyperproliferation	hyperproliferation of blood cells	8.88E-03	DOK2, DOK3, IL10	3
Cancer	cell movement	cell movement of lung cancer cell lines	2.92E-03	FUT7, S100A4	2
Cancer	hairy-cell leukemia	hairy-cell leukemia	2.92E-03	ADA, CD22	2
Cancer	survival	survival of tumor cell lines	3.31E-03	DGKA, IL10, NUAK1, PPP1R16B, PTPN7, PTPRCAP, PTPRO, PYCARD, TNFSF8	9
Cancer	survival	survival of cervical cancer cell lines	8.96E-03	DGKA, PPP1R16B, PTPN7, PTPRCAP, PTPRO	5
Cancer	tumor	tumor	4.08E-03	ADA, CD22, CD48, CD52, COL7A1, CST7, DAB2IP, DNMT1, DUSP2, GPX2, HPN, IL10, IL18BP, KCNE1, LGALS7, LIMD2, LSP1, LYZ, NR1I2, NUAK1, POR, RASSF1, S100A4, SPI1, STAT5A, TLR9, VAV1	27
Cancer	chronic lymphocytic leukemia	chronic lymphocytic leukemia	4.09E-03	ADA, CD22, CD52, TLR9	4
Cancer	Hodgkin's disease	Hodgkin's disease	5.19E-03	POR, STAT5A, TNFSF8	3
Cancer	Hodgkin's disease	Hodgkin's disease of humans	7.48E-03	STAT5A	1

¬© 2000-2009 Ingenuity Systems, Inc. All rights reserved.

Category	Biological Function	Function Annotation	P-Value	Molecules	# Molecules
Cancer	myeloproliferative syndrome	myeloproliferative syndrome of mice	6.21E-03	DOK2, SPI1	2
Cancer	cell cycle progression	cell cycle progression of tumor cell lines	6.29E-03	PADI4, PTPRO, RASSF1, S100A4, VAV1	5
Cancer	cell cycle progression	arrest in cell cycle progression of pancreatic cancer cell lines	7.48E-03	S100A4	1
Cancer	tumorigenesis	tumorigenesis of mammalia	6.98E-03	DNMT1, DOK2, LMO2, RASSF1, SPI1, STAT5A	6
Cancer	tumorigenesis	tumorigenesis of unspecified cell lines	7.48E-03	RASSF1	1
Cancer	tumorigenesis	tumorigenesis	8.51E-03	ADA, BST2, CD22, CD48, CD52, COL7A1, CST7, DAB2IP, DGKA, DNMT1, DOK2, DUSP2, GPX2, HPN, IL10, IL18BP, KCNE1, LGALS7, LIMD2, LMO2, LSP1, LTC4S, LYZ, NR1I2, NUAK1, OSM, POR, RASSF1, S100A4, SPI1, STAT5A, TLR9, TNFSF8, VAV1	34
Cancer	tumorigenesis	tumorigenesis of breast cancer cell lines	1.49E-02	DUSP2, S100A4	2
Cancer	disease	disease of leukemia cell lines	7.00E-03	NLRP3, SELPLG	2
Cancer	clearance	clearance of tumor	7.48E-03	VAV1	1
Cancer	colony survival	colony survival of adenocarcinoma cells	7.48E-03	IL10	1
Cancer	colony survival	colony survival of glioblastoma multiforme	7.48E-03	IL10	1
Cancer	dedifferentiation	dedifferentiation of erythroleukemia cells	7.48E-03	SPI1	1
Cancer	development	development of pulmonary adenoma	7.48E-03	RASSF1	1
Cancer	differentiation	differentiation of teratoma	7.48E-03	DNMT1	1
Cancer	edema	edema of leukemia cell lines	7.48E-03	NLRP3	1
Cancer	invasion	invasion of proerythroblasts	7.48E-03	SPI1	1
Cancer	invasion	invasion of synovial cells	7.48E-03	OSM	1
Cancer	multiplicity	multiplicity of skin tumor	7.48E-03	RASSF1	1
Cancer	oxidative stress response	oxidative stress response of leukemia cell lines	7.48E-03	VAV1	1
Cancer	progression	progression of cervical cancer cell lines	7.48E-03	RASSF1	1
Cancer	volume	volume of metastatic tumor	7.48E-03	IL18BP	1
Cancer	lymphocytic leukemia	lymphocytic leukemia	7.55E-03	ADA, CD22, CD52, POR, TLR9	5
Cancer	cancer	cancer of mammalia	9.31E-03	DOK2, LMO2, SPI1, STAT5A	4
Cancer	cancer	cancer of humans	1.06E-02	LMO2, STAT5A	2
Cancer	papillary thyroid carcinoma	papillary thyroid carcinoma	9.76E-03	CD48, CST7, LIMD2	3
Cancer	colony formation	colony formation of carcinoma cell lines	1.06E-02	RASSF1, RFFL	2
Cancer	G1 phase	arrest in G1 phase of breast cancer cell lines	1.27E-02	CAMKK1, RASSF1	2
Cancer	mycosis fungoides	mycosis fungoides	1.27E-02	CD52, TLR9	2
Cancer	primary tumor	primary tumor	1.29E-02	ADA, CD22, CD48, CD52, COL7A1, CST7, DAB2IP, DNMT1, DUSP2, GPX2, HPN, IL10, KCNE1, LGALS7, LIMD2, LSP1, LYZ, NR1I2, POR, RASSF1, S100A4, SPI1, STAT5A, TLR9	24
Immunological Disease	infection	infection of lymphoma cell lines	4.07E-07	FUT7, ITGAM, SELPLG	3
Immunological Disease	hypersensitive reaction	hypersensitive reaction	5.66E-07	DUSP2, FUT7, IL10, IL18BP, LAT, LTC4S, NCF1, OSM, SPI1, TNFSF8, VAV1	11
Immunological Disease	hypersensitive reaction	hypersensitive reaction of mice	3.47E-04	IL10, IL18BP, LAT, NCF1, VAV1	5
Immunological Disease	immunological disorder	immunological disorder	3.22E-05	ADA, AFF3, AIF1, ATP10A, CD6, CD22, CD52, CEBPE, CLEC4A, COL7A1, CRYBB1, DOK2, DUSP2, FUT7, ICAM3, IL10, IL27, IL18BP, IL1R2, ITGAM, KLRG1, LAT, LMO2, LTC4S, LYZ, ME1, NCF1, NCF4, OSM, PADI4, PGLYRP1, PTPRO, SH3TC1, SPI1, STAT5A, TLR9, TNFSF8, TRAF1, VAV1	39
Immunological Disease	immunological disorder	immunological disorder of mice	4.42E-05	CD22, DOK2, FUT7, IL10, IL27, IL18BP, LAT, NCF1, STAT5A, TLR9, VAV1	11
Immunological Disease	murine acquired immunodeficiency syndrome	murine acquired immunodeficiency syndrome of mice	1.65E-04	CD22, VAV1	2
Immunological Disease	apoptosis	apoptosis of plasmacytoid dendritic cells	3.29E-04	IL10, TLR9	2
Immunological Disease	apoptosis	apoptosis of leukocytes	5.48E-03	ADA, CD22, IL10, ITGAM, SPN, STAT5A, TLR9, TRAF1	8
Immunological Disease	apoptosis	apoptosis of myeloid dendritic cells	7.48E-03	IL10	1
Immunological Disease	apoptosis	apoptosis of peripheral blood monocytes	7.48E-03	IL10	1
Immunological Disease	apoptosis	apoptosis of lymphatic system cells	1.18E-02	ADA, IL10, ITGAM, STAT5A	4
Immunological Disease	apoptosis	apoptosis of lymphocytes	1.41E-02	ADA, CD22, IL10, SPN, STAT5A, TRAF1	6
Immunological Disease	necrosis	necrosis of lymphoma cell lines	5.46E-04	HOXA7, IL10	2
Immunological Disease	atopic dermatitis	atopic dermatitis	6.89E-04	DUSP2, FUT7, OSM, SPI1, TNFSF8	5
Immunological Disease	cell death	cell death of leukocytes	9.14E-04	ADA, CD22, IL10, ITGAM, LAT, PYCARD, SPN, STAT5A, TLR9, TRAF1	10
Immunological Disease	cell death	cell death of lymphoma cell lines	7.36E-03	CD48, CD79B, HOXA7, IL10, LSP1	5
Immunological Disease	cell death	cell death of lymphocytes	7.52E-03	ADA, CD22, IL10, LAT, SPN, STAT5A, TRAF1	7
Immunological Disease	cell death	cell death of T lymphocytes	8.90E-03	ADA, IL10, LAT, SPN, STAT5A, TRAF1	6
Immunological Disease	systemic anaphylaxis	systemic anaphylaxis of mice	1.14E-03	LAT, VAV1	2
Immunological Disease	lymphadenopathy	lymphadenopathy of mice	1.29E-03	DOK2, IL10, VAV1	3
Immunological Disease	autoimmune disease	autoimmune disease	5.32E-03	AFF3, AIF1, ATP10A, CD6, CD22, CD52, CLEC4A, COL7A1, CRYBB1, DUSP2, ICAM3, IL10, IL27, IL18BP, IL1R2, ITGAM, KLRG1, LMO2, LYZ, ME1, NCF1, NCF4, OSM, PADI4, PGLYRP1, PTPRO, SH3TC1, TLR9, TRAF1	29
Immunological Disease	infectious disorder	infectious disorder of antigen presenting cells	6.21E-03	ITGAM, TLR9	2
Immunological Disease	systemic lupus erythematosus	systemic lupus erythematosus	6.46E-03	CD22, IL10, ITGAM, TLR9	4
Immunological Disease	systemic lupus erythematosus	systemic lupus erythematosus of animal	1.49E-02	CD22, TLR9	2
Immunological Disease	adenosine deaminase deficiency	adenosine deaminase deficiency	7.48E-03	ADA	1
Immunological Disease	depletion	depletion of plasmacytoid dendritic cells	7.48E-03	BST2	1
Immunological Disease	leukocyte adhesion deficiency	leukocyte adhesion deficiency of mice	7.48E-03	FUT7	1
Immunological Disease	leukopenia	leukopenia of humans	7.48E-03	IL10	1
Immunological Disease	rheumatoid arthritis	onset of rheumatoid arthritis of mice	7.48E-03	IL10	1
Immunological Disease	specific granule deficiency	specific granule deficiency	7.48E-03	CEBPE	1
Immunological Disease	anaphylaxis	anaphylaxis	7.65E-03	LAT, LTC4S, VAV1	3
Immunological Disease	splenomegaly	splenomegaly of mice	9.31E-03	DOK2, STAT5A, VAV1	3
Immunological Disease	developmental process	developmental process of lymphoma cell lines	1.32E-02	HOXA7, IL10, ITGAM	3

				¬© 2000-2009 Ingenuity Systems, Inc. All rights reserved.	
**Category**	**Biological Function**	**Function Annotation**	**P-Value**	**Molecules**	**# Molecules**
Infectious Disease	infection	infection of lymphoma cell lines	4.07E-07	FUT7, ITGAM, SELPLG	3
Infectious Disease	infection	infection of normal cells	2.24E-03	CD93, ITGAM, TLR9	3
Infectious Disease	infection	infection of lung	4.76E-03	LYZ, TLR9	2
Infectious Disease	acquired immunodeficiency syndrome	acquired immunodeficiency syndrome of mammalia	4.02E-06	CD22, IL10, VAV1	3
Infectious Disease	severe acute respiratory syndrome	severe acute respiratory syndrome	3.18E-05	CEBPE, CTSZ (includes EG:1522), ITGAM, NCF1, NFE2, PADI4, PGLYRP1	7
Infectious Disease	infectious disorder	infectious disorder	1.63E-04	ADA, CD22, CD52, CD93, CEBPE, CTSZ (includes EG:1522), FUT7, GPR77, GUCY1B2, IL10, IL1R2, ITGAM, LAPTM5, LAT, LYZ, MGAT1, NCF1, NFE2, PADI4, PGLYRP1, POR, SELPLG, TLR9, TNK1, VAV1	25
Infectious Disease	infectious disorder	infectious disorder of mice	6.57E-04	CD22, FUT7, IL10, LAT, TLR9, VAV1	6
Infectious Disease	infectious disorder	infectious disorder of antigen presenting cells	6.21E-03	ITGAM, TLR9	2
Infectious Disease	murine acquired immunodeficiency syndrome	murine acquired immunodeficiency syndrome of mice	1.65E-04	CD22, VAV1	2
Infectious Disease	parasitemia	parasitemia of mice	1.93E-03	IL10, TLR9	2
Infectious Disease	endotoxin shock response	endotoxin shock response	2.43E-03	GUCY1B2, IL10, PYCARD	3
Infectious Disease	endotoxin shock response	endotoxin shock response of mice	7.00E-03	IL10, PYCARD	2
Infectious Disease	sepsis	sepsis	3.30E-03	GPR77, GUCY1B2, IL10, IL1R2	4
Infectious Disease	lyme arthritis	lyme arthritis	7.48E-03	IL10	1
Gastrointestinal Disease	injury	injury of liver	7.38E-07	IL10, NCF1, NLRP3, NR1I2, PYCARD, TLR9	6
Gastrointestinal Disease	fibrosis	fibrosis of liver	1.49E-02	IL10, NCF1	2
Hepatic System Disease	injury	injury of liver	7.38E-07	IL10, NCF1, NLRP3, NR1I2, PYCARD, TLR9	6
Hepatic System Disease	growth	arrest in growth of liver cancer cells	7.48E-03	HPN	1
Hepatic System Disease	hepatomegaly	hepatomegaly of rats	7.48E-03	NR1I2	1
Hepatic System Disease	morphology	morphology of liver cancer cells	7.48E-03	HPN	1
Hepatic System Disease	fibrosis	fibrosis of liver	1.49E-02	IL10, NCF1	2
Organismal Injury and Abnormalities	injury	injury of liver	7.38E-07	IL10, NCF1, NLRP3, NR1I2, PYCARD, TLR9	6
Organismal Injury and Abnormalities	injury	injury of organ	1.70E-05	IL10, NCF1, NLRP3, NR1I2, PYCARD, SOD3, TLR9	7
Organismal Injury and Abnormalities	necrosis	necrosis	5.20E-03	ADA, HOXA7, IL10, NUAK1, TRAF1	5
Organismal Injury and Abnormalities	fibrosis	fibrosis of mice	5.46E-03	IL10, OSM	2
Organismal Injury and Abnormalities	fibrosis	fibrosis of liver	1.49E-02	IL10, NCF1	2
Organismal Injury and Abnormalities	tumor tissue	tumor tissue	6.21E-03	DNMT1, NUAK1	2
Organismal Injury and Abnormalities	endotoxin shock response	endotoxin shock response of mice	7.00E-03	IL10, PYCARD	2
Organismal Injury and Abnormalities	edema	edema of leukemia cell lines	7.48E-03	NLRP3	1
Organismal Injury and Abnormalities	organismal abnormalities	organismal abnormalities of lung	8.05E-03	ADA, IL10, SOD3	3
Organismal Injury and Abnormalities	organismal abnormalities	organismal abnormalities of organ	8.33E-03	ADA, IL10, LAT, NCF1, SOD3	5
Hematological System Development and Function	proliferation	proliferation of lymphocytes	8.01E-07	ADA, CD6, CD22, CD37, CD48, DGKA, DOK2, IL10, IL27, ITGAM, PTPRCAP, SPI1, SPN, STAT5A, TLR9, TRAF1, VAV1	17
Hematological System Development and Function	proliferation	proliferation of T lymphocytes	2.38E-05	CD6, CD37, CD48, DGKA, DOK2, IL10, IL27, ITGAM, PTPRCAP, SPN, STAT5A, TRAF1, VAV1	13
Hematological System Development and Function	proliferation	proliferation of B lymphocytes	6.28E-05	ADA, CD22, IL10, PTPRCAP, SPI1, STAT5A, TLR9, VAV1	8
Hematological System Development and Function	proliferation	proliferation of hematopoietic cells	3.09E-04	CD22, NFE2, PTPRO, SPI1, STAT5A	5
Hematological System Development and Function	proliferation	proliferation of macrophages	6.46E-04	IL10, SPI1, STAT5A	3
Hematological System Development and Function	proliferation	proliferation of hematopoietic progenitor cells	1.49E-03	CD22, NFE2, PTPRO, STAT5A	4
Hematological System Development and Function	proliferation	proliferation of pre-B lymphocytes	1.37E-02	IL10, SPI1	2
Hematological System Development and Function	activation	activation of mononuclear leukocytes	2.22E-06	ADA, CD22, CD48, CD93, DOK3, ICAM3, IL10, IL27, LAT, SPN, STAT5A, TLR9, VAV1	13
Hematological System Development and Function	activation	activation of leukocytes	3.46E-06	ADA, CD22, CD48, CD93, CEBPE, DOK3, ICAM3, IL10, IL27, ITGAM, LAT, SPN, STAT5A, TLR9, VAV1	15
Hematological System Development and Function	activation	activation of lymphocytes	7.45E-06	ADA, CD22, CD48, DOK3, ICAM3, IL10, IL27, LAT, SPN, STAT5A, TLR9, VAV1	12
Hematological System Development and Function	activation	activation of T lymphocytes	1.10E-05	ADA, CD48, ICAM3, IL10, IL27, LAT, SPN, STAT5A, TLR9, VAV1	10
Hematological System Development and Function	activation	activation of B lymphocytes	2.27E-04	ADA, CD22, DOK3, IL10, VAV1	5
Hematological System Development and Function	activation	activation of helper T lymphocytes	7.34E-04	IL10, IL27, TLR9	3
Hematological System Development and Function	activation	activation of plasmacytoid dendritic cells	8.15E-04	IL10, TLR9	2
Hematological System Development and Function	activation	activation of Th1 cells	2.40E-03	IL10, IL27	2
Hematological System Development and Function	activation	activation of mast cells	2.43E-03	CD48, IL10, ITGAM	3
Hematological System Development and Function	activation	activation of phagocytes	2.91E-03	CEBPE, IL10, ITGAM, TLR9, VAV1	5
Hematological System Development and Function	activation	activation of peritoneal macrophages	4.76E-03	CEBPE, IL10	2
Hematological System Development and Function	activation	activation of neutrophils	1.07E-02	IL10, ITGAM, VAV1	3
Hematological System Development and Function	quantity	quantity of leukocytes	2.29E-06	ADA, CD22, DNMT1, DOK2, IL10, IL18BP, ITGAM, LAT, LSP1, LTC4S, OSM, SELPLG, SPI1, SPN, STAT5A, VAV1	16
Hematological System Development and Function	quantity	quantity of lymphocytes	3.30E-05	CD22, DNMT1, DOK2, IL10, ITGAM, LAT, LSP1, OSM, SELPLG, SPN, STAT5A, VAV1	12
Hematological System Development and Function	quantity	quantity of phagocytes	1.95E-04	ADA, IL10, IL18BP, LSP1, LTC4S, SELPLG, SPI1	7
Hematological System Development and Function	quantity	quantity of antigen presenting cells	7.34E-04	ADA, IL10, LSP1, LTC4S, SELPLG, SPI1	6
Hematological System Development and Function	quantity	quantity of T lymphocytes	9.18E-04	DNMT1, IL10, ITGAM, LAT, OSM, SELPLG, STAT5A, VAV1	8
Hematological System Development and Function	quantity	quantity of macrophages	1.29E-03	ADA, LSP1, LTC4S, SELPLG, SPI1	5
Hematological System Development and Function	quantity	quantity of neutrophils	2.89E-03	ADA, IL10, IL18BP, SELPLG	4
Hematological System Development and Function	quantity	quantity of B lymphocytes	7.17E-03	CD22, IL10, SPN, STAT5A, VAV1	5
Hematological System Development and Function	quantity	quantity of lymphocyte precursor cells	7.48E-03	DOK2	1
Hematological System Development and Function	differentiation	differentiation of blood cells	4.31E-06	ADA, CD79B, CEBPE, IL10, IL27, ITGAM, LAT, LMO2, NFE2, SPI1, SPN, STAT5A, TLR9, TNFSF8, VAV1	15
Hematological System Development and Function	differentiation	differentiation of lymphocytes	1.26E-05	ADA, CD79B, IL10, IL27, LAT, SPI1, SPN, STAT5A, TLR9, TNFSF8, VAV1	11
Hematological System Development and Function	differentiation	differentiation of leukocytes	1.78E-05	ADA, CD79B, CEBPE, IL10, IL27, ITGAM, LAT, SPI1, SPN, STAT5A, TLR9, TNFSF8, VAV1	13
Hematological System Development and Function	differentiation	differentiation of B lymphocytes	1.41E-04	CD79B, IL10, LAT, STAT5A, TLR9, TNFSF8	6
Hematological System Development and Function	differentiation	differentiation of regulatory T lymphocytes	1.93E-03	IL10, IL27	2
Hematological System Development and Function	differentiation	differentiation of leukocyte cell lines	2.51E-03	CEBPE, IL10, SPI1, STAT5A	4

¬© 2000-2009 Ingenuity Systems, Inc. All rights reserved.

Category	Biological Function	Function Annotation	P-Value	Molecules	# Molecules
Hematological System Development and Function	differentiation	differentiation of phagocytes	4.09E-03	CEBPE, IL10, ITGAM, SPI1	4
Hematological System Development and Function	differentiation	differentiation of erythroblasts	4.10E-03	SPI1, STAT5A	2
Hematological System Development and Function	differentiation	differentiation of hematopoietic progenitor cells	4.61E-03	IL10, NFE2, SPI1, STAT5A	4
Hematological System Development and Function	differentiation	differentiation of dendritic cells	6.53E-03	IL10, SPI1, TLR9	3
Hematological System Development and Function	differentiation	differentiation of natural killer cells	7.00E-03	IL10, STAT5A	2
Hematological System Development and Function	differentiation	differentiation of Langerhans cell precursors	7.48E-03	IL10	1
Hematological System Development and Function	differentiation	differentiation of Tr1 cells	7.48E-03	IL27	1
Hematological System Development and Function	differentiation	differentiation of monocytes	8.46E-03	IL10, SPI1, TLR9	3
Hematological System Development and Function	differentiation	differentiation of antigen presenting cells	8.46E-03	CEBPE, IL10, SPI1, TLR9	4
Hematological System Development and Function	differentiation	differentiation of granulocytes	9.76E-03	CEBPE, SPI1, STAT5A	3
Hematological System Development and Function	differentiation	differentiation of pre-B lymphocytes	1.27E-02	CD79B, LAT	2
Hematological System Development and Function	adhesion	adhesion of mononuclear leukocytes	5.39E-06	CD48, FUT7, IL10, ITGAM, OSM, SELPLG, SPN, VAV1	8
Hematological System Development and Function	adhesion	adhesion of leukocytes	8.15E-06	CD48, FUT7, ICAM3, IL10, ITGAM, OSM, SELPLG, SPI1, SPN, VAV1	10
Hematological System Development and Function	adhesion	adhesion of antigen presenting cells	7.96E-05	CD48, ICAM3, ITGAM, SPI1	4
Hematological System Development and Function	adhesion	adhesion of Th1 cells	8.15E-04	FUT7, SELPLG	2
Hematological System Development and Function	adhesion	adhesion of lymphocytes	9.97E-04	CD48, FUT7, OSM, SELPLG, SPN	5
Hematological System Development and Function	adhesion	adhesion of phagocytes	1.49E-03	ITGAM, SELPLG, SPI1, VAV1	4
Hematological System Development and Function	adhesion	adhesion of dendritic cells	1.93E-03	ICAM3, ITGAM	2
Hematological System Development and Function	adhesion	adhesion of granulocytes	2.51E-03	ICAM3, ITGAM, SELPLG, VAV1	4
Hematological System Development and Function	adhesion	adhesion of T lymphocytes	2.76E-03	CD48, FUT7, SELPLG, SPN	4
Hematological System Development and Function	adhesion	adhesion of monocytes	3.78E-03	IL10, ITGAM, VAV1	3
Hematological System Development and Function	adhesion	adhesion of neutrophils	6.53E-03	ITGAM, SELPLG, VAV1	3
Hematological System Development and Function	adhesion	adhesion of eosinophils	1.37E-02	ITGAM, SELPLG	2
Hematological System Development and Function	development	development of T lymphocytes	8.74E-06	ADA, CD6, IL10, IL27, LAT, OSM, SPI1, STAT5A, VAV1	9
Hematological System Development and Function	development	development of helper T lymphocytes	4.89E-03	IL10, IL27, STAT5A	3
Hematological System Development and Function	development	delay in initiation of development of lymphoid dendritic cells	7.48E-03	SPI1	1
Hematological System Development and Function	development	development of dendritic cells	1.16E-02	IL10, SPI1	2
Hematological System Development and Function	development	development of Th2 cells	1.37E-02	IL10, STAT5A	2
Hematological System Development and Function	cell movement	cell movement of granulocytes	1.87E-05	CD48, FUT7, IL10, ITGAM, LSP1, LTC4S, OSM, SELPLG, SPI1, SPN	10
Hematological System Development and Function	cell movement	cell movement of leukocytes	5.73E-05	CD48, FUT7, IL10, IL18BP, ITGAM, LSP1, LTC4S, OSM, PTGDR, SELPLG, SPI1, SPN, STAT5A	13
Hematological System Development and Function	cell movement	cell movement of Th1 cells	2.12E-04	FUT7, IL10, SELPLG	3
Hematological System Development and Function	cell movement	cell movement of eosinophils	3.28E-04	CD48, IL10, ITGAM, LTC4S, OSM	5
Hematological System Development and Function	cell movement	cell movement of neutrophils	4.43E-04	FUT7, IL10, ITGAM, LSP1, SELPLG, SPI1, SPN	7
Hematological System Development and Function	cell movement	cell movement of Th2 cells	3.48E-03	IL10, SELPLG	2
Hematological System Development and Function	cell movement	cell movement of lymphocytes	4.17E-03	FUT7, IL10, IL18BP, PTGDR, SELPLG, STAT5A	6
Hematological System Development and Function	binding	binding of phagocytes	6.00E-05	FUT7, IL10, ITGAM, LSP1, SELPLG	5
Hematological System Development and Function	binding	binding of neutrophils	6.97E-05	FUT7, ITGAM, LSP1, SELPLG	4
Hematological System Development and Function	binding	binding of leukocytes	1.48E-04	CD6, CD48, FUT7, IL10, ITGAM, LSP1, SELPLG	7
Hematological System Development and Function	binding	binding of mononuclear leukocytes	5.94E-04	CD6, FUT7, IL10, ITGAM, SELPLG	5
Hematological System Development and Function	binding	binding of helper T lymphocytes	1.51E-03	FUT7, SELPLG	2
Hematological System Development and Function	binding	binding of blood platelets	4.31E-03	ICAM4 (includes EG:3386), NFE2, SELPLG	3
Hematological System Development and Function	binding	binding of T lymphocytes	6.53E-03	CD6, FUT7, SELPLG	3
Hematological System Development and Function	binding	binding of monocytes	1.27E-02	IL10, ITGAM	2
Hematological System Development and Function	aggregation	aggregation of granulocytes	6.42E-05	ICAM3, ITGAM, SELPLG	3
Hematological System Development and Function	aggregation	aggregation of leukocytes	3.19E-04	ICAM3, ITGAM, SELPLG, SPN	4
Hematological System Development and Function	aggregation	aggregation of neutrophils	1.51E-03	ITGAM, SELPLG	2
Hematological System Development and Function	aggregation	aggregation of blood cells	4.91E-03	ICAM3, ITGAM, SELPLG, SPN, VAV1	5
Hematological System Development and Function	aggregation	aggregation of T lymphocytes	7.00E-03	ICAM3, SPN	2
Hematological System Development and Function	recruitment	recruitment of granulocytes	9.67E-05	IL10, ITGAM, LSP1, LYZ, PYCARD, STAT5A	6
Hematological System Development and Function	recruitment	recruitment of leukocytes	1.11E-04	IL10, ITGAM, LSP1, LYZ, PYCARD, SELPLG, STAT5A	7
Hematological System Development and Function	recruitment	recruitment of neutrophils	2.91E-04	IL10, ITGAM, LSP1, LYZ, PYCARD	5
Hematological System Development and Function	infiltration	infiltration of leukocytes	9.95E-05	CD48, FUT7, IL10, IL18BP, ITGAM, LTC4S, OSM, SELPLG, STAT5A	9
Hematological System Development and Function	infiltration	infiltration of eosinophils	2.66E-04	CD48, IL10, LTC4S, OSM	4
Hematological System Development and Function	infiltration	infiltration of granulocytes	6.33E-04	CD48, FUT7, IL10, ITGAM, LTC4S, OSM	6
Hematological System Development and Function	infiltration	infiltration of lymphocytes	5.18E-03	IL10, IL18BP, SELPLG, STAT5A	4
Hematological System Development and Function	infiltration	infiltration of T lymphocytes	8.46E-03	IL10, IL18BP, SELPLG	3
Hematological System Development and Function	hematopoiesis	hematopoiesis	1.35E-04	ADA, CD6, CD79B, CEBPE, IL10, IL27, LAT, OSM, SPI1, SPN, STAT5A, TLR9, TNFSF8, VAV1	14
Hematological System Development and Function	hematological process	hematological process	2.52E-04	ADA, CD6, CD79B, CEBPE, IL10, IL27, LAT, NFE2, OSM, SPI1, SPN, STAT5A, TLR9, TNFSF8, VAV1	15
Hematological System Development and Function	function	function of T lymphocytes	2.91E-04	IL10, LAT, STAT5A, VAV1	4
Hematological System Development and Function	function	function of cytotoxic T cells	7.48E-03	IL10	1
Hematological System Development and Function	generation	generation of Tr1 cells	3.29E-04	IL10, IL27	2
Hematological System Development and Function	generation	generation of naive T lymphocytes	8.15E-04	IL10, IL27	2
Hematological System Development and Function	extravasation	extravasation of leukocytes	6.46E-04	FUT7, LSP1, PRKAR1B	3
Hematological System Development and Function	extravasation	extravasation of plasma cells	7.48E-03	PRKAR1B	1
Hematological System Development and Function	inhibition	inhibition of T lymphocytes	1.04E-03	IL10, IL27, TLR9	3
Hematological System Development and Function	inhibition	inhibition of helper T lymphocytes	1.51E-03	IL10, IL27	2
Hematological System Development and Function	cell rolling	cell rolling of Th1 cells	1.14E-03	FUT7, SELPLG	2
Hematological System Development and Function	cell rolling	cell rolling of leukocytes	6.53E-03	FUT7, ITGAM, SELPLG	3
Hematological System Development and Function	cell rolling	cell rolling of blood platelets	7.48E-03	SELPLG	1
Hematological System Development and Function	cell rolling	cell rolling of pre-B lymphocytes	7.48E-03	SELPLG	1

				¬© 2000-2009 Ingenuity Systems, Inc. All rights reserved.	
**Category**	**Biological Function**	**Function Annotation**	**P-Value**	**Molecules**	**# Molecules**
Hematological System Development and Function	priming	priming of antigen presenting cells	1.51E-03	IL10, TLR9	2
Hematological System Development and Function	maturation	maturation of macrophages	2.40E-03	CEBPE, TLR9	2
Hematological System Development and Function	maturation	arrest in maturation of megakaryocytes	7.48E-03	NFE2	1
Hematological System Development and Function	polarization	polarization of T lymphocytes	3.29E-03	IL27, SPN, VAV1	3
Hematological System Development and Function	growth	growth of lymphocytes	3.42E-03	CD22, IL10, IL27, SPI1, STAT5A	5
Hematological System Development and Function	growth	growth of B lymphocytes	3.53E-03	CD22, IL10, STAT5A	3
Hematological System Development and Function	growth	growth of thymocytes	6.21E-03	IL10, SPI1	2
Hematological System Development and Function	growth	growth of T lymphocytes	7.17E-03	IL10, IL27, SPI1, STAT5A	4
Hematological System Development and Function	homing	homing of leukocytes	4.13E-03	FUT7, IL10, ITGAM, LSP1, PTGDR, SELPLG, SPI1	7
Hematological System Development and Function	homing	homing of lymphocytes	1.40E-02	FUT7, IL10, PTGDR, SELPLG	4
Hematological System Development and Function	response	response of lymphocytes	4.61E-03	CD22, IL10, SELPLG, STAT5A	4
Hematological System Development and Function	phagocytosis	phagocytosis of macrophages	5.46E-03	PGLYRP1, SPI1	2
Hematological System Development and Function	cell division	entry into cell division of T lymphocytes	7.48E-03	CD37	1
Hematological System Development and Function	cross-priming	cross-priming of antigen presenting cells	7.48E-03	TLR9	1
Hematological System Development and Function	lymphopoiesis	lymphopoiesis of lymphoid cells	7.48E-03	SPI1	1
Hematological System Development and Function	progression	progression of pro-B lymphocytes	7.48E-03	CD79B	1
Hematological System Development and Function	repopulation	repopulation of thymocytes	7.48E-03	DOK2	1
Hematological System Development and Function	homeostasis	homeostasis of T lymphocytes	7.84E-03	IL10, STAT5A	2
Hematological System Development and Function	expansion	expansion of lymphocytes	7.93E-03	IL10, IL27, SPI1, STAT5A	4
Hematological System Development and Function	survival	survival of leukocyte cell lines	1.07E-02	CEBPE, IL10, STAT5A	3
Hematological System Development and Function	survival	survival of eosinophils	1.16E-02	IL10, TNFSF8	2
Hematological System Development and Function	chemotaxis	chemotaxis of granulocytes	1.36E-02	IL10, ITGAM, LSP1, SPI1	4
Antimicrobial Response	antibacterial response	antibacterial response of organism	1.27E-06	CEBPE, IL10, LYZ, NCF1, PGLYRP1, SPN, TLR9	7
Antimicrobial Response	antiviral response	antiviral response of animal	5.46E-03	TLR9, VAV1	2
Antimicrobial Response	antiviral response	antiviral response of myeloid dendritic cells	7.48E-03	TLR9	1
Cellular Function and Maintenance	phagocytosis	phagocytosis	1.35E-06	CD93, CEBPE, IL10, ITGAM, LAT, PGLYRP1, SPI1, TLR9, VAV1	9
Cellular Function and Maintenance	phagocytosis	phagocytosis of eukaryotic cells	5.98E-06	CD93, ITGAM, LAT, PGLYRP1, SPI1, TLR9, VAV1	7
Cellular Function and Maintenance	phagocytosis	phagocytosis of normal cells	1.87E-04	CD93, ITGAM, PGLYRP1, SPI1, TLR9	5
Cellular Function and Maintenance	phagocytosis	phagocytosis of leukocytes	2.42E-04	CD93, ITGAM, PGLYRP1, SPI1	4
Cellular Function and Maintenance	phagocytosis	phagocytosis of fibroblast cell lines	3.29E-04	LAT, VAV1	2
Cellular Function and Maintenance	phagocytosis	phagocytosis of kidney cell lines	5.46E-04	LAT, VAV1	2
Cellular Function and Maintenance	phagocytosis	phagocytosis of cell lines	2.24E-03	LAT, SPI1, VAV1	3
Cellular Function and Maintenance	phagocytosis	phagocytosis of phagocytes	2.24E-03	ITGAM, PGLYRP1, SPI1	3
Cellular Function and Maintenance	phagocytosis	phagocytosis of macrophages	5.46E-03	PGLYRP1, SPI1	2
Cellular Function and Maintenance	function	function of T lymphocytes	2.91E-04	IL10, LAT, STAT5A, VAV1	4
Cellular Function and Maintenance	function	function of cytotoxic T cells	7.48E-03	IL10	1
Cellular Function and Maintenance	uptake	uptake of cells	3.61E-04	CEBPE, ITGAM, SPI1	3
Cellular Function and Maintenance	cytostasis	cytostasis	2.76E-03	ADA, CEBPE, NR1I2, PGLYRP1, SPI1, TNFSF8, VAV1	7
Cellular Function and Maintenance	cytostasis	cytostasis of eukaryotic cells	5.39E-03	ADA, CEBPE, NR1I2, SPI1, TNFSF8, VAV1	6
Cellular Function and Maintenance	progression	progression of cells	4.10E-03	CD79B, RASSF1	2
Cellular Function and Maintenance	progression	progression of pro-B lymphocytes	7.48E-03	CD79B	1
Cellular Function and Maintenance	exocytosis	exocytosis of cytotoxic granule	7.48E-03	VAV1	1
Cellular Function and Maintenance	homeostasis	homeostasis of T lymphocytes	7.84E-03	IL10, STAT5A	2
Cellular Function and Maintenance	organization	organization of cytoskeleton	1.40E-02	ICAM3, IL10, SPI1, VAV1	4
Cell-To-Cell Signaling and Interaction	activation	activation of mononuclear leukocytes	2.22E-06	ADA, CD22, CD48, CD93, DOK3, ICAM3, IL10, IL27, LAT, SPN, STAT5A, TLR9, VAV1	13
Cell-To-Cell Signaling and Interaction	activation	activation of leukocytes	3.46E-06	ADA, CD22, CD48, CD93, CEBPE, DOK3, ICAM3, IL10, IL27, ITGAM, LAT, SPN, STAT5A, TLR9, VAV1	15
Cell-To-Cell Signaling and Interaction	activation	activation of lymphocytes	7.45E-06	ADA, CD22, CD48, DOK3, ICAM3, IL10, IL27, LAT, SPN, STAT5A, TLR9, VAV1	12
Cell-To-Cell Signaling and Interaction	activation	activation of T lymphocytes	1.10E-05	ADA, CD48, ICAM3, IL10, IL27, LAT, SPN, STAT5A, TLR9, VAV1	10
Cell-To-Cell Signaling and Interaction	activation	activation of eukaryotic cells	1.88E-05	ADA, CD22, CD48, CD93, CEBPE, DOK3, ICAM3, IL10, IL27, ITGAM, LAT, NUAK1, OXT, SPN, STAT5A, TLR9, VAV1	17
Cell-To-Cell Signaling and Interaction	activation	activation of normal cells	2.82E-05	ADA, CD22, CD48, CD93, CEBPE, DOK3, ICAM3, IL10, IL27, ITGAM, LAT, OXT, SPN, STAT5A, TLR9, VAV1	16
Cell-To-Cell Signaling and Interaction	activation	activation of B lymphocytes	2.27E-04	ADA, CD22, DOK3, IL10, VAV1	5
Cell-To-Cell Signaling and Interaction	activation	activation of helper T lymphocytes	7.34E-04	IL10, IL27, TLR9	3
Cell-To-Cell Signaling and Interaction	activation	activation of plasmacytoid dendritic cells	8.15E-04	IL10, TLR9	2
Cell-To-Cell Signaling and Interaction	activation	activation of Th1 cells	2.40E-03	IL10, IL27	2
Cell-To-Cell Signaling and Interaction	activation	activation of mast cells	2.43E-03	CD48, IL10, ITGAM	3
Cell-To-Cell Signaling and Interaction	activation	activation of phagocytes	2.91E-03	CEBPE, IL10, ITGAM, TLR9, VAV1	5
Cell-To-Cell Signaling and Interaction	activation	activation of peritoneal macrophages	4.76E-03	CEBPE, IL10	2
Cell-To-Cell Signaling and Interaction	activation	activation of neutrophils	1.07E-02	IL10, ITGAM, VAV1	3
Cell-To-Cell Signaling and Interaction	adhesion	adhesion of mononuclear leukocytes	5.39E-06	CD48, FUT7, IL10, ITGAM, OSM, SELPLG, SPN, VAV1	8
Cell-To-Cell Signaling and Interaction	adhesion	adhesion of leukocytes	8.15E-06	CD48, FUT7, ICAM3, IL10, ITGAM, OSM, SELPLG, SPI1, SPN, VAV1	10
Cell-To-Cell Signaling and Interaction	adhesion	adhesion of eukaryotic cells	3.83E-05	ALOX15B, CD48, COL7A1, FUT7, ICAM3, IL10, ITGAM, LAT, OSM, RASSF1, SELPLG, SPI1, SPN, VAV1	14
Cell-To-Cell Signaling and Interaction	adhesion	adhesion of cells	4.34E-05	ALOX15B, CD48, CD93, CLEC4A, COL7A1, FUT7, ICAM3, IL10, ITGAM, LAT, LGALS7, OSM, RASSF1, SELPLG, SPI1, SPN, STAT5A, VAV1	18
Cell-To-Cell Signaling and Interaction	adhesion	adhesion of normal cells	7.06E-05	CD48, COL7A1, FUT7, ICAM3, IL10, ITGAM, OSM, SELPLG, SPI1, SPN, VAV1	11
Cell-To-Cell Signaling and Interaction	adhesion	adhesion of antigen presenting cells	7.96E-05	CD48, ICAM3, ITGAM, SPI1	4
Cell-To-Cell Signaling and Interaction	adhesion	adhesion of lymphatic system cells	3.67E-04	ICAM3, ITGAM, SELPLG, SPN, VAV1	5
Cell-To-Cell Signaling and Interaction	adhesion	adhesion of Th1 cells	8.15E-04	FUT7, SELPLG	2
Cell-To-Cell Signaling and Interaction	adhesion	adhesion of lymphocytes	9.97E-04	CD48, FUT7, OSM, SELPLG, SPN	5
Cell-To-Cell Signaling and Interaction	adhesion	adhesion of tumor cell lines	1.00E-03	ALOX15B, FUT7, ITGAM, LAT, RASSF1, SELPLG, SPN	7
Cell-To-Cell Signaling and Interaction	adhesion	adhesion of phagocytes	1.49E-03	ITGAM, SELPLG, SPI1, VAV1	4

				¬© 2000-2009 Ingenuity Systems, Inc. All rights reserved.	
**Category**	**Biological Function**	**Function Annotation**	**P-Value**	**Molecules**	**# Molecules**
Cell-To-Cell Signaling and Interaction	adhesion	adhesion of dendritic cells	1.93E-03	ICAM3, ITGAM	2
Cell-To-Cell Signaling and Interaction	adhesion	adhesion of granulocytes	2.51E-03	ICAM3, ITGAM, SELPLG, VAV1	4
Cell-To-Cell Signaling and Interaction	adhesion	adhesion of T lymphocytes	2.76E-03	CD48, FUT7, SELPLG, SPN	4
Cell-To-Cell Signaling and Interaction	adhesion	adhesion of monocytes	3.78E-03	IL10, ITGAM, VAV1	3
Cell-To-Cell Signaling and Interaction	adhesion	adhesion of neutrophils	6.53E-03	ITGAM, SELPLG, VAV1	3
Cell-To-Cell Signaling and Interaction	adhesion	adhesion of leukemia cell lines	7.65E-03	ITGAM, LAT, SELPLG	3
Cell-To-Cell Signaling and Interaction	adhesion	adhesion of eosinophils	1.37E-02	ITGAM, SELPLG	2
Cell-To-Cell Signaling and Interaction	phagocytosis	phagocytosis of eukaryotic cells	5.98E-06	CD93, ITGAM, LAT, PGLYRP1, SPI1, TLR9, VAV1	7
Cell-To-Cell Signaling and Interaction	phagocytosis	phagocytosis of normal cells	1.87E-04	CD93, ITGAM, PGLYRP1, SPI1, TLR9	5
Cell-To-Cell Signaling and Interaction	phagocytosis	phagocytosis of leukocytes	2.42E-04	CD93, ITGAM, PGLYRP1, SPI1	4
Cell-To-Cell Signaling and Interaction	phagocytosis	phagocytosis of fibroblast cell lines	3.29E-04	LAT, VAV1	2
Cell-To-Cell Signaling and Interaction	phagocytosis	phagocytosis of kidney cell lines	5.46E-04	LAT, VAV1	2
Cell-To-Cell Signaling and Interaction	phagocytosis	phagocytosis of cell lines	2.24E-03	LAT, SPI1, VAV1	3
Cell-To-Cell Signaling and Interaction	phagocytosis	phagocytosis of phagocytes	2.24E-03	ITGAM, PGLYRP1, SPI1	3
Cell-To-Cell Signaling and Interaction	phagocytosis	phagocytosis of macrophages	5.46E-03	PGLYRP1, SPI1	2
Cell-To-Cell Signaling and Interaction	binding	binding of blood cells	1.58E-05	CD6, CD48, FUT7, ICAM4 (includes EG:3386), IL10, ITGAM, LSP1, NFE2, SELPLG	9
Cell-To-Cell Signaling and Interaction	binding	binding of phagocytes	6.00E-05	FUT7, IL10, ITGAM, LSP1, SELPLG	5
Cell-To-Cell Signaling and Interaction	binding	binding of neutrophils	6.97E-05	FUT7, ITGAM, LSP1, SELPLG	4
Cell-To-Cell Signaling and Interaction	binding	binding of leukocytes	1.48E-04	CD6, CD48, FUT7, IL10, ITGAM, LSP1, SELPLG	7
Cell-To-Cell Signaling and Interaction	binding	binding of mononuclear leukocytes	5.94E-04	CD6, FUT7, IL10, ITGAM, SELPLG	5
Cell-To-Cell Signaling and Interaction	binding	binding of eukaryotic cells	6.10E-04	CD6, CD48, FUT7, ICAM4 (includes EG:3386), IL10, ITGAM, LSP1, NFE2, OSM, SELPLG, SPN	11
Cell-To-Cell Signaling and Interaction	binding	binding of leukemia cell lines	7.52E-04	FUT7, ITGAM, SELPLG, SPN	4
Cell-To-Cell Signaling and Interaction	binding	binding of helper T lymphocytes	1.51E-03	FUT7, SELPLG	2
Cell-To-Cell Signaling and Interaction	binding	binding of gonadal cell lines	4.04E-03	CD48, FUT7, ITGAM	3
Cell-To-Cell Signaling and Interaction	binding	binding of blood platelets	4.31E-03	ICAM4 (includes EG:3386), NFE2, SELPLG	3
Cell-To-Cell Signaling and Interaction	binding	binding of T lymphocytes	6.53E-03	CD6, FUT7, SELPLG	3
Cell-To-Cell Signaling and Interaction	binding	binding of tumor cell lines	7.36E-03	FUT7, ITGAM, OSM, SELPLG, SPN	5
Cell-To-Cell Signaling and Interaction	binding	binding of spleen cell lines	7.48E-03	ITGAM	1
Cell-To-Cell Signaling and Interaction	binding	binding of monocytes	1.27E-02	IL10, ITGAM	2
Cell-To-Cell Signaling and Interaction	response	response of cells	3.30E-05	CD22, IL10, ITGAM, KLRG1, LAT, LSP1, NCF1, OSM, SELPLG, SPN, STAT5A, TLR9	12
Cell-To-Cell Signaling and Interaction	response	response of leukocytes	1.91E-04	CD22, IL10, ITGAM, SELPLG, STAT5A, TLR9	6
Cell-To-Cell Signaling and Interaction	response	response of antigen presenting cells	4.22E-04	IL10, ITGAM, TLR9	3
Cell-To-Cell Signaling and Interaction	response	response of lymphocytes	4.61E-03	CD22, IL10, SELPLG, STAT5A	4
Cell-To-Cell Signaling and Interaction	response	response of embryonic cell lines	7.48E-03	TLR9	1
Cell-To-Cell Signaling and Interaction	response	response of epithelial cell lines	7.48E-03	TLR9	1
Cell-To-Cell Signaling and Interaction	response	response of kidney cell lines	7.48E-03	TLR9	1
Cell-To-Cell Signaling and Interaction	recruitment	recruitment of cells	5.15E-05	IL10, ITGAM, LSP1, LYZ, PYCARD, SELPLG, SOD3, STAT5A	8
Cell-To-Cell Signaling and Interaction	recruitment	recruitment of granulocytes	9.67E-05	IL10, ITGAM, LSP1, LYZ, PYCARD, STAT5A	6
Cell-To-Cell Signaling and Interaction	recruitment	recruitment of leukocytes	1.11E-04	IL10, ITGAM, LSP1, LYZ, PYCARD, SELPLG, STAT5A	7
Cell-To-Cell Signaling and Interaction	recruitment	recruitment of neutrophils	2.91E-04	IL10, ITGAM, LSP1, LYZ, PYCARD	5
Cell-To-Cell Signaling and Interaction	priming	priming of antigen presenting cells	1.51E-03	IL10, TLR9	2
Cell-To-Cell Signaling and Interaction	selection	selection of lymphocytes	2.43E-03	SPN, TNFSF8, VAV1	3
Cell-To-Cell Signaling and Interaction	cross-priming	cross-priming of antigen presenting cells	7.48E-03	TLR9	1
Cell-To-Cell Signaling and Interaction	modification	modification of focal adhesions	7.48E-03	S100A4	1
Cell-To-Cell Signaling and Interaction	attachment	attachment of leukocytes	1.06E-02	ITGAM, VAV1	2
Cell-To-Cell Signaling and Interaction	induction	induction of cells	1.32E-02	IL10, OSM, TLR9	3
Immune Cell Trafficking	activation	activation of mononuclear leukocytes	2.22E-06	ADA, CD22, CD48, CD93, DOK3, ICAM3, IL10, IL27, LAT, SPN, STAT5A, TLR9, VAV1	13
Immune Cell Trafficking	activation	activation of leukocytes	3.46E-06	ADA, CD22, CD48, CD93, CEBPE, DOK3, ICAM3, IL10, IL27, ITGAM, LAT, SPN, STAT5A, TLR9, VAV1	15
Immune Cell Trafficking	activation	activation of lymphocytes	7.45E-06	ADA, CD22, CD48, DOK3, ICAM3, IL10, IL27, LAT, SPN, STAT5A, TLR9, VAV1	12
Immune Cell Trafficking	activation	activation of T lymphocytes	1.10E-05	ADA, CD48, ICAM3, IL10, IL27, LAT, SPN, STAT5A, TLR9, VAV1	10
Immune Cell Trafficking	activation	activation of B lymphocytes	2.27E-04	ADA, CD22, DOK3, IL10, VAV1	5
Immune Cell Trafficking	activation	activation of helper T lymphocytes	7.34E-04	IL10, IL27, TLR9	3
Immune Cell Trafficking	activation	activation of plasmacytoid dendritic cells	8.15E-04	IL10, TLR9	2
Immune Cell Trafficking	activation	activation of Th1 cells	2.40E-03	IL10, IL27	2
Immune Cell Trafficking	activation	activation of mast cells	2.43E-03	CD48, IL10, ITGAM	3
Immune Cell Trafficking	activation	activation of phagocytes	2.91E-03	CEBPE, IL10, ITGAM, TLR9, VAV1	5
Immune Cell Trafficking	activation	activation of peritoneal macrophages	4.76E-03	CEBPE, IL10	2
Immune Cell Trafficking	activation	activation of neutrophils	1.07E-02	IL10, ITGAM, VAV1	3
Immune Cell Trafficking	adhesion	adhesion of mononuclear leukocytes	5.39E-06	CD48, FUT7, IL10, ITGAM, OSM, SELPLG, SPN, VAV1	8
Immune Cell Trafficking	adhesion	adhesion of leukocytes	8.15E-06	CD48, FUT7, ICAM3, IL10, ITGAM, OSM, SELPLG, SPI1, SPN, VAV1	10
Immune Cell Trafficking	adhesion	adhesion of antigen presenting cells	7.96E-05	CD48, ICAM3, ITGAM, SPI1	4
Immune Cell Trafficking	adhesion	adhesion of Th1 cells	8.15E-04	FUT7, SELPLG	2
Immune Cell Trafficking	adhesion	adhesion of lymphocytes	9.97E-04	CD48, FUT7, OSM, SELPLG, SPN	5
Immune Cell Trafficking	adhesion	adhesion of phagocytes	1.49E-03	ITGAM, SELPLG, SPI1, VAV1	4
Immune Cell Trafficking	adhesion	adhesion of dendritic cells	1.93E-03	ICAM3, ITGAM	2
Immune Cell Trafficking	adhesion	adhesion of granulocytes	2.51E-03	ICAM3, ITGAM, SELPLG, VAV1	4
Immune Cell Trafficking	adhesion	adhesion of T lymphocytes	2.76E-03	CD48, FUT7, SELPLG, SPN	4
Immune Cell Trafficking	adhesion	adhesion of monocytes	3.78E-03	IL10, ITGAM, VAV1	3
Immune Cell Trafficking	adhesion	adhesion of neutrophils	6.53E-03	ITGAM, SELPLG, VAV1	3
Immune Cell Trafficking	adhesion	adhesion of eosinophils	1.37E-02	ITGAM, SELPLG	2
Immune Cell Trafficking	cell movement	cell movement of granulocytes	1.87E-05	CD48, FUT7, IL10, ITGAM, LSP1, LTC4S, OSM, SELPLG, SPI1, SPN	10

				¬© 2000-2009 Ingenuity Systems, Inc. All rights reserved.	
**Category**	**Biological Function**	**Function Annotation**	**P-Value**	**Molecules**	**# Molecules**
Immune Cell Trafficking	cell movement	cell movement of leukocytes	5.73E-05	CD48, FUT7, IL10, IL18BP, ITGAM, LSP1, LTC4S, OSM, PTGDR, SELPLG, SPI1, SPN, STAT5A	13
Immune Cell Trafficking	cell movement	cell movement of Th1 cells	2.12E-04	FUT7, IL10, SELPLG	3
Immune Cell Trafficking	cell movement	cell movement of eosinophils	3.28E-04	CD48, IL10, ITGAM, LTC4S, OSM	5
Immune Cell Trafficking	cell movement	cell movement of neutrophils	4.43E-04	FUT7, IL10, ITGAM, LSP1, SELPLG, SPI1, SPN	7
Immune Cell Trafficking	cell movement	cell movement of Th2 cells	3.48E-03	IL10, SELPLG	2
Immune Cell Trafficking	cell movement	cell movement of lymphocytes	4.17E-03	FUT7, IL10, IL18BP, PTGDR, SELPLG, STAT5A	6
Immune Cell Trafficking	recruitment	recruitment of granulocytes	9.67E-05	IL10, ITGAM, LSP1, LYZ, PYCARD, STAT5A	6
Immune Cell Trafficking	recruitment	recruitment of leukocytes	1.11E-04	IL10, ITGAM, LSP1, LYZ, PYCARD, SELPLG, STAT5A	7
Immune Cell Trafficking	recruitment	recruitment of neutrophils	2.91E-04	IL10, ITGAM, LSP1, LYZ, PYCARD	5
Immune Cell Trafficking	infiltration	infiltration of leukocytes	9.95E-05	CD48, FUT7, IL10, IL18BP, ITGAM, LTC4S, OSM, SELPLG, STAT5A	9
Immune Cell Trafficking	infiltration	infiltration of eosinophils	2.66E-04	CD48, IL10, LTC4S, OSM	4
Immune Cell Trafficking	infiltration	infiltration of granulocytes	6.33E-04	CD48, FUT7, IL10, ITGAM, LTC4S, OSM	6
Immune Cell Trafficking	infiltration	infiltration of lymphocytes	5.18E-03	IL10, IL18BP, SELPLG, STAT5A	4
Immune Cell Trafficking	infiltration	infiltration of T lymphocytes	8.46E-03	IL10, IL18BP, SELPLG	3
Immune Cell Trafficking	cell rolling	cell rolling of Th1 cells	1.14E-03	FUT7, SELPLG	2
Immune Cell Trafficking	cell rolling	cell rolling of leukocytes	6.53E-03	FUT7, ITGAM, SELPLG	3
Immune Cell Trafficking	cell rolling	cell rolling of pre-B lymphocytes	7.48E-03	SELPLG	1
Immune Cell Trafficking	homing	homing of leukocytes	4.13E-03	FUT7, IL10, ITGAM, LSP1, PTGDR, SELPLG, SPI1	7
Immune Cell Trafficking	homing	homing of lymphocytes	1.40E-02	FUT7, IL10, PTGDR, SELPLG	4
Immune Cell Trafficking	migration	migration of Langerhans cells	5.46E-03	IL10, PTGDR	2
Immune Cell Trafficking	migration	migration of leukocytes	1.47E-02	CD22, CEBPE, IL10, ITGAM, LSP1, PTGDR, SELPLG, SPI1	8
Immune Cell Trafficking	mobility	mobility of B lymphocytes	7.48E-03	IL10	1
Immune Cell Trafficking	repopulation	repopulation of thymocytes	7.48E-03	DOK2	1
Immune Cell Trafficking	chemotaxis	chemotaxis of granulocytes	1.36E-02	IL10, ITGAM, LSP1, SPI1	4
Tissue Morphology	quantity	quantity of leukocytes	2.29E-06	ADA, CD22, DNMT1, DOK2, IL10, IL18BP, ITGAM, LAT, LSP1, LTC4S, OSM, SELPLG, SPI1, SPN, STAT5A, VAV1	16
Tissue Morphology	quantity	quantity of lymphocytes	3.30E-05	CD22, DNMT1, DOK2, IL10, ITGAM, LAT, LSP1, OSM, SELPLG, SPN, STAT5A, VAV1	12
Tissue Morphology	quantity	quantity of phagocytes	1.95E-04	ADA, IL10, IL18BP, LSP1, LTC4S, SELPLG, SPI1	7
Tissue Morphology	quantity	quantity of cells	3.45E-04	ADA, CD22, DNMT1, DOK2, HPN, IL10, IL18BP, ITGAM, LAT, LSP1, LTC4S, NOX4, OSM, PRKAR1B, PTPRCAP, SELPLG, SPI1, SPN, STAT5A, VAV1	20
Tissue Morphology	quantity	quantity of antigen presenting cells	7.34E-04	ADA, IL10, LSP1, LTC4S, SELPLG, SPI1	6
Tissue Morphology	quantity	quantity of T lymphocytes	9.18E-04	DNMT1, IL10, ITGAM, LAT, OSM, SELPLG, STAT5A, VAV1	8
Tissue Morphology	quantity	quantity of macrophages	1.29E-03	ADA, LSP1, LTC4S, SELPLG, SPI1	5
Tissue Morphology	quantity	quantity of neutrophils	2.89E-03	ADA, IL10, IL18BP, SELPLG	4
Tissue Morphology	quantity	quantity of B lymphocytes	7.17E-03	CD22, IL10, SPN, STAT5A, VAV1	5
Tissue Morphology	quantity	quantity of dorsal horn cells	7.48E-03	PRKAR1B	1
Tissue Morphology	quantity	quantity of lymphocyte precursor cells	7.48E-03	DOK2	1
Tissue Morphology	quantity	quantity of lymphatic system cells	1.31E-02	ADA, IL10, IL18BP, PTPRCAP, SELPLG	5
Tissue Morphology	disorganization	disorganization of tissue	2.40E-03	ADA, LAT	2
Tissue Morphology	disorganization	disorganization of hepatic plate	7.48E-03	ADA	1
Tissue Morphology	contraction	contraction of uterine tissue	7.48E-03	OXT	1
Tissue Morphology	morphology	morphology of synovial membrane	7.48E-03	OSM	1
Tissue Morphology	stimulation	stimulation of nasal cartilage	7.48E-03	OSM	1
Tissue Morphology	structural integrity	structural integrity of epithelial barrier	7.48E-03	IL10	1
Tissue Morphology	permeability	permeability of vascular tissue	1.06E-02	IL18BP, LSP1	2
Cell Death	cytotoxicity	cytotoxicity of natural killer cells	2.78E-06	CD6, CD48, IL10, LAT, PTGDR, SPN, VAV1	7
Cell Death	cytotoxicity	cytotoxicity of eukaryotic cells	4.56E-05	CD6, CD48, IL10, LAT, POR, PTGDR, SPN, VAV1	8
Cell Death	cell death	cell death	7.04E-06	ADA, AIF1, ALOX15B, CD22, CD48, CD79B, CEBPE, DAB2IP, DUSP2, GPX2, HOXA7, HPN, ICAM3, IL10, ITGAM, LAT, LGALS7, LSP1, LYZ, NCF1, NLRP3, NOX4, NR1I2, NUAK1, OSM, PADI4, PGLYRP1, PTPRO, PYCARD, RASSF1, S100A4, SOD3, SPI1, SPN, STAT5A, TLR9, TNFSF8, TRAF1, VAV1	39
Cell Death	cell death	cell death of cell lines	1.11E-05	CD22, CD48, CD79B, CEBPE, GPX2, HOXA7, HPN, ICAM3, IL10, ITGAM, LGALS7, LSP1, NLRP3, NR1I2, NUAK1, PADI4, PGLYRP1, PTPRO, PYCARD, RASSF1, S100A4, SOD3, SPI1, SPN, STAT5A, TRAF1, VAV1	27
Cell Death	cell death	cell death of eukaryotic cells	2.67E-05	ADA, CD22, CD48, CD79B, CEBPE, DAB2IP, GPX2, HOXA7, HPN, ICAM3, IL10, ITGAM, LAT, LGALS7, LSP1, NCF1, NLRP3, NOX4, NR1I2, NUAK1, PADI4, PGLYRP1, PTPRO, PYCARD, RASSF1, S100A4, SOD3, SPI1, SPN, STAT5A, TLR9, TRAF1, VAV1	33
Cell Death	cell death	cell death of tumor cell lines	4.02E-05	CD48, CD79B, GPX2, HOXA7, HPN, IL10, ITGAM, LGALS7, LSP1, NLRP3, NR1I2, NUAK1, PADI4, PTPRO, PYCARD, RASSF1, S100A4, SOD3, SPI1, SPN, TRAF1, VAV1	22
Cell Death	cell death	cell death of leukocytes	9.14E-04	ADA, CD22, IL10, ITGAM, LAT, PYCARD, SPN, STAT5A, TLR9, TRAF1	10
Cell Death	cell death	cell death of lymphoma cell lines	7.36E-03	CD48, CD79B, HOXA7, IL10, LSP1	5
Cell Death	cell death	cell death of lymphocytes	7.52E-03	ADA, CD22, IL10, LAT, SPN, STAT5A, TRAF1	7
Cell Death	cell death	cell death of T lymphocytes	8.90E-03	ADA, IL10, LAT, SPN, STAT5A, TRAF1	6
Cell Death	apoptosis	apoptosis	8.38E-06	ADA, ALOX15B, CD22, CD48, CD79B, CEBPE, DAB2IP, DUSP2, GPX2, HOXA7, HPN, ICAM3, IL10, ITGAM, LGALS7, LSP1, NCF1, NLRP3, NOX4, NR1I2, OSM, PADI4, PGLYRP1, PTPRO, PYCARD, RASSF1, S100A4, SOD3, SPI1, SPN, STAT5A, TLR9, TNFSF8, TRAF1, VAV1	35
Cell Death	apoptosis	apoptosis of eukaryotic cells	1.44E-05	ADA, CD22, CD48, CD79B, CEBPE, DAB2IP, GPX2, HOXA7, HPN, ICAM3, IL10, ITGAM, LGALS7, LSP1, NCF1, NOX4, NR1I2, PADI4, PGLYRP1, PTPRO, PYCARD, RASSF1, S100A4, SOD3, SPI1, SPN, STAT5A, TLR9, TRAF1, VAV1	30
Cell Death	apoptosis	apoptosis of cell lines	5.58E-05	CD48, CD79B, CEBPE, GPX2, HPN, ICAM3, IL10, ITGAM, LGALS7, LSP1, NR1I2, PADI4, PGLYRP1, PTPRO, PYCARD, RASSF1, S100A4, SOD3, SPI1, SPN, STAT5A, TRAF1, VAV1	23
Cell Death	apoptosis	apoptosis of plasmacytoid dendritic cells	3.29E-04	IL10, TLR9	2

¬© 2000-2009 Ingenuity Systems, Inc. All rights reserved.

Category	Biological Function	Function Annotation	P-Value	Molecules	# Molecules
Cell Death	apoptosis	apoptosis of tumor cell lines	1.10E-03	CD48, CD79B, GPX2, HPN, ITGAM, LGALS7, LSP1, NR1I2, PADI4, PTPRO, PYCARD, RASSF1, S100A4, SOD3, SPN, TRAF1, VAV1	17
Cell Death	apoptosis	apoptosis of leukocytes	5.48E-03	ADA, CD22, IL10, ITGAM, SPN, STAT5A, TLR9, TRAF1	8
Cell Death	apoptosis	apoptosis of myeloid dendritic cells	7.48E-03	IL10	1
Cell Death	apoptosis	apoptosis of peripheral blood monocytes	7.48E-03	IL10	1
Cell Death	apoptosis	apoptosis of lymphatic system cells	1.18E-02	ADA, IL10, ITGAM, STAT5A	4
Cell Death	apoptosis	apoptosis of lymphocytes	1.41E-02	ADA, CD22, IL10, SPN, STAT5A, TRAF1	6
Cell Death	killing	killing of cells	1.60E-05	CD48, CEBPE, IL10, LYZ, POR, PTPRCAP, SPI1	7
Cell Death	survival	survival of eukaryotic cells	3.92E-04	CEBPE, DGKA, DNMT1, IL10, NFE2, NUAK1, OSM, POR, PPP1R16B, PTPN7, PTPRCAP, PTPRO, PYCARD, SPN, STAT5A, TNFSF8	16
Cell Death	survival	survival of cell lines	5.52E-04	CEBPE, DGKA, IL10, NUAK1, POR, PPP1R16B, PTPN7, PTPRCAP, PTPRO, PYCARD, STAT5A, TNFSF8	12
Cell Death	survival	survival of tumor cell lines	3.31E-03	DGKA, IL10, NUAK1, PPP1R16B, PTPN7, PTPRCAP, PTPRO, PYCARD, TNFSF8	9
Cell Death	survival	survival of Sertoli cells	7.48E-03	OSM	1
Cell Death	survival	survival of primordial germ cells	7.48E-03	OSM	1
Cell Death	survival	survival of cervical cancer cell lines	8.96E-03	DGKA, PPP1R16B, PTPN7, PTPRCAP, PTPRO	5
Cell Death	survival	survival of leukocyte cell lines	1.07E-02	CEBPE, IL10, STAT5A	3
Cell Death	survival	survival of eosinophils	1.16E-02	IL10, TNFSF8	2
Cell Death	survival	survival of blood cells	1.26E-02	IL10, NFE2, SPN, STAT5A, TNFSF8	5
Cell Death	necrosis	necrosis of lymphoma cell lines	5.46E-04	HOXA7, IL10	2
Cell Death	necrosis	necrosis	5.20E-03	ADA, HOXA7, IL10, NUAK1, TRAF1	5
Cell Death	cell viability	cell viability of cytotoxic T cells	7.48E-03	IL10	1
Cell Death	colony survival	colony survival of adenocarcinoma cells	7.48E-03	IL10	1
Cell Death	repopulation	repopulation of lymphoid cells	7.48E-03	DOK2	1
Cell Death	repopulation	repopulation of thymocytes	7.48E-03	DOK2	1
Cellular Development	differentiation	differentiation of blood cells	4.31E-06	ADA, CD79B, CEBPE, IL10, IL27, ITGAM, LAT, LMO2, NFE2, SPI1, SPN, STAT5A, TLR9, TNFSF8, VAV1	15
Cellular Development	differentiation	differentiation of lymphocytes	1.26E-05	ADA, CD79B, IL10, IL27, LAT, SPI1, SPN, STAT5A, TLR9, TNFSF8, VAV1	11
Cellular Development	differentiation	differentiation of leukocytes	1.78E-05	ADA, CD79B, CEBPE, IL10, IL27, ITGAM, LAT, SPI1, SPN, STAT5A, TLR9, TNFSF8, VAV1	13
Cellular Development	differentiation	differentiation of B lymphocytes	1.41E-04	CD79B, IL10, LAT, STAT5A, TLR9, TNFSF8	6
Cellular Development	differentiation	differentiation of cells	4.19E-04	ADA, CD79B, CEBPE, DNMT1, IL10, IL27, ITGAM, LAT, LGALS7, LMO2, NFE2, NOX4, NR1I2, ONECUT2, OSM, OXT, S100A4, SPI1, SPN, STAT5A, TLR9, TNFSF8, VAV1	23
Cellular Development	differentiation	differentiation of regulatory T lymphocytes	1.93E-03	IL10, IL27	2
Cellular Development	differentiation	differentiation of leukocyte cell lines	2.51E-03	CEBPE, IL10, SPI1, STAT5A	4
Cellular Development	differentiation	differentiation of phagocytes	4.09E-03	CEBPE, IL10, ITGAM, SPI1	4
Cellular Development	differentiation	differentiation of erythroblasts	4.10E-03	SPI1, STAT5A	2
Cellular Development	differentiation	differentiation of hematopoietic progenitor cells	4.61E-03	IL10, NFE2, SPI1, STAT5A	4
Cellular Development	differentiation	differentiation of dendritic cells	6.53E-03	IL10, SPI1, TLR9	3
Cellular Development	differentiation	differentiation of natural killer cells	7.00E-03	IL10, STAT5A	2
Cellular Development	differentiation	differentiation of Langerhans cell precursors	7.48E-03	IL10	1
Cellular Development	differentiation	differentiation of Tr1 cells	7.48E-03	IL27	1
Cellular Development	differentiation	differentiation of myoepithelial cells	7.48E-03	OXT	1
Cellular Development	differentiation	differentiation of monocytes	8.46E-03	IL10, SPI1, TLR9	3
Cellular Development	differentiation	differentiation of antigen presenting cells	8.46E-03	CEBPE, IL10, SPI1, TLR9	4
Cellular Development	differentiation	differentiation of granulocytes	9.76E-03	CEBPE, SPI1, STAT5A	3
Cellular Development	differentiation	differentiation of pre-B lymphocytes	1.27E-02	CD79B, LAT	2
Cellular Development	differentiation	differentiation of stem cells	1.47E-02	DNMT1, NFE2, OSM, SPI1	4
Cellular Development	developmental process	developmental process of mononuclear leukocytes	6.47E-06	ADA, CD6, CD22, CD79B, IL10, IL27, ITGAM, LAT, OSM, SPI1, SPN, STAT5A, TLR9, TNFSF8, VAV1	15
Cellular Development	developmental process	developmental process of B lymphocytes	9.55E-06	ADA, CD22, CD79B, IL10, LAT, STAT5A, TLR9, TNFSF8, VAV1	9
Cellular Development	developmental process	developmental process of blood cells	1.07E-05	ADA, CD6, CD22, CD79B, CEBPE, IL10, IL27, ITGAM, LAT, LMO2, NFE2, OSM, SPI1, SPN, STAT5A, TLR9, TNFSF8, VAV1	18
Cellular Development	developmental process	developmental process of lymphocytes	1.16E-05	ADA, CD6, CD22, CD79B, IL10, IL27, LAT, OSM, SPI1, SPN, STAT5A, TLR9, TNFSF8, VAV1	14
Cellular Development	developmental process	developmental process of leukocytes	2.62E-05	ADA, CD6, CD22, CD79B, CEBPE, IL10, IL27, ITGAM, LAT, OSM, SPI1, SPN, STAT5A, TLR9, TNFSF8, VAV1	16
Cellular Development	developmental process	developmental process of Tr1 cells	1.65E-04	IL10, IL27	2
Cellular Development	developmental process	developmental process of monocytes	1.33E-03	IL10, ITGAM, SPI1, TLR9	4
Cellular Development	developmental process	developmental process of phagocytes	1.70E-03	CEBPE, IL10, ITGAM, SPI1, TLR9	5
Cellular Development	developmental process	developmental process of myeloid dendritic cells	1.93E-03	IL10, SPI1	2
Cellular Development	developmental process	developmental process of granulocytes	2.06E-03	CEBPE, IL10, SPI1, STAT5A	4
Cellular Development	developmental process	developmental process of multipotential hemopoietic progenitor cells	2.40E-03	SPI1, STAT5A	2
Cellular Development	developmental process	developmental process of pre-B lymphocytes	5.84E-03	CD79B, LAT, VAV1	3
Cellular Development	developmental process	developmental process of macrophages	6.23E-03	CEBPE, IL10, SPI1, TLR9	4
Cellular Development	developmental process	developmental process of bone marrow cells	7.58E-03	CEBPE, IL10, NFE2, SPI1, STAT5A, VAV1	6
Cellular Development	developmental process	developmental process of leukocyte cell lines	8.75E-03	CEBPE, IL10, OSM, SPI1, STAT5A	5
Cellular Development	developmental process	developmental process of stem cells	1.26E-02	DNMT1, NFE2, OSM, SPI1, STAT5A	5
Cellular Development	developmental process	developmental process of smooth muscle cells	1.32E-02	AIF1, OSM, OXT	3
Cellular Development	development	development of T lymphocytes	8.74E-06	ADA, CD6, IL10, IL27, LAT, OSM, SPI1, STAT5A, VAV1	9
Cellular Development	development	development of helper T lymphocytes	4.89E-03	IL10, IL27, STAT5A	3
Cellular Development	development	delay in initiation of development of lymphoid dendritic cells	7.48E-03	SPI1	1
Cellular Development	development	development of dendritic cells	1.16E-02	IL10, SPI1	2

				¬© 2000-2009 Ingenuity Systems, Inc. All rights reserved.	
**Category**	**Biological Function**	**Function Annotation**	**P-Value**	**Molecules**	**# Molecules**
Cellular Development	development	development of cells	1.37E-02	ADA, CD6, DGKA, IL10, IL27, ITGAM, LAT, LMO2, NOX4, ONECUT2, OSM, SMOC2, SPI1, SPN, STAT5A, VAV1	16
Cellular Development	development	development of Th2 cells	1.37E-02	IL10, STAT5A	2
Cellular Development	maturation	maturation of blood cells	9.50E-05	CEBPE, IL10, ITGAM, LAT, NFE2, TLR9, TNFSF8, VAV1	8
Cellular Development	maturation	maturation of leukocytes	2.73E-04	CEBPE, IL10, ITGAM, LAT, TLR9, TNFSF8, VAV1	7
Cellular Development	maturation	maturation of cells	6.84E-04	CEBPE, IL10, ITGAM, KCNE1, LAT, NFE2, TLR9, TNFSF8, VAV1	9
Cellular Development	maturation	maturation of macrophages	2.40E-03	CEBPE, TLR9	2
Cellular Development	maturation	maturation of mononuclear leukocytes	3.60E-03	ITGAM, LAT, TNFSF8, VAV1	4
Cellular Development	maturation	arrest in maturation of megakaryocytes	7.48E-03	NFE2	1
Cellular Development	growth	growth of lymphocytes	3.42E-03	CD22, IL10, IL27, SPI1, STAT5A	5
Cellular Development	growth	growth of B lymphocytes	3.53E-03	CD22, IL10, STAT5A	3
Cellular Development	growth	growth of thymocytes	6.21E-03	IL10, SPI1	2
Cellular Development	growth	growth of T lymphocytes	7.17E-03	IL10, IL27, SPI1, STAT5A	4
Cellular Development	growth	growth of bone marrow cells	7.65E-03	IL10, SPI1, VAV1	3
Cellular Development	angiogenesis	angiogenesis of endothelial cell lines	4.10E-03	DGKA, SMOC2	2
Cellular Development	conversion	conversion of dendritic cells	7.48E-03	IL10	1
Cellular Development	lymphopoiesis	lymphopoiesis of lymphoid cells	7.48E-03	SPI1	1
Cellular Development	expansion	expansion of lymphocytes	7.93E-03	IL10, IL27, SPI1, STAT5A	4
Hematopoiesis	development	development of T lymphocytes	8.74E-06	ADA, CD6, IL10, IL27, LAT, OSM, SPI1, STAT5A, VAV1	9
Hematopoiesis	development	development of helper T lymphocytes	4.89E-03	IL10, IL27, STAT5A	3
Hematopoiesis	development	delay in initiation of development of lymphoid dendritic cells	7.48E-03	SPI1	1
Hematopoiesis	development	development of dendritic cells	1.16E-02	IL10, SPI1	2
Hematopoiesis	development	development of Th2 cells	1.37E-02	IL10, STAT5A	2
Hematopoiesis	differentiation	differentiation of lymphocytes	1.26E-05	ADA, CD79B, IL10, IL27, LAT, SPI1, SPN, STAT5A, TLR9, TNFSF8, VAV1	11
Hematopoiesis	differentiation	differentiation of leukocytes	1.78E-05	ADA, CD79B, CEBPE, IL10, IL27, ITGAM, LAT, SPI1, SPN, STAT5A, TLR9, TNFSF8, VAV1	13
Hematopoiesis	differentiation	differentiation of B lymphocytes	1.41E-04	CD79B, IL10, LAT, STAT5A, TLR9, TNFSF8	6
Hematopoiesis	differentiation	differentiation of regulatory T lymphocytes	1.93E-03	IL10, IL27	2
Hematopoiesis	differentiation	differentiation of leukocyte cell lines	2.51E-03	CEBPE, IL10, SPI1, STAT5A	4
Hematopoiesis	differentiation	differentiation of phagocytes	4.09E-03	CEBPE, IL10, ITGAM, SPI1	4
Hematopoiesis	differentiation	differentiation of erythroblasts	4.10E-03	SPI1, STAT5A	2
Hematopoiesis	differentiation	differentiation of hematopoietic progenitor cells	4.61E-03	IL10, NFE2, SPI1, STAT5A	4
Hematopoiesis	differentiation	differentiation of dendritic cells	6.53E-03	IL10, SPI1, TLR9	3
Hematopoiesis	differentiation	differentiation of natural killer cells	7.00E-03	IL10, STAT5A	2
Hematopoiesis	differentiation	differentiation of Langerhans cell precursors	7.48E-03	IL10	1
Hematopoiesis	differentiation	differentiation of Tr1 cells	7.48E-03	IL27	1
Hematopoiesis	differentiation	differentiation of monocytes	8.46E-03	IL10, SPI1, TLR9	3
Hematopoiesis	differentiation	differentiation of antigen presenting cells	8.46E-03	CEBPE, IL10, SPI1, TLR9	4
Hematopoiesis	differentiation	differentiation of granulocytes	9.76E-03	CEBPE, SPI1, STAT5A	3
Hematopoiesis	differentiation	differentiation of pre-B lymphocytes	1.27E-02	CD79B, LAT	2
Hematopoiesis	hematopoiesis	hematopoiesis	1.35E-04	ADA, CD6, CD79B, CEBPE, IL10, IL27, LAT, OSM, SPI1, SPN, STAT5A, TLR9, TNFSF8, VAV1	14
Hematopoiesis	proliferation	proliferation of hematopoietic cells	3.09E-04	CD22, NFE2, PTPRO, SPI1, STAT5A	5
Hematopoiesis	proliferation	proliferation of hematopoietic progenitor cells	1.49E-03	CD22, NFE2, PTPRO, STAT5A	4
Hematopoiesis	proliferation	proliferation of pre-B lymphocytes	1.37E-02	IL10, SPI1	2
Hematopoiesis	growth	growth of thymocytes	6.21E-03	IL10, SPI1	2
Hematopoiesis	cell rolling	cell rolling of pre-B lymphocytes	7.48E-03	SELPLG	1
Hematopoiesis	colony formation	colony formation of mast precursor cells	7.48E-03	IL10	1
Hematopoiesis	invasion	invasion of proerythroblasts	7.48E-03	SPI1	1
Hematopoiesis	progression	progression of pro-B lymphocytes	7.48E-03	CD79B	1
Hematopoiesis	quantity	quantity of lymphocyte precursor cells	7.48E-03	DOK2	1
Hematopoiesis	repopulation	repopulation of thymocytes	7.48E-03	DOK2	1
Dermatological Diseases and Conditions	dermatological disorder	dermatological disorder	1.03E-05	ADA, CD22, CD52, CD79B, COL7A1, DUSP2, FUT7, GPX2, IL10, IL18BP, OSM, PKP1, SPI1, TLR9, TMC6, TMC8, TNFSF8	17
Dermatological Diseases and Conditions	epidermodysplasia verruciformis	epidermodysplasia verruciformis	5.54E-05	TMC6, TMC8	2
Dermatological Diseases and Conditions	dermatitis	dermatitis	1.74E-04	CD52, DUSP2, FUT7, IL18BP, OSM, SPI1, TNFSF8	7
Dermatological Diseases and Conditions	atopic dermatitis	atopic dermatitis	6.89E-04	DUSP2, FUT7, OSM, SPI1, TNFSF8	5
Dermatological Diseases and Conditions	multiplicity	multiplicity of skin tumor	7.48E-03	RASSF1	1
Dermatological Diseases and Conditions	mycosis fungoides	mycosis fungoides	1.27E-02	CD52, TLR9	2
Cellular Movement	cell movement	cell movement of granulocytes	1.87E-05	CD48, FUT7, IL10, ITGAM, LSP1, LTC4S, OSM, SELPLG, SPI1, SPN	10
Cellular Movement	cell movement	cell movement of eukaryotic cells	3.15E-05	CD48, DGKA, FUT7, HOXA7, IL10, IL18BP, ITGAM, LSP1, LTC4S, OSM, PTGDR, PTPRO, RASSF1, S100A4, SELPLG, SPI1, SPN, STAT5A	18
Cellular Movement	cell movement	cell movement of leukocytes	5.73E-05	CD48, FUT7, IL10, IL18BP, ITGAM, LSP1, LTC4S, OSM, PTGDR, SELPLG, SPI1, SPN, STAT5A	13
Cellular Movement	cell movement	cell movement of normal cells	7.27E-05	CD48, DGKA, FUT7, IL10, IL18BP, ITGAM, LSP1, LTC4S, OSM, PTGDR, S100A4, SELPLG, SPI1, SPN, STAT5A	15
Cellular Movement	cell movement	cell movement	7.75E-05	CD48, DGKA, FUT7, HOXA7, IL10, IL18BP, ITGAM, LSP1, LTC4S, OSM, PTGDR, PTPRO, RASSF1, S100A4, SELPLG, SPI1, SPN, STAT5A, TLR9	19
Cellular Movement	cell movement	cell movement of Th1 cells	2.12E-04	FUT7, IL10, SELPLG	3
Cellular Movement	cell movement	cell movement of eosinophils	3.28E-04	CD48, IL10, ITGAM, LTC4S, OSM	5
Cellular Movement	cell movement	cell movement of neutrophils	4.43E-04	FUT7, IL10, ITGAM, LSP1, SELPLG, SPI1, SPN	7
Cellular Movement	cell movement	cell movement of lung cancer cell lines	2.92E-03	FUT7, S100A4	2
Cellular Movement	cell movement	cell movement of Th2 cells	3.48E-03	IL10, SELPLG	2
Cellular Movement	cell movement	cell movement of lymphocytes	4.17E-03	FUT7, IL10, IL18BP, PTGDR, SELPLG, STAT5A	6

				¬© 2000-2009 Ingenuity Systems, Inc. All rights reserved.	
**Category**	**Biological Function**	**Function Annotation**	**P-Value**	**Molecules**	**# Molecules**
Cellular Movement	extravasation	extravasation of cells	4.54E-05	FUT7, ITGAM, LSP1, PRKAR1B	4
Cellular Movement	extravasation	extravasation of leukocytes	6.46E-04	FUT7, LSP1, PRKAR1B	3
Cellular Movement	extravasation	extravasation of plasma cells	7.48E-03	PRKAR1B	1
Cellular Movement	infiltration	infiltration of leukocytes	9.95E-05	CD48, FUT7, IL10, IL18BP, ITGAM, LTC4S, OSM, SELPLG, STAT5A	9
Cellular Movement	infiltration	infiltration of eosinophils	2.66E-04	CD48, IL10, LTC4S, OSM	4
Cellular Movement	infiltration	infiltration of granulocytes	6.33E-04	CD48, FUT7, IL10, ITGAM, LTC4S, OSM	6
Cellular Movement	infiltration	infiltration of lymphocytes	5.18E-03	IL10, IL18BP, SELPLG, STAT5A	4
Cellular Movement	infiltration	infiltration of T lymphocytes	8.46E-03	IL10, IL18BP, SELPLG	3
Cellular Movement	cell rolling	cell rolling of leukemia cell lines	5.46E-04	FUT7, SELPLG	2
Cellular Movement	cell rolling	cell rolling of Th1 cells	1.14E-03	FUT7, SELPLG	2
Cellular Movement	cell rolling	cell rolling of leukocytes	6.53E-03	FUT7, ITGAM, SELPLG	3
Cellular Movement	cell rolling	cell rolling of blood platelets	7.48E-03	SELPLG	1
Cellular Movement	cell rolling	cell rolling of pre-B lymphocytes	7.48E-03	SELPLG	1
Cellular Movement	cell rolling	cell rolling of lymphatic system cells	1.06E-02	FUT7, SELPLG	2
Cellular Movement	chemotaxis	chemotaxis	1.94E-03	DGKA, HOXA7, IL10, ITGAM, LSP1, PTGDR, SELPLG, SPI1, SPN, TLR9	10
Cellular Movement	chemotaxis	chemotaxis of eukaryotic cells	9.25E-03	DGKA, HOXA7, IL10, ITGAM, LSP1, PTGDR, SELPLG, SPI1	8
Cellular Movement	chemotaxis	chemotaxis of granulocytes	1.36E-02	IL10, ITGAM, LSP1, SPI1	4
Cellular Movement	homing	homing of eukaryotic cells	3.89E-03	DGKA, FUT7, HOXA7, IL10, ITGAM, LSP1, PTGDR, SELPLG, SPI1	9
Cellular Movement	homing	homing of leukocytes	4.13E-03	FUT7, IL10, ITGAM, LSP1, PTGDR, SELPLG, SPI1	7
Cellular Movement	homing	homing of lymphocytes	1.40E-02	FUT7, IL10, PTGDR, SELPLG	4
Cellular Movement	migration	migration of Langerhans cells	5.46E-03	IL10, PTGDR	2
Cellular Movement	migration	migration of leukocytes	1.47E-02	CD22, CEBPE, IL10, ITGAM, LSP1, PTGDR, SELPLG, SPI1	8
Cellular Movement	invasion	invasion of proerythroblasts	7.48E-03	SPI1	1
Cellular Movement	invasion	invasion of synovial cells	7.48E-03	OSM	1
Cellular Movement	mobility	mobility of B lymphocytes	7.48E-03	IL10	1
Post-Translational Modification	modification	modification of protein	2.68E-05	CD6, CD22, CD37, CD48, CD79B, DUSP2, IL10, ITGAM, NR1I2, OSM, OXT, PADI4, PTPN7, PTPRO, RFFL, SPI1, SPN, STAT5A, TNK1	19
Post-Translational Modification	modification	modification of amino acids	4.87E-03	CAMKK1, CD6, DUSP2, FUT7, IL10, NR1I2, OSM, PRKAR1B, PTPN7, PTPRO, STAT5A	11
Post-Translational Modification	moiety attachment	moiety attachment of protein	2.74E-05	CD6, CD22, CD37, CD48, CD79B, IL10, ITGAM, OSM, OXT, PTPRO, RFFL, SPI1, SPN, STAT5A, TNK1	15
Post-Translational Modification	moiety attachment	moiety attachment of aromatic amino acids	6.56E-04	CD6, IL10, NR1I2, OSM, STAT5A	5
Post-Translational Modification	phosphorylation	phosphorylation of protein	2.98E-05	CD6, CD48, CD79B, IL10, ITGAM, OSM, OXT, PTPRO, SPI1, SPN, STAT5A, TNK1	12
Post-Translational Modification	phosphorylation	phosphorylation of L-tyrosine	3.16E-03	CD6, IL10, OSM, STAT5A	4
Post-Translational Modification	tyrosine phosphorylation	tyrosine phosphorylation of protein	7.51E-04	CD48, CD79B, ITGAM, OSM, OXT, SPI1, SPN	7
Respiratory Disease	severe acute respiratory syndrome	severe acute respiratory syndrome	3.18E-05	CEBPE, CTSZ (includes EG:1522), ITGAM, NCF1, NFE2, PADI4, PGLYRP1	7
Respiratory Disease	disease	disease of lung	2.29E-03	ADA, IL10, LYZ, SOD3, TLR9	5
Respiratory Disease	respiratory disorder	respiratory disorder	2.54E-03	CEBPE, CTSZ (includes EG:1522), IL10, ITGAM, LYZ, NCF1, NFE2, PADI4, PGLYRP1, PTGDR, S100A4	11
Respiratory Disease	cell movement	cell movement of lung cancer cell lines	2.92E-03	FUT7, S100A4	2
Respiratory Disease	infection	infection of lung	4.76E-03	LYZ, TLR9	2
Respiratory Disease	organismal abnormalities	organismal abnormalities of lung	8.05E-03	ADA, IL10, SOD3	3
DNA Replication, Recombination, and Repair	quantity	quantity of adenosine	3.31E-05	ADA, CD37, PTGDR	3
DNA Replication, Recombination, and Repair	quantity	quantity of centrosome	1.27E-02	HOXA7, RASSF1	2
DNA Replication, Recombination, and Repair	degradation	degradation of adenosine	7.48E-03	ADA	1
DNA Replication, Recombination, and Repair	demethylation	demethylation of genomic	7.48E-03	DNMT1	1
DNA Replication, Recombination, and Repair	reduction	reduction of adenosine	7.48E-03	ADA	1
DNA Replication, Recombination, and Repair	replication	replication of chromosomes	7.48E-03	DNMT1	1
Molecular Transport	quantity	quantity of adenosine	3.31E-05	ADA, CD37, PTGDR	3
Molecular Transport	quantity	quantity of eicosanoid	1.95E-03	IL10, IL18BP, IL1R2, LTC4S	4
Molecular Transport	quantity	quantity of prostaglandin E2	2.43E-03	IL10, IL18BP, IL1R2	3
Molecular Transport	quantity	quantity of Ca2+	6.07E-03	CD22, CD52, LYZ, OXT, S1PR4, SPN, VAV1	7
Molecular Transport	quantity	quantity of calcium	1.36E-02	CD22, ITGAM, LAT, LYZ, OXT, S100A4, SPN, VAV1	8
Molecular Transport	mobilization	mobilization of Ca2+	8.17E-04	CD22, IL10, LAT, OXT, PTGDR, VAV1	6
Molecular Transport	accumulation	accumulation of dATP	7.48E-03	ADA	1
Molecular Transport	accumulation	accumulation of calcium	1.49E-02	ADA, LAT	2
Molecular Transport	clearance	clearance of acetaminophen	7.48E-03	POR	1
Nucleic Acid Metabolism	quantity	quantity of adenosine	3.31E-05	ADA, CD37, PTGDR	3
Nucleic Acid Metabolism	metabolism	metabolism of S-adenosylmethionine	1.51E-03	DNMT1, GAMT	2
Nucleic Acid Metabolism	metabolism	metabolism of pentobarbital	7.48E-03	POR	1
Nucleic Acid Metabolism	accumulation	accumulation of dATP	7.48E-03	ADA	1
Nucleic Acid Metabolism	degradation	degradation of adenosine	7.48E-03	ADA	1
Nucleic Acid Metabolism	inactivation	inactivation of adenosine	7.48E-03	ADA	1
Nucleic Acid Metabolism	modification	modification of adenine	7.48E-03	DNMT1	1
Nucleic Acid Metabolism	reduction	reduction of adenosine	7.48E-03	ADA	1
Nucleic Acid Metabolism	removal	removal of adenosine	7.48E-03	ADA	1
Small Molecule Biochemistry	quantity	quantity of adenosine	3.31E-05	ADA, CD37, PTGDR	3
Small Molecule Biochemistry	quantity	quantity of eicosanoid	1.95E-03	IL10, IL18BP, IL1R2, LTC4S	4
Small Molecule Biochemistry	quantity	quantity of prostaglandin E2	2.43E-03	IL10, IL18BP, IL1R2	3
Small Molecule Biochemistry	moiety attachment	moiety attachment of aromatic amino acids	6.56E-04	CD6, IL10, NR1I2, OSM, STAT5A	5
Small Molecule Biochemistry	metabolism	metabolism of S-adenosylmethionine	1.51E-03	DNMT1, GAMT	2
Small Molecule Biochemistry	metabolism	metabolism of pentobarbital	7.48E-03	POR	1
Small Molecule Biochemistry	metabolism	metabolism of steroid	1.27E-02	NR1I2, STAT5A	2

¬© 2000-2009 Ingenuity Systems, Inc. All rights reserved.

Category	Biological Function	Function Annotation	P-Value	Molecules	# Molecules
Small Molecule Biochemistry	phosphorylation	phosphorylation of L-tyrosine	3.16E-03	CD6, IL10, OSM, STAT5A	4
Small Molecule Biochemistry	modification	modification of amino acids	4.87E-03	CAMKK1, CD6, DUSP2, FUT7, IL10, NR1I2, OSM, PRKAR1B, PTPN7, PTPRO, STAT5A	11
Small Molecule Biochemistry	modification	modification of adenine	7.48E-03	DNMT1	1
Small Molecule Biochemistry	binding	binding of hyaluronic acid	6.21E-03	IL10, OSM	2
Small Molecule Biochemistry	accumulation	accumulation of dATP	7.48E-03	ADA	1
Small Molecule Biochemistry	biosynthesis	biosynthesis of leukotriene A4	7.48E-03	LTC4S	1
Small Molecule Biochemistry	biosynthesis	biosynthesis of leukotriene C4	7.48E-03	LTC4S	1
Small Molecule Biochemistry	clearance	clearance of acetaminophen	7.48E-03	POR	1
Small Molecule Biochemistry	clearance	clearance of corticosterone	7.48E-03	NR1I2	1
Small Molecule Biochemistry	conjugation	conjugation of 5-oxo-6-8-11-14-(e,z,z,z)-eicosatetraenoic acid	7.48E-03	LTC4S	1
Small Molecule Biochemistry	degradation	degradation of adenosine	7.48E-03	ADA	1
Small Molecule Biochemistry	exhalation	exhalation of nitric oxide	7.48E-03	IL10	1
Small Molecule Biochemistry	inactivation	inactivation of adenosine	7.48E-03	ADA	1
Small Molecule Biochemistry	production	production of 6-trans-leukotriene B4	7.48E-03	LTC4S	1
Small Molecule Biochemistry	production	production of eicosanoid	7.93E-03	IL10, IL1R2, LTC4S, STAT5A	4
Small Molecule Biochemistry	production	production of leukotriene	1.37E-02	LTC4S, STAT5A	2
Small Molecule Biochemistry	reduction	reduction of adenosine	7.48E-03	ADA	1
Small Molecule Biochemistry	reduction	reduction of nitrite	7.48E-03	POR	1
Small Molecule Biochemistry	removal	removal of adenosine	7.48E-03	ADA	1
Small Molecule Biochemistry	metabolic process	metabolic process of eicosanoid	8.46E-03	ALOX15B, LTC4S, NCF1	3
Small Molecule Biochemistry	generation	generation of inositol phosphate	8.72E-03	CD22, LAT	2
Small Molecule Biochemistry	synthesis	synthesis of leukotriene	8.72E-03	ADA, LTC4S	2
Free Radical Scavenging	production	production of superoxide	3.35E-05	CEBPE, IL10, ITGAM, NCF1, NOX4, SPI1	6
Free Radical Scavenging	production	production of reactive oxygen species	1.26E-04	CD52, IL10, LAT, NCF1, NOX4, NR1I2, SELPLG	7
Tissue Development	adhesion	adhesion of eukaryotic cells	3.83E-05	ALOX15B, CD48, COL7A1, FUT7, ICAM3, IL10, ITGAM, LAT, OSM, RASSF1, SELPLG, SPI1, SPN, VAV1	14
Tissue Development	adhesion	adhesion of cells	4.34E-05	ALOX15B, CD48, CD93, CLEC4A, COL7A1, FUT7, ICAM3, IL10, ITGAM, LAT, LGALS7, OSM, RASSF1, SELPLG, SPI1, SPN, STAT5A, VAV1	18
Tissue Development	adhesion	adhesion of antigen presenting cells	7.96E-05	CD48, ICAM3, ITGAM, SPI1	4
Tissue Development	adhesion	adhesion of Th1 cells	8.15E-04	FUT7, SELPLG	2
Tissue Development	adhesion	adhesion of lymphocytes	9.97E-04	CD48, FUT7, OSM, SELPLG, SPN	5
Tissue Development	adhesion	adhesion of dendritic cells	1.93E-03	ICAM3, ITGAM	2
Tissue Development	adhesion	adhesion of granulocytes	2.51E-03	ICAM3, ITGAM, SELPLG, VAV1	4
Tissue Development	adhesion	adhesion of T lymphocytes	2.76E-03	CD48, FUT7, SELPLG, SPN	4
Tissue Development	adhesion	adhesion of endothelial tissue	5.46E-03	ITGAM, VAV1	2
Tissue Development	adhesion	adhesion of neutrophils	6.53E-03	ITGAM, SELPLG, VAV1	3
Tissue Development	adhesion	adhesion of eosinophils	1.37E-02	ITGAM, SELPLG	2
Tissue Development	aggregation	aggregation of granulocytes	6.42E-05	ICAM3, ITGAM, SELPLG	3
Tissue Development	aggregation	aggregation of leukocytes	3.19E-04	ICAM3, ITGAM, SELPLG, SPN	4
Tissue Development	aggregation	aggregation of neutrophils	1.51E-03	ITGAM, SELPLG	2
Tissue Development	aggregation	aggregation of blood cells	4.91E-03	ICAM3, ITGAM, SELPLG, SPN, VAV1	5
Tissue Development	aggregation	aggregation of T lymphocytes	7.00E-03	ICAM3, SPN	2
Tissue Development	generation	generation of Tr1 cells	3.29E-04	IL10, IL27	2
Tissue Development	generation	generation of naive T lymphocytes	8.15E-04	IL10, IL27	2
Tissue Development	generation	generation of dendritic cells	6.21E-03	IL10, SPI1	2
Tissue Development	function	function of epithelial barrier	7.48E-03	IL10	1
Cell Signaling	Ras protein signal transduction	Ras protein signal transduction	5.02E-05	DOK2, DOK3, LAT, RASSF1, TNK1	5
Cell Signaling	tyrosine phosphorylation	tyrosine phosphorylation of protein	7.51E-04	CD48, CD79B, ITGAM, OSM, OXT, SPI1, SPN	7
Cell Signaling	mobilization	mobilization of Ca2+	8.17E-04	CD22, IL10, LAT, OXT, PTGDR, VAV1	6
Cell Signaling	cell surface receptor linked signal transduction	cell surface receptor linked signal transduction	2.79E-03	CD22, CLEC4A, DOK2, KLRG1, LAT, SPN, STAT5A, VAV1	8
Cell Signaling	quantity	quantity of Ca2+	6.07E-03	CD22, CD52, LYZ, OXT, S1PR4, SPN, VAV1	7
Cell Signaling	quantity	quantity of calcium	1.36E-02	CD22, ITGAM, LAT, LYZ, OXT, S100A4, SPN, VAV1	8
Cell Signaling	exhalation	exhalation of nitric oxide	7.48E-03	IL10	1
Cell Signaling	accumulation	accumulation of calcium	1.49E-02	ADA, LAT	2
Genetic Disorder	epidermodysplasia verruciformis	epidermodysplasia verruciformis	5.54E-05	TMC6, TMC8	2
Genetic Disorder	familial cold autoinflammatory syndrome	familial cold autoinflammatory syndrome	5.46E-04	NLRP3, NLRP12	2
Genetic Disorder	atopic dermatitis	atopic dermatitis	6.89E-04	DUSP2, FUT7, OSM, SPI1, TNFSF8	5
Genetic Disorder	systemic anaphylaxis	systemic anaphylaxis of mice	1.14E-03	LAT, VAV1	2
Genetic Disorder	hairy-cell leukemia	hairy-cell leukemia	2.92E-03	ADA, CD22	2
Genetic Disorder	chronic lymphocytic leukemia	chronic lymphocytic leukemia	4.09E-03	ADA, CD22, CD52, TLR9	4
Genetic Disorder	autoimmune disease	autoimmune disease	5.32E-03	AFF3, AIF1, ATP10A, CD6, CD22, CD52, CLEC4A, COL7A1, CRYBB1, DUSP2, ICAM3, IL10, IL27, IL18BP, IL1R2, ITGAM, KLRG1, LMO2, LYZ, ME1, NCF1, NCF4, OSM, PADI4, PGLYRP1, PTPRO, SH3TC1, TLR9, TRAF1	29
Genetic Disorder	systemic lupus erythematosus	systemic lupus erythematosus	6.46E-03	CD22, IL10, ITGAM, TLR9	4
Genetic Disorder	adenosine deaminase deficiency	adenosine deaminase deficiency	7.48E-03	ADA	1
Genetic Disorder	leukocyte adhesion deficiency	leukocyte adhesion deficiency of mice	7.48E-03	FUT7	1
Genetic Disorder	long QT syndrome 5	long QT syndrome 5	7.48E-03	KCNE1	1
Genetic Disorder	rheumatoid arthritis	onset of rheumatoid arthritis of mice	7.48E-03	IL10	1

				¬© 2000-2009 Ingenuity Systems, Inc. All rights reserved.	
**Category**	**Biological Function**	**Function Annotation**	**P-Value**	**Molecules**	**# Molecules**
Genetic Disorder	specific granule deficiency	specific granule deficiency	7.48E-03	CEBPE	1
Genetic Disorder	anaphylaxis	anaphylaxis	7.65E-03	LAT, LTC4S, VAV1	3
Genetic Disorder	Jervell and Lange-Nielsen syndrome	Jervell and Lange-Nielsen syndrome	1.49E-02	KCNE1	1
Cell Morphology	polarization	polarization of leukocytes	1.06E-04	IL10, IL27, LSP1, SPN, VAV1	5
Cell Morphology	polarization	polarization of T lymphocytes	3.29E-03	IL27, SPN, VAV1	3
Cell Morphology	morphology	morphology of cancer cells	1.14E-03	HPN, LSP1	2
Cell Morphology	morphology	morphology of hepatocytes	1.14E-03	ADA, OSM	2
Cell Morphology	morphology	morphology of eukaryotic cells	1.18E-03	ADA, AIF1, CEBPE, HOXA7, HPN, IL10, LSP1, NFE2, OSM, RASSF1	10
Cell Morphology	morphology	morphology of normal cells	1.31E-03	ADA, AIF1, CEBPE, HOXA7, IL10, NFE2, OSM	7
Cell Morphology	morphology	morphology of liver cancer cells	7.48E-03	HPN	1
Cell Morphology	morphology	morphology of blood cells	7.65E-03	CEBPE, IL10, NFE2	3
Cell Morphology	conversion	conversion of dendritic cells	7.48E-03	IL10	1
Inflammatory Disease	inflammatory disorder	inflammatory disorder of mice	1.29E-04	CD22, DOK2, GPR77, IL10, IL18BP, IL1R2, ITGAM, LTC4S, NCF1, STAT5A, VAV1	11
Inflammatory Disease	inflammatory disorder	inflammatory disorder	9.06E-04	ADA, AFF3, AIF1, ATP10A, CD6, CD22, CD48, CD52, CD79B, CLEC4A, DOK2, DUSP2, FUT7, GPR77, ICAM3, IL10, IL18BP, IL1R2, ITGAM, LMO2, LTC4S, LYZ, MPHOSPH9, NCF1, NCF4, NLRP3, NLRP12, ONECUT2, OSM, PADI4, PGLYRP1, PTGDR, S100A4, SPI1, STAT5A, TLR9, TNFSF8, TRAF1, VAV1	39
Inflammatory Disease	dermatitis	dermatitis	1.74E-04	CD52, DUSP2, FUT7, IL18BP, OSM, SPI1, TNFSF8	7
Inflammatory Disease	familial cold autoinflammatory syndrome	familial cold autoinflammatory syndrome	5.46E-04	NLRP3, NLRP12	2
Inflammatory Disease	atopic dermatitis	atopic dermatitis	6.89E-04	DUSP2, FUT7, OSM, SPI1, TNFSF8	5
Inflammatory Disease	systemic lupus erythematosus	systemic lupus erythematosus	6.46E-03	CD22, IL10, ITGAM, TLR9	4
Inflammatory Disease	systemic lupus erythematosus	systemic lupus erythematosus of animal	1.49E-02	CD22, TLR9	2
Inflammatory Disease	airway hyperresponsiveness	airway hyperresponsiveness of mice	7.26E-03	GPR77, IL10, LTC4S	3
Inflammatory Disease	lyme arthritis	lyme arthritis	7.48E-03	IL10	1
Inflammatory Disease	rheumatoid arthritis	onset of rheumatoid arthritis of mice	7.48E-03	IL10	1
Inflammatory Disease	splenomegaly	splenomegaly of mice	9.31E-03	DOK2, STAT5A, VAV1	3
Inflammatory Disease	rheumatic disease	rheumatic disease	1.14E-02	AFF3, AIF1, ATP10A, CD6, CD22, CD79B, CLEC4A, DUSP2, ICAM3, IL10, IL18BP, IL1R2, ITGAM, LMO2, LYZ, NCF1, NCF4, NLRP3, OSM, PADI4, PGLYRP1, TLR9, TRAF1	23
Inflammatory Disease	rheumatic disease	rheumatic disease of mice	1.44E-02	CD22, IL10, IL1R2, NCF1	4
Gene Expression	transactivation	transactivation of GATA-1 binding site	1.65E-04	CEBPE, SPI1	2
Gene Expression	transactivation	transactivation of Pu.1 binding site	5.46E-04	CEBPE, SPI1	2
Gene Expression	transactivation	transactivation	7.01E-04	CD6, CEBPE, LMO2, NFE2, NR1I2, OSM, PYCARD, S100A4, SELPLG, SPI1, STAT5A, TLR9, VAV1	13
Gene Expression	transactivation	transactivation of C/EBP binding site	8.15E-04	CEBPE, SPI1	2
Gene Expression	transactivation	transactivation of ER6 pregnane X response element	7.48E-03	NR1I2	1
Gene Expression	transactivation	transactivation of Ets2 binding site	7.48E-03	SPI1	1
Gene Expression	transactivation	transactivation of TATA like element	7.48E-03	OSM	1
Gene Expression	transactivation	transactivation of repeat 3 element	7.48E-03	OSM	1
Gene Expression	activation	activation of NF-E2 binding site	5.46E-04	NFE2, SPI1	2
Gene Expression	binding	binding of Foxa2 binding site	7.48E-03	NR1I2	1
Gene Expression	binding	binding of G1 proglucagon element	7.48E-03	KLRG1	1
Gene Expression	binding	binding of protein binding site	1.37E-02	SPN, VAV1	2
Gene Expression	demethylation	demethylation of genomic	7.48E-03	DNMT1	1
Gene Expression	expression	expression of PE21 element	7.48E-03	OSM	1
Neurological Disease	damage	damage of spinal cord	1.65E-04	IL10, PYCARD	2
Neurological Disease	paralysis	paralysis of mice	4.76E-03	IL10, NR1I2	2
Neurological Disease	cannabinoid withdrawal syndrome	cannabinoid withdrawal syndrome of rats	7.48E-03	OXT	1
Neurological Disease	colony survival	colony survival of glioblastoma multiforme	7.48E-03	IL10	1
Neurological Disease	Jervell and Lange-Nielsen syndrome	Jervell and Lange-Nielsen syndrome	1.49E-02	KCNE1	1
Hypersensitivity Response	infiltration	infiltration of eosinophils	2.66E-04	CD48, IL10, LTC4S, OSM	4
Hypersensitivity Response	cell movement	cell movement of eosinophils	3.28E-04	CD48, IL10, ITGAM, LTC4S, OSM	5
Hypersensitivity Response	activation	activation of mast cells	2.43E-03	CD48, IL10, ITGAM	3
Hypersensitivity Response	cytotoxic reaction	cytotoxic reaction of eosinophils	7.48E-03	ITGAM	1
Hypersensitivity Response	adhesion	adhesion of eosinophils	1.37E-02	ITGAM, SELPLG	2
Hematological Disease	apoptosis	apoptosis of plasmacytoid dendritic cells	3.29E-04	IL10, TLR9	2
Hematological Disease	apoptosis	apoptosis of leukocytes	5.48E-03	ADA, CD22, IL10, ITGAM, SPN, STAT5A, TLR9, TRAF1	8
Hematological Disease	apoptosis	apoptosis of myeloid dendritic cells	7.48E-03	IL10	1
Hematological Disease	apoptosis	apoptosis of peripheral blood monocytes	7.48E-03	IL10	1
Hematological Disease	apoptosis	apoptosis of lymphocytes	1.41E-02	ADA, CD22, IL10, SPN, STAT5A, TRAF1	6
Hematological Disease	cell rolling	cell rolling of leukemia cell lines	5.46E-04	FUT7, SELPLG	2
Hematological Disease	binding	binding of leukemia cell lines	7.52E-04	FUT7, ITGAM, SELPLG, SPN	4
Hematological Disease	cell death	cell death of leukocytes	9.14E-04	ADA, CD22, IL10, ITGAM, LAT, PYCARD, SPN, STAT5A, TLR9, TRAF1	10
Hematological Disease	cell death	cell death of lymphocytes	7.52E-03	ADA, CD22, IL10, LAT, SPN, STAT5A, TRAF1	7
Hematological Disease	cell death	cell death of T lymphocytes	8.90E-03	ADA, IL10, LAT, SPN, STAT5A, TRAF1	6
Hematological Disease	parasitemia	parasitemia of mice	1.93E-03	IL10, TLR9	2
Hematological Disease	leukemia	leukemia	2.39E-03	ADA, CD22, CD52, DOK2, LMO2, POR, SPI1, TLR9	8
Hematological Disease	leukemia	leukemia of mammalia	9.76E-03	DOK2, LMO2, SPI1	3

				¬© 2000-2009 Ingenuity Systems, Inc. All rights reserved.	
**Category**	**Biological Function**	**Function Annotation**	**P-Value**	**Molecules**	**# Molecules**
Hematological Disease	hyperproliferation	hyperproliferation of hematopoietic progenitor cells	2.40E-03	DOK2, IL10	2
Hematological Disease	hairy-cell leukemia	hairy-cell leukemia	2.92E-03	ADA, CD22	2
Hematological Disease	chronic lymphocytic leukemia	chronic lymphocytic leukemia	4.09E-03	ADA, CD22, CD52, TLR9	4
Hematological Disease	infectious disorder	infectious disorder of antigen presenting cells	6.21E-03	ITGAM, TLR9	2
Hematological Disease	myeloproliferative syndrome	myeloproliferative syndrome of mice	6.21E-03	DOK2, SPI1	2
Hematological Disease	disease	disease of leukemia cell lines	7.00E-03	NLRP3, SELPLG	2
Hematological Disease	anemia	anemia of humans	7.48E-03	IL10	1
Hematological Disease	dedifferentiation	dedifferentiation of erythroleukemia cells	7.48E-03	SPI1	1
Hematological Disease	depletion	depletion of plasmacytoid dendritic cells	7.48E-03	BST2	1
Hematological Disease	edema	edema of leukemia cell lines	7.48E-03	NLRP3	1
Hematological Disease	invasion	invasion of proerythroblasts	7.48E-03	SPI1	1
Hematological Disease	leukopenia	leukopenia of humans	7.48E-03	IL10	1
Hematological Disease	oxidative stress response	oxidative stress response of leukemia cell lines	7.48E-03	VAV1	1
Hematological Disease	lymphocytic leukemia	lymphocytic leukemia	7.55E-03	ADA, CD22, CD52, POR, TLR9	5
Hematological Disease	adhesion	adhesion of leukemia cell lines	7.65E-03	ITGAM, LAT, SELPLG	3
Infection Mechanism	production	production of HIV-1	6.46E-04	IL10, SPN, STAT5A	3
Amino Acid Metabolism	moiety attachment	moiety attachment of aromatic amino acids	6.56E-04	CD6, IL10, NR1I2, OSM, STAT5A	5
Amino Acid Metabolism	metabolism	metabolism of S-adenosylmethionine	1.51E-03	DNMT1, GAMT	2
Amino Acid Metabolism	phosphorylation	phosphorylation of L-tyrosine	3.16E-03	CD6, IL10, OSM, STAT5A	4
Amino Acid Metabolism	modification	modification of amino acids	4.87E-03	CAMKK1, CD6, DUSP2, FUT7, IL10, NR1I2, OSM, PRKAR1B, PTPN7, PTPRO, STAT5A	11
Vitamin and Mineral Metabolism	mobilization	mobilization of Ca2+	8.17E-04	CD22, IL10, LAT, OXT, PTGDR, VAV1	6
Vitamin and Mineral Metabolism	quantity	quantity of Ca2+	6.07E-03	CD22, CD52, LYZ, OXT, S1PR4, SPN, VAV1	7
Vitamin and Mineral Metabolism	quantity	quantity of calcium	1.36E-02	CD22, ITGAM, LAT, LYZ, OXT, S100A4, SPN, VAV1	8
Vitamin and Mineral Metabolism	accumulation	accumulation of calcium	1.49E-02	ADA, LAT	2
Hepatic System Development and Function	morphology	morphology of hepatocytes	1.14E-03	ADA, OSM	2
Tumor Morphology	morphology	morphology of cancer cells	1.14E-03	HPN, LSP1	2
Tumor Morphology	morphology	morphology of liver cancer cells	7.48E-03	HPN	1
Tumor Morphology	metaplasia	metaplasia of mucus	2.40E-03	ADA, IL10	2
Tumor Morphology	clearance	clearance of tumor	7.48E-03	VAV1	1
Tumor Morphology	colony survival	colony survival of adenocarcinoma cells	7.48E-03	IL10	1
Tumor Morphology	development	development of pulmonary adenoma	7.48E-03	RASSF1	1
Tumor Morphology	differentiation	differentiation of teratoma	7.48E-03	DNMT1	1
Tumor Morphology	growth	growth of prostatic carcinoma	7.48E-03	DAB2IP	1
Tumor Morphology	necrosis	necrosis of tumor tissue	7.48E-03	NUAK1	1
Tumor Morphology	volume	volume of metastatic tumor	7.48E-03	IL18BP	1
Cell Cycle	cell cycle progression	cell cycle progression of eukaryotic cells	1.26E-03	AIF1, ALOX15B, OSM, PADI4, PTPRO, RASSF1, S100A4, STAT5A, VAV1	9
Cell Cycle	cell cycle progression	cell cycle progression of tumor cell lines	6.29E-03	PADI4, PTPRO, RASSF1, S100A4, VAV1	5
Cell Cycle	cell cycle progression	arrest in cell cycle progression of pancreatic cancer cell lines	7.48E-03	S100A4	1
Cell Cycle	cell cycle progression	cell cycle progression of cell lines	1.06E-02	AIF1, PADI4, PTPRO, RASSF1, S100A4, VAV1	6
Cell Cycle	cell cycle progression	cell cycle progression of blood cells	1.38E-02	OSM, STAT5A, VAV1	3
Cell Cycle	cell division process	cell division process of cells	1.78E-03	AIF1, ALOX15B, CAMKK1, CD37, DNMT1, HOXA7, IL10, LTC4S, MPHOSPH9, OSM, PADI4, PTPRO, RASSF1, RFFL, S100A4, SPI1, STAT5A, VAV1	18
Cell Cycle	cell division process	cell division process of eukaryotic cells	6.09E-03	AIF1, ALOX15B, CAMKK1, CD37, HOXA7, LTC4S, OSM, PADI4, PTPRO, RASSF1, RFFL, S100A4, STAT5A, VAV1	14
Cell Cycle	cell division process	cell division process of T lymphocytes	6.89E-03	CD37, STAT5A, VAV1	3
Cell Cycle	cell division process	cell division process of blood cells	1.29E-02	CD37, OSM, STAT5A, VAV1	4
Cell Cycle	cell division	entry into cell division of T lymphocytes	7.48E-03	CD37	1
Cell Cycle	mitosis	delay in initiation of mitosis of fibroblast cell lines	7.48E-03	RASSF1	1
Cell Cycle	mitosis	arrest in mitosis of eukaryotic cells	1.27E-02	HOXA7, RASSF1	2
Cell Cycle	G1 phase	arrest in G1 phase of breast cancer cell lines	1.27E-02	CAMKK1, RASSF1	2
Nervous System Development and Function	action potential	action potential of normal cells	1.93E-03	KCNE1, OXT	2
Nervous System Development and Function	sleep	sleep of mice	3.48E-03	NR1I2, PTGDR	2
Nervous System Development and Function	long term depression	long term depression of dentate granule cells	7.48E-03	PRKAR1B	1
Nervous System Development and Function	quantity	quantity of dorsal horn cells	7.48E-03	PRKAR1B	1
Nervous System Development and Function	memory	memory of mice	1.07E-02	NCF1, OXT, SOD3	3
Skeletal and Muscular System Development and Function	proliferation	proliferation of PASMC cells	1.93E-03	IL10, NOX4	2
Skeletal and Muscular System Development and Function	proliferation	proliferation of smooth muscle cells	4.77E-03	AIF1, IL10, NCF1, NOX4, OXT	5
Skeletal and Muscular System Development and Function	proliferation	proliferation of myoepithelial cells	7.48E-03	OXT	1
Skeletal and Muscular System Development and Function	differentiation	differentiation of myoepithelial cells	7.48E-03	OXT	1
Skeletal and Muscular System Development and Function	morphology	morphology of synovial membrane	7.48E-03	OSM	1
Skeletal and Muscular System Development and Function	stimulation	stimulation of nasal cartilage	7.48E-03	OSM	1
Lipid Metabolism	quantity	quantity of eicosanoid	1.95E-03	IL10, IL18BP, IL1R2, LTC4S	4

				¬© 2000-2009 Ingenuity Systems, Inc. All rights reserved.	
**Category**	**Biological Function**	**Function Annotation**	**P-Value**	**Molecules**	**# Molecules**
Lipid Metabolism	quantity	quantity of prostaglandin E2	2.43E-03	IL10, IL18BP, IL1R2	3
Lipid Metabolism	biosynthesis	biosynthesis of leukotriene A4	7.48E-03	LTC4S	1
Lipid Metabolism	biosynthesis	biosynthesis of leukotriene C4	7.48E-03	LTC4S	1
Lipid Metabolism	clearance	clearance of corticosterone	7.48E-03	NR1I2	1
Lipid Metabolism	conjugation	conjugation of 5-oxo-6-8-11-14-(e,z,z,z)-eicosatetraenoic acid	7.48E-03	LTC4S	1
Lipid Metabolism	production	production of 6-trans-leukotriene B4	7.48E-03	LTC4S	1
Lipid Metabolism	production	production of eicosanoid	7.93E-03	IL10, IL1R2, LTC4S, STAT5A	4
Lipid Metabolism	production	production of leukotriene	1.37E-02	LTC4S, STAT5A	2
Lipid Metabolism	metabolic process	metabolic process of eicosanoid	8.46E-03	ALOX15B, LTC4S, NCF1	3
Lipid Metabolism	synthesis	synthesis of leukotriene	8.72E-03	ADA, LTC4S	2
Lipid Metabolism	metabolism	metabolism of steroid	1.27E-02	NR1I2, STAT5A	2
Organismal Development	developmental process	developmental process of organism	2.06E-03	ADA, AFF3, AIF1, ALOX15B, BST2, DNMT1, LMO2, MGAT1, NFE2, OSM, POR, PTGDR, SMOC2, SPI1, STAT5A, TCP11, VAV1	17
Organismal Development	development	development of organism	4.90E-03	ADA, AFF3, BST2, DNMT1, LMO2, MGAT1, NFE2, OSM, POR, SMOC2, SPI1, STAT5A, TCP11, VAV1	14
Organismal Development	development	development of mice	1.10E-02	DNMT1, MGAT1, OSM, POR, SMOC2, STAT5A, VAV1	7
Organismal Development	response	response of mice	6.89E-03	CD22, IL10, TLR9	3
Organismal Development	volume	volume of airway surface liquid	7.48E-03	ADA	1
Developmental Disorder	metaplasia	metaplasia of mucus	2.40E-03	ADA, IL10	2
Drug Metabolism	quantity	quantity of prostaglandin E2	2.43E-03	IL10, IL18BP, IL1R2	3
Drug Metabolism	binding	binding of hyaluronic acid	6.21E-03	IL10, OSM	2
Drug Metabolism	clearance	clearance of acetaminophen	7.48E-03	POR	1
Drug Metabolism	clearance	clearance of cyclophosphamide	7.48E-03	POR	1
Drug Metabolism	half-life	half-life of cyclophosphamide	7.48E-03	POR	1
Cellular Compromise	degranulation	degranulation of cells	2.45E-03	GPR77, ITGAM, LAT, STAT5A, VAV1	5
Cellular Compromise	degranulation	degranulation of eukaryotic cells	1.32E-02	ITGAM, LAT, STAT5A, VAV1	4
Cellular Compromise	destabilization	destabilization of myosin filaments	7.48E-03	S100A4	1
Cellular Compromise	oxidative stress response	oxidative stress response of leukemia cell lines	7.48E-03	VAV1	1
Organismal Survival	survival	survival of mice	2.49E-03	CD6, DNMT1, DOK2, IL10, ITGAM, MGAT1, NCF1, STAT5A, TLR9	9
Reproductive System Development and Function	binding	binding of gonadal cell lines	4.04E-03	CD48, FUT7, ITGAM	3
Reproductive System Development and Function	contraction	contraction of uterine tissue	7.48E-03	OXT	1
Reproductive System Development and Function	survival	survival of Sertoli cells	7.48E-03	OSM	1
Reproductive System Development and Function	survival	survival of primordial germ cells	7.48E-03	OSM	1
Cardiovascular System Development and Function	angiogenesis	angiogenesis of endothelial cell lines	4.10E-03	DGKA, SMOC2	2
Cardiovascular System Development and Function	angiogenesis	angiogenesis of eye	7.48E-03	IL18BP	1
Cardiovascular System Development and Function	angiogenesis	angiogenesis of organ	1.49E-02	ADA, IL18BP	2
Cardiovascular System Development and Function	adhesion	adhesion of endothelial tissue	5.46E-03	ITGAM, VAV1	2
Cardiovascular System Development and Function	mass	mass of right ventricle of heart	7.48E-03	IL10	1
Cardiovascular System Development and Function	permeability	permeability of vascular tissue	1.06E-02	IL18BP, LSP1	2
Lymphoid Tissue Structure and Development	development	development of helper T lymphocytes	4.89E-03	IL10, IL27, STAT5A	3
Lymphoid Tissue Structure and Development	lymphopoiesis	lymphopoiesis of lymphoid cells	7.48E-03	SPI1	1
Lymphoid Tissue Structure and Development	morphology	morphology of germinal center	7.48E-03	ADA	1
Lymphoid Tissue Structure and Development	quantity	quantity of lymphatic system cells	1.31E-02	ADA, IL10, IL18BP, PTPRCAP, SELPLG	5
Carbohydrate Metabolism	binding	binding of hyaluronic acid	6.21E-03	IL10, OSM	2
Carbohydrate Metabolism	generation	generation of inositol phosphate	8.72E-03	CD22, LAT	2
Connective Tissue Disorders	systemic lupus erythematosus	systemic lupus erythematosus	6.46E-03	CD22, IL10, ITGAM, TLR9	4
Connective Tissue Disorders	systemic lupus erythematosus	systemic lupus erythematosus of animal	1.49E-02	CD22, TLR9	2
Connective Tissue Disorders	invasion	invasion of synovial cells	7.48E-03	OSM	1
Connective Tissue Disorders	lyme arthritis	lyme arthritis	7.48E-03	IL10	1
Connective Tissue Disorders	rheumatoid arthritis	onset of rheumatoid arthritis of mice	7.48E-03	IL10	1
Connective Tissue Disorders	connective tissue disorder	connective tissue disorder	1.05E-02	AFF3, AIF1, ATP10A, CD6, CD22, CD79B, CLEC4A, COL7A1, DUSP2, ICAM3, IL10, IL18BP, IL1R2, ITGAM, LMO2, LYZ, NCF1, NCF4, NLRP3, OSM, PADI4, PGLYRP1, TLR9, TRAF1	24
Connective Tissue Disorders	rheumatic disease	rheumatic disease	1.14E-02	AFF3, AIF1, ATP10A, CD6, CD22, CD79B, CLEC4A, DUSP2, ICAM3, IL10, IL18BP, IL1R2, ITGAM, LMO2, LYZ, NCF1, NCF4, NLRP3, OSM, PADI4, PGLYRP1, TLR9, TRAF1	23
Connective Tissue Disorders	rheumatic disease	rheumatic disease of mice	1.44E-02	CD22, IL10, IL1R2, NCF1	4
Skeletal and Muscular Disorders	systemic lupus erythematosus	systemic lupus erythematosus	6.46E-03	CD22, IL10, ITGAM, TLR9	4
Skeletal and Muscular Disorders	systemic lupus erythematosus	systemic lupus erythematosus of animal	1.49E-02	CD22, TLR9	2
Skeletal and Muscular Disorders	cardiac fibrosis	cardiac fibrosis of myocardium	7.48E-03	NCF1	1
Skeletal and Muscular Disorders	destabilization	destabilization of myosin filaments	7.48E-03	S100A4	1
Skeletal and Muscular Disorders	lyme arthritis	lyme arthritis	7.48E-03	IL10	1
Skeletal and Muscular Disorders	rheumatoid arthritis	onset of rheumatoid arthritis of mice	7.48E-03	IL10	1
Skeletal and Muscular Disorders	rheumatic disease	rheumatic disease	1.14E-02	AFF3, AIF1, ATP10A, CD6, CD22, CD79B, CLEC4A, DUSP2, ICAM3, IL10, IL18BP, IL1R2, ITGAM, LMO2, LYZ, NCF1, NCF4, NLRP3, OSM, PADI4, PGLYRP1, TLR9, TRAF1	23
Skeletal and Muscular Disorders	rheumatic disease	rheumatic disease of mice	1.44E-02	CD22, IL10, IL1R2, NCF1	4
Organismal Functions	response	response of mice	6.89E-03	CD22, IL10, TLR9	3
Cardiovascular Disease	long QT syndrome 5	long QT syndrome 5	7.48E-03	KCNE1	1
Cardiovascular Disease	mass	mass of thrombus	7.48E-03	IL10	1
Cardiovascular Disease	Jervell and Lange-Nielsen syndrome	Jervell and Lange-Nielsen syndrome	1.49E-02	KCNE1	1
Cellular Assembly and Organization	exocytosis	exocytosis of cytotoxic granule	7.48E-03	VAV1	1
Cellular Assembly and Organization	production	production of milk fat globules	7.48E-03	STAT5A	1

				¬© 2000-2009 Ingenuity Systems, Inc. All rights reserved.	
**Category**	**Biological Function**	**Function Annotation**	**P-Value**	**Molecules**	**# Molecules**
Cellular Assembly and Organization	recruitment	recruitment of actin cytoskeleton	7.48E-03	VAV1	1
Cellular Assembly and Organization	recruitment	recruitment of membrane rafts	7.48E-03	CD48	1
Cellular Assembly and Organization	shape	shape of nuclear envelope	7.48E-03	ADA	1
Cellular Assembly and Organization	translocation	translocation of membrane rafts	7.48E-03	VAV1	1
Cellular Assembly and Organization	formation	formation of cellular protrusions	9.90E-03	ACAP1, PKP1, RASSF1, VAV1	4
Cellular Assembly and Organization	quantity	quantity of centrosome	1.27E-02	HOXA7, RASSF1	2
Cellular Assembly and Organization	organization	organization of cytoskeleton	1.40E-02	ICAM3, IL10, SPI1, VAV1	4
Connective Tissue Development and Function	mitosis	delay in initiation of mitosis of fibroblast cell lines	7.48E-03	RASSF1	1
Connective Tissue Development and Function	morphology	morphology of synovial membrane	7.48E-03	OSM	1
Connective Tissue Development and Function	stimulation	stimulation of nasal cartilage	7.48E-03	OSM	1
Connective Tissue Development and Function	survival	survival of Sertoli cells	7.48E-03	OSM	1
Connective Tissue Development and Function	proliferation	proliferation of synovial cells	7.84E-03	AIF1, OSM	2
Embryonic Development	disorganization	disorganization of hepatic plate	7.48E-03	ADA	1
Embryonic Development	response	response of embryonic cell lines	7.48E-03	TLR9	1
Endocrine System Disorders	cell cycle progression	arrest in cell cycle progression of pancreatic cancer cell lines	7.48E-03	S100A4	1
Hair and Skin Development and Function	function	function of epithelial barrier	7.48E-03	IL10	1
Hair and Skin Development and Function	response	response of epithelial cell lines	7.48E-03	TLR9	1
Hair and Skin Development and Function	structural integrity	structural integrity of epithelial barrier	7.48E-03	IL10	1
Hair and Skin Development and Function	re-epithelialization	re-epithelialization	1.06E-02	IL10, LGALS7	2
Organ Development	angiogenesis	angiogenesis of eye	7.48E-03	IL18BP	1
Organ Development	angiogenesis	angiogenesis of organ	1.49E-02	ADA, IL18BP	2
Organ Morphology	mass	mass of right ventricle of heart	7.48E-03	IL10	1
Psychological Disorders	cannabinoid withdrawal syndrome	cannabinoid withdrawal syndrome of rats	7.48E-03	OXT	1
Renal and Urological System Development and Function	response	response of kidney cell lines	7.48E-03	TLR9	1
Reproductive System Disease	differentiation	differentiation of teratoma	7.48E-03	DNMT1	1
Reproductive System Disease	growth	growth of prostatic carcinoma	7.48E-03	DAB2IP	1
Reproductive System Disease	progression	progression of cervical cancer cell lines	7.48E-03	RASSF1	1
Reproductive System Disease	survival	survival of cervical cancer cell lines	8.96E-03	DGKA, PPP1R16B, PTPN7, PTPRCAP, PTPRO	5
Reproductive System Disease	G1 phase	arrest in G1 phase of breast cancer cell lines	1.27E-02	CAMKK1, RASSF1	2
Reproductive System Disease	tumorigenesis	tumorigenesis of breast cancer cell lines	1.49E-02	DUSP2, S100A4	2
Visual System Development and Function	angiogenesis	angiogenesis of eye	7.48E-03	IL18BP	1
Protein Synthesis	synthesis	synthesis of protein	8.10E-03	CD22, CEBPE, IL10, IL27, NLRP12, SPN, STAT5A, TLR9	8
Protein Synthesis	biosynthesis	biosynthesis of protein	1.02E-02	CEBPE, IL10, IL27, NLRP12, SPN, STAT5A, TLR9	7
Auditory Disease	Jervell and Lange-Nielsen syndrome	Jervell and Lange-Nielsen syndrome	1.49E-02	KCNE1	1
