# Supplementary material for: Structural and Regulatory Characterization of the Placental Epigenome at Its Maternal Interface
Source: PLoS One. 2011 Feb 23;6(2):e14723. doi: 10.1371/journal.pone.0014723 (PMC3044138; doi:10.1371/journal.pone.0014723)
Supplement: Table S3 — IPA biological network analysis of genes hypomethylated in CVS versus MBC. (0.07 MB PDF) [file pone.0014723.s003.pdf]

Hypomethylated in CVS versus MBC **Table S3**

¬© 2000-2009 Ingenuity Systems, Inc. All rights reserved.

ID	Top Functions	Molecules in Network	Score	Focus Molecules
1	Gastrointestinal Disease, Genetic Disorder, Inflammatory Disease	AOC3,CFHR1,CRYBB2,DAK,DEFB4 (includes EG:1673),ELF3,ELF5,EMR1,FCGR3A,FGG,Fibrinogen,IFN Beta,IFNA8,IFNB1,IgG,IL1,IL12 (complex),IL1F5,Interferon alpha,KRT8,KRTAP11-1,LBP,LILRB3 (includes EG:11025),NCR1,NFkB (complex),PDCD1,PIGR,PLAT,PRSS8,S100P,SERPINB5,ST18,Tlr,TLR10,TRIM69	44	27
2	Gene Expression, Cell-To-Cell Signaling and Interaction, Cellular Assembly and Organization	ABCB4,APOE,CHN2,CHRNA2,CHRNA10,CUZD1,DNMT3L,ERK,FGF1,FXR ligand-FXR-Retinoic acid-RXRα,GAST,Growth hormone,H19,HDC,INSL4,KLB,LGI1,Mapk,MIA,MST1R,NR1H4,NRG,NRTN,NTF3,NTSR2,PVALB,RAPGEF1,Rsk,Rxr,SULT2A1,TRHR,UCN, UCN3,VitaminD3-VDR-RXR,WISP1	43	27
3	Cardiovascular Disease, Cell Morphology, Cell-To-Cell Signaling and Interaction	ACTA2,Actin,ADAM30,ADAM21 (includes EG:8747),ALOX12,CCBP2,CCL14,CX3CL1,DAPK3,EPHB1,GAL3ST1,Integrin,KLK15,Laminin,Metalloprotease,Mmp,MMP11,MYH7,MYL7, Myosin,P38 MAPK,PFDN2,PROZ,PRSS1 (includes EG:5644),PRX,SERPINA10,Tgf beta,THY1,TINAG,TMPRSS3,TMPRSS11F,TNNC1,TRIM54,Trypsin,TSPAN4	41	26
4	Post-Translational Modification, Carbohydrate Metabolism, Drug Metabolism	ACR,ADAMTS14,C11ORF52,CASP14,CD1D,CLPB,COQ3,CPA5,CXADR,CYP2B6,CYP4F3,DPM1,DPM2,ENPEP,FURIN,HNF4A,IL20,IL22RA1 ,IMMP2L,ITIH4,MYO1A,PEPC (includes EG:109616),peptidase,PGM3,PHB2,PRODH2,PRT5,PRT6,RAI2,RNF150,SENP5 (includes EG:303874),SLC22A18AS,TM4SF4,UPG2,UROS	39	25
5	Energy Production, Cell Morphology, Skeletal and Muscular System Development and Function	ABLIM1,ACTN3,Akt,Alpha Actinin,AMPK,ANGPT2,ASAH2,Collagen(s),COX6A2,CSRP3,F Actin,FAK,Glycogen synthase,GNA13,Gsk3,GYS2,HSPB8,INS,Insulin,p70 S6k,PAX4,PCK1,PDPK1,PEPCK,PKN1,PP2A,PYGM,Rac,Ras homolog,RETNLB (includes EG:84666),RHOD,SORBS2,SSTR5,STS,TNS1	30	21
6	Cell Cycle, Cellular Assembly and Organization, DNA Replication, Recombination, and Repair	14-3-3,AR,BACH1,BRCA1,BRDT,C9,Calcineurin protein(s),Caspase,CNKSR1,Cyclin A,E2f,Filamin,FRK,FXYD3,GAS2L1,Hdac,HEXIM1,Histone h4,Hsp70,Hsp90,Jnk,KIF5A,MAP2K1/2,Nfat (family),NOC2L,PFTK1,PTGES,Ras,Rb,RUNX3,SPOCD1,TACC2,TP73,TRPV6,Vegf	22	19
7	Cancer, Cell Morphology, Cellular Development	ADCY,AMH,Ap1,ATAD4,CACNA1S,Calmodulin,Cbp/p300,CDH1,Ck2,CNGA3,COL3A1,Creb,ERK1/2,FSH,G,G alpha,GNAS,GNRHR,hCG,Histone h3,HSD17B1,IL10RA,KCNN4,KRT1,LDL,MED22,NIPSNAP1,PI3K,Pka,PKLR,PLC,PRKACG,RNA polymerase II,SRRM2,VAMP8	20	18
8	Molecular Transport, Gene Expression, Organismal Development	AQP1,ASCL2,BCAS1,beta-estradiol,C9ORF84,CFTR,CLCA1 (includes EG:1179),CLCN3,CSTB,FAM101A,FDFT1,INPP5J,INPP5K,KCNMB1,LGALS4,LIMA1,LRRN4,MAST2,MDFI (includes EG:4188),MKI67,PDZD3,PDZK1,PHLDA1,PLEKHA6,PRB2,RCN2,RGS3,SEC61A1,SEC61A2,SLC17A1,SLC34A1,SLC34A2,SPRR3 (includes EG:6707),SUSD2,TMEM43	17	14
9	Cell Signaling, Molecular Transport, Vitamin and Mineral Metabolism	5-HTR3,ACER1,AMPAR,ATP2A1,Ca2+,CABP5,Cacng,CACNG5,CACNG7,Calmodulin-Ca2+-CaMKII+Calmodulin-Ca2,Calmodulin-CaMKII-Ca2+,CaMKII,CCL28,CCR10,CES1 (includes EG:1066),CES2 (includes EG:234671),DLG4,EPX,FCN2,FKBP7,GRIN2D,HRC,HSPA5,HTR3A,HTR3B,ITGAD,KLF16,MASP1,MPP1,NLGN3,PDE6B,RIC3 (includes EG:79608),SLN (includes EG:6588),SP1,TRPM8	17	14
10	Carbohydrate Metabolism, Drug Metabolism, Small Molecule Biochemistry	ACSM3,ANXA8 (includes EG:653145),BAMBI,CBR3,CHMP2B,CNGA1,CNN1,DECR1,EPB41L1,FBXW10 (includes EG:10517),FILIP1L,GJA1,GLCE,HAS1,HAS3,LSR,MAST2,MXI1,NUMA1,PLOD1,PRODH,RDH11,RNF43,RYBP,SHMT1,SLC16A3,SLC40A1, SOX30,SUZ12,TGFB1,TRAF2,UBB,Ube3,VASN,VHL	15	13
11	Drug Metabolism, Endocrine System Development and Function, Lipid Metabolism	ARNT2,ATXN1,BCL6,CLEC4F,CRISP2,DSC1,EIF4ENIF1,FOXI1,HGFAC,HNF1A,ICMT,KCNJ15,KIF12,KLF11,MAGEA9,OAZ1,PANX3,SLC12 A3,SLC22A11,SMCR7,ST6GALNAC6,TACR2,TCF7L1,TRIM32,UBQLN1,UGT1A6,UGT1A10 (includes EG:54575),UGT1A8 (includes EG:54576),UGT1A9 (includes EG:54600),UGT2B4,UGT2B7,UGT2B11,UGT2B15,ZG16,ZNF443	15	13
12	Cellular Development, Hair and Skin Development and Function, Cancer	1700052K11RIK,AANAT,ATP6V0D2,CAM,CLDN6,CLDN9,CLDN13,CLDN14,CLDN17,CLDN19,CLDN20,CLDN21,CLDN22,CLDN23,CREB1, FN1,GBGT1,GK2,JUN,LACRT,LACTB,lipid,NUS1 (includes EG:52014),PDGF BB,RB1,SNAPC5,SPRR1A,SPRR2D,SPTLC3,TGM1,TGM3,TNFSF11,TNKS1BP1,TOB1,Transferase	15	13
13	Cell-To-Cell Signaling and Interaction, Cellular Growth and Proliferation, Hematological System Development and Function	ARL4C,BTNL2,CACNG3,CCL3L1,CD97,CD274,CD276,CDA,cholecalciferol,CLEC4C,CLEC7A,CREBBP,CSF2,CX3CR1,EFS,F2,FFAR2,GPNM B,heparin,IL2,IL6,JUNB,KLRB1C,PDCD1LG2,PKHD1,PRELP,RAB8A,RNASE3,SPRR1B,TIMD4,TPP1,TREML1,UCK2,UGT1A3,VTCN1	15	13
14	Cancer, Cell Morphology, Renal and Urological Disease	BCL9L,CDH22,CDH4 (includes EG:1002),CEACAM7,CRAT,CTNNAL1,CTNNB1,DDX19B,DPPA2,DVL3,DVL1L1,EGF,FBXO44,FBXW2,FGF18,FRAT1,FZD6,GCM1,KCTD1,L RRFIP2,MYOZ3,NFYB,PDPN,PHLDA2,PPP3CA,PTGFR,RWDD2A,RWDD2B,SALL4,SEMA6B,SFRP4,SH2D3A,SKP1,TAX1BP3,thyroid hormone	14	13
15	Metabolic Disease, Nutritional Disease, Kidney Failure	ACO1,ADAMTS10 (includes EG:81794),AKT1,APOA1,BSG,C1QC,EPO,GALP,HK3,KISS1R,LECT1,LEP,LRIT1,MCTS1,NTS,NTSR1,NUPR1,PDE1A,PDE3A,PDE3B,PDE6C ,PIK3IP1,PLCL2,PPP1CA,PRDM7,PRLH,propionic acid,RRP1B,SLC13A2,SLC16A1,SLC16A4,SLC44A2,TCL1B,TIE1,TUSC3	13	12
16	Lipid Metabolism, Molecular Transport, Small Molecule Biochemistry	AGT,ALB,aldosterone,arachidonic acid,ARFGAP3,AZGP1,CUBN,CYP11B2,DCD,DGKG,FXYD4,GIF,GPNMB,HLX,HNF1B,IFNG,IFT88,KCNK18,KCNMB1,LUC7L,MCPT1,NMUR2 ,PABPC3,PLA2G3,PLA2G1B,PLA2G2D,PLA2G2E,PLA2G2F,PLA2G4C,PLA2G4F,PMP2,RRAGD,SLC14A2,STAT4,STAT4 dimer	13	12
17	Cell Signaling, Nucleic Acid Metabolism, Small Molecule Biochemistry	ACAA1B,ADIG,ATP4A,ATP4B,CCND1,CCNDBP1,cyclic AMP,DPT,EIF2C4,ERBB2,GDP-Gnat1-Gngt1-Transducin beta (rod),GDP-Gnat2-Gngt2-Transducin beta (cone),GPHA2,GPHB5,GPR3,GPR12,GPR65,GRM4,H+/K+-exchanging ATPase,LPCAT1,LRFN3,MIR125B1,NUP50,OPN3,OPN4,OPN5,OPN1LW (includes EG:20164),OPN1LW (includes EG:5956),OPN1MW,OPN1SW,Opsin,POMC,PPARG,RGR,SAG	13	12
18	Genetic Disorder, Inflammatory Disease, Neurological Disease	C1q,C1QB,C1QC,CD93,CD300E,CFH,CR,CRIM1,ELSPBP1,FMOD,HFE2,Inflammasome,iron,MYCN,MYOD1,NALP,NLR,NLRP2,NLRP3,NLR P5,NLRP6,NLRP7,NLRP8,NLRP9,NLRP10,NLRP11,NLRP12,NLRP13,NLRP14,PPP4C,PXMP2,SCN4A,TMEM109,TNF,UMOD	12	11

Hypomethylated in CVS versus MBC **Table S1C**

¬© 2000-2009 Ingenuity Systems, Inc. All rights reserved.

ID	Top Functions	Molecules in Network	Score	Focus Molecules
19	Genetic Disorder, Immunological Disease, Post-Translational Modification	AHCTF1,AIPL1,C12ORF4,C20ORF160,CRCT1 (includes EG:54544),DNAJA2,DNAJB7,DNAJB8,DNAJB12,DNAJB13,DNAJB14,DNAJC4,DNAJC8,DNAJC9,DNAJC10,DNAJC11,DNAJC15,DNAJC16,DNAJC17,DNAJC18,DNAJC19,DNAJC21,DNAJC5B,DNAJC5G,FAM76A,GNAO1,Hsp22/Hsp40/Hsp90,KCTD12,MIR93 (includes EG:407050),MIRLET7B (includes EG:406884),MIRLET7E (includes EG:406887),RGS11,SLC35C1,SLMAP,TAAR5	12	11
20	Amino Acid Metabolism, Post-Translational Modification, Small Molecule Biochemistry	amino acids,APP,ASNA1,BMPR2,CD5L,CSNK1G1,Cu+,DULLARD,DUSP8,GDF6,GFRA4,GLRX,HMGCS2,hydrogen peroxide,ICMT,MAMDC2,MAP3K6,MAP3K5 (includes EG:293015),MAT1A,MIR199A1,MMP10,OGDH,PIP,PPME1,PRKX,PSPN,PTCH2,PTPRO,RABGGTA,RET,SGK1,SLC7A11,TPTE,TSSK6,TWF1	11	11
21	Genetic Disorder, Metabolic Disease, Cellular Assembly and Organization	ACTL6B,APOL5,ASB16,CHRD,CSH2,DDX52,DUSP15,ESR1,esr1/esr2,FBXO2,GCDH,GLTSCR1,GRB2,ISG20L2,MAPK15,MYC,MYO9A,PALM2-AKAP2 (includes EG:445815),PEX10,PEX12,PEX13,PEX19,PHACTR2,PHF5A,PRICKLE3,PROK1,PTPRC,PTPRS (includes EG:5802),PXMP3,SAFB2,SEPN1 (includes EG:57190),SHROOM2,SLCO1C1,SMARCA4,SRC	11	10
22	Cellular Assembly and Organization, Genetic Disorder, Respiratory Disease	14-3-3(β,γ,θ,η,ζ),ABI3,ARHGAP21,ARHGEF16,C12ORF51,C22ORF9,CBY1,CEP170,CTCF,DENND4A,DNAI1,DYNLT1,HSPB9,KIF1C,KLC4,KRTAP19-5,LARP1,MIR1,MIRN339,NADK (includes EG:65220),OSBPL7,OTUB1,PHLDB2,RAB25,RAB11FIP1,RAB11FIP2,REM1,SAMD4B,SH3BP5L,SLC5A10,SRGAP2,SYNPO2,YWHAD,YWHAG,YWHAZ	11	10
23	Developmental Disorder, Ophthalmic Disease, Cancer	APCDD1,APOBEC1,APOBEC2,ASB2,ATF5,BLZF1,C1QTNF5,CCL18,CYP2C18,CYP2C39,CYP2D12,CYP2E1,CYP2S1,CYP4B1,CYP4F11,DLGAP5,HERC3,HS3ST1,MAPK6,Mdm2-Tp53-ubiquitin,MFRP,MT1L,PLGLB2,Proteasome,retinoic acid,SLC19A2,SOD2,STRA6,TCEB1,TCEB3C,TP53,TRIM22,TULP2,Ubiquitin,WSB1	10	10
24	Gene Expression	TAF3,TBPL2	2	1
25	Renal and Urological System Development and Function, Cardiovascular Disease, Cell Morphology	ACE,PRSS21	2	1
26	Carbohydrate Metabolism, Small Molecule Biochemistry, Nervous System Development and Function	GPR35,kynurenic acid	2	1
27	Molecular Transport, Protein Trafficking, Cancer	RGPD5,TNPO1	2	1
28	Cancer, Reproductive System Disease	BPIL2,MIR26B (includes EG:407017)	2	1
29	Carbohydrate Metabolism, Molecular Transport, Small Molecule Biochemistry	GPR81,lactic acid	2	1
30	Organ Development, Renal and Urological System Development and Function	BCL9,PYGO1	2	1
31	Endocrine System Disorders, Genetic Disorder, Metabolic Disease	BSCL2,TMEM19	2	1
32	Cell Morphology, Renal and Urological System Development and Function, Genetic Disorder	UPK2,UPK1A	2	1
33	Skeletal and Muscular System Development and Function	CLEC3A,MIRN352	2	1
34	Cancer, Reproductive System Disease	SCGB1D2,SCGB2A2 (includes EG:4250)	2	1
35	Amino Acid Metabolism, Molecular Transport, Small Molecule Biochemistry	Gat,SLC6A13	2	1
