# Supplementary material for: Structural and Regulatory Characterization of the Placental Epigenome at Its Maternal Interface
Source: PLoS One. 2011 Feb 23;6(2):e14723. doi: 10.1371/journal.pone.0014723 (PMC3044138; doi:10.1371/journal.pone.0014723)
Supplement: Table S4 — IPA biological pathway analysis of genes hypomethylated in CVS versus MBC. (0.31 MB PDF) [file pone.0014723.s004.pdf]

				¬© 2000-2009 Ingenuity Systems, Inc. All rights reserved.	
**Category**	**Function**	**Function Annotation**	**P-Value**	**Molecules**	**# Molecules**
Gene Expression	binding	binding of AP1 consensus site	1.94E-05	ALOX12, AR, CYP11B2, FGF1, GNRHR, IFNB1, INS, NR1H4	8
Gene Expression	binding	binding of gastrin response element	6.28E-04	GAST, HDC	2
Gene Expression	activation	activation of DR1 response element	1.21E-02	HNF4A, NR1H4	2
Gene Expression	activation	activation of sterol response element	2.01E-02	BRCA1, GNA13	2
Gene Expression	activation	activation of hypoxia response element	2.47E-02	HNF4A, VHL	2
Gene Expression	activation	activation of gastrin response element	2.51E-02	GAST	1
Gene Expression	transcription	initiation of transcription of synthetic promoter	2.51E-02	BRCA1	1
Cancer	tumorigenesis	tumorigenesis	4.50E-05	ABCB4, ACTA2, ACTN3, AMH, ANGPT2, ANKRD9, AOC3, APOE, AR, ATF5, ATP4A, ATP4B, BACH1, BCAS1, BRCA1, C9, CACNA1S, CACNG3, CCBP2, CD1D, CDH1, CFHR1, CHRNA2, CHRNA10, COL3A1, COX6A2, CRYBB2, CSRP3, CTNNAL1, CX3CL1, CXADR, CYP11B2, CYP2E1, DAK, DCD, DNAJC15, DPM1, DPT, DSC1, EFS, ENPEP, FCGR3A, FGF1, FGG, FURIN, GAST, GNA13, GNAS, GNRHR, H19, HNF4A, HSPB8, HTR3A, HTR3B, IFNB1, IFT88, INPP5J, INS, ITIH4, KLK15, KRT23, LACRT, LBP, LGI1, LRRN4, MIA, MMP11, MST1R, MXI1, MYO1A, NTSR1, NUP50, PDPK1, PIP, PKHD1, PLAT, PLGLB2, PPP4C, PRELP, PROK1, PTCH2, PTGES, RGPD5, RUNX3, S100P, SCGB1D2, SCGB2A1, SCN4A, SCRG1, SERPINB5, SLC16A4, SLC17A4, SLC39A5, SLC7A11, SORBS2, SPRR2D, SSTR5, STS, TCL1B, TGM3, TM4SF4, TNS1, TP73, TSPAN4, TSSK6, TUSC3, VHL, VTCN1, WISP1	109
Cancer	tumorigenesis	tumorigenesis of kidney cell lines	5.98E-03	DAK, TSSK6	2
Cancer	tumorigenesis	tumorigenesis of prostate cancer cell lines	1.59E-02	CXADR, GNAS	2
Cancer	neoplasia	neoplasia	1.95E-04	ABCB4, ACTA2, ACTN3, AMH, ANGPT2, ANKRD9, AOC3, APOE, AR, ATF5, ATP4A, ATP4B, BACH1, BCAS1, BRCA1, C9, CACNA1S, CACNG3, CCBP2, CDH1, CFHR1, CHRNA2, CHRNA10, COL3A1, COX6A2, CRYBB2, CSRP3, CTNNAL1, CX3CL1, CYP11B2, CYP2E1, DCD, DNAJC15, DPM1, DPT, EFS, ENPEP, FCGR3A, FGF1, FGG, FURIN, GAST, GNA13, GNAS, GNRHR, H19, HNF4A, HSPB8, HTR3A, HTR3B, IFNB1, IFT88, INPP5J, INS, ITIH4, KLK15, KRT23, LACRT, LBP, LGI1, LRRN4, MIA, MMP11, MST1R, MXI1, MYO1A, NTSR1, NUP50, PDPK1, PIP, PKHD1, PLAT, PLGLB2, PPP4C, PRELP, PTCH2, PTGES, RGPD5, S100P, SCGB1D2, SCGB2A1, SCN4A, SCRG1, SLC16A4, SLC17A4, SLC39A5, SLC7A11, SORBS2, SPRR2D, SSTR5, STS, TCL1B, TGM3, TM4SF4, TP73, TSPAN4, TUSC3, VHL, VTCN1, WISP1	100
Cancer	developmental process	developmental process of bladder cancer cell lines	3.20E-04	CDH1, CXADR, FGF1, TP73	4
Cancer	cancer	cancer	4.16E-04	ABCB4, ACTA2, AMH, ANGPT2, ANKRD9, AOC3, APOE, AR, ATF5, ATP4A, ATP4B, BACH1, BCAS1, BRCA1, C9, CACNA1S, CACNG3, CCBP2, CDH1, CFHR1, CHRNA2, CHRNA10, COL3A1, COX6A2, CRYBB2, CTNNAL1, CX3CL1, CYP2E1, DCD, DNAJC15, DPM1, DPT, EFS, ENPEP, FCGR3A, FGF1, FGG, FURIN, GAST, GNA13, GNAS, GNRHR, H19, HNF4A, HSPB8, HTR3A, HTR3B, IFT88, INPP5J, ITIH4, KLK15, KRT23, LACRT, LBP, LGI1, LRRN4, MIA, MMP11, MST1R, MXI1, MYO1A, NTSR1, NUP50, PDPK1, PIP, PKHD1, PLAT, PLGLB2, PPP4C, PRELP, PTCH2, PTGES, RGPD5, S100P, SCGB1D2, SCGB2A1, SCN4A, SCRG1, SLC16A4, SLC17A4, SLC39A5, SLC7A11, SORBS2, SPRR2D, SSTR5, STS, TCL1B, TGM3, TM4SF4, TP73, TSPAN4, TUSC3, VHL, VTCN1, WISP1	95
Cancer	adhesion	adhesion of kidney cancer cell lines	6.28E-04	CDH1, VHL	2
Cancer	adhesion	adhesion of germ cell tumor cell lines	2.51E-02	EPHB1	1
Cancer	binding	binding of bladder cancer cell lines	6.28E-04	CX3CL1, CXADR	2
Cancer	binding	binding of tumor cell lines	2.11E-02	AR, CX3CL1, CXADR, GAL3ST1, INS, NR1H4, PHB2, SERPINB5, THY1	9
Cancer	binding	binding of colon carcinoma cells	2.51E-02	ST6GALNAC6	1
Cancer	signaling	signaling of tumor cell lines	6.28E-04	AR, PDCD1	2
Cancer	tumor	tumor	1.13E-03	ACTA2, ACTN3, AMH, ANGPT2, APOE, AR, ATP4A, ATP4B, BRCA1, C9, CACNG3, CCBP2, CDH1, CHRNA2, CHRNA10, COL3A1, COX6A2, CTNNAL1, CX3CL1, CYP11B2, DCD, DPT, DSC1, ENPEP, FCGR3A, FGF1, FGG, FURIN, GAST, GNA13, GNAS, GNRHR, H19, HNF4A, HSPB8, HTR3A, HTR3B, IFT88, INPP5J, KLK15, LACRT, LGI1, LRRN4, MIA, MMP11, MST1R, MXI1, NTSR1, NUP50, PDPK1, PKHD1, PLAT, PPP4C, PRELP, PROK1, PTCH2, PTGES, RGPD5, SCGB1D2, SCN4A, SCRG1, SERPINB5, SLC16A4, SLC7A11, SORBS2, SPRR2D, SSTR5, STS, TCL1B, TGM3, TNS1, TP73, TSPAN4, TUSC3, VHL, WISP1	76
Cancer	primary tumor	primary tumor	1.72E-03	ACTA2, ACTN3, AMH, ANGPT2, APOE, AR, ATP4A, ATP4B, BRCA1, C9, CACNG3, CCBP2, CDH1, CHRNA2, CHRNA10, COL3A1, COX6A2, CTNNAL1, CX3CL1, CYP11B2, DCD, DPT, ENPEP, FCGR3A, FGF1, FGG, FURIN, GAST, GNA13, GNAS, GNRHR, H19, HNF4A, HSPB8, HTR3A, HTR3B, IFT88, INPP5J, KLK15, LACRT, LGI1, LRRN4, MIA, MMP11, MST1R, MXI1, NTSR1, NUP50, PDPK1, PLAT, PPP4C, PRELP, PTCH2, PTGES, RGPD5, SCGB1D2, SCN4A, SCRG1, SLC16A4, SLC7A11, SORBS2, SPRR2D, SSTR5, STS, TCL1B, TGM3, TP73, TSPAN4, TUSC3, VHL, WISP1	71
Cancer	malignant tumor	malignant tumor	3.04E-03	ACTA2, AMH, ANGPT2, APOE, AR, ATP4A, ATP4B, BRCA1, C9, CACNG3, CCBP2, CDH1, CHRNA2, CHRNA10, COL3A1, COX6A2, CTNNAL1, CX3CL1, DCD, DPT, ENPEP, FCGR3A, FGF1, FGG, FURIN, GAST, GNA13, GNAS, GNRHR, H19, HNF4A, HSPB8, HTR3A, HTR3B, IFT88, INPP5J, KLK15, LACRT, LGI1, LRRN4, MIA, MMP11, MST1R, MXI1, NTSR1, NUP50, PDPK1, PLAT, PPP4C, PRELP, PTCH2, PTGES, RGPD5, SCGB1D2, SCN4A, SCRG1, SLC16A4, SLC7A11, SORBS2, SPRR2D, SSTR5, STS, TCL1B, TGM3, TP73, TSPAN4, TUSC3, VHL	68
Cancer	cell-cell contact	cell-cell contact of tumor cell lines	3.65E-03	CDH1, TRPV6	2
Cancer	cell-cell contact	cell-cell contact of squamous carcinoma cells	2.51E-02	CDH1	1
Cancer	growth	growth of bladder cancer cell lines	3.72E-03	CDH1, CXADR, TP73	3
Cancer	growth	arrest in growth of kidney cancer cell lines	2.51E-02	VHL	1
Cancer	survival	survival of prostate cancer cell lines	3.72E-03	ALOX12, AR, TRPM8	3
Cancer	pathological cyst	pathological cyst	5.71E-03	DSC1, PKHD1, PROK1, SSTR5, TNS1	5

				¬© 2000-2009 Ingenuity Systems, Inc. All rights reserved.	
**Category**	**Function**	**Function Annotation**	**P-Value**	**Molecules**	**# Molecules**
Cancer	adrenal cortex adenoma	adrenal cortex adenoma	5.98E-03	AR, CYP11B2	2
Cancer	invasion	invasion of carcinoma cells	8.21E-03	ADAM21 (includes EG:8747), MST1R, SERPINB5	3
Cancer	invasion	invasion of cancer cells	1.61E-02	ADAM21 (includes EG:8747), CDH1, MST1R, SERPINB5	4
Cancer	invasion	invasion of pancreatic cancer cell lines	2.15E-02	MST1R, PLAT, S100P	3
Cancer	invasion	invasion of tumor	2.40E-02	APOE, FURIN, VHL	3
Cancer	quantity	quantity of tumor cell lines	8.36E-03	AMH, AR, BRCA1, IFNB1, INS, SERPINB5, VHL	7
Cancer	quantity	quantity of prostate cancer cell lines	1.21E-02	AR, SERPINB5	2
Cancer	cell viability	cell viability of prostate cancer cell lines	1.13E-02	AR, BRCA1, SERPINB5	3
Cancer	Zollinger-Ellison syndrome	Zollinger-Ellison syndrome	1.21E-02	ATP4A, ATP4B	2
Cancer	endocrine adenoma	endocrine adenoma	1.21E-02	ATP4A, ATP4B	2
Cancer	ovarian cancer	ovarian cancer	1.25E-02	AMH, APOE, AR, BRCA1, CDH1, CTNNAL1, ENPEP, H19, KRT23, LRRN4, PTGES, S100P, SCGB2A1, TSPAN4, VHL, VTCN1	16
Cancer	formation	formation of pathological cyst	1.30E-02	DSC1, PROK1, TNS1	3
Cancer	formation	formation of B cell lymphoma cells	2.51E-02	MXI1	1
Cancer	formation	formation of androblastoma	2.51E-02	AMH	1
Cancer	formation	formation of leydig cell tumor	2.51E-02	AMH	1
Cancer	morphology	morphology of pheochromocytoma cell lines	1.59E-02	EPB41L1, GNA13	2
Cancer	carcinoid tumor	carcinoid tumor	1.70E-02	HTR3A, HTR3B, SSTR5	3
Cancer	cytostasis	cytostasis of breast cancer cell lines	1.91E-02	BRCA1, CDH1, RHOD	3
Cancer	G0 phase	initiation of G0 phase of kidney cancer cell lines	2.51E-02	VHL	1
Cancer	G2/M phase transition	arrest in G2/M phase transition of hepatoma cell lines	2.51E-02	ATF5	1
Cancer	S phase	entry into S phase of skin cancer cell lines	2.51E-02	CDH1	1
Cancer	androblastoma	androblastoma	2.51E-02	AMH	1
Cancer	apoptosis	delay in initiation of apoptosis of prostate cancer cell lines	2.51E-02	ALOX12	1
Cancer	apoptosis	delay in initiation of apoptosis of skin cancer cell lines	2.51E-02	ALOX12	1
Cancer	attachment	attachment of lung cancer cells	2.51E-02	VHL	1
Cancer	branching	branching of breast cancer cell lines	2.51E-02	CDH1	1
Cancer	cell cycle progression	exit from cell cycle progression of kidney cancer cell lines	2.51E-02	VHL	1
Cancer	cell movement	cell movement of lung cancer cells	2.51E-02	VHL	1
Cancer	cell spreading	cell spreading of lung cancer cells	2.51E-02	VHL	1
Cancer	ciliogenesis	ciliogenesis of kidney cancer cell lines	2.51E-02	VHL	1
Cancer	colony formation	colony formation of skin cancer cell lines	2.51E-02	PTPRS (includes EG:5802)	1
Cancer	cytolysis	cytolysis of bladder cancer cell lines	2.51E-02	CX3CL1	1
Cancer	dispersion	dispersion of bladder cancer cell lines	2.51E-02	FGF1	1
Cancer	epithelial-mesenchymal transition	epithelial-mesenchymal transition of bladder cancer cell lines	2.51E-02	FGF1	1
Cancer	expansion	expansion of bladder cancer cell lines	2.51E-02	CDH1	1
Cancer	hyperplasia	hyperplasia of breast	2.51E-02	BRCA1	1
Cancer	micronucleation	micronucleation of breast cancer cell lines	2.51E-02	BRCA1	1
Cancer	migration	migration of germ cell tumor cell lines	2.51E-02	EPHB1	1
Cancer	mitosis	delay in initiation of mitosis of breast cancer cell lines	2.51E-02	BRCA1	1
Cancer	morphogenesis	morphogenesis of hepatoma cell lines	2.51E-02	CDH1	1
Organismal Development	secretion	secretion of bodily fluid	7.22E-05	ATP4A, ATP4B, GAST, HDC, KCNN4, RUNX3, SLC34A1, UCN	8
Organismal Development	secretion	secretion of gastric acid	2.33E-04	ATP4A, ATP4B, GAST, HDC	4
Organismal Development	sensitivity	sensitivity of mice	8.81E-03	ABCB4, LBP	2
Organismal Development	mass	mass of rodents	1.66E-02	APOE, AR, GALP, INS, NR1H4, PCK1, PDPK1, PRSS8, PTGES, UCN, VHL	11
Cardiovascular Disease	abdominal aortic aneurysm	abdominal aortic aneurysm	1.51E-04	ANGPT2, APOE, PTGES	3
Cardiovascular Disease	destruction	destruction of elastic lamina	1.85E-03	APOE, MMP11	2
Cardiovascular Disease	destruction	destruction of glomerular capillary	2.51E-02	THY1	1
Cardiovascular Disease	hypertrophic cardiomyopathy	hypertrophic cardiomyopathy	3.77E-03	CYP11B2, MYH7, TNNC1, TRIM54	4
Cardiovascular Disease	injury	injury of artery	8.81E-03	APOE, PLAT	2
Cardiovascular Disease	injury	injury of aorta	2.51E-02	APOE	1
Cardiovascular Disease	cardiovascular disorder	cardiovascular disorder of rodents	9.73E-03	ANGPT2, APOE, AR, CSRP3, CX3CL1, FGF1, IFNB1, PDCD1, PDPK1, PSPN, PTGES, RETNLB (includes EG:84666), TRIM54, UCN, UCN3	15
Cardiovascular Disease	cardiovascular disorder	cardiovascular disorder of mice	2.12E-02	ANGPT2, APOE, AR, CSRP3, CX3CL1, IFNB1, PDCD1, PDPK1, PSPN, PTGES, RETNLB (includes EG:84666), TRIM54, UCN3	13
Cardiovascular Disease	quantity	quantity of atherosclerotic lesion	1.21E-02	APOE, PTGES	2
Cardiovascular Disease	damage	damage of blood vessel	1.30E-02	APOE, COL3A1, PLAT	3
Cardiovascular Disease	cerebral amyloid angiopathy	cerebral amyloid angiopathy	1.59E-02	ACTA2, APOE	2
Cardiovascular Disease	leakage	leakage of blood vessel	1.59E-02	APOE, SERPINB5	2
Cardiovascular Disease	leakage	leakage of carotid artery	2.51E-02	APOE	1
Cardiovascular Disease	leakage	leakage of neovessel	2.51E-02	SERPINB5	1
Cardiovascular Disease	hypertension	hypertension of rodents	2.06E-02	ANGPT2, APOE, FGF1, PTGES	4
Cardiovascular Disease	area	area of atherosclerotic lesion	2.40E-02	APOE, CX3CL1, NR1H4	3
Cardiovascular Disease	infarction	infarction of mice	2.47E-02	APOE, PSPN	2
Cardiovascular Disease	atherosclerosis	atherosclerosis of carotid sinus	2.51E-02	APOE	1
Cardiovascular Disease	atherosclerosis	atherosclerosis of coronary artery	2.51E-02	APOE	1

¬© 2000-2009 Ingenuity Systems, Inc. All rights reserved.

Category	Function	Function Annotation	P-Value	Molecules	# Molecules
Cardiovascular Disease	calcification	calcification of aortic valve	2.51E-02	LECT1	1
Cardiovascular Disease	dysfunction	dysfunction of vasculature	2.51E-02	INS	1
Cardiovascular Disease	endothelial dysfunction	endothelial dysfunction of mice	2.51E-02	APOE	1
Cardiovascular Disease	instability	instability of vasculature	2.51E-02	ANGPT2	1
Cardiovascular Disease	mild hypertriglyceridemia	mild hypertriglyceridemia of mice	2.51E-02	APOE	1
Cardiovascular Disease	morphology	morphology of atherosclerotic lesion	2.51E-02	APOE	1
Renal and Urological Disease	developmental process	developmental process of bladder cancer cell lines	3.20E-04	CDH1, CXADR, FGF1, TP73	4
Renal and Urological Disease	adhesion	adhesion of kidney cancer cell lines	6.28E-04	CDH1, VHL	2
Renal and Urological Disease	binding	binding of bladder cancer cell lines	6.28E-04	CX3CL1, CXADR	2
Renal and Urological Disease	growth	growth of bladder cancer cell lines	3.72E-03	CDH1, CXADR, TP73	3
Renal and Urological Disease	growth	arrest in growth of kidney cancer cell lines	2.51E-02	VHL	1
Renal and Urological Disease	tumorigenesis	tumorigenesis of kidney cell lines	5.98E-03	DAK, TSSK6	2
Renal and Urological Disease	G0 phase	initiation of G0 phase of kidney cancer cell lines	2.51E-02	VHL	1
Renal and Urological Disease	cell cycle progression	exit from cell cycle progression of kidney cancer cell lines	2.51E-02	VHL	1
Renal and Urological Disease	ciliogenesis	ciliogenesis of kidney cancer cell lines	2.51E-02	VHL	1
Renal and Urological Disease	cytolysis	cytolysis of bladder cancer cell lines	2.51E-02	CX3CL1	1
Renal and Urological Disease	cytolysis	cytolysis of mesangial cells	2.51E-02	THY1	1
Renal and Urological Disease	destruction	destruction of glomerular capillary	2.51E-02	THY1	1
Renal and Urological Disease	dispersion	dispersion of bladder cancer cell lines	2.51E-02	FGF1	1
Renal and Urological Disease	epithelial-mesenchymal transition	epithelial-mesenchymal transition of bladder cancer cell lines	2.51E-02	FGF1	1
Renal and Urological Disease	expansion	expansion of bladder cancer cell lines	2.51E-02	CDH1	1
Renal and Urological Disease	incidence	incidence of renal lesion	2.51E-02	PLAT	1
Lipid Metabolism	modification	modification of eicosanoid	3.88E-04	ALOX12, APOE, CYP2E1, CYP4F3, PTGES, UGT1A3	6
Lipid Metabolism	modification	modification of leukotriene	5.09E-04	ALOX12, CYP4F3, UGT1A3	3
Lipid Metabolism	moiety attachment	moiety attachment of lipid	4.26E-04	CYP2E1, CYP4F3, GBGT1, INS, PCK1, ST6GALNAC6, UGT1A3	7
Lipid Metabolism	moiety attachment	moiety attachment of fatty acid	1.12E-03	CYP2E1, CYP4F3, INS, PCK1	4
Lipid Metabolism	moiety attachment	moiety attachment of leukotriene B4	5.98E-03	CYP4F3, UGT1A3	2
Lipid Metabolism	release	release of fatty acid	5.59E-04	GPR81, INS, PLA2G4C, PTGES	4
Lipid Metabolism	release	release of cholesterol	2.47E-02	APOE, FGF1	2
Lipid Metabolism	biogenesis	biogenesis of cholesterol	6.28E-04	APOE, FGF1	2
Lipid Metabolism	uptake	uptake of digoxin	1.18E-03	ABCB4, NR1H4, SLCO1C1	3
Lipid Metabolism	degradation	degradation of ceramide	1.85E-03	ACER1, ASAH2	2
Lipid Metabolism	degradation	degradation of cholesterol	2.01E-02	APOE, NR1H4	2
Lipid Metabolism	quantity	quantity of sulfatides	3.65E-03	APOE, GAL3ST1	2
Lipid Metabolism	quantity	quantity of 12-hydroxyeicosatetraenoic acid	2.01E-02	ALOX12, APOE	2
Lipid Metabolism	biliary excretion	biliary excretion of lipid	5.98E-03	ABCB4, NR1H4	2
Lipid Metabolism	re-esterification	re-esterification of fatty acid	5.98E-03	INS, PCK1	2
Lipid Metabolism	excretion	excretion of lipid	6.89E-03	ABCB4, NR1H4, PTGES	3
Lipid Metabolism	excretion	excretion of bile acid	1.21E-02	KLB, NR1H4	2
Lipid Metabolism	conversion	conversion of eicosanoid	1.49E-02	ALOX12, CYP2E1, PTGES	3
Lipid Metabolism	conversion	conversion of prostaglandin h2	2.01E-02	CYP2E1, PTGES	2
Lipid Metabolism	conversion	conversion of leukotriene A4	2.51E-02	ALOX12	1
Lipid Metabolism	conversion	conversion of lipoxin A4	2.51E-02	ALOX12	1
Lipid Metabolism	conversion	conversion of steroid	2.51E-02	STS	1
Lipid Metabolism	hydroxylation	hydroxylation of fatty acid	1.59E-02	CYP2E1, CYP4F3	2
Lipid Metabolism	hydroxylation	hydroxylation of corticosterone	2.51E-02	CYP11B2	1
Lipid Metabolism	hydroxylation	hydroxylation of leukotriene B4	2.51E-02	CYP4F3	1
Lipid Metabolism	metabolism	metabolism of glycolipid	1.60E-02	ACER1, ASAH2, GAL3ST1, GBGT1, ST6GALNAC6	5
Lipid Metabolism	metabolism	metabolism of ceramide	2.47E-02	ACER1, ASAH2	2
Lipid Metabolism	metabolism	metabolism of D-erythro-C16-ceramide	2.51E-02	ACER1	1
Lipid Metabolism	production	production of aldosterone	2.01E-02	ALOX12, ANGPT2	2
Lipid Metabolism	absorption	absorption of digoxin	2.51E-02	ABCB4	1
Lipid Metabolism	absorption	absorption of paclitaxel	2.51E-02	ABCB4	1
Lipid Metabolism	binding	binding of lipoteichoic acid	2.51E-02	LBP	1
Lipid Metabolism	binding	binding of triacylglycerol	2.51E-02	APOE	1
Lipid Metabolism	biosynthesis	biosynthesis of epoprostenol	2.51E-02	PTGES	1
Lipid Metabolism	biosynthesis	biosynthesis of galactosylceramide	2.51E-02	GAL3ST1	1
Lipid Metabolism	concentration	concentration of 1-alpha, 25-dihydroxy vitamin D3	2.51E-02	SLC34A1	1
Lipid Metabolism	concentration	concentration of bile acid	2.51E-02	NR1H4	1
Lipid Metabolism	concentration	concentration of taurocholic acid	2.51E-02	NR1H4	1
Lipid Metabolism	elimination	elimination of cholesterol	2.51E-02	NR1H4	1
Lipid Metabolism	formation	formation of 11-cis-retinal	2.51E-02	RGR	1
Lipid Metabolism	generation	generation of lipid peroxide	2.51E-02	CYP2E1	1
Small Molecule Biochemistry	modification	modification of eicosanoid	3.88E-04	ALOX12, APOE, CYP2E1, CYP4F3, PTGES, UGT1A3	6
Small Molecule Biochemistry	modification	modification of leukotriene	5.09E-04	ALOX12, CYP4F3, UGT1A3	3
Small Molecule Biochemistry	moiety attachment	moiety attachment of lipid	4.26E-04	CYP2E1, CYP4F3, GBGT1, INS, PCK1, ST6GALNAC6, UGT1A3	7
Small Molecule Biochemistry	moiety attachment	moiety attachment of fatty acid	1.12E-03	CYP2E1, CYP4F3, INS, PCK1	4

				¬© 2000-2009 Ingenuity Systems, Inc. All rights reserved.	
**Category**	**Function**	**Function Annotation**	**P-Value**	**Molecules**	**# Molecules**
Small Molecule Biochemistry	moiety attachment	moiety attachment of leukotriene B4	5.98E-03	CYP4F3, UGT1A3	2
Small Molecule Biochemistry	release	release of fatty acid	5.59E-04	GPR81, INS, PLA2G4C, PTGES	4
Small Molecule Biochemistry	release	release of cholesterol	2.47E-02	APOE, FGF1	2
Small Molecule Biochemistry	biogenesis	biogenesis of cholesterol	6.28E-04	APOE, FGF1	2
Small Molecule Biochemistry	hydroxylation	hydroxylation of bupropion	6.28E-04	CYP2B6, CYP2E1	2
Small Molecule Biochemistry	hydroxylation	hydroxylation of fatty acid	1.59E-02	CYP2E1, CYP4F3	2
Small Molecule Biochemistry	hydroxylation	hydroxylation of chlorzoxazone	2.51E-02	CYP2E1	1
Small Molecule Biochemistry	hydroxylation	hydroxylation of corticosterone	2.51E-02	CYP11B2	1
Small Molecule Biochemistry	hydroxylation	hydroxylation of leukotriene B4	2.51E-02	CYP4F3	1
Small Molecule Biochemistry	uptake	uptake of digoxin	1.18E-03	ABCB4, NR1H4, SLCO1C1	3
Small Molecule Biochemistry	uptake	uptake of indomethacin	5.98E-03	SLC13A2, SLC22A11	2
Small Molecule Biochemistry	uptake	uptake of salicylic acid	5.98E-03	SLC13A2, SLC22A11	2
Small Molecule Biochemistry	production	production of D-glucose	1.69E-03	APOE, INS, NR1H4, PCK1, RETNLB (includes EG:84666)	5
Small Molecule Biochemistry	production	production of ATP	8.81E-03	INS, PAX4	2
Small Molecule Biochemistry	production	production of aldosterone	2.01E-02	ALOX12, ANGPT2	2
Small Molecule Biochemistry	degradation	degradation of ceramide	1.85E-03	ACER1, ASAH2	2
Small Molecule Biochemistry	degradation	degradation of cholesterol	2.01E-02	APOE, NR1H4	2
Small Molecule Biochemistry	homeostasis	homeostasis of inorganic cation	3.06E-03	APOE, CCL14, CSRP3, GPR12, MT4, PYGM, SLC39A5, TMPRSS3, TRPM8	9
Small Molecule Biochemistry	homeostasis	homeostasis of divalent cations	4.48E-03	APOE, CCL14, CSRP3, GPR12, PYGM, SLC39A5, TRPM8	7
Small Molecule Biochemistry	homeostasis	homeostasis of Ca2+	7.09E-03	APOE, CCL14, CSRP3, GPR12, PYGM, TRPM8	6
Small Molecule Biochemistry	quantity	quantity of sulfatides	3.65E-03	APOE, GAL3ST1	2
Small Molecule Biochemistry	quantity	quantity of 12-hydroxyeicosatetraenoic acid	2.01E-02	ALOX12, APOE	2
Small Molecule Biochemistry	biliary excretion	biliary excretion of lipid	5.98E-03	ABCB4, NR1H4	2
Small Molecule Biochemistry	re-esterification	re-esterification of fatty acid	5.98E-03	INS, PCK1	2
Small Molecule Biochemistry	excretion	excretion of lipid	6.89E-03	ABCB4, NR1H4, PTGES	3
Small Molecule Biochemistry	excretion	excretion of bile acid	1.21E-02	KLB, NR1H4	2
Small Molecule Biochemistry	glucuronidation	glucuronidation of thyroxine	1.21E-02	UGT1A3, UGT1A6	2
Small Molecule Biochemistry	glucuronidation	glucuronidation of acetaminophen	2.51E-02	UGT1A6	1
Small Molecule Biochemistry	conversion	conversion of eicosanoid	1.49E-02	ALOX12, CYP2E1, PTGES	3
Small Molecule Biochemistry	conversion	conversion of prostaglandin h2	2.01E-02	CYP2E1, PTGES	2
Small Molecule Biochemistry	conversion	conversion of glycerol	2.51E-02	PCK1	1
Small Molecule Biochemistry	conversion	conversion of leukotriene A4	2.51E-02	ALOX12	1
Small Molecule Biochemistry	conversion	conversion of lipoxin A4	2.51E-02	ALOX12	1
Small Molecule Biochemistry	conversion	conversion of steroid	2.51E-02	STS	1
Small Molecule Biochemistry	metabolism	metabolism of glycolipid	1.60E-02	ACER1, ASAH2, GAL3ST1, GBGT1, ST6GALNAC6	5
Small Molecule Biochemistry	metabolism	metabolism of ceramide	2.47E-02	ACER1, ASAH2	2
Small Molecule Biochemistry	metabolism	metabolism of D-erythro-C16-ceramide	2.51E-02	ACER1	1
Small Molecule Biochemistry	metabolism	metabolism of GDP-D-mannose	2.51E-02	DPM1	1
Small Molecule Biochemistry	metabolism	metabolism of ethanol	2.51E-02	CYP2E1	1
Small Molecule Biochemistry	metabolism	metabolism of glucose-1-phosphate	2.51E-02	PGM3	1
Small Molecule Biochemistry	metabolism	metabolism of glucuronic acid	2.51E-02	UGT1A6	1
Small Molecule Biochemistry	absorption	absorption of digoxin	2.51E-02	ABCB4	1
Small Molecule Biochemistry	absorption	absorption of paclitaxel	2.51E-02	ABCB4	1
Small Molecule Biochemistry	binding	binding of chondroitin sulfate proteoglycan	2.51E-02	APOE	1
Small Molecule Biochemistry	binding	binding of lipoteichoic acid	2.51E-02	LBP	1
Small Molecule Biochemistry	binding	binding of triacylglycerol	2.51E-02	APOE	1
Small Molecule Biochemistry	biosynthesis	biosynthesis of epoprostenol	2.51E-02	PTGES	1
Small Molecule Biochemistry	biosynthesis	biosynthesis of galactosylceramide	2.51E-02	GAL3ST1	1
Small Molecule Biochemistry	biosynthesis	biosynthesis of uroporphyrinogen III	2.51E-02	UROS	1
Small Molecule Biochemistry	concentration	concentration of 1-alpha, 25-dihydroxy vitamin D3	2.51E-02	SLC34A1	1
Small Molecule Biochemistry	concentration	concentration of bile acid	2.51E-02	NR1H4	1
Small Molecule Biochemistry	concentration	concentration of glycine	2.51E-02	SLC6A18	1
Small Molecule Biochemistry	concentration	concentration of taurocholic acid	2.51E-02	NR1H4	1
Small Molecule Biochemistry	deamidation	deamidation of glutamine	2.51E-02	TGM1	1
Small Molecule Biochemistry	deamination	deamination of cytidine	2.51E-02	CDA	1
Small Molecule Biochemistry	demethylation	demethylation of benzphetamine	2.51E-02	CYP2B6	1
Small Molecule Biochemistry	elimination	elimination of cholesterol	2.51E-02	NR1H4	1
Small Molecule Biochemistry	formation	formation of 11-cis-retinal	2.51E-02	RGR	1
Small Molecule Biochemistry	formation	formation of GABA	2.51E-02	GAST	1
Small Molecule Biochemistry	formation	formation of bupropion	2.51E-02	CYP2B6	1
Small Molecule Biochemistry	formation	formation of histamine	2.51E-02	HDC	1
Small Molecule Biochemistry	generation	generation of lipid peroxide	2.51E-02	CYP2E1	1
Small Molecule Biochemistry	inactivation	inactivation of cytarabine	2.51E-02	CDA	1
Neurological Disease	neurological disorder	neurological disorder of mammalia	4.49E-04	APOE, AR, C9, CCBP2, CD1D, CHN2, CHRNA2, CX3CL1, FGF1, FGG, FOXI1, FURIN, GAL3ST1, GCM1, GNAS, GRM4, HDC, HSPB8, IFNB1, IL10RA, KIF5A, LGI1, NRTN, NTF3, PLA2G4C, PLAT, PRODH, PRX, PSPN, PTGES, PVALB, TAAR6, VTCN1	33
Neurological Disease	neurological disorder	neurological disorder of primate	5.02E-04	APOE, AR, C9, CHN2, FGF1, FURIN, GNAS, GRM4, HSPB8, IFNB1, IL10RA, KIF5A, LGI1, NRTN, NTF3, PLA2G4C, PRODH, TAAR6	18

				¬© 2000-2009 Ingenuity Systems, Inc. All rights reserved.	
**Category**	**Function**	**Function Annotation**	**P-Value**	**Molecules**	**# Molecules**
Neurological Disease	neurological disorder	neurological disorder of humans	1.20E-03	APOE, AR, C9, CHN2, FGF1, FURIN, GNAS, GRM4, HSPB8, IFNB1, IL10RA, KIF5A, LGI1, NTF3, PLA2G4C, PRODH, TAAR6	17
Neurological Disease	neurological disorder	neurological disorder of normal cells	6.20E-03	APOE, AR, FGF1, KIF5A, NTF3, PLAT, PRX	7
Neurological Disease	neurological disorder	neurological disorder of rodents	9.39E-03	APOE, AR, CCBP2, CD1D, CHRNA2, CX3CL1, FGG, FOXI1, GAL3ST1, GCM1, GNAS, HDC, IFNB1, KIF5A, NTF3, PLAT, PRX, PSPN, PTGES, PVALB, VTCN1	21
Neurological Disease	neurological disorder	neurological disorder of rats	2.08E-02	CHRNA2, CX3CL1, NTF3, PLAT, PSPN	5
Neurological Disease	dementia	dementia of humans	4.65E-03	APOE, C9, FURIN	3
Neurological Disease	neurodegenerative disorder	neurodegenerative disorder of humans	6.89E-03	APOE, C9, KIF5A	3
Neurological Disease	schizophrenia	schizophrenia of humans	8.90E-03	APOE, CHN2, FGF1, GNAS, GRM4, IL10RA, LGI1, NTF3, PLA2G4C, PRODH, TAAR6	11
Neurological Disease	degeneration	degeneration of neurons	9.37E-03	APOE, AR, FBXO2, KIF5A, NRTN, NTF3, PLAT	7
Neurological Disease	degeneration	degeneration of spiral ganglion	2.51E-02	FBXO2	1
Neurological Disease	degeneration	delay in initiation of degeneration of axons	2.51E-02	PLAT	1
Neurological Disease	neurodegeneration	neurodegeneration of normal cells	1.06E-02	APOE, AR, FGF1, KIF5A, NTF3, PLAT	6
Neurological Disease	cerebrotendinous xanthomatosis	cerebrotendinous xanthomatosis	1.21E-02	APOE, HNF4A	2
Neurological Disease	cerebrotendinous xanthomatosis	cerebrotendinous xanthomatosis of mice	2.51E-02	APOE	1
Neurological Disease	Alzheimer's disease	Alzheimer's disease of organism	1.30E-02	APOE, C9, NTF3	3
Neurological Disease	disease	disease of cerebral cortex	1.30E-02	APOE, GNA13, PTGES	3
Neurological Disease	cerebral amyloid angiopathy	cerebral amyloid angiopathy	1.59E-02	ACTA2, APOE	2
Neurological Disease	organismal abnormalities	organismal abnormalities of cerebral cortex	1.59E-02	APOE, PTGES	2
Neurological Disease	familial Alzheimer's disease	familial Alzheimer's disease	2.01E-02	ACTA2, APOE	2
Neurological Disease	apoptosis	delay in initiation of apoptosis of Purkinje cells	2.51E-02	PLAT	1
Neurological Disease	apoptosis	delay in initiation of apoptosis of granule cells	2.51E-02	PLAT	1
Neurological Disease	astrocytosis	astrocytosis of cell lines	2.51E-02	APOE	1
Neurological Disease	ataxia	ataxia of limb	2.51E-02	RUNX3	1
Neurological Disease	autosomal dominant partial epilepsy with auditory features	autosomal dominant partial epilepsy with auditory features	2.51E-02	LGI1	1
Neurological Disease	demyelination	delay in initiation of demyelination of spinal cord	2.51E-02	PLAT	1
Neurological Disease	diastematomyelia	diastematomyelia of mice	2.51E-02	GCM1	1
Neurological Disease	edema	edema of cerebral cortex	2.51E-02	PTGES	1
Neurological Disease	familial Danish dementia	familial Danish dementia of humans	2.51E-02	FURIN	1
Neurological Disease	loss	loss of striatonigral neurons	2.51E-02	NRTN	1
Neurological Disease	masculinization	masculinization of brain	2.51E-02	AR	1
Neurological Disease	microgliosis	microgliosis of cell lines	2.51E-02	APOE	1
Cellular Development	developmental process	developmental process of epithelial cells	4.82E-04	ABCB4, BRCA1, CDH1, ELF5, FGF1, GNRHR, IL20, KRT8, MST1R, NTF3, RUNX3, SPRR1A, SPRR1B, TGM1, TGM3, UPK2, VHL	17
Cellular Development	differentiation	differentiation of epithelial cells	2.17E-03	BRCA1, CDH1, ELF5, FGF1, IL20, KRT8, SPRR1A, SPRR1B, TGM1, TGM3, UPK2, VHL	12
Cellular Development	differentiation	differentiation of keratinocytes	5.28E-03	CDH1, IL20, SPRR1A, SPRR1B, TGM1, TGM3	6
Cellular Development	differentiation	differentiation of acinar gland cells	5.98E-03	BRCA1, KRT8	2
Cellular Development	differentiation	differentiation of hepatocytes	1.21E-02	FGF1, HNF4A	2
Cellular Development	differentiation	differentiation of endocrine cells	1.49E-02	BRCA1, FGF1, KRT8	3
Cellular Development	differentiation	differentiation of Leydig precursor cells	2.51E-02	AMH	1
Cellular Development	epithelial-mesenchymal transition	epithelial-mesenchymal transition of cells	8.99E-03	CDH1, FGF1, HNF4A, MST1R	4
Cellular Development	epithelial-mesenchymal transition	epithelial-mesenchymal transition of fibroblast cell lines	2.51E-02	HNF4A	1
Cellular Development	growth	growth of exocrine cells	1.21E-02	AR, FGF1	2
Cellular Development	growth	growth of chondrocytes	2.01E-02	FGF1, INS	2
Cellular Development	growth	growth of fibroblastoids	2.51E-02	CDH1	1
Cellular Development	development	development of myofiber	2.47E-02	CXADR, NTF3	2
Cellular Development	development	development of invariant natural killer T cells	2.51E-02	CD1D	1
Cellular Development	development	development of muscle sensory neurons	2.51E-02	NTF3	1
Cellular Development	commitment	commitment of granulocyte-macrophage progenitor cells	2.51E-02	PDPK1	1
Cellular Development	expansion	expansion of granule cell precursors	2.51E-02	IFT88	1
Cellular Development	spermatogenesis	arrest in spermatogenesis of spermatocytes	2.51E-02	AR	1
Visual System Development and Function	transduction	transduction of light stimulus	5.09E-04	AIPL1, PDE6B, PDE6C	3
Visual System Development and Function	vision	vision of organism	2.39E-03	ABLIM1, AIPL1, CNGA1, CNGA3, CRYBB2, MFRP, PDE6B, PDE6C, RGR, TULP2	10
Visual System Development and Function	development	development of photoreceptor outer segments	2.51E-02	IFT88	1
Visual System Development and Function	innervation	innervation of lacrimal gland	2.51E-02	NRTN	1
Molecular Transport	release	release of fatty acid	5.59E-04	GPR81, INS, PLA2G4C, PTGES	4
Molecular Transport	release	release of cholesterol	2.47E-02	APOE, FGF1	2
Molecular Transport	quantity	quantity of calcium	9.79E-04	ANGPT2, APOE, CACNA1S, CCBP2, CX3CL1, FCGR3A, FGF1, GAST, GNA13, GNAS, GNRHR, HNF4A, INS, NMUR2, NTF3, PAX4, SLC34A1, TACR2, THY1, TP73, TRHR, TRPM8	22
Molecular Transport	quantity	quantity of sulfatides	3.65E-03	APOE, GAL3ST1	2
Molecular Transport	quantity	quantity of glycogen	1.22E-02	GNAS, INS, NR1H4, PCK1	4
Molecular Transport	quantity	quantity of Ca2+	1.50E-02	ANGPT2, APOE, CCBP2, CCL28, FCGR3A, FGF1, GAST, GNA13, GNAS, INS, NMUR2, TACR2, TRHR, TRPM8	14
Molecular Transport	quantity	quantity of 12-hydroxyeicosatetraenoic acid	2.01E-02	ALOX12, APOE	2
Molecular Transport	quantity	quantity of phosphate	2.01E-02	HDC, SLC34A1	2
Molecular Transport	uptake	uptake of digoxin	1.18E-03	ABCB4, NR1H4, SLCO1C1	3
Molecular Transport	uptake	uptake of indomethacin	5.98E-03	SLC13A2, SLC22A11	2

				¬© 2000-2009 Ingenuity Systems, Inc. All rights reserved.	
**Category**	**Function**	**Function Annotation**	**P-Value**	**Molecules**	**# Molecules**
Molecular Transport	uptake	uptake of salicylic acid	5.98E-03	SLC13A2, SLC22A11	2
Molecular Transport	biliary excretion	biliary excretion of lipid	5.98E-03	ABCB4, NR1H4	2
Molecular Transport	excretion	excretion of lipid	6.89E-03	ABCB4, NR1H4, PTGES	3
Molecular Transport	excretion	excretion of bile acid	1.21E-02	KLB, NR1H4	2
Molecular Transport	excretion	excretion of Ca2+	2.51E-02	PVALB	1
Molecular Transport	excretion	excretion of phosphate	2.51E-02	SLC34A1	1
Molecular Transport	transport	transport of phosphoric acid	2.47E-02	SLC17A1, SLC34A1	2
Molecular Transport	absorption	absorption of digoxin	2.51E-02	ABCB4	1
Molecular Transport	absorption	absorption of paclitaxel	2.51E-02	ABCB4	1
Molecular Transport	concentration	concentration of 1-alpha, 25-dihydroxy vitamin D3	2.51E-02	SLC34A1	1
Molecular Transport	concentration	concentration of glycine	2.51E-02	SLC6A18	1
Molecular Transport	elimination	elimination of cholesterol	2.51E-02	NR1H4	1
Cell Morphology	volume	volume of muscle cells	6.28E-04	ANGPT2, PDPK1	2
Cell Morphology	morphology	morphology of microvilli	1.85E-03	MIA, MYO1A	2
Cell Morphology	morphology	morphology of plasma membrane projections	2.18E-03	APOE, CHN2, MIA, MYO1A, NTF3	5
Cell Morphology	morphology	morphology of pheochromocytoma cell lines	1.59E-02	EPB41L1, GNA13	2
Cell Morphology	morphology	morphology of cells	1.59E-02	APOE, ATP4A, CDH1, CLCA1 (includes EG:1179), DAPK3, EPB41L1, FOXI1, GAL3ST1, GNA13, HAS1, HNF4A, KRT8, MIA, MST1R, NTF3, RHOD, SERPINB5, TACC2, TNS1, TRPV6	20
Cell Morphology	morphology	morphology of epithelial cells	1.61E-02	ATP4A, CLCA1 (includes EG:1179), FOXI1, MST1R	4
Cell Morphology	morphology	morphology of axons	2.51E-02	APOE	1
Cell Morphology	morphology	morphology of endocytic tubules	2.51E-02	SLC34A1	1
Cell Morphology	morphology	morphology of paranodal junctions	2.51E-02	GAL3ST1	1
Cell Morphology	stratification	stratification of eukaryotic cells	3.65E-03	CDH1, TRPV6	2
Cell Morphology	collapse	collapse of cytoskeleton	5.98E-03	RHOD, SORBS2	2
Cell Morphology	collapse	collapse of actin cytoskeleton	2.51E-02	SORBS2	1
Cell Morphology	remodeling	remodeling of cytoskeleton	1.49E-02	CX3CL1, GNRHR, INS	3
Cell Morphology	permeability	permeability of plasma membrane	2.01E-02	CCL28, FGF1	2
Cell Morphology	branching	branching of breast cancer cell lines	2.51E-02	CDH1	1
Cell Morphology	cell spreading	cell spreading of kidney cells	2.51E-02	VHL	1
Cell Morphology	cell spreading	cell spreading of lung cancer cells	2.51E-02	VHL	1
Cell Morphology	contractility	contractility of myofibroblasts	2.51E-02	ACTA2	1
Cell Morphology	diameter	diameter of sensory axons	2.51E-02	KIF5A	1
Cell Morphology	length	length of cilia	2.51E-02	IFT88	1
Cell Morphology	length	length of nodes of Ranvier	2.51E-02	GAL3ST1	1
Cell Morphology	micronucleation	micronucleation of breast cancer cell lines	2.51E-02	BRCA1	1
Cell Morphology	morphogenesis	morphogenesis of hepatoma cell lines	2.51E-02	CDH1	1
Cell-To-Cell Signaling and Interaction	adhesion	adhesion of kidney cancer cell lines	6.28E-04	CDH1, VHL	2
Cell-To-Cell Signaling and Interaction	adhesion	adhesion of cells	6.65E-03	ACTA2, ALOX12, ANGPT2, AOC3, APOE, AZGP1, CCL28, CDH1, CHRD, CRISP2, CX3CL1, CXADR, ELF3, EMR1, EPHB1, FGF1, FGG, GNRHR, GPNMB, HAS1, INS, ITGAD, NLGN3, PDPK1, PIGR, PKHD1, RAPGEF1, RHOD, SERPINB5, THY1, TINAG, TRPV6, VHL	33
Cell-To-Cell Signaling and Interaction	adhesion	adhesion of fibroblasts	1.47E-02	CDH1, RAPGEF1, RHOD, THY1	4
Cell-To-Cell Signaling and Interaction	adhesion	adhesion of normal cells	1.80E-02	ACTA2, ALOX12, AOC3, APOE, CCL28, CDH1, CX3CL1, FGF1, FGG, INS, ITGAD, PIGR, RAPGEF1, RHOD, SERPINB5, THY1	16
Cell-To-Cell Signaling and Interaction	adhesion	adhesion of breast cell lines	2.01E-02	CDH1, ELF3	2
Cell-To-Cell Signaling and Interaction	adhesion	adhesion of Th2 cells	2.51E-02	AOC3	1
Cell-To-Cell Signaling and Interaction	adhesion	adhesion of endodermal cells	2.51E-02	SERPINB5	1
Cell-To-Cell Signaling and Interaction	adhesion	adhesion of germ cell tumor cell lines	2.51E-02	EPHB1	1
Cell-To-Cell Signaling and Interaction	adhesion	adhesion of nasopharyngeal epithelial cells	2.51E-02	PIGR	1
Cell-To-Cell Signaling and Interaction	adhesion	adhesion of neural stem cells	2.51E-02	CDH1	1
Cell-To-Cell Signaling and Interaction	binding	binding of bladder cancer cell lines	6.28E-04	CX3CL1, CXADR	2
Cell-To-Cell Signaling and Interaction	binding	binding of cell lines	7.27E-04	AOC3, AR, CX3CL1, CXADR, FGF1, GAL3ST1, GNAS, IFNB1, INS, ITGAD, KRT1, LBP, NR1H4, PHB2, RAPGEF1, SERPINB5, THY1	17
Cell-To-Cell Signaling and Interaction	binding	binding of PBMC cells	1.85E-03	CX3CL1, IFNB1	2
Cell-To-Cell Signaling and Interaction	binding	binding of eukaryotic cells	2.74E-03	ACR, AOC3, APOE, AR, CX3CL1, CXADR, FCGR3A, FGF1, GAL3ST1, GNAS, IFNB1, INS, ITGAD, KRT1, LBP, NR1H4, PHB2, PIGR, RAPGEF1, SERPINB5, ST6GALNAC6, THY1	22
Cell-To-Cell Signaling and Interaction	binding	binding of cells	2.75E-03	ACR, AOC3, APOE, AR, CX3CL1, CXADR, DEFB4 (includes EG:1673), FCGR3A, FGF1, GAL3ST1, GNAS, IFNB1, INS, ITGAD, KRT1, LBP, NR1H4, PHB2, PIGR, RAPGEF1, SERPINB5, ST6GALNAC6, THY1	23
Cell-To-Cell Signaling and Interaction	binding	binding of tumor cell lines	2.11E-02	AR, CX3CL1, CXADR, GAL3ST1, INS, NR1H4, PHB2, SERPINB5, THY1	9
Cell-To-Cell Signaling and Interaction	binding	binding of colon carcinoma cells	2.51E-02	ST6GALNAC6	1
Cell-To-Cell Signaling and Interaction	binding	binding of skeletal muscle cells	2.51E-02	INS	1
Cell-To-Cell Signaling and Interaction	signaling	signaling of tumor cell lines	6.28E-04	AR, PDCD1	2
Cell-To-Cell Signaling and Interaction	dissociation	dissociation of cell lines	1.65E-03	FGF1, GAST, MST1R	3
Cell-To-Cell Signaling and Interaction	dissociation	dissociation of epithelial cell lines	2.51E-02	GAST	1
Cell-To-Cell Signaling and Interaction	dissociation	dissociation of gastric cell lines	2.51E-02	GAST	1
Cell-To-Cell Signaling and Interaction	cell-cell contact	cell-cell contact of tumor cell lines	3.65E-03	CDH1, TRPV6	2
Cell-To-Cell Signaling and Interaction	cell-cell contact	cell-cell contact of embryonic cell lines	2.51E-02	CDH1	1
Cell-To-Cell Signaling and Interaction	cell-cell contact	cell-cell contact of embryonic stem cells	2.51E-02	CDH1	1
Cell-To-Cell Signaling and Interaction	cell-cell contact	cell-cell contact of squamous carcinoma cells	2.51E-02	CDH1	1

¬© 2000-2009 Ingenuity Systems, Inc. All rights reserved.

Category	Function	Function Annotation	P-Value	Molecules	# Molecules
Cell-To-Cell Signaling and Interaction	response	response of neurons	4.65E-03	NRTN, NTF3, PSPN	3
Cell-To-Cell Signaling and Interaction	attachment	attachment of kidney cells	5.98E-03	EPHB1, VHL	2
Cell-To-Cell Signaling and Interaction	attachment	attachment of astrocytes	2.51E-02	THY1	1
Cell-To-Cell Signaling and Interaction	attachment	attachment of lung cancer cells	2.51E-02	VHL	1
Cell-To-Cell Signaling and Interaction	fusion	fusion of trophoblast cells	5.98E-03	ASCL2, GCM1	2
Cell-To-Cell Signaling and Interaction	fusion	fusion of myotube	1.59E-02	AR, TRIM54	2
Cell-To-Cell Signaling and Interaction	fusion	fusion of normal cells	1.71E-02	AR, ASCL2, CACNA1S, GCM1, TRIM54	5
Cell-To-Cell Signaling and Interaction	quantity	quantity of intercellular junctions	1.91E-02	APOE, CDH1, NTF3	3
Cell-To-Cell Signaling and Interaction	development	development of cell-matrix contacts	2.51E-02	TNS1	1
Cell-To-Cell Signaling and Interaction	morphology	morphology of paranodal junctions	2.51E-02	GAL3ST1	1
Energy Production	gluconeogenesis	gluconeogenesis of pyruvic acid	6.28E-04	INS, PCK1	2
Energy Production	conversion	conversion of pyruvic acid	1.85E-03	PCK1, PKLR	2
Energy Production	production	production of ATP	8.81E-03	INS, PAX4	2
Skeletal and Muscular System Development and Function	volume	volume of muscle cells	6.28E-04	ANGPT2, PDPK1	2
Skeletal and Muscular System Development and Function	relaxation	relaxation of cardiomyocytes	1.65E-03	PVALB, SLN (includes EG:6588), TNNC1	3
Skeletal and Muscular System Development and Function	mass	mass of muscle	3.77E-03	AR, NTF3, PDPK1, UCN	4
Skeletal and Muscular System Development and Function	mass	mass of cardiac muscle	2.51E-02	PDPK1	1
Skeletal and Muscular System Development and Function	contraction	contraction of muscle	8.58E-03	ACTA2, ACTN3, CACNA1S, HRC, MYH7, SCN4A, SLMAP, SLN (includes EG:6588), TACR2, TNNC1, TRIM54	11
Skeletal and Muscular System Development and Function	fusion	fusion of myotube	1.59E-02	AR, TRIM54	2
Skeletal and Muscular System Development and Function	force generation	force generation	2.47E-02	AR, TNNC1	2
Skeletal and Muscular System Development and Function	force generation	force generation of extensor digitorum longus muscle	2.51E-02	AR	1
Skeletal and Muscular System Development and Function	binding	binding of skeletal muscle cells	2.51E-02	INS	1
Skeletal and Muscular System Development and Function	contractility	contractility of myofibroblasts	2.51E-02	ACTA2	1
Skeletal and Muscular System Development and Function	half-relaxation time	half-relaxation time of muscle	2.51E-02	PVALB	1
Skeletal and Muscular System Development and Function	migration	migration of cardiomyocytes	2.51E-02	FURIN	1
Tissue Morphology	volume	volume of muscle cells	6.28E-04	ANGPT2, PDPK1	2
Tissue Morphology	quantity	quantity of muscle sensory neurons	1.85E-03	NTF3, RUNX3	2
Tissue Morphology	quantity	quantity of foam cells	8.81E-03	APOE, PTGES	2
Tissue Morphology	quantity	quantity of atherosclerotic lesion	1.21E-02	APOE, PTGES	2
Tissue Morphology	quantity	quantity of cerebral cortex cells	2.01E-02	NTF3, TP73	2
Tissue Morphology	thickness	thickness of carotid artery	3.65E-03	APOE, FGF1	2
Tissue Morphology	mass	mass of muscle	3.77E-03	AR, NTF3, PDPK1, UCN	4
Tissue Morphology	mass	mass of cardiac muscle	2.51E-02	PDPK1	1
Tissue Morphology	mass	mass of inguinal tissue	2.51E-02	NR1H4	1
Tissue Morphology	density	density of blood vessel	8.06E-03	APOE, INS, SERPINB5, VHL	4
Tissue Morphology	density	density of microvessel	1.21E-02	SERPINB5, VHL	2
Tissue Morphology	density	density of collagen bundle	2.51E-02	MIA	1
Tissue Morphology	density	density of vasa vasorum	2.51E-02	APOE	1
Tissue Morphology	contraction	contraction of muscle	8.58E-03	ACTA2, ACTN3, CACNA1S, HRC, MYH7, SCN4A, SLMAP, SLN (includes EG:6588), TACR2, TNNC1, TRIM54	11
Tissue Morphology	contraction	contraction of granulation tissue	2.51E-02	ACTA2	1
Tissue Morphology	relaxation	relaxation of tissue	1.61E-02	ALOX12, CYP2B6, PVALB, SLN (includes EG:6588)	4
Tissue Morphology	area	area of atherosclerotic lesion	2.40E-02	APOE, CX3CL1, NR1H4	3
Tissue Morphology	arrangement	arrangement of collagen bundle	2.51E-02	MIA	1
Tissue Morphology	degeneration	degeneration of spiral ganglion	2.51E-02	FBXO2	1
Tissue Morphology	destruction	destruction of glomerular capillary	2.51E-02	THY1	1
Tissue Morphology	diameter	diameter of capillary vessel	2.51E-02	ANGPT2	1
Tissue Morphology	diameter	diameter of collagen bundle	2.51E-02	MIA	1
Tissue Morphology	functional integrity	functional integrity of basement membrane	2.51E-02	PLAT	1
Tissue Morphology	morphology	morphology of atherosclerotic lesion	2.51E-02	APOE	1
Tissue Morphology	morphology	morphology of mullerian duct	2.51E-02	AMH	1
Cellular Movement	cell movement	cell movement of eukaryotic cells	7.04E-04	ABCB4, ANGPT2, AOC3, APOE, AR, CCBP2, CCL14, CCL18, CCL28, CDH1, CX3CL1, DEFB4 (includes EG:1673), EFS, ELF3, EPHB1, FCGR3A, FGF1, FURIN, GAL3ST1, GNA13, GNAS, IFNB1, INS, ITGAD, MST1R, NTF3, PIGR, PLAT, PTGES, PTPRO, RUNX3, SERPINB5, THY1, VHL, VTCN1, WISP1	36

				¬© 2000-2009 Ingenuity Systems, Inc. All rights reserved.	
**Category**	**Function**	**Function Annotation**	**P-Value**	**Molecules**	**# Molecules**
Cellular Movement	cell movement	cell movement of leukocytes	1.05E-03	ABCB4, ANGPT2, AOC3, APOE, CCBP2, CCL14, CCL18, CCL28, CX3CL1, DEFB4 (includes EG:1673), EFS, FCGR3A, GAL3ST1, GNA13, GNAS, IFNB1, INS, ITGAD, PIGR, PLAT, PTGES, RUNX3, THY1, VTCN1	24
Cellular Movement	cell movement	cell movement	1.86E-03	ABCB4, ABI3, ALOX12, ANGPT2, AOC3, APOE, AR, CCBP2, CCL14, CCL18, CCL28, CDH1, CX3CL1, DEFB4 (includes EG:1673), EFS, ELF3, EPHB1, FCGR3A, FGF1, FURIN, GAL3ST1, GNA13, GNAS, IFNB1, INS, ITGAD, MST1R, NTF3, PDPK1, PIGR, PLAT, PTGES, PTPRO, RUNX3, SERPINB5, THY1, VHL, VTCN1, WISP1	39
Cellular Movement	cell movement	cell movement of normal cells	2.77E-03	ABCB4, ANGPT2, AOC3, APOE, AR, CCBP2, CCL14, CCL18, CCL28, CX3CL1, DEFB4 (includes EG:1673), EFS, FCGR3A, GAL3ST1, GNA13, GNAS, IFNB1, INS, ITGAD, MST1R, NTF3, PIGR, PLAT, PTGES, RUNX3, THY1, VHL, VTCN1	28
Cellular Movement	cell movement	cell movement of mononuclear leukocytes	4.82E-03	ANGPT2, AOC3, APOE, CCBP2, CCL14, CCL18, CCL28, CX3CL1, DEFB4 (includes EG:1673), EFS, GNA13, GNAS, IFNB1, VTCN1	14
Cellular Movement	cell movement	cell movement of lymphocytes	1.36E-02	AOC3, CCBP2, CCL18, CCL28, CX3CL1, DEFB4 (includes EG:1673), EFS, GNA13, GNAS, IFNB1, VTCN1	11
Cellular Movement	cell movement	cell movement of T lymphocytes	2.14E-02	AOC3, CCBP2, CCL18, CCL28, CX3CL1, DEFB4 (includes EG:1673), EFS, VTCN1	8
Cellular Movement	cell movement	cell movement of lung cancer cells	2.51E-02	VHL	1
Cellular Movement	cell movement	cell movement of uterine cells	2.51E-02	AR	1
Cellular Movement	homing	homing of mononuclear leukocytes	3.15E-03	ANGPT2, AOC3, CCBP2, CCL14, CCL18, CCL28, CX3CL1, DEFB4 (includes EG:1673), GNA13, GNAS, IFNB1	11
Cellular Movement	homing	homing of lymphocytes	4.03E-03	AOC3, CCBP2, CCL18, CCL28, CX3CL1, DEFB4 (includes EG:1673), GNA13, GNAS, IFNB1	9
Cellular Movement	chemotaxis	chemotaxis of mononuclear leukocytes	5.26E-03	ANGPT2, CCBP2, CCL14, CCL18, CCL28, CX3CL1, DEFB4 (includes EG:1673), GNA13, GNAS, IFNB1	10
Cellular Movement	chemotaxis	chemotaxis of lymphocytes	6.48E-03	CCBP2, CCL18, CCL28, CX3CL1, DEFB4 (includes EG:1673), GNA13, GNAS, IFNB1	8
Cellular Movement	chemotaxis	chemotaxis of natural killer cells	1.91E-02	CX3CL1, GNA13, GNAS	3
Cellular Movement	chemotaxis	chemotaxis of plasma cells	2.51E-02	CCL28	1
Cellular Movement	invasion	invasion of carcinoma cells	8.21E-03	ADAM21 (includes EG:8747), MST1R, SERPINB5	3
Cellular Movement	invasion	invasion of cancer cells	1.61E-02	ADAM21 (includes EG:8747), CDH1, MST1R, SERPINB5	4
Cellular Movement	invasion	invasion of pancreatic cancer cell lines	2.15E-02	MST1R, PLAT, S100P	3
Cellular Movement	movement	movement of cells	1.15E-02	ABI3, ALOX12, ANGPT2, AOC3, APOE, AR, CCBP2, CCL14, CCL18, CCL28, CDH1, CHN2, CHRD, CX3CL1, DAPK3, DEFB4 (includes EG:1673), EPHB1, FGF1, FURIN, GAST, GNA13, GNAS, HAS1, IFNB1, IFT88, IL10RA, ITGAD, MIA, MST1R, NRTN, NTF3, PDPK1, PIGR, PLAT, PROK1, RAPGEF1, RHOD, RUNX3, S100P, SLC16A4, THY1, TNS1, TP73	43
Cellular Movement	movement	movement of normal cells	2.12E-02	ANGPT2, AOC3, APOE, CCBP2, CCL14, CCL18, CCL28, CDH1, CX3CL1, DAPK3, DEFB4 (includes EG:1673), FGF1, FURIN, GNA13, GNAS, IFNB1, IFT88, IL10RA, ITGAD, MIA, NRTN, NTF3, PDPK1, PIGR, PLAT, PROK1, RAPGEF1, RHOD, RUNX3, S100P, THY1	31
Cellular Movement	movement	movement of eukaryotic cells	2.13E-02	ALOX12, ANGPT2, AOC3, APOE, AR, CCBP2, CCL14, CCL18, CCL28, CDH1, CHN2, CX3CL1, DAPK3, DEFB4 (includes EG:1673), EPHB1, FGF1, FURIN, GAST, GNA13, GNAS, HAS1, IFNB1, IFT88, IL10RA, ITGAD, MIA, MST1R, NRTN, NTF3, PDPK1, PIGR, PLAT, PROK1, RAPGEF1, RHOD, RUNX3, S100P, SLC16A4, THY1	39
Cellular Movement	migration	migration of cells	1.46E-02	ABI3, ALOX12, ANGPT2, AOC3, APOE, AR, CCBP2, CCL14, CCL18, CCL28, CDH1, CHN2, CHRD, CX3CL1, DAPK3, DEFB4 (includes EG:1673), EPHB1, FGF1, FURIN, GAST, GNA13, GNAS, HAS1, IFNB1, IL10RA, ITGAD, MIA, MST1R, NRTN, NTF3, PDPK1, PIGR, PLAT, PROK1, RAPGEF1, RHOD, RUNX3, S100P, SLC16A4, THY1, TNS1, TP73	42
Cellular Movement	migration	migration of fibroblasts	1.89E-02	APOE, DAPK3, FGF1, GNA13, RAPGEF1, RHOD	6
Cellular Movement	migration	migration of leukocytes	2.25E-02	ANGPT2, AOC3, APOE, CCBP2, CCL14, CCL18, CCL28, CX3CL1, DEFB4 (includes EG:1673), GNA13, GNAS, IFNB1, IL10RA, ITGAD, PIGR, RHOD, RUNX3, THY1	18
Cellular Movement	migration	migration of cardiomyocytes	2.51E-02	FURIN	1
Cellular Movement	migration	migration of endodermal cells	2.51E-02	FURIN	1
Cellular Movement	migration	migration of gastric cell lines	2.51E-02	GAST	1
Cellular Movement	migration	migration of germ cell tumor cell lines	2.51E-02	EPHB1	1
Cellular Movement	migration	migration of unspecified cell lines	2.51E-02	CDH1	1
Cellular Movement	innervation	innervation of neurons	1.59E-02	NRTN, NTF3	2
Cellular Movement	innervation	innervation of vestibular ganglion neurons	2.51E-02	NTF3	1
Cellular Movement	transmigration	transmigration of normal cells	2.21E-02	AOC3, APOE, IFNB1, ITGAD, THY1	5
Cellular Movement	dispersion	dispersion of bladder cancer cell lines	2.51E-02	FGF1	1
Cell Signaling	quantity	quantity of calcium	9.79E-04	ANGPT2, APOE, CACNA1S, CCBP2, CX3CL1, FCGR3A, FGF1, GAST, GNA13, GNAS, GNRHR, HNF4A, INS, NMUR2, NTF3, PAX4, SLC34A1, TACR2, THY1, TP73, TRHR, TRPM8	22
Cell Signaling	quantity	quantity of Ca2+	1.50E-02	ANGPT2, APOE, CCBP2, CCL28, FCGR3A, FGF1, GAST, GNA13, GNAS, INS, NMUR2, TACR2, TRHR, TRPM8	14
Cell Signaling	homeostasis	homeostasis of Ca2+	7.09E-03	APOE, CCL14, CSRP3, GPR12, PYGM, TRPM8	6
Cell Signaling	excretion	excretion of Ca2+	2.51E-02	PVALB	1
Vitamin and Mineral Metabolism	quantity	quantity of calcium	9.79E-04	ANGPT2, APOE, CACNA1S, CCBP2, CX3CL1, FCGR3A, FGF1, GAST, GNA13, GNAS, GNRHR, HNF4A, INS, NMUR2, NTF3, PAX4, SLC34A1, TACR2, THY1, TP73, TRHR, TRPM8	22
Vitamin and Mineral Metabolism	quantity	quantity of Ca2+	1.50E-02	ANGPT2, APOE, CCBP2, CCL28, FCGR3A, FGF1, GAST, GNA13, GNAS, INS, NMUR2, TACR2, TRHR, TRPM8	14
Vitamin and Mineral Metabolism	homeostasis	homeostasis of Ca2+	7.09E-03	APOE, CCL14, CSRP3, GPR12, PYGM, TRPM8	6
Vitamin and Mineral Metabolism	concentration	concentration of 1-alpha, 25-dihydroxy vitamin D3	2.51E-02	SLC34A1	1
Vitamin and Mineral Metabolism	excretion	excretion of Ca2+	2.51E-02	PVALB	1
Vitamin and Mineral Metabolism	formation	formation of 11-cis-retinal	2.51E-02	RGR	1

				¬© 2000-2009 Ingenuity Systems, Inc. All rights reserved.	
**Category**	**Function**	**Function Annotation**	**P-Value**	**Molecules**	**# Molecules**
Hematological System Development and Function	cell movement	cell movement of leukocytes	1.05E-03	ABCB4, ANGPT2, AOC3, APOE, CCBP2, CCL14, CCL18, CCL28, CX3CL1, DEFB4 (includes EG:1673), EFS, FCGR3A, GAL3ST1, GNA13, GNAS, IFNB1, INS, ITGAD, PIGR, PLAT, PTGES, RUNX3, THY1, VTCN1	24
Hematological System Development and Function	cell movement	cell movement of mononuclear leukocytes	4.82E-03	ANGPT2, AOC3, APOE, CCBP2, CCL14, CCL18, CCL28, CX3CL1, DEFB4 (includes EG:1673), EFS, GNA13, GNAS, IFNB1, VTCN1	14
Hematological System Development and Function	cell movement	cell movement of lymphocytes	1.36E-02	AOC3, CCBP2, CCL18, CCL28, CX3CL1, DEFB4 (includes EG:1673), EFS, GNA13, GNAS, IFNB1, VTCN1	11
Hematological System Development and Function	cell movement	cell movement of T lymphocytes	2.14E-02	AOC3, CCBP2, CCL18, CCL28, CX3CL1, DEFB4 (includes EG:1673), EFS, VTCN1	8
Hematological System Development and Function	binding	binding of PBMC cells	1.85E-03	CX3CL1, IFNB1	2
Hematological System Development and Function	homing	homing of mononuclear leukocytes	3.15E-03	ANGPT2, AOC3, CCBP2, CCL14, CCL18, CCL28, CX3CL1, DEFB4 (includes EG:1673), GNA13, GNAS, IFNB1	11
Hematological System Development and Function	homing	homing of lymphocytes	4.03E-03	AOC3, CCBP2, CCL18, CCL28, CX3CL1, DEFB4 (includes EG:1673), GNA13, GNAS, IFNB1	9
Hematological System Development and Function	coagulation	coagulation of blood	4.37E-03	APOE, C9, CX3CL1, EPX, GNA13, HNF4A, PLAT, PROZ	8
Hematological System Development and Function	chemotaxis	chemotaxis of mononuclear leukocytes	5.26E-03	ANGPT2, CCBP2, CCL14, CCL18, CCL28, CX3CL1, DEFB4 (includes EG:1673), GNA13, GNAS, IFNB1	10
Hematological System Development and Function	chemotaxis	chemotaxis of lymphocytes	6.48E-03	CCBP2, CCL18, CCL28, CX3CL1, DEFB4 (includes EG:1673), GNA13, GNAS, IFNB1	8
Hematological System Development and Function	chemotaxis	chemotaxis of natural killer cells	1.91E-02	CX3CL1, GNA13, GNAS	3
Hematological System Development and Function	chemotaxis	chemotaxis of plasma cells	2.51E-02	CCL28	1
Hematological System Development and Function	proliferation	proliferation of natural killer T lymphocytes	5.98E-03	APOE, FCGR3A	2
Hematological System Development and Function	quantity	quantity of foam cells	8.81E-03	APOE, PTGES	2
Hematological System Development and Function	accumulation	accumulation of antigen presenting cells	1.34E-02	APOE, CX3CL1, FGF1, RUNX3	4
Hematological System Development and Function	accumulation	accumulation of blood cells	2.27E-02	APOE, C9, CX3CL1, DSC1, FGF1, HDC, IL10RA, PLAT, RUNX3	9
Hematological System Development and Function	adhesion	adhesion of Th2 cells	2.51E-02	AOC3	1
Hematological System Development and Function	commitment	commitment of granulocyte-macrophage progenitor cells	2.51E-02	PDPK1	1
Hematological System Development and Function	development	development of invariant natural killer T cells	2.51E-02	CD1D	1
Hematological System Development and Function	displacement	displacement of blood	2.51E-02	PLAT	1
Hematological System Development and Function	ingestion	ingestion of thymocytes	2.51E-02	APOE	1
Immune Cell Trafficking	cell movement	cell movement of leukocytes	1.05E-03	ABCB4, ANGPT2, AOC3, APOE, CCBP2, CCL14, CCL18, CCL28, CX3CL1, DEFB4 (includes EG:1673), EFS, FCGR3A, GAL3ST1, GNA13, GNAS, IFNB1, INS, ITGAD, PIGR, PLAT, PTGES, RUNX3, THY1, VTCN1	24
Immune Cell Trafficking	cell movement	cell movement of mononuclear leukocytes	4.82E-03	ANGPT2, AOC3, APOE, CCBP2, CCL14, CCL18, CCL28, CX3CL1, DEFB4 (includes EG:1673), EFS, GNA13, GNAS, IFNB1, VTCN1	14
Immune Cell Trafficking	cell movement	cell movement of lymphocytes	1.36E-02	AOC3, CCBP2, CCL18, CCL28, CX3CL1, DEFB4 (includes EG:1673), EFS, GNA13, GNAS, IFNB1, VTCN1	11
Immune Cell Trafficking	cell movement	cell movement of T lymphocytes	2.14E-02	AOC3, CCBP2, CCL18, CCL28, CX3CL1, DEFB4 (includes EG:1673), EFS, VTCN1	8
Immune Cell Trafficking	homing	homing of mononuclear leukocytes	3.15E-03	ANGPT2, AOC3, CCBP2, CCL14, CCL18, CCL28, CX3CL1, DEFB4 (includes EG:1673), GNA13, GNAS, IFNB1	11
Immune Cell Trafficking	homing	homing of lymphocytes	4.03E-03	AOC3, CCBP2, CCL18, CCL28, CX3CL1, DEFB4 (includes EG:1673), GNA13, GNAS, IFNB1	9
Immune Cell Trafficking	chemotaxis	chemotaxis of mononuclear leukocytes	5.26E-03	ANGPT2, CCBP2, CCL14, CCL18, CCL28, CX3CL1, DEFB4 (includes EG:1673), GNA13, GNAS, IFNB1	10
Immune Cell Trafficking	chemotaxis	chemotaxis of lymphocytes	6.48E-03	CCBP2, CCL18, CCL28, CX3CL1, DEFB4 (includes EG:1673), GNA13, GNAS, IFNB1	8
Immune Cell Trafficking	chemotaxis	chemotaxis of natural killer cells	1.91E-02	CX3CL1, GNA13, GNAS	3
Immune Cell Trafficking	chemotaxis	chemotaxis of plasma cells	2.51E-02	CCL28	1
Immune Cell Trafficking	accumulation	accumulation of antigen presenting cells	1.34E-02	APOE, CX3CL1, FGF1, RUNX3	4
Immune Cell Trafficking	migration	migration of leukocytes	2.25E-02	ANGPT2, AOC3, APOE, CCBP2, CCL14, CCL18, CCL28, CX3CL1, DEFB4 (includes EG:1673), GNA13, GNAS, IFNB1, IL10RA, ITGAD, PIGR, RHOD, RUNX3, THY1	18
Immune Cell Trafficking	adhesion	adhesion of Th2 cells	2.51E-02	AOC3	1
Carbohydrate Metabolism	uptake	uptake of digoxin	1.18E-03	ABCB4, NR1H4, SLCO1C1	3
Carbohydrate Metabolism	production	production of D-glucose	1.69E-03	APOE, INS, NR1H4, PCK1, RETNLB (includes EG:84666)	5
Carbohydrate Metabolism	production	production of carbohydrate	1.14E-02	APOE, GAST, GNRHR, GPR35, HAS1, INS, NR1H4, PCK1, RETNLB (includes EG:84666)	9
Carbohydrate Metabolism	quantity	quantity of glycogen	1.22E-02	GNAS, INS, NR1H4, PCK1	4
Carbohydrate Metabolism	accumulation	accumulation of glycosides	1.59E-02	ABCB4, DNAJC15	2
Carbohydrate Metabolism	catabolism	catabolism of glycogen	1.59E-02	INS, PYGM	2
Carbohydrate Metabolism	absorption	absorption of digoxin	2.51E-02	ABCB4	1

				¬© 2000-2009 Ingenuity Systems, Inc. All rights reserved.	
**Category**	**Function**	**Function Annotation**	**P-Value**	**Molecules**	**# Molecules**
Carbohydrate Metabolism	binding	binding of chondroitin sulfate proteoglycan	2.51E-02	APOE	1
Carbohydrate Metabolism	conversion	conversion of glycerol	2.51E-02	PCK1	1
Carbohydrate Metabolism	metabolism	metabolism of GDP-D-mannose	2.51E-02	DPM1	1
Carbohydrate Metabolism	metabolism	metabolism of glucose-1-phosphate	2.51E-02	PGM3	1
Carbohydrate Metabolism	metabolism	metabolism of glucuronic acid	2.51E-02	UGT1A6	1
Cellular Assembly and Organization	quantity	quantity of dendrites	1.18E-03	APOE, NRTN, NTF3	3
Cellular Assembly and Organization	quantity	quantity of axons	3.25E-03	APOE, AR, KIF5A, NRTN	4
Cellular Assembly and Organization	quantity	quantity of neurites	6.26E-03	APOE, AR, KIF5A, NRTN, NTF3	5
Cellular Assembly and Organization	quantity	quantity of intercellular junctions	1.91E-02	APOE, CDH1, NTF3	3
Cellular Assembly and Organization	quantity	quantity of plasma membrane projections	2.21E-02	APOE, AR, KIF5A, MIA, NRTN, NTF3	6
Cellular Assembly and Organization	formation	formation of cilia	2.23E-03	IFT88, RAB8A, VHL	3
Cellular Assembly and Organization	formation	formation of cellular inclusion bodies	2.06E-02	AIPL1, APOE, AR, KRT8	4
Cellular Assembly and Organization	formation	formation of paranodes	2.51E-02	GAL3ST1	1
Cellular Assembly and Organization	formation	formation of sex bodies	2.51E-02	BRCA1	1
Cellular Assembly and Organization	reorganization	reorganization of chromatin	8.81E-03	BRCA1, BRDT	2
Cellular Assembly and Organization	remodeling	remodeling of cytoskeleton	1.49E-02	CX3CL1, GNRHR, INS	3
Cellular Assembly and Organization	aggregation	aggregation of filaments	2.01E-02	APOE, NUMA1	2
Cellular Assembly and Organization	permeability	permeability of plasma membrane	2.01E-02	CCL28, FGF1	2
Cellular Assembly and Organization	activation	activation of nucleus	2.51E-02	UCN	1
Cellular Assembly and Organization	cross-linkage	cross-linkage of microfilaments	2.51E-02	GAS2L1	1
Cellular Assembly and Organization	density	density of collagen bundle	2.51E-02	MIA	1
Cellular Assembly and Organization	density	density of sensory projections	2.51E-02	NTF3	1
Cellular Assembly and Organization	development	development of cell-matrix contacts	2.51E-02	TNS1	1
Cellular Assembly and Organization	development	development of photoreceptor outer segments	2.51E-02	IFT88	1
Cellular Assembly and Organization	diameter	diameter of sensory axons	2.51E-02	KIF5A	1
Cellular Assembly and Organization	disruption	disruption of spindle pole	2.51E-02	NUMA1	1
Cellular Assembly and Organization	fusion	fusion of GLUT4 vesicles	2.51E-02	INS	1
Drug Metabolism	uptake	uptake of digoxin	1.18E-03	ABCB4, NR1H4, SLCO1C1	3
Drug Metabolism	uptake	uptake of indomethacin	5.98E-03	SLC13A2, SLC22A11	2
Drug Metabolism	uptake	uptake of salicylic acid	5.98E-03	SLC13A2, SLC22A11	2
Drug Metabolism	glucuronidation	glucuronidation of thyroxine	1.21E-02	UGT1A3, UGT1A6	2
Drug Metabolism	glucuronidation	glucuronidation of coumarin	2.47E-02	UGT1A3, UGT1A6	2
Drug Metabolism	glucuronidation	glucuronidation of acetaminophen	2.51E-02	UGT1A6	1
Drug Metabolism	absorption	absorption of digoxin	2.51E-02	ABCB4	1
Drug Metabolism	absorption	absorption of paclitaxel	2.51E-02	ABCB4	1
Drug Metabolism	activation	activation of cyclophosphamide	2.51E-02	CYP2B6	1
Drug Metabolism	biosynthesis	biosynthesis of epoprostenol	2.51E-02	PTGES	1
Drug Metabolism	clearance	clearance of lithium	2.51E-02	PVALB	1
Drug Metabolism	concentration	concentration of 1-alpha, 25-dihydroxy vitamin D3	2.51E-02	SLC34A1	1
Drug Metabolism	inactivation	inactivation of cytarabine	2.51E-02	CDA	1
Drug Metabolism	metabolism	metabolism of halothane	2.51E-02	CYP2E1	1
Nervous System Development and Function	quantity	quantity of dendrites	1.18E-03	APOE, NRTN, NTF3	3
Nervous System Development and Function	quantity	quantity of muscle sensory neurons	1.85E-03	NTF3, RUNX3	2
Nervous System Development and Function	quantity	quantity of axons	3.25E-03	APOE, AR, KIF5A, NRTN	4
Nervous System Development and Function	quantity	quantity of neurites	6.26E-03	APOE, AR, KIF5A, NRTN, NTF3	5
Nervous System Development and Function	quantity	quantity of cerebral cortex cells	2.01E-02	NTF3, TP73	2
Nervous System Development and Function	response	response of neurons	4.65E-03	NRTN, NTF3, PSPN	3
Nervous System Development and Function	survival	survival of sympathetic neuron	6.89E-03	NRTN, NTF3, PSPN	3
Nervous System Development and Function	survival	survival of motor neurons	1.11E-02	AMH, NRTN, NTF3, PSPN	4
Nervous System Development and Function	survival	survival of dopaminergic neurons	2.47E-02	NRTN, PSPN	2
Nervous System Development and Function	regulation	regulation of neurons	8.81E-03	NRTN, NTF3	2
Nervous System Development and Function	innervation	innervation of neurons	1.59E-02	NRTN, NTF3	2
Nervous System Development and Function	innervation	innervation of organ	1.59E-02	NRTN, NTF3	2
Nervous System Development and Function	innervation	innervation of lacrimal gland	2.51E-02	NRTN	1
Nervous System Development and Function	innervation	innervation of myenteric plexus	2.51E-02	NRTN	1
Nervous System Development and Function	innervation	innervation of submandibular gland	2.51E-02	NRTN	1
Nervous System Development and Function	innervation	innervation of vestibular ganglion neurons	2.51E-02	NTF3	1
Nervous System Development and Function	attachment	attachment of astrocytes	2.51E-02	THY1	1
Nervous System Development and Function	cell viability	cell viability of pyramidal neurons	2.51E-02	APOE	1
Nervous System Development and Function	density	density of sensory projections	2.51E-02	NTF3	1
Nervous System Development and Function	development	development of muscle sensory neurons	2.51E-02	NTF3	1
Nervous System Development and Function	diameter	diameter of sensory axons	2.51E-02	KIF5A	1
Nervous System Development and Function	expansion	expansion of granule cell precursors	2.51E-02	IFT88	1
Nervous System Development and Function	formation	formation of paranodes	2.51E-02	GAL3ST1	1
Nervous System Development and Function	length	length of nodes of Ranvier	2.51E-02	GAL3ST1	1
Nervous System Development and Function	morphology	morphology of axons	2.51E-02	APOE	1
Nervous System Development and Function	morphology	morphology of paranodal junctions	2.51E-02	GAL3ST1	1
Cellular Compromise	degeneration	degeneration of photoreceptors	1.37E-03	AIPL1, FGF1, NTF3, PDE6B	4
Cellular Compromise	degeneration	degeneration of cells	1.50E-03	AIPL1, APOE, AR, FBXO2, FGF1, KIF5A, NRTN, NTF3, PDCD1, PDE6B, PLAT	11

				¬© 2000-2009 Ingenuity Systems, Inc. All rights reserved.	
**Category**	**Function**	**Function Annotation**	**P-Value**	**Molecules**	**# Molecules**
Cellular Compromise	degeneration	degeneration of neurons	9.37E-03	APOE, AR, FBXO2, KIF5A, NRTN, NTF3, PLAT	7
Cellular Compromise	degeneration	degeneration of keratohyalin granules	2.51E-02	TGM1	1
Cellular Compromise	degeneration	degeneration of nucleus	2.51E-02	TGM1	1
Cellular Compromise	degeneration	degeneration of retinal cone cells	2.51E-02	AIPL1	1
Cellular Compromise	degeneration	degeneration of retinal rods	2.51E-02	AIPL1	1
Cellular Compromise	degeneration	delay in initiation of degeneration of axons	2.51E-02	PLAT	1
Cellular Compromise	collapse	collapse of cytoskeleton	5.98E-03	RHOD, SORBS2	2
Cellular Compromise	collapse	collapse of actin cytoskeleton	2.51E-02	SORBS2	1
Cellular Compromise	oxidative stress response	oxidative stress response	7.47E-03	ALOX12, APOE, BRCA1, CYP2E1, VHL	5
Cellular Compromise	oxidative stress response	oxidative stress response of cells	1.47E-02	ALOX12, BRCA1, CYP2E1, VHL	4
Cellular Compromise	disruption	disruption of spindle pole	2.51E-02	NUMA1	1
Cellular Compromise	micronucleation	micronucleation of breast cancer cell lines	2.51E-02	BRCA1	1
Ophthalmic Disease	degeneration	degeneration of photoreceptors	1.37E-03	AIPL1, FGF1, NTF3, PDE6B	4
Ophthalmic Disease	degeneration	degeneration of retinal cone cells	2.51E-02	AIPL1	1
Ophthalmic Disease	degeneration	degeneration of retinal rods	2.51E-02	AIPL1	1
Ophthalmic Disease	microphthalmia	microphthalmia	1.70E-02	CLDN19, MFRP, STRA6	3
Ophthalmic Disease	autosomal recessive retinitis pigmentosa	autosomal recessive retinitis pigmentosa	2.01E-02	CNGA1, PDE6B	2
Ophthalmic Disease	atrophy	atrophy of retinal pigment epithelium	2.51E-02	APOE	1
Ophthalmic Disease	hyperpigmentation	hyperpigmentation of retinal pigment epithelium	2.51E-02	APOE	1
Ophthalmic Disease	hypopigmentation	hypopigmentation of retinal pigment epithelium	2.51E-02	APOE	1
Post-Translational Modification	glycosylation	glycosylation of protein	1.48E-03	DPM1, DPM2, GAL3ST1, TM4SF4, TUSC3	5
Post-Translational Modification	glucuronidation	glucuronidation of thyroxine	1.21E-02	UGT1A3, UGT1A6	2
Post-Translational Modification	O-glycosylation	O-glycosylation of protein	1.59E-02	DPM1, DPM2	2
Post-Translational Modification	binding	binding of protein	1.61E-02	APOE, CDA, EPHB1, GAST, NR1H4, TGM3	6
Post-Translational Modification	N-glycosylation	N-glycosylation of protein	2.15E-02	GAL3ST1, TM4SF4, TUSC3	3
Post-Translational Modification	deamidation	deamidation of glutamine	2.51E-02	TGM1	1
Post-Translational Modification	esterification	esterification of protein	2.51E-02	TGM1	1
Post-Translational Modification	farnesylation	farnesylation of protein	2.51E-02	AIPL1	1
Cardiovascular System Development and Function	relaxation	relaxation of cardiomyocytes	1.65E-03	PVALB, SLN (includes EG:6588), TNNC1	3
Cardiovascular System Development and Function	thickness	thickness of aortic valve	1.85E-03	APOE, LECT1	2
Cardiovascular System Development and Function	thickness	thickness of carotid artery	3.65E-03	APOE, FGF1	2
Cardiovascular System Development and Function	vasculogenesis	vasculogenesis of extraembryonic tissue	5.98E-03	FURIN, VHL	2
Cardiovascular System Development and Function	density	density of blood vessel	8.06E-03	APOE, INS, SERPINB5, VHL	4
Cardiovascular System Development and Function	density	density of microvessel	1.21E-02	SERPINB5, VHL	2
Cardiovascular System Development and Function	density	density of vasa vasorum	2.51E-02	APOE	1
Cardiovascular System Development and Function	development	development of vascular tissue	2.47E-02	FURIN, PDPK1	2
Cardiovascular System Development and Function	angiogenesis	angiogenesis of cardiac valve	2.51E-02	LECT1	1
Cardiovascular System Development and Function	angiogenesis	angiogenesis of granulation tissue	2.51E-02	PTGES	1
Cardiovascular System Development and Function	contraction	contraction of atrium	2.51E-02	MYL7	1
Cardiovascular System Development and Function	diameter	diameter of capillary vessel	2.51E-02	ANGPT2	1
Cardiovascular System Development and Function	formation	formation of vascular tissue	2.51E-02	ANGPT2	1
Cardiovascular System Development and Function	isovolumic relaxation	isovolumic relaxation of heart	2.51E-02	PVALB	1
Cardiovascular System Development and Function	migration	migration of cardiomyocytes	2.51E-02	FURIN	1
Cellular Function and Maintenance	relaxation	relaxation of cardiomyocytes	1.65E-03	PVALB, SLN (includes EG:6588), TNNC1	3
Cellular Function and Maintenance	homeostasis	homeostasis of Ca2+	7.09E-03	APOE, CCL14, CSRP3, GPR12, PYGM, TRPM8	6
Cellular Function and Maintenance	regulation	regulation of neurons	8.81E-03	NRTN, NTF3	2
Cellular Function and Maintenance	cytostasis	cytostasis of breast cancer cell lines	1.91E-02	BRCA1, CDH1, RHOD	3
Cellular Function and Maintenance	cytostasis	cytostasis	2.49E-02	ABCB4, AMH, BRCA1, CDH1, FGF1, HAS1, IFNA8, IFNB1, MXI1, PDPK1, RHOD, TP73	12
Cellular Function and Maintenance	ingestion	ingestion of thymocytes	2.51E-02	APOE	1
Digestive System Development and Function	proliferation	proliferation of bile duct	1.85E-03	ABCB4, NR1H4	2
Digestive System Development and Function	biliary excretion	biliary excretion of lipid	5.98E-03	ABCB4, NR1H4	2

				¬© 2000-2009 Ingenuity Systems, Inc. All rights reserved.	
**Category**	**Function**	**Function Annotation**	**P-Value**	**Molecules**	**# Molecules**
Digestive System Development and Function	contraction	contraction of gastrointestinal tract	2.51E-02	NRTN	1
Digestive System Development and Function	dissociation	dissociation of gastric cell lines	2.51E-02	GAST	1
Digestive System Development and Function	expansion	expansion of intestinal villus	2.51E-02	EFS	1
Digestive System Development and Function	innervation	innervation of submandibular gland	2.51E-02	NRTN	1
Digestive System Development and Function	migration	migration of gastric cell lines	2.51E-02	GAST	1
Genetic Disorder	hypokalemic periodic paralysis	hypokalemic periodic paralysis	1.85E-03	CACNA1S, SCN4A	2
Genetic Disorder	genetic disorder	genetic disorder of humans	2.75E-03	ABCB4, APOE, AR, C9, CHN2, FGF1, FURIN, GNAS, GRM4, HSPB8, IFNB1, IL10RA, INS, KIF5A, LGI1, NTF3, PLA2G4C, PRODH, TAAR6	19
Genetic Disorder	intrahepatic cholestasis of pregnancy	intrahepatic cholestasis of pregnancy	3.65E-03	ABCB4, SLCO1C1	2
Genetic Disorder	schizophrenia	schizophrenia of humans	8.90E-03	APOE, CHN2, FGF1, GNAS, GRM4, IL10RA, LGI1, NTF3, PLA2G4C, PRODH, TAAR6	11
Genetic Disorder	cerebrotendinous xanthomatosis	cerebrotendinous xanthomatosis	1.21E-02	APOE, HNF4A	2
Genetic Disorder	cerebrotendinous xanthomatosis	cerebrotendinous xanthomatosis of mice	2.51E-02	APOE	1
Genetic Disorder	porphyria	porphyria	1.59E-02	KRT8, UROS	2
Genetic Disorder	cystic fibrosis	cystic fibrosis	1.70E-02	ATP4A, ATP4B, INS	3
Genetic Disorder	irritable bowel syndrome	irritable bowel syndrome	1.70E-02	ALOX12, HTR3A, HTR3B	3
Genetic Disorder	autosomal recessive retinitis pigmentosa	autosomal recessive retinitis pigmentosa	2.01E-02	CNGA1, PDE6B	2
Genetic Disorder	familial Alzheimer's disease	familial Alzheimer's disease	2.01E-02	ACTA2, APOE	2
Genetic Disorder	Chuvash polycythemia	Chuvash polycythemia	2.51E-02	VHL	1
Genetic Disorder	Ehlers-Danlos Syndrome Type IV	Ehlers-Danlos Syndrome Type IV	2.51E-02	COL3A1	1
Genetic Disorder	McArdle's syndrome	McArdle's syndrome	2.51E-02	PYGM	1
Genetic Disorder	autosomal dominant partial epilepsy with auditory features	autosomal dominant partial epilepsy with auditory features	2.51E-02	LGI1	1
Genetic Disorder	congenital erythropoietic porphyria	congenital erythropoietic porphyria	2.51E-02	UROS	1
Genetic Disorder	familial Danish dementia	familial Danish dementia of humans	2.51E-02	FURIN	1
Genetic Disorder	hyperprolinemia 1	hyperprolinemia 1	2.51E-02	PRODH	1
Genetic Disorder	hypofibrinogenemia	hypofibrinogenemia	2.51E-02	FGG	1
Genetic Disorder	ichthyosis hystrix, Curth Macklin type	ichthyosis hystrix, Curth Macklin type	2.51E-02	KRT1	1
Genetic Disorder	leukocyte adhesion deficiency ii	leukocyte adhesion deficiency ii	2.51E-02	SLC35C1	1
Genetic Disorder	mild hypertriglyceridemia	mild hypertriglyceridemia of mice	2.51E-02	APOE	1
Metabolic Disease	hypokalemic periodic paralysis	hypokalemic periodic paralysis	1.85E-03	CACNA1S, SCN4A	2
Metabolic Disease	hyperglycemia	hyperglycemia of rats	3.65E-03	INS, PCK1	2
Metabolic Disease	hyperglycemia	hyperglycemia of mice	2.34E-02	APOE, INS, PCK1, RETNLB (includes EG:84666), UCN3	5
Metabolic Disease	hyperinsulinism	hyperinsulinism	1.12E-02	HDC, HNF4A, INS, PCK1, UCN3	5
Metabolic Disease	hyperinsulinism	hyperinsulinism of rodents	2.23E-02	HDC, HNF4A, PCK1, UCN3	4
Metabolic Disease	cerebrotendinous xanthomatosis	cerebrotendinous xanthomatosis	1.21E-02	APOE, HNF4A	2
Metabolic Disease	cerebrotendinous xanthomatosis	cerebrotendinous xanthomatosis of mice	2.51E-02	APOE	1
Metabolic Disease	metabolic disorder	metabolic disorder of rodents	1.23E-02	ALOX12, AOC3, APOE, CD1D, GNAS, HDC, HNF4A, INS, KRT8, NTSR1, PCK1, PDCD1, RETNLB (includes EG:84666), UCN3	14
Metabolic Disease	metabolic disorder	metabolic disorder of mice	1.96E-02	ALOX12, AOC3, APOE, CD1D, GNAS, HDC, HNF4A, INS, KRT8, PCK1, PDCD1, RETNLB (includes EG:84666), UCN3	13
Metabolic Disease	porphyria	porphyria	1.59E-02	KRT8, UROS	2
Metabolic Disease	McArdle's syndrome	McArdle's syndrome	2.51E-02	PYGM	1
Metabolic Disease	congenital erythropoietic porphyria	congenital erythropoietic porphyria	2.51E-02	UROS	1
Metabolic Disease	hyperprolinemia 1	hyperprolinemia 1	2.51E-02	PRODH	1
Metabolic Disease	hypoinsulinemia	hypoinsulinemia of rats	2.51E-02	INS	1
Metabolic Disease	mild hypertriglyceridemia	mild hypertriglyceridemia of mice	2.51E-02	APOE	1
Organ Morphology	thickness	thickness of aortic valve	1.85E-03	APOE, LECT1	2
Organ Morphology	morphology	morphology of skin	5.70E-03	DSC1, IL1F5, TGM1	3
Organ Morphology	morphology	morphology of organ	2.27E-02	AR, DSC1, FGF1, IL1F5, INS, TGM1, VHL	7
Organ Morphology	morphology	morphology of pancreatic acinus	2.51E-02	KRT8	1
Organ Morphology	contraction	contraction of atrium	2.51E-02	MYL7	1
Organ Morphology	destruction	destruction of glomerular capillary	2.51E-02	THY1	1
Organ Morphology	dysplasia	dysplasia of exocrine region of pancreas	2.51E-02	KRT8	1
Organ Morphology	hypoplasia	hypoplasia of islets of Langerhans	2.51E-02	PTPRS (includes EG:5802)	1
Organ Morphology	induction	induction of kidney	2.51E-02	DPT	1
Organ Morphology	isovolumic relaxation	isovolumic relaxation of heart	2.51E-02	PVALB	1
Organ Morphology	mass	mass of thyroid gland	2.51E-02	FGF1	1
Gastrointestinal Disease	gastroesophageal reflux	gastroesophageal reflux	2.79E-03	ATP4A, ATP4B, HTR3A, HTR3B	4
Gastrointestinal Disease	nonulcer dyspepsia	nonulcer dyspepsia	8.81E-03	ATP4A, ATP4B	2

				¬© 2000-2009 Ingenuity Systems, Inc. All rights reserved.	
**Category**	**Function**	**Function Annotation**	**P-Value**	**Molecules**	**# Molecules**
Gastrointestinal Disease	nausea	nausea	8.99E-03	ATP4A, ATP4B, HTR3A, HTR3B	4
Gastrointestinal Disease	non-erosive reflux disease	non-erosive reflux disease	1.21E-02	ATP4A, ATP4B	2
Gastrointestinal Disease	peptic disorder	peptic disorder	1.21E-02	ATP4A, ATP4B	2
Gastrointestinal Disease	erosive esophagitis	erosive esophagitis	1.59E-02	ATP4A, ATP4B	2
Gastrointestinal Disease	irritable bowel syndrome	irritable bowel syndrome	1.70E-02	ALOX12, HTR3A, HTR3B	3
Gastrointestinal Disease	apoptosis	apoptosis of gastric epithelium	2.51E-02	RUNX3	1
Gastrointestinal Disease	atrophy	atrophy of gastric parietal cells	2.51E-02	GAST	1
Gastrointestinal Disease	binding	binding of colon carcinoma cells	2.51E-02	ST6GALNAC6	1
Gastrointestinal Disease	cirrhosis	cirrhosis of liver	2.51E-02	ABCB4	1
Gastrointestinal Disease	dysfunction	dysfunction of gastrointestinal tract	2.51E-02	EPX	1
Gastrointestinal Disease	enlargement	enlargement of crypt	2.51E-02	EFS	1
Gastrointestinal Disease	metaplasia	metaplasia of intestine	2.51E-02	GAST	1
Gastrointestinal Disease	metaplasia	metaplasia of stomach	2.51E-02	ATP4A	1
Embryonic Development	stratification	stratification of eukaryotic cells	3.65E-03	CDH1, TRPV6	2
Embryonic Development	fusion	fusion of trophoblast cells	5.98E-03	ASCL2, GCM1	2
Embryonic Development	fusion	delay in initiation of fusion of definitive endoderm	2.51E-02	FURIN	1
Embryonic Development	fusion	fusion of heart tube	2.51E-02	FURIN	1
Embryonic Development	vasculogenesis	vasculogenesis of extraembryonic tissue	5.98E-03	FURIN, VHL	2
Embryonic Development	developmental process	developmental process of embryoblast	2.01E-02	SALL4, SERPINB5	2
Embryonic Development	G2/M phase transition	G2/M phase transition of trophoblast cells	2.51E-02	ASCL2	1
Embryonic Development	adhesion	adhesion of embryoblast	2.51E-02	CDH1	1
Embryonic Development	adhesion	adhesion of endodermal cells	2.51E-02	SERPINB5	1
Embryonic Development	cell-cell contact	cell-cell contact of embryonic cell lines	2.51E-02	CDH1	1
Embryonic Development	cell-cell contact	cell-cell contact of embryonic stem cells	2.51E-02	CDH1	1
Embryonic Development	expansion	expansion of granule cell precursors	2.51E-02	IFT88	1
Embryonic Development	formation	formation of S-shaped bodies	2.51E-02	TINAG	1
Embryonic Development	formation	formation of blastocele	2.51E-02	CDH1	1
Embryonic Development	formation	formation of mullerian duct	2.51E-02	AMH	1
Embryonic Development	formation	formation of trophoectoderm	2.51E-02	CDH1	1
Embryonic Development	loss	loss of ectodermal cells	2.51E-02	SERPINB5	1
Embryonic Development	migration	migration of endodermal cells	2.51E-02	FURIN	1
Embryonic Development	morphology	morphology of mullerian duct	2.51E-02	AMH	1
Endocrine System Disorders	hyperglycemia	hyperglycemia of rats	3.65E-03	INS, PCK1	2
Endocrine System Disorders	hyperglycemia	hyperglycemia of mice	2.34E-02	APOE, INS, PCK1, RETNLB (includes EG:84666), UCN3	5
Endocrine System Disorders	endocrine system disorder	endocrine system disorder of mice	6.73E-03	ALOX12, AOC3, APOE, CD1D, CUZD1, GNAS, HDC, HNF4A, INS, PCK1, PDCD1, RETNLB (includes EG:84666), UCN3	13
Endocrine System Disorders	hyperinsulinism	hyperinsulinism	1.12E-02	HDC, HNF4A, INS, PCK1, UCN3	5
Endocrine System Disorders	hyperinsulinism	hyperinsulinism of rodents	2.23E-02	HDC, HNF4A, PCK1, UCN3	4
Endocrine System Disorders	morphology	morphology of pheochromocytoma cell lines	1.59E-02	EPB41L1, GNA13	2
Endocrine System Disorders	invasion	invasion of pancreatic cancer cell lines	2.15E-02	MST1R, PLAT, S100P	3
Endocrine System Disorders	enlargement	enlargement of islets of Langerhans	2.51E-02	RETNLB (includes EG:84666)	1
Endocrine System Disorders	hypoplasia	hypoplasia of islets of Langerhans	2.51E-02	PTPRS (includes EG:5802)	1
Hepatic System Disease	intrahepatic cholestasis of pregnancy	intrahepatic cholestasis of pregnancy	3.65E-03	ABCB4, SLCO1C1	2
Hepatic System Disease	G2/M phase transition	arrest in G2/M phase transition of hepatoma cell lines	2.51E-02	ATF5	1
Hepatic System Disease	cirrhosis	cirrhosis of liver	2.51E-02	ABCB4	1
Hepatic System Disease	morphogenesis	morphogenesis of hepatoma cell lines	2.51E-02	CDH1	1
Tissue Development	stratification	stratification of eukaryotic cells	3.65E-03	CDH1, TRPV6	2
Tissue Development	vasculogenesis	vasculogenesis of extraembryonic tissue	5.98E-03	FURIN, VHL	2
Tissue Development	adhesion	adhesion of cells	6.65E-03	ACTA2, ALOX12, ANGPT2, AOC3, APOE, AZGP1, CCL28, CDH1, CHRD, CRISP2, CX3CL1, CXADR, ELF3, EMR1, EPHB1, FGF1, FGG, GNRHR, GPNMB, HAS1, INS, ITGAD, NLGN3, PDPK1, PIGR, PKHD1, RAPGEF1, RHOD, SERPINB5, THY1, TINAG, TRPV6, VHL	33
Tissue Development	adhesion	adhesion of fibroblasts	1.47E-02	CDH1, RAPGEF1, RHOD, THY1	4
Tissue Development	adhesion	adhesion of Th2 cells	2.51E-02	AOC3	1
Tissue Development	adhesion	adhesion of embryoblast	2.51E-02	CDH1	1
Tissue Development	adhesion	adhesion of endodermal cells	2.51E-02	SERPINB5	1
Tissue Development	adhesion	adhesion of nasopharyngeal epithelial cells	2.51E-02	PIGR	1
Tissue Development	accumulation	accumulation of antigen presenting cells	1.34E-02	APOE, CX3CL1, FGF1, RUNX3	4
Tissue Development	accumulation	accumulation of cells	1.44E-02	APOE, AR, C9, CCL28, CX3CL1, DSC1, FGF1, HDC, IL10RA, NTF3, PLAT, RUNX3	12
Tissue Development	accumulation	accumulation of blood cells	2.27E-02	APOE, C9, CX3CL1, DSC1, FGF1, HDC, IL10RA, PLAT, RUNX3	9
Tissue Development	deposition	deposition of extracellular matrix	2.01E-02	PLAT, SERPINB5	2
Tissue Development	developmental process	developmental process of embryoblast	2.01E-02	SALL4, SERPINB5	2
Tissue Development	development	development of vascular tissue	2.47E-02	FURIN, PDPK1	2
Tissue Development	development	arrest in development of mammary gland	2.51E-02	ELF5	1
Tissue Development	angiogenesis	angiogenesis of granulation tissue	2.51E-02	PTGES	1
Tissue Development	formation	formation of S-shaped bodies	2.51E-02	TINAG	1
Tissue Development	formation	formation of blastocele	2.51E-02	CDH1	1
Tissue Development	formation	formation of mullerian duct	2.51E-02	AMH	1
Tissue Development	formation	formation of trophoectoderm	2.51E-02	CDH1	1

				¬© 2000-2009 Ingenuity Systems, Inc. All rights reserved.	
**Category**	**Function**	**Function Annotation**	**P-Value**	**Molecules**	**# Molecules**
Tissue Development	formation	formation of vascular tissue	2.51E-02	ANGPT2	1
Tissue Development	glyceroneogenesis	glyceroneogenesis of adipose tissue	2.51E-02	PCK1	1
Tissue Development	innervation	innervation of myenteric plexus	2.51E-02	NRTN	1
Cell Death	survival	survival of prostate cancer cell lines	3.72E-03	ALOX12, AR, TRPM8	3
Cell Death	survival	survival of sympathetic neuron	6.89E-03	NRTN, NTF3, PSPN	3
Cell Death	survival	survival of motor neurons	1.11E-02	AMH, NRTN, NTF3, PSPN	4
Cell Death	survival	survival of dopaminergic neurons	2.47E-02	NRTN, PSPN	2
Cell Death	lysis	lysis of eukaryotic cells	7.44E-03	C9, CD1D, CX3CL1, FCGR3A, FURIN, HNF4A, THY1	7
Cell Death	lysis	lysis of cells	8.83E-03	C9, CD1D, CX3CL1, FCGR3A, FURIN, HNF4A, NCR1, THY1	8
Cell Death	cell viability	cell viability of prostate cancer cell lines	1.13E-02	AR, BRCA1, SERPINB5	3
Cell Death	cell viability	cell viability of pyramidal neurons	2.51E-02	APOE	1
Cell Death	cell death	cell death of epithelial cells	1.79E-02	AMH, CD1D, CDH1, EPX, FGF1, KRT8, MIA, PLAT, RUNX3, SERPINB5	10
Cell Death	cell death	cell death of bone marrow-derived macrophages	2.47E-02	CD5L, IFNB1	2
Cell Death	apoptosis	apoptosis of fibroblastoids	2.51E-02	CDH1	1
Cell Death	apoptosis	apoptosis of gastric epithelium	2.51E-02	RUNX3	1
Cell Death	apoptosis	delay in initiation of apoptosis of Purkinje cells	2.51E-02	PLAT	1
Cell Death	apoptosis	delay in initiation of apoptosis of granule cells	2.51E-02	PLAT	1
Cell Death	apoptosis	delay in initiation of apoptosis of prostate cancer cell lines	2.51E-02	ALOX12	1
Cell Death	apoptosis	delay in initiation of apoptosis of skin cancer cell lines	2.51E-02	ALOX12	1
Cell Death	cytolysis	cytolysis of bladder cancer cell lines	2.51E-02	CX3CL1	1
Cell Death	cytolysis	cytolysis of mesangial cells	2.51E-02	THY1	1
Cell Death	loss	loss of ectodermal cells	2.51E-02	SERPINB5	1
Cell Death	loss	loss of striatonigral neurons	2.51E-02	NRTN	1
Cellular Growth and Proliferation	growth	growth of bladder cancer cell lines	3.72E-03	CDH1, CXADR, TP73	3
Cellular Growth and Proliferation	growth	growth of exocrine cells	1.21E-02	AR, FGF1	2
Cellular Growth and Proliferation	growth	growth of chondrocytes	2.01E-02	FGF1, INS	2
Cellular Growth and Proliferation	growth	arrest in growth of kidney cancer cell lines	2.51E-02	VHL	1
Cellular Growth and Proliferation	growth	growth of fibroblastoids	2.51E-02	CDH1	1
Cellular Growth and Proliferation	proliferation	proliferation of natural killer T lymphocytes	5.98E-03	APOE, FCGR3A	2
Cellular Growth and Proliferation	proliferation	proliferation of cells	1.51E-02	ALOX12, AMH, ANGPT2, APOE, AR, ASAH2, ASCL2, ATF5, BRCA1, CCL14, CD1D, CDH1, CHN2, CHRD, CHRNA10, CSH2, CXADR, CYP11B2, EFS, ELF3, ELF5, ENPEP, FBXO2, FCGR3A, FGF1, FRK, FURIN, GAST, GNRHR, GPNMB, H19, HNF4A, HSPB8, IFNB1, IFT88, IL10RA, INS, INSL4, ITGAD, KCNN4, KRT8, LACRT, LGI1, MIA, MMP11, MST1R, MXI1, NTF3, PAX4, PDCD1, PDPK1, PFTK1, PIGR, PLAT, PRKACG, PROK1, PTGES, PTPRO, RUNX3, S100P, SALL4, SERPINB5, SLC7A11, SSTR5, STS, SULT2A1, TACC2, THY1, TIMD4, TM4SF4, TP73, UCN, VHL, VTCN1	74
Cellular Growth and Proliferation	quantity	quantity of tumor cell lines	8.36E-03	AMH, AR, BRCA1, IFNB1, INS, SERPINB5, VHL	7
Cellular Growth and Proliferation	colony formation	colony formation of skin cancer cell lines	2.51E-02	PTPRS (includes EG:5802)	1
Cellular Growth and Proliferation	expansion	expansion of bladder cancer cell lines	2.51E-02	CDH1	1
Cellular Growth and Proliferation	expansion	expansion of granule cell precursors	2.51E-02	IFT88	1
Cellular Growth and Proliferation	formation	formation of B cell lymphoma cells	2.51E-02	MXI1	1
Cellular Growth and Proliferation	formation	formation of primordial germ cells	2.51E-02	CDH1	1
Reproductive System Disease	survival	survival of prostate cancer cell lines	3.72E-03	ALOX12, AR, TRPM8	3
Reproductive System Disease	endometriosis	endometriosis	6.12E-03	ACTA2, AIPL1, ANGPT2, APOE, AR, BRCA1, CCL18, CCL28, CX3CL1, CXADR, GNAS, GNRHR, HSD17B1, KRT8, MMP11, MST1R, PCK1, SCGB2A1, STS, THY1	20
Reproductive System Disease	cell viability	cell viability of prostate cancer cell lines	1.13E-02	AR, BRCA1, SERPINB5	3
Reproductive System Disease	quantity	quantity of prostate cancer cell lines	1.21E-02	AR, SERPINB5	2
Reproductive System Disease	ovarian cancer	ovarian cancer	1.25E-02	AMH, APOE, AR, BRCA1, CDH1, CTNNAL1, ENPEP, H19, KRT23, LRRN4, PTGES, S100P, SCGB2A1, TSPAN4, VHL, VTCN1	16
Reproductive System Disease	tumorigenesis	tumorigenesis of prostate cancer cell lines	1.59E-02	CXADR, GNAS	2
Reproductive System Disease	cytostasis	cytostasis of breast cancer cell lines	1.91E-02	BRCA1, CDH1, RHOD	3
Reproductive System Disease	adhesion	adhesion of germ cell tumor cell lines	2.51E-02	EPHB1	1
Reproductive System Disease	apoptosis	delay in initiation of apoptosis of prostate cancer cell lines	2.51E-02	ALOX12	1
Reproductive System Disease	branching	branching of breast cancer cell lines	2.51E-02	CDH1	1
Reproductive System Disease	formation	formation of leydig cell tumor	2.51E-02	AMH	1
Reproductive System Disease	hemorrhage	hemorrhage of testis	2.51E-02	AMH	1
Reproductive System Disease	masculinization	masculinization of brain	2.51E-02	AR	1
Reproductive System Disease	micronucleation	micronucleation of breast cancer cell lines	2.51E-02	BRCA1	1
Reproductive System Disease	migration	migration of germ cell tumor cell lines	2.51E-02	EPHB1	1
Reproductive System Disease	mitosis	delay in initiation of mitosis of breast cancer cell lines	2.51E-02	BRCA1	1
Endocrine System Development and Function	glucose tolerance	glucose tolerance	4.16E-03	GNAS, HDC, HNF4A, INS, NR1H4, PCK1	6
Endocrine System Development and Function	glucose tolerance	glucose tolerance of mice	1.60E-02	GNAS, HDC, HNF4A, NR1H4, PCK1	5
Endocrine System Development and Function	differentiation	differentiation of acinar gland cells	5.98E-03	BRCA1, KRT8	2
Endocrine System Development and Function	differentiation	differentiation of endocrine cells	1.49E-02	BRCA1, FGF1, KRT8	3

¬© 2000-2009 Ingenuity Systems, Inc. All rights reserved.

Category	Function	Function Annotation	P-Value	Molecules	# Molecules
Endocrine System Development and Function	production	production of aldosterone	2.01E-02	ALOX12, ANGPT2	2
Endocrine System Development and Function	hydroxylation	hydroxylation of corticosterone	2.51E-02	CYP11B2	1
Endocrine System Development and Function	insulin sensitivity	insulin sensitivity of muscle	2.51E-02	GNAS	1
Endocrine System Development and Function	mass	mass of thyroid gland	2.51E-02	FGF1	1
Endocrine System Development and Function	morphology	morphology of pancreatic acinus	2.51E-02	KRT8	1
Hair and Skin Development and Function	development	development of epidermis	4.79E-03	CASP14, KRT1, NTF3, PTCH2, SPRR1A, SPRR1B, STS	7
Hair and Skin Development and Function	development	development of hair cuticle	2.51E-02	CDH1	1
Hair and Skin Development and Function	development	development of inner root sheath	2.51E-02	CDH1	1
Hair and Skin Development and Function	differentiation	differentiation of keratinocytes	5.28E-03	CDH1, IL20, SPRR1A, SPRR1B, TGM1, TGM3	6
Hair and Skin Development and Function	morphology	morphology of skin	5.70E-03	DSC1, IL1F5, TGM1	3
Hair and Skin Development and Function	dissociation	dissociation of epithelial cell lines	2.51E-02	GAST	1
Hair and Skin Development and Function	fragility	fragility of epidermis	2.51E-02	DSC1	1
Organ Development	development	development of epidermis	4.79E-03	CASP14, KRT1, NTF3, PTCH2, SPRR1A, SPRR1B, STS	7
Organ Development	development	arrest in development of mammary gland	2.51E-02	ELF5	1
Organ Development	development	development of hair cuticle	2.51E-02	CDH1	1
Organ Development	development	development of inner root sheath	2.51E-02	CDH1	1
Organ Development	angiogenesis	angiogenesis of cardiac valve	2.51E-02	LECT1	1
Organ Development	expansion	expansion of intestinal villus	2.51E-02	EFS	1
Organ Development	formation	formation of utricle	2.51E-02	DSC1	1
Organ Development	induction	induction of kidney	2.51E-02	DPT	1
Organ Development	innervation	innervation of lacrimal gland	2.51E-02	NRTN	1
Organ Development	innervation	innervation of submandibular gland	2.51E-02	NRTN	1
Organismal Injury and Abnormalities	pathological cyst	pathological cyst	5.71E-03	DSC1, PKHD1, PROK1, SSTR5, TNS1	5
Organismal Injury and Abnormalities	quantity	quantity of atherosclerotic lesion	1.21E-02	APOE, PTGES	2
Organismal Injury and Abnormalities	development	development of lesion	1.34E-02	APOE, EFS, IFNB1, PDCD1	4
Organismal Injury and Abnormalities	area	area of atherosclerotic lesion	2.40E-02	APOE, CX3CL1, NR1H4	3
Organismal Injury and Abnormalities	astrocytosis	astrocytosis of cell lines	2.51E-02	APOE	1
Organismal Injury and Abnormalities	fibrosis	fibrosis of exocrine region of pancreas	2.51E-02	KRT8	1
Organismal Injury and Abnormalities	formation	formation of ulcerating lesion	2.51E-02	DSC1	1
Organismal Injury and Abnormalities	microgliosis	microgliosis of cell lines	2.51E-02	APOE	1
Organismal Injury and Abnormalities	morphology	morphology of atherosclerotic lesion	2.51E-02	APOE	1
Cell-mediated Immune Response	proliferation	proliferation of natural killer T lymphocytes	5.98E-03	APOE, FCGR3A	2
Cell-mediated Immune Response	immune response	immune response of organism	1.20E-02	ACR, APOE, CCL18, CD1D, CLEC1A, COL3A1, CX3CL1, KCNN4, KRT8, LBP, LILRB3 (includes EG:11025), MST1R, PDCD1, PKLR	14
Cell-mediated Immune Response	immune response	immune response of invariant natural killer T cells	2.51E-02	CD1D	1
Cell-mediated Immune Response	immune response	immune response of natural killer T lymphocytes	2.51E-02	CD1D	1
Cell-mediated Immune Response	complement activation	complement activation	1.49E-02	C9, CFHR1, KRT1	3
Cell-mediated Immune Response	inflammatory response	inflammatory response	1.76E-02	ANGPT2, AOC3, APOE, CCBP2, CCL14, CCL18, CCL28, CX3CL1, DEFB4 (includes EG:1673), EPX, FGG, GALP, GNA13, GNAS, HNF4A, IFNB1, IL20, LBP, PIGR, PTGES, TP73	21
Cell-mediated Immune Response	chemotaxis	chemotaxis of natural killer cells	1.91E-02	CX3CL1, GNA13, GNAS	3
Cell-mediated Immune Response	cell movement	cell movement of T lymphocytes	2.14E-02	AOC3, CCBP2, CCL18, CCL28, CX3CL1, DEFB4 (includes EG:1673), EFS, VTCN1	8
Cell-mediated Immune Response	adhesion	adhesion of Th2 cells	2.51E-02	AOC3	1
Cell-mediated Immune Response	chemoattraction	chemoattraction of effector T lymphocytes	2.51E-02	DEFB4 (includes EG:1673)	1
Cell-mediated Immune Response	development	development of invariant natural killer T cells	2.51E-02	CD1D	1
Renal and Urological System Development and Function	attachment	attachment of kidney cells	5.98E-03	EPHB1, VHL	2
Renal and Urological System Development and Function	buffering	buffering of urine	2.51E-02	FOXI1	1
Renal and Urological System Development and Function	cell spreading	cell spreading of kidney cells	2.51E-02	VHL	1
Renal and Urological System Development and Function	induction	induction of kidney	2.51E-02	DPT	1
Reproductive System Development and Function	fertilization	fertilization of female germ cells	8.06E-03	ACR, ADAM21 (includes EG:8747), AR, MST1R	4
Reproductive System Development and Function	fertilization	fertilization	1.83E-02	ACR, ADAM21 (includes EG:8747), AR, MAEL, MST1R	5
Reproductive System Development and Function	fertilization	fertilization of ova	2.15E-02	ACR, ADAM21 (includes EG:8747), MST1R	3
Reproductive System Development and Function	meiosis	meiosis of germ cells	1.90E-02	AMH, AR, GNAS, MAEL	4
Reproductive System Development and Function	adhesion	adhesion of breast cell lines	2.01E-02	CDH1, ELF3	2
Reproductive System Development and Function	decidualization	decidualization of endometrial stromal cells	2.51E-02	TGM1	1

¬© 2000-2009 Ingenuity Systems, Inc. All rights reserved.

Category	Function	Function Annotation	P-Value	Molecules	# Molecules
Reproductive System Development and Function	development	arrest in development of mammary gland	2.51E-02	ELF5	1
Reproductive System Development and Function	differentiation	differentiation of Leydig precursor cells	2.51E-02	AMH	1
Reproductive System Development and Function	erection	erection of rats	2.51E-02	INS	1
Reproductive System Development and Function	formation	formation of primordial germ cells	2.51E-02	CDH1	1
Reproductive System Development and Function	premeiotic phase	arrest in premeiotic phase of spermatogenic cells	2.51E-02	AR	1
Reproductive System Development and Function	spermatogenesis	arrest in spermatogenesis of spermatocytes	2.51E-02	AR	1
Tumor Morphology	invasion	invasion of carcinoma cells	8.21E-03	ADAM21 (includes EG:8747), MST1R, SERPINB5	3
Tumor Morphology	invasion	invasion of cancer cells	1.61E-02	ADAM21 (includes EG:8747), CDH1, MST1R, SERPINB5	4
Tumor Morphology	invasion	invasion of tumor	2.40E-02	APOE, FURIN, VHL	3
Tumor Morphology	formation	formation of pathological cyst	1.30E-02	DSC1, PROK1, TNS1	3
Tumor Morphology	formation	formation of androblastoma	2.51E-02	AMH	1
Tumor Morphology	formation	formation of leydig cell tumor	2.51E-02	AMH	1
Tumor Morphology	metaplasia	metaplasia of stomach	2.51E-02	ATP4A	1
Antigen Presentation	quantity	quantity of foam cells	8.81E-03	APOE, PTGES	2
Antigen Presentation	immune response	immune response of organism	1.20E-02	ACR, APOE, CCL18, CD1D, CLEC1A, COL3A1, CX3CL1, KCNN4, KRT8, LBP, LILRB3 (includes EG:11025), MST1R, PDCD1, PKLR	14
Antigen Presentation	complement activation	complement activation	1.49E-02	C9, CFHR1, KRT1	3
Antigen Presentation	inflammatory response	inflammatory response	1.76E-02	ANGPT2, AOC3, APOE, CCBP2, CCL14, CCL18, CCL28, CX3CL1, DEFB4 (includes EG:1673), EPX, FGG, GALP, GNA13, GNAS, HNF4A, IFNB1, IL20, LBP, PIGR, PTGES, TP73	21
Cell Cycle	reorganization	reorganization of chromatin	8.81E-03	BRCA1, BRDT	2
Cell Cycle	meiosis	meiosis of germ cells	1.90E-02	AMH, AR, GNAS, MAEL	4
Cell Cycle	G0 phase	initiation of G0 phase of kidney cancer cell lines	2.51E-02	VHL	1
Cell Cycle	G1/S phase transition	G1/S phase transition of insect cell lines	2.51E-02	INS	1
Cell Cycle	G2/M phase transition	G2/M phase transition of trophoblast cells	2.51E-02	ASCL2	1
Cell Cycle	G2/M phase transition	arrest in G2/M phase transition of hepatoma cell lines	2.51E-02	ATF5	1
Cell Cycle	S phase	entry into S phase of skin cancer cell lines	2.51E-02	CDH1	1
Cell Cycle	cell cycle progression	exit from cell cycle progression of kidney cancer cell lines	2.51E-02	VHL	1
Cell Cycle	cytokinesis	cytokinesis of cardiomyocytes	2.51E-02	FGF1	1
Cell Cycle	formation	formation of sex bodies	2.51E-02	BRCA1	1
Cell Cycle	mitosis	delay in initiation of mitosis of breast cancer cell lines	2.51E-02	BRCA1	1
Cell Cycle	premeiotic phase	arrest in premeiotic phase of spermatogenic cells	2.51E-02	AR	1
DNA Replication, Recombination, and Repair	reorganization	reorganization of chromatin	8.81E-03	BRCA1, BRDT	2
DNA Replication, Recombination, and Repair	formation	formation of sex bodies	2.51E-02	BRCA1	1
Inflammatory Response	quantity	quantity of foam cells	8.81E-03	APOE, PTGES	2
Inflammatory Response	immune response	immune response of organism	1.20E-02	ACR, APOE, CCL18, CD1D, CLEC1A, COL3A1, CX3CL1, KCNN4, KRT8, LBP, LILRB3 (includes EG:11025), MST1R, PDCD1, PKLR	14
Inflammatory Response	complement activation	complement activation	1.49E-02	C9, CFHR1, KRT1	3
Inflammatory Response	inflammatory response	inflammatory response	1.76E-02	ANGPT2, AOC3, APOE, CCBP2, CCL14, CCL18, CCL28, CX3CL1, DEFB4 (includes EG:1673), EPX, FGG, GALP, GNA13, GNAS, HNF4A, IFNB1, IL20, LBP, PIGR, PTGES, TP73	21
Inflammatory Response	inflammation	inflammation of gall bladder	2.51E-02	NR1H4	1
Inflammatory Response	inflammation	inflammation of large intestine	2.51E-02	ABCB4	1
Inflammatory Response	inflammation	inflammation of liver portal space	2.51E-02	ABCB4	1
Nucleic Acid Metabolism	production	production of ATP	8.81E-03	INS, PAX4	2
Nucleic Acid Metabolism	deamination	deamination of cytidine	2.51E-02	CDA	1
Nucleic Acid Metabolism	inactivation	inactivation of cytarabine	2.51E-02	CDA	1
Nucleic Acid Metabolism	metabolism	metabolism of GDP-D-mannose	2.51E-02	DPM1	1
Psychological Disorders	schizophrenia	schizophrenia of humans	8.90E-03	APOE, CHN2, FGF1, GNAS, GRM4, IL10RA, LGI1, NTF3, PLA2G4C, PRODH, TAAR6	11
Behavior	learning	learning	1.14E-02	APOE, ASNA1, CHRD, GRM4, NLGN3, NTF3, PLAT, STS, UCN	9
Behavior	aversive behavior	aversive behavior of rodents	2.47E-02	PLAT, UCN	2
Humoral Immune Response	immune response	immune response of organism	1.20E-02	ACR, APOE, CCL18, CD1D, CLEC1A, COL3A1, CX3CL1, KCNN4, KRT8, LBP, LILRB3 (includes EG:11025), MST1R, PDCD1, PKLR	14
Humoral Immune Response	complement activation	complement activation	1.49E-02	C9, CFHR1, KRT1	3
Humoral Immune Response	inflammatory response	inflammatory response	1.76E-02	ANGPT2, AOC3, APOE, CCBP2, CCL14, CCL18, CCL28, CX3CL1, DEFB4 (includes EG:1673), EPX, FGG, GALP, GNA13, GNAS, HNF4A, IFNB1, IL20, LBP, PIGR, PTGES, TP73	21
Humoral Immune Response	chemotaxis	chemotaxis of plasma cells	2.51E-02	CCL28	1
Amino Acid Metabolism	glucuronidation	glucuronidation of thyroxine	1.21E-02	UGT1A3, UGT1A6	2
Amino Acid Metabolism	concentration	concentration of glycine	2.51E-02	SLC6A18	1
Amino Acid Metabolism	deamidation	deamidation of glutamine	2.51E-02	TGM1	1
Amino Acid Metabolism	formation	formation of GABA	2.51E-02	GAST	1
Hepatic System Development and Function	differentiation	differentiation of hepatocytes	1.21E-02	FGF1, HNF4A	2

¬© 2000-2009 Ingenuity Systems, Inc. All rights reserved.

Category	Function	Function Annotation	P-Value	Molecules	# Molecules
Infection Mechanism	replication	replication of hepatitis B virus	1.21E-02	HNF4A, IFNB1	2
Infection Mechanism	replication	replication of cytomegalovirus	1.59E-02	IFNB1, IL10RA	2
Infection Mechanism	binding	binding of adenoviridae	2.51E-02	CXADR	1
Infection Mechanism	infection	infection of Newcastle disease virus	2.51E-02	IFNB1	1
Infection Mechanism	transcription	initiation of transcription of adenoviridae	2.51E-02	BRCA1	1
Connective Tissue Development and Function	adhesion	adhesion of fibroblasts	1.47E-02	CDH1, RAPGEF1, RHOD, THY1	4
Connective Tissue Development and Function	growth	growth of chondrocytes	2.01E-02	FGF1, INS	2
Connective Tissue Development and Function	growth	growth of fibroblastoids	2.51E-02	CDH1	1
Connective Tissue Development and Function	angiogenesis	angiogenesis of granulation tissue	2.51E-02	PTGES	1
Connective Tissue Development and Function	arrangement	arrangement of collagen bundle	2.51E-02	MIA	1
Connective Tissue Development and Function	contraction	contraction of granulation tissue	2.51E-02	ACTA2	1
Connective Tissue Development and Function	decidualization	decidualization of endometrial stromal cells	2.51E-02	TGM1	1
Connective Tissue Development and Function	density	density of collagen bundle	2.51E-02	MIA	1
Connective Tissue Development and Function	diameter	diameter of collagen bundle	2.51E-02	MIA	1
Connective Tissue Development and Function	glyceroneogenesis	glyceroneogenesis of adipose tissue	2.51E-02	PCK1	1
Hematological Disease	porphyria	porphyria	1.59E-02	KRT8, UROS	2
Hematological Disease	hemorrhage	hemorrhage	2.27E-02	AMH, ANGPT2, APOE, AR, FGG, PLAT, PROZ	7
Hematological Disease	cell death	cell death of bone marrow-derived macrophages	2.47E-02	CD5L, IFNB1	2
Hematological Disease	Chuvash polycythemia	Chuvash polycythemia	2.51E-02	VHL	1
Hematological Disease	bleeding	bleeding of rats	2.51E-02	PLAT	1
Hematological Disease	congenital erythropoietic porphyria	congenital erythropoietic porphyria	2.51E-02	UROS	1
Hematological Disease	hypofibrinogenemia	hypofibrinogenemia	2.51E-02	FGG	1
Inflammatory Disease	erosive esophagitis	erosive esophagitis	1.59E-02	ATP4A, ATP4B	2
Organismal Functions	glycemic control	glycemic control of mice	1.59E-02	APOE, INS	2
Developmental Disorder	microphthalmia	microphthalmia	1.70E-02	CLDN19, MFRP, STRA6	3
Developmental Disorder	diastematomyelia	diastematomyelia of mice	2.51E-02	GCM1	1
Developmental Disorder	dysplasia	dysplasia of exocrine region of pancreas	2.51E-02	KRT8	1
Developmental Disorder	hypoplasia	hypoplasia of islets of Langerhans	2.51E-02	PTPRS (includes EG:5802)	1
Developmental Disorder	masculinization	masculinization of brain	2.51E-02	AR	1
Developmental Disorder	metaplasia	metaplasia of stomach	2.51E-02	ATP4A	1
Respiratory Disease	cystic fibrosis	cystic fibrosis	1.70E-02	ATP4A, ATP4B, INS	3
Respiratory Disease	attachment	attachment of lung cancer cells	2.51E-02	VHL	1
Respiratory Disease	cell movement	cell movement of lung cancer cells	2.51E-02	VHL	1
Respiratory Disease	cell spreading	cell spreading of lung cancer cells	2.51E-02	VHL	1
Dermatological Diseases and Conditions	regression	regression of hair follicle	2.01E-02	NRTN, NTF3	2
Dermatological Diseases and Conditions	Ehlers-Danlos Syndrome Type IV	Ehlers-Danlos Syndrome Type IV	2.51E-02	COL3A1	1
Dermatological Diseases and Conditions	S phase	entry into S phase of skin cancer cell lines	2.51E-02	CDH1	1
Dermatological Diseases and Conditions	acantholysis	acantholysis of granular layer of epidermis	2.51E-02	DSC1	1
Dermatological Diseases and Conditions	apoptosis	delay in initiation of apoptosis of skin cancer cell lines	2.51E-02	ALOX12	1
Dermatological Diseases and Conditions	colony formation	colony formation of skin cancer cell lines	2.51E-02	PTPRS (includes EG:5802)	1
Dermatological Diseases and Conditions	formation	formation of ulcerating lesion	2.51E-02	DSC1	1
Dermatological Diseases and Conditions	hyperpigmentation	hyperpigmentation of retinal pigment epithelium	2.51E-02	APOE	1
Dermatological Diseases and Conditions	hypopigmentation	hypopigmentation of retinal pigment epithelium	2.51E-02	APOE	1
Dermatological Diseases and Conditions	ichthyosis hystrix, Curth Macklin type	ichthyosis hystrix, Curth Macklin type	2.51E-02	KRT1	1
Immunological Disease	cell death	cell death of bone marrow-derived macrophages	2.47E-02	CD5L, IFNB1	2
Immunological Disease	formation	formation of B cell lymphoma cells	2.51E-02	MXI1	1
Immunological Disease	leukocyte adhesion deficiency ii	leukocyte adhesion deficiency ii	2.51E-02	SLC35C1	1
Antimicrobial Response	clearance	clearance of human herpesvirus 1	2.51E-02	CD1D	1
Auditory and Vestibular System Development and Function	formation	formation of utricle	2.51E-02	DSC1	1
Auditory and Vestibular System Development and Function	innervation	innervation of vestibular ganglion neurons	2.51E-02	NTF3	1
Connective Tissue Disorders	Ehlers-Danlos Syndrome Type IV	Ehlers-Danlos Syndrome Type IV	2.51E-02	COL3A1	1
Connective Tissue Disorders	apoptosis	apoptosis of fibroblastoids	2.51E-02	CDH1	1
Connective Tissue Disorders	degeneration	degeneration of stromal cells	2.51E-02	FBXO2	1
Hematopoiesis	commitment	commitment of granulocyte-macrophage progenitor cells	2.51E-02	PDPK1	1

				¬© 2000-2009 Ingenuity Systems, Inc. All rights reserved.	
**Category**	**Function**	**Function Annotation**	**P-Value**	**Molecules**	**# Molecules**
Hematopoiesis	development	development of invariant natural killer T cells	2.51E-02	CD1D	1
Lymphoid Tissue Structure and Development	expansion	expansion of tunica media	2.51E-02	APOE	1
Respiratory System Development and Function	adhesion	adhesion of nasopharyngeal epithelial cells	2.51E-02	PIGR	1
Skeletal and Muscular Disorders	Ehlers-Danlos Syndrome Type IV	Ehlers-Danlos Syndrome Type IV	2.51E-02	COL3A1	1
Skeletal and Muscular Disorders	ataxia	ataxia of limb	2.51E-02	RUNX3	1
