# Supplementary material for: Structural and Regulatory Characterization of the Placental Epigenome at Its Maternal Interface
Source: PLoS One. 2011 Feb 23;6(2):e14723. doi: 10.1371/journal.pone.0014723 (PMC3044138; doi:10.1371/journal.pone.0014723)
Supplement: Table S5 — IPA biological network analysis of genes over-expressed in MBC versus CVS. (0.05 MB PDF) [file pone.0014723.s005.pdf]

Highly Expressed in MBC versus CVS **Table S5**

¬© 2000-2009 Ingenuity Systems, Inc. All rights reserved.

ID	Top Functions	Molecules in Network	Score	Focus Molecules
1	Antigen Presentation, Cell-mediated Immune Response, Humoral Immune Response	C5AR1,CD2,CD3,CD8,CD48,CD52,CD247,CD3-TCR,CD8A,CST7,FCGR1A/2A/3A,FCGR2A,FGR,HCK,IL7R,ITK,LCK,LGALS3,LITAF,LTB,Nfat (family),NFkB (complex),PF4,PI3K,PIK3AP1,PRF1,PTPRC,SATB1,SELL,Sos,SRC,SYK/ZAP,TCR,TNFAIP6,VAV	46	23
2	Cell-To-Cell Signaling and Interaction, Hematological System Development and Function, Hematopoiesis	Akt,Ap1,Caspase,CX3CR1,CYTIP,ERK,ERK1/2,FCGR3B,FPR1,G alphai,IgG,IL1,IL12 (complex),IL8RB,Interferon alpha,Jnk,LDL,LTF,LYZ,Mapk,MNDA,NAMPT,NCF2,P38 MAPK,PDGF BB,Pkc(s),PLC,PROK2,Ras,RASSF5,RGS18,S100A8,SIGLEC5,STAT5a/b,TNFSF13B	32	16
3	Cancer, Cell Cycle, Immunological Disease	AMPD1,C1ORF38,CD40LG,CEPT1,CFP,DNAJC4,DNAJC21,DUB2,ERBB2,Erbb2 dimer,ETFB,FAM129A,GZMH,GZMK,HBD,HBQ1 (includes EG:3049),Hsp90,HTT,IFNA2,IL2,LRRK2,MGAT1,MYC,MYH8,MYH16,MYL4,MYL9 (includes EG:10398),NFE2,NPTX2,PABPC1,SESN1,SESN3,SOD2,SQRDL (includes EG:58472),TRIM14	22	12
4	Hematological Disease, Organismal Injury and Abnormalities, Amino Acid Metabolism	amino acids,AOC3,AQP9,B3GALT2,Ck2,CTR9,cyclic AMP,CYP4F3,DYRK2,EPB42,EPB49,FCN1,HAT1,HEBP1,HRH2,IL13,Insulin,KCNJ15,leukotriene B4,MN1,NDST1,P2RY13,PCCB,PDGF-CC,PDZK1IP1,PRKX,SLC4A1,SSH2,STARD10,STK17A,TGFB1,UCN3,UGT1A8 (includes EG:54576),UST,XK	20	11
5	Cell-mediated Immune Response, Cellular Growth and Proliferation, Hematological System Development and Function	5430435G22RIK,CCL16,CD53,CD160,CD274,CORO1A,CX3CR1,dihydrotestosterone,FFAR2,GBP1 (includes EG:14468),GZMB,IFNB1,IL6,IL10,IL15,IRF3 dimer,KLK1,KLK8,KRT1,LILRA2,NIACR2,RBP3,RPL27,RPS28,S100A12,SLC16A6,SLC25A37,SLC7A7,SLCO1A1,SORL1,SRGN,TLR3/4,TNFRSF13C,TNIP3,VTCN1	13	8
6	Genetic Disorder, Hematological Disease, Cellular Assembly and Organization	ITGA2B (includes EG:3674),SLC25A39	2	1
7	Cell-To-Cell Signaling and Interaction, Cellular Growth and Proliferation, Connective Tissue Development and Function	FGD3,FGD1/3	2	1
8	Organismal Development	GMP reductase,GMPR	2	1
9	Cell Morphology, Cellular Assembly and Organization, Cell-To-Cell Signaling and Interaction	ITGB2,Linear amide carbon-nitrogen bond hydrolase,VNN2	2	1
