# Supplementary material for: Structural and Regulatory Characterization of the Placental Epigenome at Its Maternal Interface
Source: PLoS One. 2011 Feb 23;6(2):e14723. doi: 10.1371/journal.pone.0014723 (PMC3044138; doi:10.1371/journal.pone.0014723)
Supplement: Table S6 — IPA biological pathway analysis of genes over-expressed in MBC versus CVS. (0.37 MB PDF) [file pone.0014723.s006.pdf]

## Highly Expressed in MBC versus CVS

**Table S6**

¬© 2000-2009 Ingenuity Systems, Inc. All rights reserved.

| Category | Function | Function Annotation | P-value | Molecules | # Molecules |
|---|---|---|---|---|---|
| Antigen Presentation | immune response | immune response | 1.56E-13 | AQP9, C5AR1, CD2, CD48, CD247, CD8A, CST7, CX3CR1, FCGR2A, FCGR3B, FPR1, HCK, IL7R, IL8RB, ITK, LCK, LTB, LTF, NCF2, PF4, PIK3AP1, PRF1, PTPRC, SATB1, SELL, TNFSF13B | 26 |
| Antigen Presentation | immune response | immune response of organism | 4.69E-03 | CD48, CX3CR1, FCGR2A, NCF2, PIK3AP1, TNFSF13B | 6 |
| Antigen Presentation | inflammatory response | inflammatory response | 3.83E-06 | C5AR1, FCGR2A, FGR, FPR1, HCK, IL8RB, LTF, LYZ, PF4, S100A8, S100A12, SELL, TNFAIP6 | 13 |
| Antigen Presentation | respiratory burst | respiratory burst | 2.13E-05 | FGR, FPR1, HCK, NCF2, PF4 | 5 |
| Antigen Presentation | binding | binding of antigen presenting cells | 4.00E-05 | FPR1, LCK, PF4, PTPRC | 4 |
| Antigen Presentation | binding | binding of macrophages | 4.84E-04 | FPR1, PF4, PTPRC | 3 |
| Antigen Presentation | inflammation | inflammation | 5.30E-05 | C5AR1, FCGR2A, ITK, PRF1, PTPRC, S100A8, S100A12, SELL, TNFSF13B | 9 |
| Antigen Presentation | cytotoxic T lymphocyte response | cytotoxic T lymphocyte response | 1.74E-04 | CD247, CD8A, SRGN | 3 |
| Antigen Presentation | antibacterial response | antibacterial response of organism | 7.46E-04 | CFP, FGR, HCK, LYZ | 4 |
| Antigen Presentation | recruitment | recruitment of macrophages | 1.20E-03 | C5AR1, CD8A, CX3CR1 | 3 |
| Antigen Presentation | antibody response | antibody response | 7.13E-03 | LTF, PTPRC, TNFSF13B | 3 |
| Antigen Presentation | activation | activation of antigen presenting cells | 8.53E-03 | FCGR2A, HCK, LTB, LTF | 4 |
| Antigen Presentation | detachment | detachment of macrophages | 1.10E-02 | PTPRC | 1 |
| Antigen Presentation | adhesion | adhesion of antigen presenting cells | 1.27E-02 | CD2, CD48 | 2 |
| Cell-mediated Immune Response | immune response | immune response | 1.56E-13 | AQP9, C5AR1, CD2, CD48, CD247, CD8A, CST7, CX3CR1, FCGR2A, FCGR3B, FPR1, HCK, IL7R, IL8RB, ITK, LCK, LTB, LTF, NCF2, PF4, PIK3AP1, PRF1, PTPRC, SATB1, SELL, TNFSF13B | 26 |
| Cell-mediated Immune Response | immune response | immune response of organism | 4.69E-03 | CD48, CX3CR1, FCGR2A, NCF2, PIK3AP1, TNFSF13B | 6 |
| Cell-mediated Immune Response | inflammatory response | inflammatory response | 3.83E-06 | C5AR1, FCGR2A, FGR, FPR1, HCK, IL8RB, LTF, LYZ, PF4, S100A8, S100A12, SELL, TNFAIP6 | 13 |
| Cell-mediated Immune Response | activation | activation of T lymphocytes | 6.15E-06 | CD2, CD48, CD247, CD8A, ITK, LCK, PRF1, PTPRC, SATB1 | 9 |
| Cell-mediated Immune Response | activation | activation of natural killer cells | 1.31E-03 | CD2, FCGR2A, LTF, PRF1 | 4 |
| Cell-mediated Immune Response | activation | activation of pro-T lymphocytes | 5.54E-03 | CD2 | 1 |
| Cell-mediated Immune Response | development | development of T lymphocytes | 7.20E-06 | CD247, CD8A, FCGR2A, IL7R, ITK, LCK, PTPRC, SATB1 | 8 |
| Cell-mediated Immune Response | development | development of intraepithelial T lymphocytes | 2.99E-04 | LCK, PTPRC | 2 |
| Cell-mediated Immune Response | development | development of alpha-beta T lymphocytes | 6.24E-04 | FCGR2A, LCK | 2 |
| Cell-mediated Immune Response | development | development of thymocytes | 4.89E-03 | IL7R, PTPRC, SATB1 | 3 |
| Cell-mediated Immune Response | development | development of peripheral T lymphocyte | 5.54E-03 | IL7R | 1 |
| Cell-mediated Immune Response | development | arrest in development of alpha-beta T lymphocytes | 1.10E-02 | LCK | 1 |
| Cell-mediated Immune Response | quantity | quantity of T lymphocytes | 1.70E-05 | CD247, IL7R, ITK, LCK, LTB, PABPC1, PTPRC, SATB1, TNFSF13B | 9 |
| Cell-mediated Immune Response | quantity | quantity of thymocytes | 2.39E-04 | CD247, IL7R, ITK, LCK, SATB1 | 5 |
| Cell-mediated Immune Response | quantity | quantity of natural killer cells | 1.20E-03 | LTB, PRF1, PTPRC | 3 |
| Cell-mediated Immune Response | quantity | quantity of intraepithelial T lymphocytes | 1.32E-03 | IL7R, PTPRC | 2 |
| Cell-mediated Immune Response | quantity | quantity of memory T lymphocytes | 5.39E-03 | ITK, LTB | 2 |
| Cell-mediated Immune Response | respiratory burst | respiratory burst | 2.13E-05 | FGR, FPR1, HCK, NCF2, PF4 | 5 |
| Cell-mediated Immune Response | proliferation | proliferation of T lymphocytes | 2.86E-05 | CD2, CD48, CD247, CD8A, IL7R, ITK, LCK, PTPRC, SATB1, SRGN, TNFSF13B | 11 |
| Cell-mediated Immune Response | proliferation | proliferation of memory T lymphocytes | 5.94E-03 | CD2, IL7R | 2 |
| Cell-mediated Immune Response | antibody-dependent cell-mediated cytotoxic reaction | antibody-dependent cell-mediated cytotoxic reaction of leukocyte cell lines | 3.03E-05 | CD247, FCGR2A | 2 |
| Cell-mediated Immune Response | inflammation | inflammation | 5.30E-05 | C5AR1, FCGR2A, ITK, PRF1, PTPRC, S100A8, S100A12, SELL, TNFSF13B | 9 |
| Cell-mediated Immune Response | adhesion | adhesion of T lymphocytes | 7.03E-05 | CD2, CD48, ITK, RASSF5, SELL | 5 |
| Cell-mediated Immune Response | contact growth inhibition | contact growth inhibition of T lymphocytes | 9.05E-05 | LCK, PTPRC | 2 |
| Cell-mediated Immune Response | cytotoxic T lymphocyte response | cytotoxic T lymphocyte response | 1.74E-04 | CD247, CD8A, SRGN | 3 |
| Cell-mediated Immune Response | differentiation | differentiation of gamma-delta T lymphocytes | 4.48E-04 | LCK, PTPRC | 2 |
| Cell-mediated Immune Response | differentiation | differentiation of T lymphocytes | 1.02E-03 | CD2, CD8A, IL7R, ITK, LCK, PTPRC | 6 |
| Cell-mediated Immune Response | antibacterial response | antibacterial response of organism | 7.46E-04 | CFP, FGR, HCK, LYZ | 4 |
| Cell-mediated Immune Response | cytotoxic reaction | cytotoxic reaction of natural killer cells | 8.30E-04 | CD48, FCGR2A | 2 |
| Cell-mediated Immune Response | cytotoxic reaction | cytotoxic reaction of eukaryotic cells | 2.22E-03 | CD48, FCGR2A, LCK | 3 |
| Cell-mediated Immune Response | cytotoxic reaction | cytotoxic reaction of leukemia cell lines | 1.10E-02 | LCK | 1 |
| Cell-mediated Immune Response | expansion | expansion of T lymphocytes | 1.43E-03 | C5AR1, CD2, PRF1, SRGN | 4 |
| Cell-mediated Immune Response | binding | binding of T lymphocytes | 2.81E-03 | CD2, ITK, LCK | 3 |
| Cell-mediated Immune Response | functional avidity | functional avidity of cytotoxic T cells | 5.54E-03 | CD8A | 1 |
| Cell-mediated Immune Response | immunosuppression | immunosuppression of T lymphocytes | 5.54E-03 | PF4 | 1 |
| Cell-mediated Immune Response | infiltration | infiltration of cytotoxic T cells | 5.54E-03 | PRF1 | 1 |

| | | | | ¬© 2000-2009 Ingenuity Systems, Inc. All rights reserved. | |
|---|---|---|---|---|---|
| **Category** | **Function** | **Function Annotation** | **P-value** | **Molecules** | **# Molecules** |
| Cell-mediated Immune Response | antibody response | antibody response | 7.13E-03 | LTF, PTPRC, TNFSF13B | 3 |
| Cell-mediated Immune Response | graft-vs-leukemia effect | graft-vs-leukemia effect of mice | 1.10E-02 | PRF1 | 1 |
| Cell-mediated Immune Response | influx | influx of T lymphocytes | 1.10E-02 | ITK | 1 |
| Cell-mediated Immune Response | selection | selection of T lymphocytes | 1.19E-02 | ITK, PTPRC | 2 |
| Humoral Immune Response | immune response | immune response | 1.56E-13 | AQP9, C5AR1, CD2, CD48, CD247, CD8A, CST7, CX3CR1, FCGR2A, FCGR3B, FPR1, HCK, IL7R, IL8RB, ITK, LCK, LTB, LTF, NCF2, PF4, PIK3AP1, PRF1, PTPRC, SATB1, SELL, TNFSF13B | 26 |
| Humoral Immune Response | immune response | immune response of organism | 4.69E-03 | CD48, CX3CR1, FCGR2A, NCF2, PIK3AP1, TNFSF13B | 6 |
| Humoral Immune Response | inflammatory response | inflammatory response | 3.83E-06 | C5AR1, FCGR2A, FGR, FPR1, HCK, IL8RB, LTF, LYZ, PF4, S100A8, S100A12, SELL, TNFAIP6 | 13 |
| Humoral Immune Response | respiratory burst | respiratory burst | 2.13E-05 | FGR, FPR1, HCK, NCF2, PF4 | 5 |
| Humoral Immune Response | inflammation | inflammation | 5.30E-05 | C5AR1, FCGR2A, ITK, PRF1, PTPRC, S100A8, S100A12, SELL, TNFSF13B | 9 |
| Humoral Immune Response | cytotoxic T lymphocyte response | cytotoxic T lymphocyte response | 1.74E-04 | CD247, CD8A, SRGN | 3 |
| Humoral Immune Response | proliferation | proliferation of B lymphocytes | 4.94E-04 | CD8A, HCK, IL7R, PIK3AP1, PTPRC, TNFSF13B | 6 |
| Humoral Immune Response | antibacterial response | antibacterial response of organism | 7.46E-04 | CFP, FGR, HCK, LYZ | 4 |
| Humoral Immune Response | quantity | quantity of plasma cells | 2.64E-03 | IL7R, TNFSF13B | 2 |
| Humoral Immune Response | quantity | quantity of B-1b lymphocytes | 5.54E-03 | PIK3AP1 | 1 |
| Humoral Immune Response | quantity | quantity of transitional type 3 B lymphocytes | 5.54E-03 | TNFSF13B | 1 |
| Humoral Immune Response | quantity | quantity of B lymphocytes | 1.22E-02 | FCGR2A, IL7R, PIK3AP1, TNFSF13B | 4 |
| Humoral Immune Response | quantity | quantity of B-1 lymphocytes | 1.35E-02 | IL7R, PIK3AP1 | 2 |
| Humoral Immune Response | quantity | quantity of pre-B lymphocytes | 1.60E-02 | IL7R, TNFSF13B | 2 |
| Humoral Immune Response | development | development of transitional type 1 B lymphocytes | 5.54E-03 | TNFSF13B | 1 |
| Humoral Immune Response | development | development of pre-B lymphocytes | 1.04E-02 | IL7R, TNFSF13B | 2 |
| Humoral Immune Response | differentiation | differentiation of transitional type 2 B lymphocytes | 5.54E-03 | TNFSF13B | 1 |
| Humoral Immune Response | formation | formation of memory B cells | 5.54E-03 | LTB | 1 |
| Humoral Immune Response | generation | generation of pro-B lymphocytes | 5.54E-03 | IL7R | 1 |
| Humoral Immune Response | antibody response | antibody response | 7.13E-03 | LTF, PTPRC, TNFSF13B | 3 |
| Humoral Immune Response | antibody response | antibody response of mice | 7.72E-03 | PTPRC, TNFSF13B | 2 |
| Humoral Immune Response | activation | activation of B lymphocytes | 7.99E-03 | CD8A, PTPRC, TNFSF13B | 3 |
| Humoral Immune Response | co-stimulation | co-stimulation of B lymphocytes | 1.10E-02 | TNFSF13B | 1 |
| Humoral Immune Response | migration | migration of plasma cells | 1.10E-02 | TNFSF13B | 1 |
| Inflammatory Response | immune response | immune response | 1.56E-13 | AQP9, C5AR1, CD2, CD48, CD247, CD8A, CST7, CX3CR1, FCGR2A, FCGR3B, FPR1, HCK, IL7R, IL8RB, ITK, LCK, LTB, LTF, NCF2, PF4, PIK3AP1, PRF1, PTPRC, SATB1, SELL, TNFSF13B | 26 |
| Inflammatory Response | immune response | immune response of organism | 4.69E-03 | CD48, CX3CR1, FCGR2A, NCF2, PIK3AP1, TNFSF13B | 6 |
| Inflammatory Response | binding | binding of phagocytes | 6.99E-10 | FCGR2A, FCGR3B, FGR, FPR1, HCK, PF4, PTPRC, SELL | 8 |
| Inflammatory Response | binding | binding of neutrophils | 5.84E-07 | FCGR2A, FCGR3B, FGR, HCK, SELL | 5 |
| Inflammatory Response | binding | binding of macrophages | 4.84E-04 | FPR1, PF4, PTPRC | 3 |
| Inflammatory Response | chemotaxis | chemotaxis of neutrophils | 7.27E-07 | FCGR2A, FGR, FPR1, HCK, IL8RB, PF4, S100A8 | 7 |
| Inflammatory Response | activation | activation of neutrophils | 1.03E-06 | C5AR1, FPR1, IL8RB, LTF, PF4, SELL | 6 |
| Inflammatory Response | activation | activation of phagocytes | 7.42E-06 | C5AR1, FPR1, HCK, IL8RB, LTF, PF4, SELL | 7 |
| Inflammatory Response | migration | migration of phagocytes | 1.67E-06 | FGR, FPR1, HCK, IL8RB, LGALS3, S100A8, SELL | 7 |
| Inflammatory Response | migration | migration of neutrophils | 4.43E-06 | FGR, FPR1, HCK, IL8RB, S100A8, SELL | 6 |
| Inflammatory Response | inflammatory response | inflammatory response | 3.83E-06 | C5AR1, FCGR2A, FGR, FPR1, HCK, IL8RB, LTF, LYZ, PF4, S100A8, S100A12, SELL, TNFAIP6 | 13 |
| Inflammatory Response | cell movement | cell movement of neutrophils | 7.49E-06 | FCGR2A, FGR, FPR1, HCK, IL8RB, PF4, S100A8, SELL | 8 |
| Inflammatory Response | phagocytosis | phagocytosis of blood cells | 1.53E-05 | FCGR2A, FCGR3B, FGR, HCK, PF4 | 5 |
| Inflammatory Response | phagocytosis | phagocytosis of leukocyte cell lines | 2.99E-04 | FCGR2A, FGR | 2 |
| Inflammatory Response | phagocytosis | phagocytosis of leukocytes | 1.50E-03 | FCGR2A, FCGR3B, PF4 | 3 |
| Inflammatory Response | phagocytosis | phagocytosis of gonadal cell lines | 5.54E-03 | FCGR2A | 1 |
| Inflammatory Response | respiratory burst | respiratory burst | 2.13E-05 | FGR, FPR1, HCK, NCF2, PF4 | 5 |
| Inflammatory Response | respiratory burst | respiratory burst of neutrophils | 7.22E-04 | FGR, FPR1, HCK | 3 |
| Inflammatory Response | respiratory burst | respiratory burst of macrophages | 8.30E-04 | FGR, HCK | 2 |
| Inflammatory Response | cell spreading | cell spreading of blood platelets | 2.61E-05 | FCGR2A, FGR, HCK | 3 |
| Inflammatory Response | cell spreading | cell spreading of neutrophils | 1.93E-03 | FGR, HCK | 2 |
| Inflammatory Response | shape change | shape change of neutrophils | 4.49E-05 | FGR, HCK, SELL | 3 |
| Inflammatory Response | inflammation | inflammation | 5.30E-05 | C5AR1, FCGR2A, ITK, PRF1, PTPRC, S100A8, S100A12, SELL, TNFSF13B | 9 |
| Inflammatory Response | inflammation | inflammation of endothelial cells | 9.05E-05 | S100A8, S100A12 | 2 |

| | | | | ¬© 2000-2009 Ingenuity Systems, Inc. All rights reserved. | |
|---|---|---|---|---|---|
| **Category** | **Function** | **Function Annotation** | **P-value** | **Molecules** | **# Molecules** |
| Inflammatory Response | inflammation | inflammation of organ | 2.26E-03 | C5AR1, FCGR2A, ITK, PRF1, TNFSF13B | 5 |
| Inflammatory Response | inflammation | inflammation of peritoneum | 1.10E-02 | SELL | 1 |
| Inflammatory Response | inflammation | inflammation of skin | 1.35E-02 | C5AR1, FCGR2A | 2 |
| Inflammatory Response | recruitment | recruitment of neutrophils | 7.03E-05 | C5AR1, CD8A, CX3CR1, IL8RB, LYZ | 5 |
| Inflammatory Response | recruitment | recruitment of macrophages | 1.20E-03 | C5AR1, CD8A, CX3CR1 | 3 |
| Inflammatory Response | recruitment | delay in initiation of recruitment of neutrophils | 5.54E-03 | IL8RB | 1 |
| Inflammatory Response | recruitment | initiation of recruitment of neutrophils | 5.54E-03 | C5AR1 | 1 |
| Inflammatory Response | cytotoxic T lymphocyte response | cytotoxic T lymphocyte response | 1.74E-04 | CD247, CD8A, SRGN | 3 |
| Inflammatory Response | adhesion | adhesion of neutrophils | 1.78E-04 | IL8RB, PF4, S100A8, SELL | 4 |
| Inflammatory Response | experimentally induced inflammation | experimentally induced inflammation of airway | 1.80E-04 | CD2, CD48 | 2 |
| Inflammatory Response | presence | presence of neutrophils | 1.80E-04 | FGR, HCK | 2 |
| Inflammatory Response | antibacterial response | antibacterial response of organism | 7.46E-04 | CFP, FGR, HCK, LYZ | 4 |
| Inflammatory Response | quantity | quantity of neutrophils | 9.54E-04 | C5AR1, PROK2, S100A8, SELL | 4 |
| Inflammatory Response | quantity | quantity of phagocytes | 1.92E-03 | C5AR1, LGALS3, PROK2, S100A8, SELL | 5 |
| Inflammatory Response | aggregation | aggregation of phagocytes | 3.03E-03 | LCK, SELL | 2 |
| Inflammatory Response | transmigration | transmigration of neutrophils | 4.37E-03 | FPR1, IL8RB | 2 |
| Inflammatory Response | proinflammatory response | proinflammatory response of mice | 5.54E-03 | C5AR1 | 1 |
| Inflammatory Response | antibody response | antibody response | 7.13E-03 | LTF, PTPRC, TNFSF13B | 3 |
| Inflammatory Response | desensitization | desensitization of neutrophils | 1.10E-02 | IL8RB | 1 |
| Inflammatory Response | detachment | detachment of macrophages | 1.10E-02 | PTPRC | 1 |
| Inflammatory Response | chemoattraction | chemoattraction of leukocytes | 1.35E-02 | C5AR1, PF4 | 2 |
| Cell-To-Cell Signaling and Interaction | binding | binding of blood cells | 1.65E-13 | CD2, CD48, FCGR2A, FCGR3B, FGR, FPR1, HCK, ITK, LCK, LTF, NFE2, PF4, PTPRC, SELL, SIGLEC5 | 15 |
| Cell-To-Cell Signaling and Interaction | binding | binding of leukocytes | 2.08E-12 | CD2, CD48, FCGR2A, FCGR3B, FGR, FPR1, HCK, ITK, LCK, LTF, PF4, PTPRC, SELL | 13 |
| Cell-To-Cell Signaling and Interaction | binding | binding of eukaryotic cells | 3.97E-12 | CD2, CD48, FCGR2A, FCGR3B, FGR, FPR1, HCK, IL8RB, ITK, KRT1, LCK, LGALS3, LTF, NFE2, PF4, PTPRC, SELL, SIGLEC5, SORL1 | 19 |
| Cell-To-Cell Signaling and Interaction | binding | binding of normal cells | 7.95E-12 | CD2, CD48, FCGR2A, FCGR3B, FGR, FPR1, HCK, IL8RB, ITK, LCK, LTF, NFE2, PF4, PTPRC, SELL, SIGLEC5 | 16 |
| Cell-To-Cell Signaling and Interaction | binding | binding of phagocytes | 6.99E-10 | FCGR2A, FCGR3B, FGR, FPR1, HCK, PF4, PTPRC, SELL | 8 |
| Cell-To-Cell Signaling and Interaction | binding | binding of red blood cells | 3.51E-09 | CD2, FCGR2A, FCGR3B, LTF, SIGLEC5 | 5 |
| Cell-To-Cell Signaling and Interaction | binding | binding of cell lines | 4.03E-08 | CD2, CD48, FCGR2A, IL8RB, KRT1, LCK, LGALS3, LTF, PF4, SELL, SIGLEC5, SORL1 | 12 |
| Cell-To-Cell Signaling and Interaction | binding | binding of granulocytes | 1.86E-07 | FCGR2A, FCGR3B, FGR, HCK, LTF, SELL | 6 |
| Cell-To-Cell Signaling and Interaction | binding | binding of neutrophils | 5.84E-07 | FCGR2A, FCGR3B, FGR, HCK, SELL | 5 |
| Cell-To-Cell Signaling and Interaction | binding | binding of antigen presenting cells | 4.00E-05 | FPR1, LCK, PF4, PTPRC | 4 |
| Cell-To-Cell Signaling and Interaction | binding | binding of tumor cell lines | 2.73E-04 | FCGR2A, IL8RB, LCK, LGALS3, LTF, SELL | 6 |
| Cell-To-Cell Signaling and Interaction | binding | binding of endothelial cell lines | 2.75E-04 | KRT1, LGALS3, PF4, SELL | 4 |
| Cell-To-Cell Signaling and Interaction | binding | binding of macrophages | 4.84E-04 | FPR1, PF4, PTPRC | 3 |
| Cell-To-Cell Signaling and Interaction | binding | binding of lymphocytes | 6.38E-04 | CD2, ITK, LCK, SELL | 4 |
| Cell-To-Cell Signaling and Interaction | binding | binding of gonadal cell lines | 1.72E-03 | CD48, LTF, PF4 | 3 |
| Cell-To-Cell Signaling and Interaction | binding | binding of kidney cell lines | 1.72E-03 | FCGR2A, SIGLEC5, SORL1 | 3 |
| Cell-To-Cell Signaling and Interaction | binding | binding of T lymphocytes | 2.81E-03 | CD2, ITK, LCK | 3 |
| Cell-To-Cell Signaling and Interaction | binding | binding of leukemia cell lines | 3.48E-03 | IL8RB, LCK, SELL | 3 |
| Cell-To-Cell Signaling and Interaction | binding | binding of lung cell lines | 1.10E-02 | PF4 | 1 |
| Cell-To-Cell Signaling and Interaction | binding | binding of oligodendrocyte precursor cells | 1.10E-02 | IL8RB | 1 |
| Cell-To-Cell Signaling and Interaction | activation | activation of normal cells | 4.26E-12 | C5AR1, CD2, CD48, CD247, CD8A, CX3CR1, FCGR2A, FFAR2, FPR1, HCK, IL8RB, ITK, LCK, LGALS3, LTB, LTF, PF4, PRF1, PTPRC, SATB1, SELL, TNFSF13B | 22 |
| Cell-To-Cell Signaling and Interaction | activation | activation of leukocytes | 1.34E-11 | C5AR1, CD2, CD48, CD247, CD8A, FCGR2A, FPR1, HCK, IL8RB, ITK, LCK, LTB, LTF, PF4, PRF1, PTPRC, SATB1, SELL, TNFSF13B | 19 |
| Cell-To-Cell Signaling and Interaction | activation | activation of lymphocytes | 2.84E-07 | CD2, CD48, CD247, CD8A, FCGR2A, ITK, LCK, LTF, PRF1, PTPRC, SATB1, TNFSF13B | 12 |
| Cell-To-Cell Signaling and Interaction | activation | activation of lymphatic system cells | 3.30E-07 | C5AR1, FPR1, IL8RB, LTF, PF4, PRF1, SELL | 7 |
| Cell-To-Cell Signaling and Interaction | activation | activation of neutrophils | 1.03E-06 | C5AR1, FPR1, IL8RB, LTF, PF4, SELL | 6 |
| Cell-To-Cell Signaling and Interaction | activation | activation of T lymphocytes | 6.15E-06 | CD2, CD48, CD247, CD8A, ITK, LCK, PRF1, PTPRC, SATB1 | 9 |
| Cell-To-Cell Signaling and Interaction | activation | activation of phagocytes | 7.42E-06 | C5AR1, FPR1, HCK, IL8RB, LTF, PF4, SELL | 7 |
| Cell-To-Cell Signaling and Interaction | activation | activation of leukemia cell lines | 1.18E-05 | CD247, CD8A, FCGR2A, IL8RB | 4 |
| Cell-To-Cell Signaling and Interaction | activation | activation of cell lines | 4.91E-04 | CD247, CD8A, FCGR2A, IL8RB, LGALS3 | 5 |

| | | | | ¬© 2000-2009 Ingenuity Systems, Inc. All rights reserved. | |
|---|---|---|---|---|---|
| **Category** | **Function** | **Function Annotation** | **P-value** | **Molecules** | **# Molecules** |
| Cell-To-Cell Signaling and Interaction | activation | activation of natural killer cells | 1.31E-03 | CD2, FCGR2A, LTF, PRF1 | 4 |
| Cell-To-Cell Signaling and Interaction | activation | activation of pro-T lymphocytes | 5.54E-03 | CD2 | 1 |
| Cell-To-Cell Signaling and Interaction | activation | activation of B lymphocytes | 7.99E-03 | CD8A, PTPRC, TNFSF13B | 3 |
| Cell-To-Cell Signaling and Interaction | activation | activation of antigen presenting cells | 8.53E-03 | FCGR2A, HCK, LTB, LTF | 4 |
| Cell-To-Cell Signaling and Interaction | activation | activation of adipocytes | 1.10E-02 | FFAR2 | 1 |
| Cell-To-Cell Signaling and Interaction | adhesion | adhesion of normal cells | 8.07E-11 | AMICA1, CD2, CD48, CD8A, FCGR2A, FGR, HCK, IL8RB, ITK, LGALS3, PF4, PTPRC, RASSF5, S100A8, SELL, SLC4A1 | 16 |
| Cell-To-Cell Signaling and Interaction | adhesion | adhesion of blood cells | 1.88E-10 | CD2, CD48, FCGR2A, FGR, HCK, IL8RB, ITK, LGALS3, PF4, PTPRC, RASSF5, S100A8, SELL, SLC4A1 | 14 |
| Cell-To-Cell Signaling and Interaction | adhesion | adhesion of leukocytes | 3.38E-10 | CD2, CD48, FCGR2A, FGR, HCK, IL8RB, ITK, LGALS3, PF4, PTPRC, RASSF5, S100A8, SELL | 13 |
| Cell-To-Cell Signaling and Interaction | adhesion | adhesion of eukaryotic cells | 4.25E-10 | AMICA1, CD2, CD48, CD8A, CYTIP, FCGR2A, FGR, HCK, IL8RB, ITK, LGALS3, LTF, PF4, PTPRC, RASSF5, S100A8, SELL, SLC4A1 | 18 |
| Cell-To-Cell Signaling and Interaction | adhesion | adhesion of cells | 4.53E-10 | AMICA1, C1ORF38, CD2, CD48, CD8A, CX3CR1, CYTIP, FCGR2A, FGR, HCK, IL8RB, ITK, LCK, LGALS3, LTF, PF4, PTPRC, RASSF5, S100A8, SELL, SLC4A1, TNFAIP6 | 22 |
| Cell-To-Cell Signaling and Interaction | adhesion | adhesion of granulocytes | 1.95E-07 | FGR, HCK, IL8RB, LGALS3, PF4, S100A8, SELL | 7 |
| Cell-To-Cell Signaling and Interaction | adhesion | adhesion of T lymphocytes | 7.03E-05 | CD2, CD48, ITK, RASSF5, SELL | 5 |
| Cell-To-Cell Signaling and Interaction | adhesion | adhesion of neutrophils | 1.78E-04 | IL8RB, PF4, S100A8, SELL | 4 |
| Cell-To-Cell Signaling and Interaction | adhesion | adhesion of myeloid leukemia cells | 2.99E-04 | AMICA1, SELL | 2 |
| Cell-To-Cell Signaling and Interaction | adhesion | adhesion of cell lines | 1.24E-03 | CYTIP, IL8RB, LGALS3, LTF, PF4, SELL, SLC4A1 | 7 |
| Cell-To-Cell Signaling and Interaction | adhesion | adhesion of endothelial cell lines | 6.32E-03 | IL8RB, LGALS3, PF4 | 3 |
| Cell-To-Cell Signaling and Interaction | adhesion | adhesion of endothelial cells | 9.89E-03 | AMICA1, SELL, SLC4A1 | 3 |
| Cell-To-Cell Signaling and Interaction | adhesion | initiation of adhesion of monocytes | 1.10E-02 | SELL | 1 |
| Cell-To-Cell Signaling and Interaction | adhesion | adhesion of antigen presenting cells | 1.27E-02 | CD2, CD48 | 2 |
| Cell-To-Cell Signaling and Interaction | adhesion | adhesion of leukocyte cell lines | 1.51E-02 | LTF, SELL | 2 |
| Cell-To-Cell Signaling and Interaction | phagocytosis | phagocytosis of blood cells | 1.53E-05 | FCGR2A, FCGR3B, FGR, HCK, PF4 | 5 |
| Cell-To-Cell Signaling and Interaction | phagocytosis | phagocytosis of red blood cells | 2.02E-04 | FCGR2A, FGR, HCK | 3 |
| Cell-To-Cell Signaling and Interaction | phagocytosis | phagocytosis of leukocyte cell lines | 2.99E-04 | FCGR2A, FGR | 2 |
| Cell-To-Cell Signaling and Interaction | phagocytosis | phagocytosis of monocytes | 2.99E-04 | FCGR2A, PF4 | 2 |
| Cell-To-Cell Signaling and Interaction | phagocytosis | phagocytosis of leukocytes | 1.50E-03 | FCGR2A, FCGR3B, PF4 | 3 |
| Cell-To-Cell Signaling and Interaction | phagocytosis | phagocytosis of eosinophils | 5.54E-03 | FCGR2A | 1 |
| Cell-To-Cell Signaling and Interaction | phagocytosis | phagocytosis of gonadal cell lines | 5.54E-03 | FCGR2A | 1 |
| Cell-To-Cell Signaling and Interaction | phagocytosis | phagocytosis of neutrophils | 5.94E-03 | FCGR2A, FCGR3B | 2 |
| Cell-To-Cell Signaling and Interaction | recruitment | recruitment of normal cells | 4.23E-05 | C5AR1, CD8A, CX3CR1, IL8RB, LGALS3, LYZ, SELL | 7 |
| Cell-To-Cell Signaling and Interaction | recruitment | recruitment of neutrophils | 7.03E-05 | C5AR1, CD8A, CX3CR1, IL8RB, LYZ | 5 |
| Cell-To-Cell Signaling and Interaction | recruitment | recruitment of leukocytes | 1.55E-04 | C5AR1, CD8A, CX3CR1, IL8RB, LYZ, SELL | 6 |
| Cell-To-Cell Signaling and Interaction | recruitment | recruitment of macrophages | 1.20E-03 | C5AR1, CD8A, CX3CR1 | 3 |
| Cell-To-Cell Signaling and Interaction | recruitment | delay in initiation of recruitment of neutrophils | 5.54E-03 | IL8RB | 1 |
| Cell-To-Cell Signaling and Interaction | recruitment | initiation of recruitment of neutrophils | 5.54E-03 | C5AR1 | 1 |
| Cell-To-Cell Signaling and Interaction | recruitment | recruitment of leukemia cell lines | 1.10E-02 | IL8RB | 1 |
| Cell-To-Cell Signaling and Interaction | contact growth inhibition | contact growth inhibition of T lymphocytes | 9.05E-05 | LCK, PTPRC | 2 |
| Cell-To-Cell Signaling and Interaction | stimulation | stimulation of eukaryotic cells | 1.50E-04 | C5AR1, CD2, CD8A, FGD3, PTPRC, TNFSF13B | 6 |
| Cell-To-Cell Signaling and Interaction | stimulation | stimulation of normal cells | 7.90E-04 | C5AR1, CD2, FGD3, PTPRC, TNFSF13B | 5 |
| Cell-To-Cell Signaling and Interaction | stimulation | stimulation of leukocytes | 1.31E-03 | C5AR1, CD2, PTPRC, TNFSF13B | 4 |
| Cell-To-Cell Signaling and Interaction | stimulation | stimulation of lymphocytes | 2.97E-03 | CD2, PTPRC, TNFSF13B | 3 |
| Cell-To-Cell Signaling and Interaction | response | response of cells | 2.88E-04 | C5AR1, CX3CR1, IL8RB, ITK, LTB, MNDA, NCF2, PRF1, S100A8 | 9 |
| Cell-To-Cell Signaling and Interaction | response | response of normal cells | 7.19E-03 | C5AR1, ITK, LTB, S100A8 | 4 |
| Cell-To-Cell Signaling and Interaction | detachment | detachment of leukocytes | 1.06E-03 | PTPRC, SELL | 2 |
| Cell-To-Cell Signaling and Interaction | detachment | detachment of macrophages | 1.10E-02 | PTPRC | 1 |
| Cell-To-Cell Signaling and Interaction | immunosuppression | immunosuppression of T lymphocytes | 5.54E-03 | PF4 | 1 |
| Cell-To-Cell Signaling and Interaction | co-stimulation | co-stimulation of B lymphocytes | 1.10E-02 | TNFSF13B | 1 |
| Cell-To-Cell Signaling and Interaction | co-stimulation | co-stimulation of eukaryotic cells | 1.27E-02 | LCK, TNFSF13B | 2 |
| Cell-To-Cell Signaling and Interaction | conjugation | conjugation of leukocyte cell lines | 1.10E-02 | LCK | 1 |
| Cell-To-Cell Signaling and Interaction | desensitization | desensitization of neutrophils | 1.10E-02 | IL8RB | 1 |
| Cell-To-Cell Signaling and Interaction | selection | selection of T lymphocytes | 1.19E-02 | ITK, PTPRC | 2 |
| Cell-To-Cell Signaling and Interaction | chemoattraction | chemoattraction of leukocytes | 1.35E-02 | C5AR1, PF4 | 2 |
| Hematological System Development and Function | binding | binding of leukocytes | 2.08E-12 | CD2, CD48, FCGR2A, FCGR3B, FGR, FPR1, HCK, ITK, LCK, LTF, PF4, PTPRC, SELL | 13 |

| | | | | ¬© 2000-2009 Ingenuity Systems, Inc. All rights reserved. | |
|---|---|---|---|---|---|
| **Category** | **Function** | **Function Annotation** | **P-value** | **Molecules** | **# Molecules** |
| Hematological System Development and Function | binding | binding of phagocytes | 6.99E-10 | FCGR2A, FCGR3B, FGR, FPR1, HCK, PF4, PTPRC, SELL | 8 |
| Hematological System Development and Function | binding | binding of red blood cells | 3.51E-09 | CD2, FCGR2A, FCGR3B, LTF, SIGLEC5 | 5 |
| Hematological System Development and Function | binding | binding of granulocytes | 1.86E-07 | FCGR2A, FCGR3B, FGR, HCK, LTF, SELL | 6 |
| Hematological System Development and Function | binding | binding of neutrophils | 5.84E-07 | FCGR2A, FCGR3B, FGR, HCK, SELL | 5 |
| Hematological System Development and Function | binding | binding of antigen presenting cells | 4.00E-05 | FPR1, LCK, PF4, PTPRC | 4 |
| Hematological System Development and Function | binding | binding of macrophages | 4.84E-04 | FPR1, PF4, PTPRC | 3 |
| Hematological System Development and Function | binding | binding of lymphocytes | 6.38E-04 | CD2, ITK, LCK, SELL | 4 |
| Hematological System Development and Function | binding | binding of T lymphocytes | 2.81E-03 | CD2, ITK, LCK | 3 |
| Hematological System Development and Function | quantity | quantity of blood cells | 5.59E-12 | C5AR1, CD247, CD8A, CYTIP, FCGR2A, HBD, IL7R, ITK, LCK, LGALS3, LTB, PABPC1, PF4, PIK3AP1, PRF1, PROK2, PTPRC, S100A8, SATB1, SELL, TNFSF13B | 21 |
| Hematological System Development and Function | quantity | quantity of leukocytes | 6.71E-11 | C5AR1, CD247, CD8A, CYTIP, FCGR2A, IL7R, ITK, LCK, LGALS3, LTB, PABPC1, PIK3AP1, PRF1, PROK2, PTPRC, S100A8, SATB1, SELL, TNFSF13B | 19 |
| Hematological System Development and Function | quantity | quantity of mononuclear leukocytes | 6.00E-10 | C5AR1, CD247, CYTIP, FCGR2A, IL7R, ITK, LCK, LTB, PABPC1, PIK3AP1, PRF1, PROK2, PTPRC, SATB1, SELL, TNFSF13B | 16 |
| Hematological System Development and Function | quantity | quantity of lymphocytes | 2.80E-09 | C5AR1, CD247, CYTIP, FCGR2A, IL7R, ITK, LCK, LTB, PABPC1, PIK3AP1, PRF1, PTPRC, SATB1, SELL, TNFSF13B | 15 |
| Hematological System Development and Function | quantity | quantity of T lymphocytes | 1.70E-05 | CD247, IL7R, ITK, LCK, LTB, PABPC1, PTPRC, SATB1, TNFSF13B | 9 |
| Hematological System Development and Function | quantity | quantity of granulocytes | 7.52E-05 | C5AR1, CD8A, LGALS3, PROK2, S100A8, SELL | 6 |
| Hematological System Development and Function | quantity | quantity of thymocytes | 2.39E-04 | CD247, IL7R, ITK, LCK, SATB1 | 5 |
| Hematological System Development and Function | quantity | quantity of neutrophils | 9.54E-04 | C5AR1, PROK2, S100A8, SELL | 4 |
| Hematological System Development and Function | quantity | quantity of natural killer cells | 1.20E-03 | LTB, PRF1, PTPRC | 3 |
| Hematological System Development and Function | quantity | quantity of intraepithelial T lymphocytes | 1.32E-03 | IL7R, PTPRC | 2 |
| Hematological System Development and Function | quantity | quantity of phagocytes | 1.92E-03 | C5AR1, LGALS3, PROK2, S100A8, SELL | 5 |
| Hematological System Development and Function | quantity | quantity of eosinophils | 1.96E-03 | C5AR1, CD8A, LGALS3 | 3 |
| Hematological System Development and Function | quantity | quantity of plasma cells | 2.64E-03 | IL7R, TNFSF13B | 2 |
| Hematological System Development and Function | quantity | quantity of memory T lymphocytes | 5.39E-03 | ITK, LTB | 2 |
| Hematological System Development and Function | quantity | quantity of B-1b lymphocytes | 5.54E-03 | PIK3AP1 | 1 |
| Hematological System Development and Function | quantity | quantity of transitional type 3 B lymphocytes | 5.54E-03 | TNFSF13B | 1 |
| Hematological System Development and Function | quantity | quantity of B lymphocytes | 1.22E-02 | FCGR2A, IL7R, PIK3AP1, TNFSF13B | 4 |
| Hematological System Development and Function | quantity | quantity of B-1 lymphocytes | 1.35E-02 | IL7R, PIK3AP1 | 2 |
| Hematological System Development and Function | quantity | quantity of pre-B lymphocytes | 1.60E-02 | IL7R, TNFSF13B | 2 |
| Hematological System Development and Function | activation | activation of leukocytes | 1.34E-11 | C5AR1, CD2, CD48, CD247, CD8A, FCGR2A, FPR1, HCK, IL8RB, ITK, LCK, LTB, LTF, PF4, PRF1, PTPRC, SATB1, SELL, TNFSF13B | 19 |
| Hematological System Development and Function | activation | activation of lymphocytes | 2.84E-07 | CD2, CD48, CD247, CD8A, FCGR2A, ITK, LCK, LTF, PRF1, PTPRC, SATB1, TNFSF13B | 12 |

¬© 2000-2009 Ingenuity Systems, Inc. All rights reserved.

| Category | Function | Function Annotation | P-value | Molecules | # Molecules |
|---|---|---|---|---|---|
| Hematological System Development and Function | activation | activation of neutrophils | 1.03E-06 | C5AR1, FPR1, IL8RB, LTF, PF4, SELL | 6 |
| Hematological System Development and Function | activation | activation of T lymphocytes | 6.15E-06 | CD2, CD48, CD247, CD8A, ITK, LCK, PRF1, PTPRC, SATB1 | 9 |
| Hematological System Development and Function | activation | activation of phagocytes | 7.42E-06 | C5AR1, FPR1, HCK, IL8RB, LTF, PF4, SELL | 7 |
| Hematological System Development and Function | activation | activation of natural killer cells | 1.31E-03 | CD2, FCGR2A, LTF, PRF1 | 4 |
| Hematological System Development and Function | activation | activation of pro-T lymphocytes | 5.54E-03 | CD2 | 1 |
| Hematological System Development and Function | activation | activation of B lymphocytes | 7.99E-03 | CD8A, PTPRC, TNFSF13B | 3 |
| Hematological System Development and Function | activation | activation of antigen presenting cells | 8.53E-03 | FCGR2A, HCK, LTB, LTF | 4 |
| Hematological System Development and Function | adhesion | adhesion of leukocytes | 3.38E-10 | CD2, CD48, FCGR2A, FGR, HCK, IL8RB, ITK, LGALS3, PF4, PTPRC, RASSF5, S100A8, SELL | 13 |
| Hematological System Development and Function | adhesion | adhesion of granulocytes | 1.95E-07 | FGR, HCK, IL8RB, LGALS3, PF4, S100A8, SELL | 7 |
| Hematological System Development and Function | adhesion | adhesion of T lymphocytes | 7.03E-05 | CD2, CD48, ITK, RASSF5, SELL | 5 |
| Hematological System Development and Function | adhesion | adhesion of neutrophils | 1.78E-04 | IL8RB, PF4, S100A8, SELL | 4 |
| Hematological System Development and Function | adhesion | initiation of adhesion of monocytes | 1.10E-02 | SELL | 1 |
| Hematological System Development and Function | adhesion | adhesion of antigen presenting cells | 1.27E-02 | CD2, CD48 | 2 |
| Hematological System Development and Function | adhesion | adhesion of leukocyte cell lines | 1.51E-02 | LTF, SELL | 2 |
| Hematological System Development and Function | cell movement | cell movement of granulocytes | 1.25E-08 | CD2, CD48, FCGR2A, FGR, FPR1, HCK, IL8RB, ITK, LGALS3, PF4, S100A8, SELL | 12 |
| Hematological System Development and Function | cell movement | cell movement of leukocytes | 3.15E-07 | C5AR1, CD2, CD48, FCGR2A, FGR, FPR1, HCK, IL8RB, ITK, LGALS3, PF4, PRF1, S100A8, SELL | 14 |
| Hematological System Development and Function | cell movement | cell movement of neutrophils | 7.49E-06 | FCGR2A, FGR, FPR1, HCK, IL8RB, PF4, S100A8, SELL | 8 |
| Hematological System Development and Function | cell movement | cell movement of eosinophils | 7.94E-05 | CD2, CD48, FPR1, ITK, LGALS3 | 5 |
| Hematological System Development and Function | cell movement | cell movement of mononuclear leukocytes | 2.76E-03 | FPR1, IL8RB, ITK, PF4, PRF1, SELL | 6 |
| Hematological System Development and Function | cell movement | cell movement of antigen presenting cells | 6.66E-03 | C5AR1, FPR1, PF4, SELL | 4 |
| Hematological System Development and Function | cell movement | cell movement of peripheral blood leukocytes | 7.72E-03 | FPR1, SELL | 2 |
| Hematological System Development and Function | cell movement | cell movement of monocytes | 1.53E-02 | FPR1, IL8RB, PF4 | 3 |
| Hematological System Development and Function | chemotaxis | chemotaxis of neutrophils | 7.27E-07 | FCGR2A, FGR, FPR1, HCK, IL8RB, PF4, S100A8 | 7 |
| Hematological System Development and Function | chemotaxis | chemotaxis of leukocytes | 9.02E-05 | C5AR1, FCGR2A, FGR, FPR1, HCK, IL8RB, PF4, S100A8 | 8 |
| Hematological System Development and Function | chemotaxis | chemotaxis of antigen presenting cells | 1.09E-02 | C5AR1, FPR1, PF4 | 3 |
| Hematological System Development and Function | development | development of leukocytes | 3.22E-06 | CD247, CD8A, FCGR2A, IL7R, ITK, LCK, LTB, PTPRC, SATB1, TNFSF13B | 10 |
| Hematological System Development and Function | development | development of T lymphocytes | 7.20E-06 | CD247, CD8A, FCGR2A, IL7R, ITK, LCK, PTPRC, SATB1 | 8 |
| Hematological System Development and Function | development | development of lymphocytes | 7.52E-06 | CD247, CD8A, FCGR2A, IL7R, ITK, LCK, PTPRC, SATB1, TNFSF13B | 9 |
| Hematological System Development and Function | development | development of intraepithelial T lymphocytes | 2.99E-04 | LCK, PTPRC | 2 |
| Hematological System Development and Function | development | development of alpha-beta T lymphocytes | 6.24E-04 | FCGR2A, LCK | 2 |

¬© 2000-2009 Ingenuity Systems, Inc. All rights reserved.

| Category | Function | Function Annotation | P-value | Molecules | # Molecules |
|---|---|---|---|---|---|
| Hematological System Development and Function | development | development of thymocytes | 4.89E-03 | IL7R, PTPRC, SATB1 | 3 |
| Hematological System Development and Function | development | development of transitional type 1 B lymphocytes | 5.54E-03 | TNFSF13B | 1 |
| Hematological System Development and Function | development | development of pre-B lymphocytes | 1.04E-02 | IL7R, TNFSF13B | 2 |
| Hematological System Development and Function | development | arrest in development of alpha-beta T lymphocytes | 1.10E-02 | LCK | 1 |
| Hematological System Development and Function | hematological process | hematological process | 6.72E-06 | CD2, CD247, CD8A, FCGR2A, HBD, IL7R, ITK, LCK, LTB, LTF, NFE2, PF4, PTPRC, SATB1, TNFSF13B | 15 |
| Hematological System Development and Function | proliferation | proliferation of lymphocytes | 1.10E-05 | CD2, CD48, CD247, CD8A, HCK, IL7R, ITK, LCK, PIK3AP1, PTPRC, SATB1, SRGN, TNFSF13B | 13 |
| Hematological System Development and Function | proliferation | proliferation of T lymphocytes | 2.86E-05 | CD2, CD48, CD247, CD8A, IL7R, ITK, LCK, PTPRC, SATB1, SRGN, TNFSF13B | 11 |
| Hematological System Development and Function | proliferation | proliferation of hematopoietic progenitor cells | 4.83E-04 | IL7R, NFE2, PF4, PTPRC | 4 |
| Hematological System Development and Function | proliferation | proliferation of B lymphocytes | 4.94E-04 | CD8A, HCK, IL7R, PIK3AP1, PTPRC, TNFSF13B | 6 |
| Hematological System Development and Function | proliferation | proliferation of memory T lymphocytes | 5.94E-03 | CD2, IL7R | 2 |
| Hematological System Development and Function | proliferation | proliferation of peripheral blood lymphocytes | 8.36E-03 | ITK, TNFSF13B | 2 |
| Hematological System Development and Function | proliferation | proliferation of leukocyte cell lines | 1.64E-02 | CD8A, HCK, IL7R, PTPRC | 4 |
| Hematological System Development and Function | hematopoiesis | hematopoiesis | 2.09E-05 | CD2, CD247, CD8A, FCGR2A, HBD, IL7R, ITK, LCK, LTB, LTF, PTPRC, SATB1, TNFSF13B | 13 |
| Hematological System Development and Function | cell spreading | cell spreading of blood platelets | 2.61E-05 | FCGR2A, FGR, HCK | 3 |
| Hematological System Development and Function | cell spreading | cell spreading of neutrophils | 1.93E-03 | FGR, HCK | 2 |
| Hematological System Development and Function | spherocytosis | spherocytosis of red blood cells | 3.03E-05 | EPB49, SLC4A1 | 2 |
| Hematological System Development and Function | shape change | shape change of neutrophils | 4.49E-05 | FGR, HCK, SELL | 3 |
| Hematological System Development and Function | recruitment | recruitment of neutrophils | 7.03E-05 | C5AR1, CD8A, CX3CR1, IL8RB, LYZ | 5 |
| Hematological System Development and Function | recruitment | recruitment of leukocytes | 1.55E-04 | C5AR1, CD8A, CX3CR1, IL8RB, LYZ, SELL | 6 |
| Hematological System Development and Function | recruitment | recruitment of macrophages | 1.20E-03 | C5AR1, CD8A, CX3CR1 | 3 |
| Hematological System Development and Function | recruitment | delay in initiation of recruitment of neutrophils | 5.54E-03 | IL8RB | 1 |
| Hematological System Development and Function | recruitment | initiation of recruitment of neutrophils | 5.54E-03 | C5AR1 | 1 |
| Hematological System Development and Function | maturation | maturation of thymocytes | 7.08E-05 | CD247, LCK, PTPRC | 3 |
| Hematological System Development and Function | maturation | maturation of lymphocytes | 7.48E-05 | CD247, ITK, LCK, PTPRC, TNFSF13B | 5 |
| Hematological System Development and Function | maturation | maturation of T lymphocytes | 1.09E-04 | CD247, ITK, LCK, PTPRC | 4 |
| Hematological System Development and Function | maturation | arrest in maturation of megakaryocytes | 5.54E-03 | NFE2 | 1 |
| Hematological System Development and Function | contact growth inhibition | contact growth inhibition of T lymphocytes | 9.05E-05 | LCK, PTPRC | 2 |
| Hematological System Development and Function | infiltration | infiltration of granulocytes | 1.23E-04 | CD2, CD48, IL8RB, ITK, PF4, SELL | 6 |
| Hematological System Development and Function | infiltration | infiltration of leukocytes | 4.39E-04 | CD2, CD48, IL8RB, ITK, PF4, PRF1, SELL | 7 |
| Hematological System Development and Function | infiltration | infiltration of eosinophils | 1.61E-03 | CD2, CD48, ITK | 3 |

¬© 2000-2009 Ingenuity Systems, Inc. All rights reserved.

| Category | Function | Function Annotation | P-value | Molecules | # Molecules |
|---|---|---|---|---|---|
| Hematological System Development and Function | infiltration | infiltration of mononuclear cells | 6.51E-03 | LTB, PF4 | 2 |
| Hematological System Development and Function | cell rolling | cell rolling of leukocytes | 1.78E-04 | FCGR2A, IL8RB, LGALS3, SELL | 4 |
| Hematological System Development and Function | cell rolling | cell rolling of granulocytes | 5.39E-03 | LGALS3, SELL | 2 |
| Hematological System Development and Function | cell rolling | cell rolling of naive T lymphocytes | 5.54E-03 | SELL | 1 |
| Hematological System Development and Function | cell rolling | cell rolling of mononuclear leukocytes | 5.94E-03 | IL8RB, SELL | 2 |
| Hematological System Development and Function | cell rolling | arrest in cell rolling of monocytes | 1.65E-02 | IL8RB | 1 |
| Hematological System Development and Function | presence | presence of neutrophils | 1.80E-04 | FGR, HCK | 2 |
| Hematological System Development and Function | phagocytosis | phagocytosis of red blood cells | 2.02E-04 | FCGR2A, FGR, HCK | 3 |
| Hematological System Development and Function | phagocytosis | phagocytosis of leukocyte cell lines | 2.99E-04 | FCGR2A, FGR | 2 |
| Hematological System Development and Function | phagocytosis | phagocytosis of eosinophils | 5.54E-03 | FCGR2A | 1 |
| Hematological System Development and Function | phagocytosis | phagocytosis of neutrophils | 5.94E-03 | FCGR2A, FCGR3B | 2 |
| Hematological System Development and Function | expansion | expansion of lymphocytes | 2.86E-04 | C5AR1, CD2, IL7R, PRF1, SRGN | 5 |
| Hematological System Development and Function | expansion | expansion of T lymphocytes | 1.43E-03 | C5AR1, CD2, PRF1, SRGN | 4 |
| Hematological System Development and Function | differentiation | differentiation of gamma-delta T lymphocytes | 4.48E-04 | LCK, PTPRC | 2 |
| Hematological System Development and Function | differentiation | differentiation of mononuclear leukocytes | 4.98E-04 | CD2, CD8A, IL7R, ITK, LCK, PF4, PTPRC, TNFSF13B | 8 |
| Hematological System Development and Function | differentiation | differentiation of T lymphocytes | 1.02E-03 | CD2, CD8A, IL7R, ITK, LCK, PTPRC | 6 |
| Hematological System Development and Function | differentiation | differentiation of lymphocytes | 1.22E-03 | CD2, CD8A, IL7R, ITK, LCK, PTPRC, TNFSF13B | 7 |
| Hematological System Development and Function | differentiation | differentiation of blood cells | 1.70E-03 | CD2, CD8A, IL7R, ITK, LCK, NFE2, PF4, PTPRC, TNFSF13B | 9 |
| Hematological System Development and Function | differentiation | differentiation of transitional type 2 B lymphocytes | 5.54E-03 | TNFSF13B | 1 |
| Hematological System Development and Function | differentiation | differentiation of megakaryocytes | 7.72E-03 | NFE2, PF4 | 2 |
| Hematological System Development and Function | differentiation | arrest in differentiation of cord blood cells | 1.10E-02 | PTPRC | 1 |
| Hematological System Development and Function | differentiation | arrest in differentiation of erythroblasts | 1.10E-02 | PTPRC | 1 |
| Hematological System Development and Function | detachment | detachment of leukocytes | 1.06E-03 | PTPRC, SELL | 2 |
| Hematological System Development and Function | detachment | detachment of macrophages | 1.10E-02 | PTPRC | 1 |
| Hematological System Development and Function | stimulation | stimulation of leukocytes | 1.31E-03 | C5AR1, CD2, PTPRC, TNFSF13B | 4 |
| Hematological System Development and Function | stimulation | stimulation of lymphocytes | 2.97E-03 | CD2, PTPRC, TNFSF13B | 3 |
| Hematological System Development and Function | aggregation | aggregation of granulocytes | 1.61E-03 | FPR1, SELL | 2 |
| Hematological System Development and Function | aggregation | aggregation of leukocytes | 1.84E-03 | FPR1, LCK, SELL | 3 |
| Hematological System Development and Function | aggregation | aggregation of phagocytes | 3.03E-03 | LCK, SELL | 2 |
| Hematological System Development and Function | aggregation | aggregation of blood cells | 8.94E-03 | FCGR2A, FPR1, LCK, SELL | 4 |

¬© 2000-2009 Ingenuity Systems, Inc. All rights reserved.

| Category | Function | Function Annotation | P-value | Molecules | # Molecules |
|---|---|---|---|---|---|
| Hematological System Development and Function | survival | survival of red blood cells | 1.61E-03 | NFE2, PF4 | 2 |
| Hematological System Development and Function | survival | survival of lymphocytes | 4.27E-03 | CD8A, HCK, NCF2, TNFSF13B | 4 |
| Hematological System Development and Function | survival | survival of transitional type 2 B lymphocytes | 5.54E-03 | TNFSF13B | 1 |
| Hematological System Development and Function | survival | survival of transitional type 3 B lymphocytes | 5.54E-03 | TNFSF13B | 1 |
| Hematological System Development and Function | survival | survival of hematopoietic cells | 9.03E-03 | PF4, PROK2 | 2 |
| Hematological System Development and Function | mobilization | mobilization of blood cells | 1.96E-03 | FPR1, PF4, SELL | 3 |
| Hematological System Development and Function | mobilization | mobilization of myeloid cells | 5.94E-03 | FPR1, PF4 | 2 |
| Hematological System Development and Function | mobilization | mobilization of hematopoietic progenitor cells | 8.36E-03 | PF4, SELL | 2 |
| Hematological System Development and Function | morphology | morphology of red blood cells | 2.27E-03 | HBD, NFE2 | 2 |
| Hematological System Development and Function | sequestration | sequestration of blood cells | 2.27E-03 | FCGR2A, IL8RB | 2 |
| Hematological System Development and Function | sequestration | sequestration of red blood cells | 5.54E-03 | FCGR2A | 1 |
| Hematological System Development and Function | function | function of lymphocytes | 3.14E-03 | FCGR2A, PRF1, TNFSF13B | 3 |
| Hematological System Development and Function | transmigration | transmigration of neutrophils | 4.37E-03 | FPR1, IL8RB | 2 |
| Hematological System Development and Function | clearance | clearance of red blood cells | 5.54E-03 | FCGR2A | 1 |
| Hematological System Development and Function | co-localization | co-localization of red blood cells | 5.54E-03 | FCGR2A | 1 |
| Hematological System Development and Function | formation | formation of memory B cells | 5.54E-03 | LTB | 1 |
| Hematological System Development and Function | generation | generation of pro-B lymphocytes | 5.54E-03 | IL7R | 1 |
| Hematological System Development and Function | immunosuppression | immunosuppression of T lymphocytes | 5.54E-03 | PF4 | 1 |
| Hematological System Development and Function | influx | influx of eosinophils | 5.54E-03 | ITK | 1 |
| Hematological System Development and Function | influx | influx of granulocytes | 9.03E-03 | FCGR2A, ITK | 2 |
| Hematological System Development and Function | influx | influx of T lymphocytes | 1.10E-02 | ITK | 1 |
| Hematological System Development and Function | co-stimulation | co-stimulation of B lymphocytes | 1.10E-02 | TNFSF13B | 1 |
| Hematological System Development and Function | colony survival | colony survival of CD34+ cells | 1.10E-02 | PF4 | 1 |
| Hematological System Development and Function | conjugation | conjugation of leukocyte cell lines | 1.10E-02 | LCK | 1 |
| Hematological System Development and Function | desensitization | desensitization of neutrophils | 1.10E-02 | IL8RB | 1 |
| Hematological System Development and Function | elimination | elimination of T lymphocytes | 1.10E-02 | PRF1 | 1 |
| Hematological System Development and Function | red cell distribution width | red cell distribution width of red blood cells | 1.10E-02 | HBD | 1 |
| Hematological System Development and Function | chemoattraction | chemoattraction of leukocytes | 1.35E-02 | C5AR1, PF4 | 2 |
| Tissue Morphology | quantity | quantity of blood cells | 5.59E-12 | C5AR1, CD247, CD8A, CYTIP, FCGR2A, HBD, IL7R, ITK, LCK, LGALS3, LTB, PABPC1, PF4, PIK3AP1, PRF1, PROK2, PTPRC, S100A8, SATB1, SELL, TNFSF13B | 21 |

## Highly Expressed in MBC versus CVS



¬© 2000-2009 Ingenuity Systems, Inc. All rights reserved.

| Category | Function | Function Annotation | P-value | Molecules | # Molecules |
|---|---|---|---|---|---|
| Tissue Morphology | quantity | quantity of leukocytes | 6.71E-11 | C5AR1, CD247, CD8A, CYTIP, FCGR2A, IL7R, ITK, LCK, LGALS3, LTB, PABPC1, PIK3AP1, PRF1, PROK2, PTPRC, S100A8, SATB1, SELL, TNFSF13B | 19 |
| Tissue Morphology | quantity | quantity of mononuclear leukocytes | 6.00E-10 | C5AR1, CD247, CYTIP, FCGR2A, IL7R, ITK, LCK, LTB, PABPC1, PIK3AP1, PRF1, PROK2, PTPRC, SATB1, SELL, TNFSF13B | 16 |
| Tissue Morphology | quantity | quantity of cells | 1.51E-09 | C5AR1, CD247, CD8A, CX3CR1, CYTIP, FCGR2A, HBD, IL7R, IL8RB, ITK, LCK, LGALS3, LTB, PABPC1, PF4, PIK3AP1, PRF1, PROK2, PTPRC, S100A8, SATB1, SELL, SLC4A1, SRGN, TNFSF13B | 25 |
| Tissue Morphology | quantity | quantity of lymphocytes | 2.80E-09 | C5AR1, CD247, CYTIP, FCGR2A, IL7R, ITK, LCK, LTB, PABPC1, PIK3AP1, PRF1, PTPRC, SATB1, SELL, TNFSF13B | 15 |
| Tissue Morphology | quantity | quantity of T lymphocytes | 1.70E-05 | CD247, IL7R, ITK, LCK, LTB, PABPC1, PTPRC, SATB1, TNFSF13B | 9 |
| Tissue Morphology | quantity | quantity of granulocytes | 7.52E-05 | C5AR1, CD8A, LGALS3, PROK2, S100A8, SELL | 6 |
| Tissue Morphology | quantity | quantity of thymocytes | 2.39E-04 | CD247, IL7R, ITK, LCK, SATB1 | 5 |
| Tissue Morphology | quantity | quantity of neutrophils | 9.54E-04 | C5AR1, PROK2, S100A8, SELL | 4 |
| Tissue Morphology | quantity | quantity of natural killer cells | 1.20E-03 | LTB, PRF1, PTPRC | 3 |
| Tissue Morphology | quantity | quantity of intraepithelial T lymphocytes | 1.32E-03 | IL7R, PTPRC | 2 |
| Tissue Morphology | quantity | quantity of phagocytes | 1.92E-03 | C5AR1, LGALS3, PROK2, S100A8, SELL | 5 |
| Tissue Morphology | quantity | quantity of eosinophils | 1.96E-03 | C5AR1, CD8A, LGALS3 | 3 |
| Tissue Morphology | quantity | quantity of plasma cells | 2.64E-03 | IL7R, TNFSF13B | 2 |
| Tissue Morphology | quantity | quantity of memory T lymphocytes | 5.39E-03 | ITK, LTB | 2 |
| Tissue Morphology | quantity | quantity of B-1b lymphocytes | 5.54E-03 | PIK3AP1 | 1 |
| Tissue Morphology | quantity | quantity of transitional type 3 B lymphocytes | 5.54E-03 | TNFSF13B | 1 |
| Tissue Morphology | quantity | quantity of mesenteric lymph node | 1.10E-02 | IL7R | 1 |
| Tissue Morphology | quantity | quantity of B lymphocytes | 1.22E-02 | FCGR2A, IL7R, PIK3AP1, TNFSF13B | 4 |
| Tissue Morphology | quantity | quantity of B-1 lymphocytes | 1.35E-02 | IL7R, PIK3AP1 | 2 |
| Tissue Morphology | quantity | quantity of pre-B lymphocytes | 1.60E-02 | IL7R, TNFSF13B | 2 |
| Immune Cell Trafficking | activation | activation of leukocytes | 1.34E-11 | C5AR1, CD2, CD48, CD247, CD8A, FCGR2A, FPR1, HCK, IL8RB, ITK, LCK, LTB, LTF, PF4, PRF1, PTPRC, SATB1, SELL, TNFSF13B | 19 |
| Immune Cell Trafficking | activation | activation of lymphocytes | 2.84E-07 | CD2, CD48, CD247, CD8A, FCGR2A, ITK, LCK, LTF, PRF1, PTPRC, SATB1, TNFSF13B | 12 |
| Immune Cell Trafficking | activation | activation of neutrophils | 1.03E-06 | C5AR1, FPR1, IL8RB, LTF, PF4, SELL | 6 |
| Immune Cell Trafficking | activation | activation of T lymphocytes | 6.15E-06 | CD2, CD48, CD247, CD8A, ITK, LCK, PRF1, PTPRC, SATB1 | 9 |
| Immune Cell Trafficking | activation | activation of phagocytes | 7.42E-06 | C5AR1, FPR1, HCK, IL8RB, LTF, PF4, SELL | 7 |
| Immune Cell Trafficking | activation | activation of natural killer cells | 1.31E-03 | CD2, FCGR2A, LTF, PRF1 | 4 |
| Immune Cell Trafficking | activation | activation of pro-T lymphocytes | 5.54E-03 | CD2 | 1 |
| Immune Cell Trafficking | activation | activation of B lymphocytes | 7.99E-03 | CD8A, PTPRC, TNFSF13B | 3 |
| Immune Cell Trafficking | activation | activation of antigen presenting cells | 8.53E-03 | FCGR2A, HCK, LTB, LTF | 4 |
| Immune Cell Trafficking | adhesion | adhesion of leukocytes | 3.38E-10 | CD2, CD48, FCGR2A, FGR, HCK, IL8RB, ITK, LGALS3, PF4, PTPRC, RASSF5, S100A8, SELL | 13 |
| Immune Cell Trafficking | adhesion | adhesion of granulocytes | 1.95E-07 | FGR, HCK, IL8RB, LGALS3, PF4, S100A8, SELL | 7 |
| Immune Cell Trafficking | adhesion | adhesion of T lymphocytes | 7.03E-05 | CD2, CD48, ITK, RASSF5, SELL | 5 |
| Immune Cell Trafficking | adhesion | adhesion of neutrophils | 1.78E-04 | IL8RB, PF4, S100A8, SELL | 4 |
| Immune Cell Trafficking | adhesion | initiation of adhesion of monocytes | 1.10E-02 | SELL | 1 |
| Immune Cell Trafficking | adhesion | adhesion of antigen presenting cells | 1.27E-02 | CD2, CD48 | 2 |
| Immune Cell Trafficking | migration | migration of leukocytes | 9.86E-09 | C5AR1, CYTIP, FCGR2A, FGR, FPR1, HCK, IL8RB, ITK, LCK, LGALS3, PF4, PROK2, S100A8, SELL, TNFSF13B | 15 |
| Immune Cell Trafficking | migration | migration of neutrophils | 4.43E-06 | FGR, FPR1, HCK, IL8RB, S100A8, SELL | 6 |
| Immune Cell Trafficking | migration | migration of mononuclear leukocytes | 3.32E-04 | CYTIP, ITK, LCK, LGALS3, PROK2, TNFSF13B | 6 |
| Immune Cell Trafficking | migration | migration of plasma cells | 1.10E-02 | TNFSF13B | 1 |
| Immune Cell Trafficking | cell movement | cell movement of granulocytes | 1.25E-08 | CD2, CD48, FCGR2A, FGR, FPR1, HCK, IL8RB, ITK, LGALS3, PF4, S100A8, SELL | 12 |
| Immune Cell Trafficking | cell movement | cell movement of leukocytes | 3.15E-07 | C5AR1, CD2, CD48, FCGR2A, FGR, FPR1, HCK, IL8RB, ITK, LGALS3, PF4, PRF1, S100A8, SELL | 14 |
| Immune Cell Trafficking | cell movement | cell movement of neutrophils | 7.49E-06 | FCGR2A, FGR, FPR1, HCK, IL8RB, PF4, S100A8, SELL | 8 |
| Immune Cell Trafficking | cell movement | cell movement of eosinophils | 7.94E-05 | CD2, CD48, FPR1, ITK, LGALS3 | 5 |
| Immune Cell Trafficking | cell movement | cell movement of mononuclear leukocytes | 2.76E-03 | FPR1, IL8RB, ITK, PF4, PRF1, SELL | 6 |
| Immune Cell Trafficking | cell movement | cell movement of antigen presenting cells | 6.66E-03 | C5AR1, FPR1, PF4, SELL | 4 |
| Immune Cell Trafficking | cell movement | cell movement of peripheral blood leukocytes | 7.72E-03 | FPR1, SELL | 2 |
| Immune Cell Trafficking | cell movement | cell movement of monocytes | 1.53E-02 | FPR1, IL8RB, PF4 | 3 |
| Immune Cell Trafficking | chemotaxis | chemotaxis of neutrophils | 7.27E-07 | FCGR2A, FGR, FPR1, HCK, IL8RB, PF4, S100A8 | 7 |

| | | | | ¬© 2000-2009 Ingenuity Systems, Inc. All rights reserved. | |
|---|---|---|---|---|---|
| **Category** | **Function** | **Function Annotation** | **P-value** | **Molecules** | **# Molecules** |
| Immune Cell Trafficking | chemotaxis | chemotaxis of leukocytes | 9.02E-05 | C5AR1, FCGR2A, FGR, FPR1, HCK, IL8RB, PF4, S100A8 | 8 |
| Immune Cell Trafficking | chemotaxis | chemotaxis of antigen presenting cells | 1.09E-02 | C5AR1, FPR1, PF4 | 3 |
| Immune Cell Trafficking | recruitment | recruitment of neutrophils | 7.03E-05 | C5AR1, CD8A, CX3CR1, IL8RB, LYZ | 5 |
| Immune Cell Trafficking | recruitment | recruitment of leukocytes | 1.55E-04 | C5AR1, CD8A, CX3CR1, IL8RB, LYZ, SELL | 6 |
| Immune Cell Trafficking | recruitment | recruitment of macrophages | 1.20E-03 | C5AR1, CD8A, CX3CR1 | 3 |
| Immune Cell Trafficking | recruitment | delay in initiation of recruitment of neutrophils | 5.54E-03 | IL8RB | 1 |
| Immune Cell Trafficking | recruitment | initiation of recruitment of neutrophils | 5.54E-03 | C5AR1 | 1 |
| Immune Cell Trafficking | infiltration | infiltration of granulocytes | 1.23E-04 | CD2, CD48, IL8RB, ITK, PF4, SELL | 6 |
| Immune Cell Trafficking | infiltration | infiltration of leukocytes | 4.39E-04 | CD2, CD48, IL8RB, ITK, PF4, PRF1, SELL | 7 |
| Immune Cell Trafficking | infiltration | infiltration of eosinophils | 1.61E-03 | CD2, CD48, ITK | 3 |
| Immune Cell Trafficking | infiltration | infiltration of mononuclear cells | 6.51E-03 | LTB, PF4 | 2 |
| Immune Cell Trafficking | cell rolling | cell rolling of leukocytes | 1.78E-04 | FCGR2A, IL8RB, LGALS3, SELL | 4 |
| Immune Cell Trafficking | cell rolling | cell rolling of granulocytes | 5.39E-03 | LGALS3, SELL | 2 |
| Immune Cell Trafficking | cell rolling | cell rolling of naive T lymphocytes | 5.54E-03 | SELL | 1 |
| Immune Cell Trafficking | cell rolling | cell rolling of mononuclear leukocytes | 5.94E-03 | IL8RB, SELL | 2 |
| Immune Cell Trafficking | cell rolling | arrest in cell rolling of monocytes | 1.65E-02 | IL8RB | 1 |
| Immune Cell Trafficking | emigration | emigration of leukocytes | 6.57E-04 | CYTIP, FCGR2A, SELL | 3 |
| Immune Cell Trafficking | detachment | detachment of leukocytes | 1.06E-03 | PTPRC, SELL | 2 |
| Immune Cell Trafficking | detachment | detachment of macrophages | 1.10E-02 | PTPRC | 1 |
| Immune Cell Trafficking | transmigration | transmigration of neutrophils | 4.37E-03 | FPR1, IL8RB | 2 |
| Immune Cell Trafficking | mobilization | mobilization of myeloid cells | 5.94E-03 | FPR1, PF4 | 2 |
| Immune Cell Trafficking | influx | influx of granulocytes | 9.03E-03 | FCGR2A, ITK | 2 |
| Immune Cell Trafficking | influx | influx of T lymphocytes | 1.10E-02 | ITK | 1 |
| Immune Cell Trafficking | chemoattraction | chemoattraction of leukocytes | 1.35E-02 | C5AR1, PF4 | 2 |
| Tissue Development | adhesion | adhesion of eukaryotic cells | 4.25E-10 | AMICA1, CD2, CD48, CD8A, CYTIP, FCGR2A, FGR, HCK, IL8RB, ITK, LGALS3, LTF, PF4, PTPRC, RASSF5, S100A8, SELL, SLC4A1 | 18 |
| Tissue Development | adhesion | adhesion of cells | 4.53E-10 | AMICA1, C1ORF38, CD2, CD48, CD8A, CX3CR1, CYTIP, FCGR2A, FGR, HCK, IL8RB, ITK, LCK, LGALS3, LTF, PF4, PTPRC, RASSF5, S100A8, SELL, SLC4A1, TNFAIP6 | 22 |
| Tissue Development | adhesion | adhesion of granulocytes | 1.95E-07 | FGR, HCK, IL8RB, LGALS3, PF4, S100A8, SELL | 7 |
| Tissue Development | adhesion | adhesion of T lymphocytes | 7.03E-05 | CD2, CD48, ITK, RASSF5, SELL | 5 |
| Tissue Development | adhesion | adhesion of neutrophils | 1.78E-04 | IL8RB, PF4, S100A8, SELL | 4 |
| Tissue Development | adhesion | adhesion of endothelial cells | 9.89E-03 | AMICA1, SELL, SLC4A1 | 3 |
| Tissue Development | adhesion | adhesion of vascular endothelial tissue | 1.10E-02 | SELL | 1 |
| Tissue Development | adhesion | adhesion of antigen presenting cells | 1.27E-02 | CD2, CD48 | 2 |
| Tissue Development | aggregation | aggregation of cells | 4.28E-04 | CD2, FCGR2A, FPR1, LCK, LTF, PTPRC, SELL | 7 |
| Tissue Development | aggregation | aggregation of granulocytes | 1.61E-03 | FPR1, SELL | 2 |
| Tissue Development | aggregation | aggregation of leukocytes | 1.84E-03 | FPR1, LCK, SELL | 3 |
| Tissue Development | aggregation | aggregation of phagocytes | 3.03E-03 | LCK, SELL | 2 |
| Tissue Development | aggregation | aggregation of blood cells | 8.94E-03 | FCGR2A, FPR1, LCK, SELL | 4 |
| Tissue Development | generation | generation of pro-B lymphocytes | 5.54E-03 | IL7R | 1 |
| Tissue Development | assembly | assembly of extracellular matrix | 9.55E-03 | LGALS3, MPZL3, TNFAIP6 | 3 |
| Cell Signaling | mobilization | mobilization of calcium | 8.30E-10 | C5AR1, CD2, CD247, CX3CR1, FCGR2A, FCGR3B, FPR1, IL8RB, ITK, LCK, LGALS3, PIK3AP1, PROK2, PTPRC, SELL | 15 |
| Cell Signaling | mobilization | mobilization of Ca2+ | 1.61E-04 | CD247, FCGR2A, FPR1, IL8RB, LCK, SELL | 6 |
| Cell Signaling | quantity | quantity of calcium | 8.15E-09 | C5AR1, CD2, CD247, CD8A, CX3CR1, FCGR2A, FCGR3B, FPR1, IL8RB, LCK, LYZ, PTPRC, S100A8, SELL, SIGLEC5 | 15 |
| Cell Signaling | quantity | quantity of Ca2+ | 1.97E-04 | C5AR1, CD52, FPR1, IL8RB, LCK, LYZ, PROK2, PTPRC | 8 |
| Cell Signaling | flux | flux of calcium | 6.37E-08 | CD2, CD247, CD8A, FCGR2A, FCGR3B, FGR, FPR1, HCK, ITK, PRF1, SIGLEC5 | 11 |
| Cell Signaling | tyrosine phosphorylation | tyrosine phosphorylation of protein | 1.53E-06 | CD2, CD48, CD247, CD8A, FCGR2A, HCK, LCK, PTPRC, SELL | 9 |
| Cell Signaling | release | release of calcium | 3.65E-04 | FCGR2A, FPR1, IL8RB, ITK, LCK, PTPRC | 6 |
| Cell Signaling | release | release of Ca2+ | 9.23E-03 | FCGR2A, IL8RB, ITK | 3 |
| Cell Signaling | cell surface receptor linked signal transduction | cell surface receptor linked signal transduction | 3.90E-04 | CD2, CD247, CD8A, FCGR2A, IL7R, LCK, PF4, PTPRC | 8 |
| Cell Signaling | activation | activation of MAP kinase | 5.12E-04 | C5AR1, FPR1, PROK2, PTPRC | 4 |
| Cell Signaling | secretion | secretion of nitric oxide | 2.27E-03 | CD8A, PTPRC | 2 |
| Cell Signaling | response | response of calcium | 4.37E-03 | ITK, LCK | 2 |
| Cell Signaling | influx | influx of calcium | 5.68E-03 | FCGR2A, FCGR3B, FPR1, ITK | 4 |

| | | | | ¬© 2000-2009 Ingenuity Systems, Inc. All rights reserved. | |
|---|---|---|---|---|---|
| **Category** | **Function** | **Function Annotation** | **P-value** | **Molecules** | **# Molecules** |
| Molecular Transport | mobilization | mobilization of calcium | 8.30E-10 | C5AR1, CD2, CD247, CX3CR1, FCGR2A, FCGR3B, FPR1, IL8RB, ITK, LCK, LGALS3, PIK3AP1, PROK2, PTPRC, SELL | 15 |
| Molecular Transport | mobilization | mobilization of Ca2+ | 1.61E-04 | CD247, FCGR2A, FPR1, IL8RB, LCK, SELL | 6 |
| Molecular Transport | quantity | quantity of calcium | 8.15E-09 | C5AR1, CD2, CD247, CD8A, CX3CR1, FCGR2A, FCGR3B, FPR1, IL8RB, LCK, LYZ, PTPRC, S100A8, SELL, SIGLEC5 | 15 |
| Molecular Transport | quantity | quantity of Ca2+ | 1.97E-04 | C5AR1, CD52, FPR1, IL8RB, LCK, LYZ, PROK2, PTPRC | 8 |
| Molecular Transport | flux | flux of calcium | 6.37E-08 | CD2, CD247, CD8A, FCGR2A, FCGR3B, FGR, FPR1, HCK, ITK, PRF1, SIGLEC5 | 11 |
| Molecular Transport | release | release of superoxide | 2.78E-05 | FCGR2A, FGR, HCK, NCF2 | 4 |
| Molecular Transport | release | release of calcium | 3.65E-04 | FCGR2A, FPR1, IL8RB, ITK, LCK, PTPRC | 6 |
| Molecular Transport | release | release of 5-hydroxytryptamine | 6.57E-04 | CD247, FCGR2A, SIGLEC5 | 3 |
| Molecular Transport | release | release of Ca2+ | 9.23E-03 | FCGR2A, IL8RB, ITK | 3 |
| Molecular Transport | secretion | secretion of nitric oxide | 2.27E-03 | CD8A, PTPRC | 2 |
| Molecular Transport | efflux | efflux of sulfate | 5.54E-03 | SLC4A1 | 1 |
| Molecular Transport | transport | transport of amine | 5.54E-03 | AQP9 | 1 |
| Molecular Transport | transport | transport of carboxylic acid | 5.54E-03 | AQP9 | 1 |
| Molecular Transport | transport | transport of purine base | 5.54E-03 | AQP9 | 1 |
| Molecular Transport | transport | transport of pyrimidine base | 1.10E-02 | AQP9 | 1 |
| Molecular Transport | transport | transport of taurine | 1.10E-02 | SLC4A1 | 1 |
| Molecular Transport | influx | influx of calcium | 5.68E-03 | FCGR2A, FCGR3B, FPR1, ITK | 4 |
| Molecular Transport | accumulation | accumulation of phosphatidylinositol 4,5-diphosphate | 1.10E-02 | FCGR2A | 1 |
| Molecular Transport | co-localization | co-localization of F-actin | 1.10E-02 | FCGR2A | 1 |
| Molecular Transport | concentration | concentration of bilirubin | 1.10E-02 | HBD | 1 |
| Molecular Transport | uptake | uptake of DNA fragment | 1.10E-02 | LTF | 1 |
| Vitamin and Mineral Metabolism | mobilization | mobilization of calcium | 8.30E-10 | C5AR1, CD2, CD247, CX3CR1, FCGR2A, FCGR3B, FPR1, IL8RB, ITK, LCK, LGALS3, PIK3AP1, PROK2, PTPRC, SELL | 15 |
| Vitamin and Mineral Metabolism | mobilization | mobilization of Ca2+ | 1.61E-04 | CD247, FCGR2A, FPR1, IL8RB, LCK, SELL | 6 |
| Vitamin and Mineral Metabolism | quantity | quantity of calcium | 8.15E-09 | C5AR1, CD2, CD247, CD8A, CX3CR1, FCGR2A, FCGR3B, FPR1, IL8RB, LCK, LYZ, PTPRC, S100A8, SELL, SIGLEC5 | 15 |
| Vitamin and Mineral Metabolism | quantity | quantity of Ca2+ | 1.97E-04 | C5AR1, CD52, FPR1, IL8RB, LCK, LYZ, PROK2, PTPRC | 8 |
| Vitamin and Mineral Metabolism | flux | flux of calcium | 6.37E-08 | CD2, CD247, CD8A, FCGR2A, FCGR3B, FGR, FPR1, HCK, ITK, PRF1, SIGLEC5 | 11 |
| Vitamin and Mineral Metabolism | release | release of calcium | 3.65E-04 | FCGR2A, FPR1, IL8RB, ITK, LCK, PTPRC | 6 |
| Vitamin and Mineral Metabolism | release | release of Ca2+ | 9.23E-03 | FCGR2A, IL8RB, ITK | 3 |
| Vitamin and Mineral Metabolism | response | response of calcium | 4.37E-03 | ITK, LCK | 2 |
| Vitamin and Mineral Metabolism | influx | influx of calcium | 5.68E-03 | FCGR2A, FCGR3B, FPR1, ITK | 4 |
| Cell Death | cytotoxicity | cytotoxicity of leukocytes | 2.90E-09 | CD2, CD48, FCGR2A, FCGR3B, FGR, HCK, ITK, LCK, PRF1, PTPRC | 10 |
| Cell Death | cytotoxicity | cytotoxicity of lymphocytes | 3.54E-07 | CD2, CD48, FCGR2A, FCGR3B, ITK, LCK, PRF1, PTPRC | 8 |
| Cell Death | cytotoxicity | cytotoxicity of natural killer cells | 3.59E-07 | CD2, CD48, FCGR2A, FCGR3B, ITK, LCK, PRF1 | 7 |
| Cell Death | cytotoxicity | cytotoxicity of macrophages | 1.06E-03 | FGR, HCK | 2 |
| Cell Death | cytotoxicity | cytotoxicity of cytotoxic T cells | 9.71E-03 | PRF1, PTPRC | 2 |
| Cell Death | cytotoxicity | cytotoxicity of endothelial cell lines | 1.10E-02 | PRF1 | 1 |
| Cell Death | cytolysis | cytolysis | 8.81E-08 | CD48, CD247, CX3CR1, FCGR2A, LCK, PRF1, PTPRC, SLC4A1 | 8 |
| Cell Death | cytolysis | cytolysis of eukaryotic cells | 6.17E-07 | CD48, CX3CR1, FCGR2A, LCK, PRF1, PTPRC | 6 |
| Cell Death | cytolysis | cytolysis of T lymphocytes | 6.51E-07 | LCK, PRF1, PTPRC | 3 |
| Cell Death | cytolysis | cytolysis of intraepithelial T lymphocytes | 3.03E-05 | LCK, PTPRC | 2 |
| Cell Death | cytolysis | cytolysis of tumor cells | 2.27E-03 | FCGR2A, PRF1 | 2 |
| Cell Death | cytolysis | cytolysis of myeloid cells | 5.54E-03 | PRF1 | 1 |
| Cell Death | cytolysis | cytolysis of cell lines | 9.71E-03 | CD48, CX3CR1 | 2 |
| Cell Death | apoptosis | apoptosis of leukocytes | 5.93E-07 | CD2, CD247, IL7R, ITK, LCK, LGALS3, NAMPT, PF4, PRF1, PTPRC, SATB1, TNFSF13B | 12 |
| Cell Death | apoptosis | apoptosis of mononuclear leukocytes | 2.41E-06 | CD2, CD247, IL7R, ITK, LCK, PF4, PRF1, PTPRC, SATB1, TNFSF13B | 10 |
| Cell Death | apoptosis | apoptosis of lymphocytes | 1.42E-05 | CD2, CD247, IL7R, ITK, LCK, PRF1, PTPRC, SATB1, TNFSF13B | 9 |
| Cell Death | apoptosis | apoptosis of T lymphocytes | 1.78E-05 | CD2, CD247, IL7R, ITK, LCK, PRF1, PTPRC, SATB1 | 8 |
| Cell Death | apoptosis | apoptosis of leukemia cell lines | 2.34E-05 | FGR, LCK, LGALS3, PRF1, PTPRC, RASSF5, S100A8, TNFSF13B | 8 |
| Cell Death | apoptosis | apoptosis of normal cells | 4.43E-03 | CD2, CD247, IL7R, IL8RB, ITK, LCK, LGALS3, NAMPT, PF4, PRF1, PTPRC, SATB1, TNFSF13B | 13 |
| Cell Death | apoptosis | apoptosis of intraepithelial T lymphocytes | 5.54E-03 | PTPRC | 1 |
| Cell Death | apoptosis | apoptosis of hematopoietic progenitor cells | 7.72E-03 | PF4, PRF1 | 2 |

| | | | | ¬© 2000-2009 Ingenuity Systems, Inc. All rights reserved. | |
|---|---|---|---|---|---|
| **Category** | **Function** | **Function Annotation** | **P-value** | **Molecules** | **# Molecules** |
| Cell Death | apoptosis | apoptosis of eukaryotic cells | 8.71E-03 | CD2, CD48, CD247, CD8A, FGR, IL7R, IL8RB, ITK, LCK, LGALS3, NAMPT, PF4, PRF1, PTPRC, RASSF5, S100A8, SATB1, TNFSF13B | 18 |
| Cell Death | apoptosis | apoptosis | 8.94E-03 | CD2, CD48, CD247, CD8A, FGR, HCK, IL7R, IL8RB, ITK, LCK, LGALS3, LTB, NAMPT, PF4, PRF1, PTPRC, RASSF5, S100A8, SATB1, SRGN, TNFSF13B | 21 |
| Cell Death | apoptosis | apoptosis of B lymphocytes | 1.06E-02 | PRF1, PTPRC, TNFSF13B | 3 |
| Cell Death | apoptosis | apoptosis of naive T lymphocytes | 1.10E-02 | PRF1 | 1 |
| Cell Death | apoptosis | apoptosis of oligodendrocyte precursor cells | 1.10E-02 | IL8RB | 1 |
| Cell Death | apoptosis | apoptosis of CD34+ cells | 1.65E-02 | PF4 | 1 |
| Cell Death | lysis | lysis of eukaryotic cells | 6.26E-07 | CD48, CX3CR1, FCGR2A, LCK, PRF1, PTPRC, SLC4A1 | 7 |
| Cell Death | lysis | lysis of blood cells | 3.55E-05 | LCK, PRF1, PTPRC, SLC4A1 | 4 |
| Cell Death | lysis | lysis of cell lines | 1.50E-03 | CD48, CX3CR1, PRF1 | 3 |
| Cell Death | lysis | lysis of red blood cells | 5.39E-03 | PRF1, SLC4A1 | 2 |
| Cell Death | cell death | cell death of leukemia cell lines | 8.69E-07 | FGR, GZMH, GZMK, LCK, LGALS3, PRF1, PTPRC, RASSF5, S100A8, TNFSF13B | 10 |
| Cell Death | cell death | cell death of eukaryotic cells | 1.09E-03 | CD2, CD48, CD247, CD8A, CX3CR1, FGR, GZMH, GZMK, IL7R, IL8RB, ITK, LCK, LGALS3, LTB, NAMPT, NCF2, PF4, PRF1, PTPRC, RASSF5, S100A8, SATB1, TNFSF13B | 23 |
| Cell Death | cell death | cell death | 1.44E-03 | CD2, CD48, CD247, CD8A, CX3CR1, FGR, GZMH, GZMK, HCK, IL7R, IL8RB, ITK, LCK, LGALS3, LTB, LYZ, NAMPT, NCF2, PF4, PRF1, PTPRC, RASSF5, S100A8, SATB1, SRGN, TNFSF13B | 26 |
| Cell Death | cell death | cell death of normal cells | 1.44E-03 | CD2, CD247, CX3CR1, IL7R, IL8RB, ITK, LCK, LGALS3, LTB, NAMPT, NCF2, PF4, PRF1, PTPRC, SATB1, TNFSF13B | 16 |
| Cell Death | cell death | cell death of tumor cell lines | 1.10E-02 | CD48, FGR, GZMH, GZMK, LCK, LGALS3, LTB, NAMPT, PRF1, PTPRC, RASSF5, S100A8, TNFSF13B | 13 |
| Cell Death | cell death | cell death of lymphoma cell lines | 1.24E-02 | CD48, LGALS3, PTPRC, TNFSF13B | 4 |
| Cell Death | killing | killing of cells | 2.89E-05 | CD48, LGALS3, LTF, LYZ, PRF1, S100A8 | 6 |
| Cell Death | killing | killing of lymphocytes | 6.57E-04 | CD48, LGALS3, PRF1 | 3 |
| Cell Death | killing | killing of natural killer cells | 2.27E-03 | CD48, PRF1 | 2 |
| Cell Death | survival | survival of blood cells | 7.23E-05 | CD8A, HCK, NCF2, NFE2, PF4, PROK2, TNFSF13B | 7 |
| Cell Death | survival | survival of red blood cells | 1.61E-03 | NFE2, PF4 | 2 |
| Cell Death | survival | survival of lymphocytes | 4.27E-03 | CD8A, HCK, NCF2, TNFSF13B | 4 |
| Cell Death | survival | survival of transitional type 2 B lymphocytes | 5.54E-03 | TNFSF13B | 1 |
| Cell Death | survival | survival of transitional type 3 B lymphocytes | 5.54E-03 | TNFSF13B | 1 |
| Cell Death | survival | survival of hematopoietic cells | 9.03E-03 | PF4, PROK2 | 2 |
| Cell Death | survival | survival of eukaryotic cells | 1.51E-02 | CD8A, FGR, HCK, LCK, LGALS3, NCF2, NFE2, PF4, PROK2, TNFSF13B | 10 |
| Cell Death | activation-induced cell death | activation-induced cell death | 3.67E-03 | ITK, LTB, PRF1 | 3 |
| Cell Death | activation-induced cell death | activation-induced cell death of T lymphocytes | 1.12E-02 | ITK, PRF1 | 2 |
| Cell Death | colony survival | colony survival of CD34+ cells | 1.10E-02 | PF4 | 1 |
| Hematopoiesis | binding | binding of red blood cells | 3.51E-09 | CD2, FCGR2A, FCGR3B, LTF, SIGLEC5 | 5 |
| Hematopoiesis | development | development of leukocytes | 3.22E-06 | CD247, CD8A, FCGR2A, IL7R, ITK, LCK, LTB, PTPRC, SATB1, TNFSF13B | 10 |
| Hematopoiesis | development | development of T lymphocytes | 7.20E-06 | CD247, CD8A, FCGR2A, IL7R, ITK, LCK, PTPRC, SATB1 | 8 |
| Hematopoiesis | development | development of lymphocytes | 7.52E-06 | CD247, CD8A, FCGR2A, IL7R, ITK, LCK, PTPRC, SATB1, TNFSF13B | 9 |
| Hematopoiesis | development | development of intraepithelial T lymphocytes | 2.99E-04 | LCK, PTPRC | 2 |
| Hematopoiesis | development | development of alpha-beta T lymphocytes | 6.24E-04 | FCGR2A, LCK | 2 |
| Hematopoiesis | development | development of thymocytes | 4.89E-03 | IL7R, PTPRC, SATB1 | 3 |
| Hematopoiesis | development | development of transitional type 1 B lymphocytes | 5.54E-03 | TNFSF13B | 1 |
| Hematopoiesis | development | development of pre-B lymphocytes | 1.04E-02 | IL7R, TNFSF13B | 2 |
| Hematopoiesis | development | arrest in development of alpha-beta T lymphocytes | 1.10E-02 | LCK | 1 |
| Hematopoiesis | hematopoiesis | hematopoiesis | 2.09E-05 | CD2, CD247, CD8A, FCGR2A, HBD, IL7R, ITK, LCK, LTB, LTF, PTPRC, SATB1, TNFSF13B | 13 |
| Hematopoiesis | quantity | quantity of thymocytes | 2.39E-04 | CD247, IL7R, ITK, LCK, SATB1 | 5 |
| Hematopoiesis | quantity | quantity of transitional type 3 B lymphocytes | 5.54E-03 | TNFSF13B | 1 |
| Hematopoiesis | quantity | quantity of pre-B lymphocytes | 1.60E-02 | IL7R, TNFSF13B | 2 |
| Hematopoiesis | differentiation | differentiation of gamma-delta T lymphocytes | 4.48E-04 | LCK, PTPRC | 2 |
| Hematopoiesis | differentiation | differentiation of mononuclear leukocytes | 4.98E-04 | CD2, CD8A, IL7R, ITK, LCK, PF4, PTPRC, TNFSF13B | 8 |
| Hematopoiesis | differentiation | differentiation of T lymphocytes | 1.02E-03 | CD2, CD8A, IL7R, ITK, LCK, PTPRC | 6 |
| Hematopoiesis | differentiation | differentiation of lymphocytes | 1.22E-03 | CD2, CD8A, IL7R, ITK, LCK, PTPRC, TNFSF13B | 7 |
| Hematopoiesis | differentiation | differentiation of transitional type 2 B lymphocytes | 5.54E-03 | TNFSF13B | 1 |

| | | | | ¬© 2000-2009 Ingenuity Systems, Inc. All rights reserved. | |
|---|---|---|---|---|---|
| **Category** | **Function** | **Function Annotation** | **P-value** | **Molecules** | **# Molecules** |
| Hematopoiesis | differentiation | differentiation of megakaryocytes | 7.72E-03 | NFE2, PF4 | 2 |
| Hematopoiesis | differentiation | arrest in differentiation of erythroblasts | 1.10E-02 | PTPRC | 1 |
| Hematopoiesis | proliferation | proliferation of hematopoietic progenitor cells | 4.83E-04 | IL7R, NFE2, PF4, PTPRC | 4 |
| Hematopoiesis | morphology | morphology of red blood cells | 2.27E-03 | HBD, NFE2 | 2 |
| Hematopoiesis | activation | activation of pro-T lymphocytes | 5.54E-03 | CD2 | 1 |
| Hematopoiesis | generation | generation of pro-B lymphocytes | 5.54E-03 | IL7R | 1 |
| Hematopoiesis | mobilization | mobilization of hematopoietic progenitor cells | 8.36E-03 | PF4, SELL | 2 |
| Hematopoiesis | colony survival | colony survival of CD34+ cells | 1.10E-02 | PF4 | 1 |
| Cellular Movement | migration | migration of leukocytes | 9.86E-09 | C5AR1, CYTIP, FCGR2A, FGR, FPR1, HCK, IL8RB, ITK, LCK, LGALS3, PF4, PROK2, S100A8, SELL, TNFSF13B | 15 |
| Cellular Movement | migration | migration of phagocytes | 1.67E-06 | FGR, FPR1, HCK, IL8RB, LGALS3, S100A8, SELL | 7 |
| Cellular Movement | migration | migration of neutrophils | 4.43E-06 | FGR, FPR1, HCK, IL8RB, S100A8, SELL | 6 |
| Cellular Movement | migration | migration of mononuclear leukocytes | 3.32E-04 | CYTIP, ITK, LCK, LGALS3, PROK2, TNFSF13B | 6 |
| Cellular Movement | migration | migration of cells | 6.70E-04 | C5AR1, CYTIP, FCGR2A, FGR, FPR1, HCK, IL8RB, ITK, LCK, LGALS3, PF4, PROK2, RGS18, S100A8, SELL, TNFSF13B | 16 |
| Cellular Movement | migration | migration of lymphocytes | 6.49E-03 | CYTIP, ITK, LCK, TNFSF13B | 4 |
| Cellular Movement | migration | arrest in migration of oligodendrocyte precursor cells | 1.10E-02 | IL8RB | 1 |
| Cellular Movement | migration | migration of plasma cells | 1.10E-02 | TNFSF13B | 1 |
| Cellular Movement | cell movement | cell movement of granulocytes | 1.25E-08 | CD2, CD48, FCGR2A, FGR, FPR1, HCK, IL8RB, ITK, LGALS3, PF4, S100A8, SELL | 12 |
| Cellular Movement | cell movement | cell movement of blood cells | 6.13E-08 | C5AR1, CD2, CD48, FCGR2A, FGR, FPR1, HCK, IL8RB, ITK, LGALS3, LTB, PF4, PRF1, S100A8, SELL | 15 |
| Cellular Movement | cell movement | cell movement of normal cells | 3.01E-07 | C5AR1, CD2, CD48, CX3CR1, FCGR2A, FGR, FPR1, HCK, IL8RB, ITK, LGALS3, LTB, PF4, PRF1, S100A8, SELL | 16 |
| Cellular Movement | cell movement | cell movement of leukocytes | 3.15E-07 | C5AR1, CD2, CD48, FCGR2A, FGR, FPR1, HCK, IL8RB, ITK, LGALS3, PF4, PRF1, S100A8, SELL | 14 |
| Cellular Movement | cell movement | cell movement | 7.69E-07 | C5AR1, CD2, CD48, CX3CR1, FCGR2A, FGR, FPR1, HCK, IL8RB, ITK, LCK, LGALS3, LTB, PF4, PRF1, PROK2, S100A8, SELL, VNN2 | 19 |
| Cellular Movement | cell movement | cell movement of eukaryotic cells | 1.73E-06 | C5AR1, CD2, CD48, CX3CR1, FCGR2A, FGR, FPR1, HCK, IL8RB, ITK, LCK, LGALS3, LTB, PF4, PRF1, S100A8, SELL | 17 |
| Cellular Movement | cell movement | cell movement of neutrophils | 7.49E-06 | FCGR2A, FGR, FPR1, HCK, IL8RB, PF4, S100A8, SELL | 8 |
| Cellular Movement | cell movement | cell movement of eosinophils | 7.94E-05 | CD2, CD48, FPR1, ITK, LGALS3 | 5 |
| Cellular Movement | cell movement | cell movement of leukemia cell lines | 2.36E-03 | FPR1, LCK, SELL | 3 |
| Cellular Movement | cell movement | cell movement of mononuclear leukocytes | 2.76E-03 | FPR1, IL8RB, ITK, PF4, PRF1, SELL | 6 |
| Cellular Movement | cell movement | cell movement of antigen presenting cells | 6.66E-03 | C5AR1, FPR1, PF4, SELL | 4 |
| Cellular Movement | cell movement | cell movement of peripheral blood leukocytes | 7.72E-03 | FPR1, SELL | 2 |
| Cellular Movement | cell movement | cell movement of monocytes | 1.53E-02 | FPR1, IL8RB, PF4 | 3 |
| Cellular Movement | chemotaxis | chemotaxis of neutrophils | 7.27E-07 | FCGR2A, FGR, FPR1, HCK, IL8RB, PF4, S100A8 | 7 |
| Cellular Movement | chemotaxis | chemotaxis | 6.08E-06 | C5AR1, CX3CR1, FCGR2A, FGR, FPR1, HCK, IL8RB, LCK, LGALS3, PF4, PROK2, S100A8 | 12 |
| Cellular Movement | chemotaxis | chemotaxis of eukaryotic cells | 1.00E-05 | C5AR1, CX3CR1, FCGR2A, FGR, FPR1, HCK, IL8RB, LCK, LGALS3, PF4, S100A8 | 11 |
| Cellular Movement | chemotaxis | chemotaxis of normal cells | 6.45E-05 | C5AR1, CX3CR1, FCGR2A, FGR, FPR1, HCK, IL8RB, PF4, S100A8 | 9 |
| Cellular Movement | chemotaxis | chemotaxis of leukocytes | 9.02E-05 | C5AR1, FCGR2A, FGR, FPR1, HCK, IL8RB, PF4, S100A8 | 8 |
| Cellular Movement | chemotaxis | chemotaxis of endothelial cell lines | 5.94E-03 | IL8RB, LGALS3 | 2 |
| Cellular Movement | chemotaxis | chemotaxis of antigen presenting cells | 1.09E-02 | C5AR1, FPR1, PF4 | 3 |
| Cellular Movement | chemotaxis | chemotaxis of cell lines | 1.64E-02 | FPR1, IL8RB, LCK, LGALS3 | 4 |
| Cellular Movement | movement | movement of normal cells | 1.20E-05 | C5AR1, CD8A, CYTIP, FCGR2A, FGR, FPR1, HCK, IL8RB, ITK, LCK, LGALS3, PF4, PROK2, S100A8, SELL, TNFSF13B | 16 |
| Cellular Movement | movement | movement of cells | 2.53E-04 | C5AR1, CD8A, CYTIP, FCGR2A, FGR, FPR1, HCK, IL8RB, ITK, LCK, LGALS3, PF4, PROK2, RGS18, S100A8, SELL, TNFSF13B | 17 |
| Cellular Movement | infiltration | infiltration of blood cells | 9.02E-05 | CD2, CD48, IL8RB, ITK, LTB, PF4, PRF1, SELL | 8 |
| Cellular Movement | infiltration | infiltration of granulocytes | 1.23E-04 | CD2, CD48, IL8RB, ITK, PF4, SELL | 6 |
| Cellular Movement | infiltration | infiltration of leukocytes | 4.39E-04 | CD2, CD48, IL8RB, ITK, PF4, PRF1, SELL | 7 |
| Cellular Movement | infiltration | infiltration of eosinophils | 1.61E-03 | CD2, CD48, ITK | 3 |
| Cellular Movement | infiltration | infiltration of mononuclear cells | 6.51E-03 | LTB, PF4 | 2 |
| Cellular Movement | infiltration | infiltration of kidney | 1.10E-02 | LTB | 1 |
| Cellular Movement | cell rolling | cell rolling of leukocytes | 1.78E-04 | FCGR2A, IL8RB, LGALS3, SELL | 4 |
| Cellular Movement | cell rolling | cell rolling of granulocytes | 5.39E-03 | LGALS3, SELL | 2 |

| | | | | ¬© 2000-2009 Ingenuity Systems, Inc. All rights reserved. | |
|---|---|---|---|---|---|
| **Category** | **Function** | **Function Annotation** | **P-value** | **Molecules** | **# Molecules** |
| Cellular Movement | cell rolling | cell rolling of naive T lymphocytes | 5.54E-03 | SELL | 1 |
| Cellular Movement | cell rolling | cell rolling of mononuclear leukocytes | 5.94E-03 | IL8RB, SELL | 2 |
| Cellular Movement | cell rolling | cell rolling of lymphoma cell lines | 1.10E-02 | SELL | 1 |
| Cellular Movement | cell rolling | arrest in cell rolling of monocytes | 1.65E-02 | IL8RB | 1 |
| Cellular Movement | mobilization | mobilization of normal cells | 2.23E-04 | FPR1, PF4, PROK2, SELL | 4 |
| Cellular Movement | mobilization | mobilization of blood cells | 1.96E-03 | FPR1, PF4, SELL | 3 |
| Cellular Movement | mobilization | mobilization of myeloid cells | 5.94E-03 | FPR1, PF4 | 2 |
| Cellular Movement | mobilization | mobilization of hematopoietic progenitor cells | 8.36E-03 | PF4, SELL | 2 |
| Cellular Movement | emigration | emigration of leukocytes | 6.57E-04 | CYTIP, FCGR2A, SELL | 3 |
| Cellular Movement | sequestration | sequestration of blood cells | 2.27E-03 | FCGR2A, IL8RB | 2 |
| Cellular Movement | sequestration | sequestration of red blood cells | 5.54E-03 | FCGR2A | 1 |
| Cellular Movement | transmigration | transmigration of neutrophils | 4.37E-03 | FPR1, IL8RB | 2 |
| Cellular Movement | co-localization | co-localization of red blood cells | 5.54E-03 | FCGR2A | 1 |
| Cellular Movement | distribution | distribution of oligodendrocyte precursor cells | 5.54E-03 | IL8RB | 1 |
| Cellular Movement | influx | influx of eosinophils | 5.54E-03 | ITK | 1 |
| Cellular Movement | influx | influx of granulocytes | 9.03E-03 | FCGR2A, ITK | 2 |
| Cellular Movement | influx | influx of T lymphocytes | 1.10E-02 | ITK | 1 |
| Cellular Movement | positioning | positioning of cells | 7.72E-03 | IL8RB, LTB | 2 |
| Cellular Movement | scattering | scattering of keratinocytes | 1.10E-02 | CD8A | 1 |
| Cellular Movement | chemoattraction | chemoattraction of leukocytes | 1.35E-02 | C5AR1, PF4 | 2 |
| Immunological Disease | immunological disorder | immunological disorder | 1.35E-07 | AMICA1, AQP9, CD2, CD52, CD247, CD8A, CFP, CX3CR1, CYP4F3, FAM129A, FCGR2A, FCN1, HCK, IL7R, IL8RB, ITK, KCNJ15, LCK, LRRK2, LTB, LTF, LYZ, NAMPT, NCF2, P2RY13, PF4, PIK3AP1, PRF1, PTPRC, S100A8, S100A12, SELL, SORL1, TAGAP, TNFAIP6, TNFSF13B | 36 |
| Immunological Disease | immunological disorder | immunological disorder of mammalia | 4.35E-03 | CX3CR1, FCGR2A, IL7R, LTB, NCF2, PRF1, SELL | 7 |
| Immunological Disease | immunological disorder | immunological disorder of mice | 1.10E-02 | CX3CR1, FCGR2A, IL7R, LTB, PRF1, SELL | 6 |
| Immunological Disease | apoptosis | apoptosis of leukocytes | 5.93E-07 | CD2, CD247, IL7R, ITK, LCK, LGALS3, NAMPT, PF4, PRF1, PTPRC, SATB1, TNFSF13B | 12 |
| Immunological Disease | apoptosis | apoptosis of mononuclear leukocytes | 2.41E-06 | CD2, CD247, IL7R, ITK, LCK, PF4, PRF1, PTPRC, SATB1, TNFSF13B | 10 |
| Immunological Disease | apoptosis | apoptosis of lymphocytes | 1.42E-05 | CD2, CD247, IL7R, ITK, LCK, PRF1, PTPRC, SATB1, TNFSF13B | 9 |
| Immunological Disease | apoptosis | apoptosis of T lymphocytes | 1.78E-05 | CD2, CD247, IL7R, ITK, LCK, PRF1, PTPRC, SATB1 | 8 |
| Immunological Disease | apoptosis | apoptosis of intraepithelial T lymphocytes | 5.54E-03 | PTPRC | 1 |
| Immunological Disease | apoptosis | apoptosis of B lymphocytes | 1.06E-02 | PRF1, PTPRC, TNFSF13B | 3 |
| Immunological Disease | apoptosis | apoptosis of naive T lymphocytes | 1.10E-02 | PRF1 | 1 |
| Immunological Disease | cytolysis | cytolysis of T lymphocytes | 6.51E-07 | LCK, PRF1, PTPRC | 3 |
| Immunological Disease | cytolysis | cytolysis of intraepithelial T lymphocytes | 3.03E-05 | LCK, PTPRC | 2 |
| Immunological Disease | cytolysis | cytolysis of myeloid cells | 5.54E-03 | PRF1 | 1 |
| Immunological Disease | polyarticular juvenile rheumatoid arthritis | polyarticular juvenile rheumatoid arthritis | 7.32E-06 | P2RY13, PTPRC, S100A8, S100A12, SORL1, TNFSF13B | 6 |
| Immunological Disease | rheumatoid arthritis | rheumatoid arthritis | 3.81E-05 | AQP9, CYP4F3, FAM129A, FCN1, HCK, IL7R, IL8RB, KCNJ15, LRRK2, LTB, LTF, LYZ, NAMPT, P2RY13, PIK3AP1, PTPRC, S100A8, S100A12, SORL1, TAGAP, TNFAIP6, TNFSF13B | 22 |
| Immunological Disease | autoimmune disease | autoimmune disease | 5.48E-05 | AMICA1, AQP9, CD2, CD52, CX3CR1, CYP4F3, FAM129A, FCGR2A, FCN1, HCK, IL7R, IL8RB, KCNJ15, LRRK2, LTB, LTF, LYZ, NAMPT, P2RY13, PIK3AP1, PTPRC, S100A8, S100A12, SELL, SORL1, TAGAP, TNFAIP6, TNFSF13B | 28 |
| Immunological Disease | autoimmune disease | autoimmune disease of rodents | 8.16E-03 | CX3CR1, FCGR2A, IL7R, LTB, SELL | 5 |
| Immunological Disease | graft-vs-host disease | graft-vs-host disease | 6.17E-05 | CD2, CD52, CD247, PRF1 | 4 |
| Immunological Disease | severe combined immunodeficiency | severe combined immunodeficiency | 8.68E-05 | CD247, IL7R, PTPRC | 3 |
| Immunological Disease | immunodeficiency | immunodeficiency | 1.65E-04 | CD247, CFP, IL7R, PTPRC | 4 |
| Immunological Disease | killing | killing of lymphocytes | 6.57E-04 | CD48, LGALS3, PRF1 | 3 |
| Immunological Disease | killing | killing of natural killer cells | 2.27E-03 | CD48, PRF1 | 2 |
| Immunological Disease | autoimmune hemolytic anemia | autoimmune hemolytic anemia of mice | 1.06E-03 | FCGR2A, IL7R | 2 |
| Immunological Disease | detachment | detachment of leukocytes | 1.06E-03 | PTPRC, SELL | 2 |
| Immunological Disease | detachment | detachment of macrophages | 1.10E-02 | PTPRC | 1 |
| Immunological Disease | disease | disease of lymphoid organ | 1.20E-03 | LCK, LTB, PRF1 | 3 |
| Immunological Disease | disease | disease of leukocytes | 3.14E-03 | FPR1, LTF, PF4 | 3 |
| Immunological Disease | disease | disease of spleen | 8.36E-03 | LTB, PRF1 | 2 |
| Immunological Disease | disease | disease of mononuclear leukocytes | 1.19E-02 | FPR1, PF4 | 2 |

| | | | | ¬© 2000-2009 Ingenuity Systems, Inc. All rights reserved. | |
|---|---|---|---|---|---|
| **Category** | **Function** | **Function Annotation** | **P-value** | **Molecules** | **# Molecules** |
| Immunological Disease | systemic lupus erythematosus | systemic lupus erythematosus | 2.20E-03 | FCGR2A, IL7R, PTPRC, TNFSF13B | 4 |
| Immunological Disease | systemic lupus erythematosus | systemic lupus erythematosus of animal | 8.36E-03 | PTPRC, TNFSF13B | 2 |
| Immunological Disease | familial CD8 deficiency | familial CD8 deficiency | 5.54E-03 | CD8A | 1 |
| Immunological Disease | immunosuppression | immunosuppression of T lymphocytes | 5.54E-03 | PF4 | 1 |
| Immunological Disease | lymphadenopathy | lymphadenopathy of skin | 5.54E-03 | SELL | 1 |
| Immunological Disease | size | size of lymphoma cell lines | 5.54E-03 | PTPRC | 1 |
| Immunological Disease | infection | infection of leukocytes | 9.03E-03 | FPR1, LTF | 2 |
| Immunological Disease | cell rolling | cell rolling of lymphoma cell lines | 1.10E-02 | SELL | 1 |
| Immunological Disease | deletion | delay in initiation of deletion of T lymphocytes | 1.10E-02 | CD2 | 1 |
| Immunological Disease | development | development of thymic lymphoma | 1.10E-02 | PTPRC | 1 |
| Immunological Disease | familial hemophagocytic lymphohistiocytosis, type 2 | familial hemophagocytic lymphohistiocytosis, type 2 | 1.10E-02 | PRF1 | 1 |
| Immunological Disease | lymphomagenesis | lymphomagenesis of natural killer cell lymphoma | 1.10E-02 | PRF1 | 1 |
| Immunological Disease | activation-induced cell death | activation-induced cell death of T lymphocytes | 1.12E-02 | ITK, PRF1 | 2 |
| Immunological Disease | cell death | cell death of lymphoma cell lines | 1.24E-02 | CD48, LGALS3, PTPRC, TNFSF13B | 4 |
| Infectious Disease | severe acute respiratory syndrome | severe acute respiratory syndrome | 3.12E-07 | CD247, FPR1, ITK, LCK, LTF, MNDA, NFE2, S100A12 | 8 |
| Infectious Disease | infectious disorder | infectious disorder | 7.89E-05 | CD52, CD247, CX3CR1, FCGR2A, FPR1, HCK, IL7R, IL8RB, ITK, LCK, LTB, LTF, LYZ, MNDA, NFE2, PF4, PRF1, PTPRC, S100A12, SLC4A1, TRIM58 | 21 |
| Infectious Disease | infectious disorder | infectious disorder of organ | 4.84E-04 | IL8RB, LTB, LYZ | 3 |
| Infectious Disease | herpetic stromal keratitis | herpetic stromal keratitis of eye | 5.54E-03 | IL8RB | 1 |
| Infectious Disease | infection | infection of leukocytes | 9.03E-03 | FPR1, LTF | 2 |
| Infectious Disease | infection | infection of mice | 1.02E-02 | CX3CR1, ITK, PRF1 | 3 |
| Respiratory Disease | severe acute respiratory syndrome | severe acute respiratory syndrome | 3.12E-07 | CD247, FPR1, ITK, LCK, LTF, MNDA, NFE2, S100A12 | 8 |
| Respiratory Disease | respiratory disorder | respiratory disorder | 8.38E-06 | CD247, CD8A, CX3CR1, FPR1, HCK, IL8RB, ITK, LCK, LTF, LYZ, MNDA, NFE2, S100A12 | 13 |
| Respiratory Disease | disease | disease of lung | 6.02E-05 | CD8A, HCK, ITK, LGALS3, LYZ, PRF1 | 6 |
| Respiratory Disease | experimentally induced inflammation | experimentally induced inflammation of airway | 1.80E-04 | CD2, CD48 | 2 |
| Respiratory Disease | chronic obstructive pulmonary disease | chronic obstructive pulmonary disease | 1.98E-04 | CD8A, HCK, IL8RB, LTF, LYZ | 5 |
| Respiratory Disease | emphysema | emphysema of lung | 1.93E-03 | CD8A, HCK | 2 |
| Respiratory Disease | chronic bronchitis | chronic bronchitis | 1.04E-02 | LTF, LYZ | 2 |
| Respiratory Disease | cell cycle progression | delay in initiation of cell cycle progression of lung cancer cell lines | 1.10E-02 | RASSF5 | 1 |
| Respiratory Disease | growth | growth of murine Lewis lung carcinoma | 1.10E-02 | IL8RB | 1 |
| Cellular Development | developmental process | developmental process of T lymphocytes | 5.27E-07 | C5AR1, CD2, CD247, CD8A, FCGR2A, IL7R, ITK, LCK, PRF1, PTPRC, SATB1, SRGN | 12 |
| Cellular Development | developmental process | developmental process of mononuclear leukocytes | 7.71E-07 | C5AR1, CD2, CD247, CD8A, FCGR2A, IL7R, ITK, LCK, PF4, PRF1, PTPRC, SATB1, SRGN, TNFSF13B | 14 |
| Cellular Development | developmental process | developmental process of lymphocytes | 1.83E-06 | C5AR1, CD2, CD247, CD8A, FCGR2A, IL7R, ITK, LCK, PRF1, PTPRC, SATB1, SRGN, TNFSF13B | 13 |
| Cellular Development | developmental process | developmental process of leukocytes | 2.38E-06 | C5AR1, CD2, CD247, CD8A, FCGR2A, IL7R, ITK, LCK, LTB, PF4, PRF1, PTPRC, SATB1, SRGN, TNFSF13B | 15 |
| Cellular Development | developmental process | developmental process of blood cells | 2.86E-06 | C5AR1, CD2, CD247, CD8A, FCGR2A, IL7R, ITK, LCK, LTB, NFE2, PF4, PRF1, PTPRC, SATB1, SRGN, TNFSF13B | 16 |
| Cellular Development | developmental process | developmental process of thymocytes | 2.60E-05 | CD2, CD247, IL7R, LCK, PTPRC, SATB1 | 6 |
| Cellular Development | developmental process | arrest in developmental process of blood cells | 8.64E-04 | LCK, NFE2, PTPRC | 3 |
| Cellular Development | development | development of leukocytes | 3.22E-06 | CD247, CD8A, FCGR2A, IL7R, ITK, LCK, LTB, PTPRC, SATB1, TNFSF13B | 10 |
| Cellular Development | development | development of T lymphocytes | 7.20E-06 | CD247, CD8A, FCGR2A, IL7R, ITK, LCK, PTPRC, SATB1 | 8 |
| Cellular Development | development | development of lymphocytes | 7.52E-06 | CD247, CD8A, FCGR2A, IL7R, ITK, LCK, PTPRC, SATB1, TNFSF13B | 9 |
| Cellular Development | development | development of intraepithelial T lymphocytes | 2.99E-04 | LCK, PTPRC | 2 |
| Cellular Development | development | development of cells | 6.05E-04 | CD247, CD8A, EPB42, FCGR2A, FGR, HCK, IL7R, IL8RB, ITK, LCK, LGALS3, LTB, PTPRC, SATB1, SELL, TNFSF13B | 16 |
| Cellular Development | development | development of alpha-beta T lymphocytes | 6.24E-04 | FCGR2A, LCK | 2 |
| Cellular Development | development | development of thymocytes | 4.89E-03 | IL7R, PTPRC, SATB1 | 3 |
| Cellular Development | development | development of transitional type 1 B lymphocytes | 5.54E-03 | TNFSF13B | 1 |

¬© 2000-2009 Ingenuity Systems, Inc. All rights reserved.

| Category | Function | Function Annotation | P-value | Molecules | # Molecules |
|---|---|---|---|---|---|
| Cellular Development | development | development of pre-B lymphocytes | 1.04E-02 | IL7R, TNFSF13B | 2 |
| Cellular Development | development | arrest in development of alpha-beta T lymphocytes | 1.10E-02 | LCK | 1 |
| Cellular Development | maturation | maturation of thymocytes | 7.08E-05 | CD247, LCK, PTPRC | 3 |
| Cellular Development | maturation | maturation of lymphocytes | 7.48E-05 | CD247, ITK, LCK, PTPRC, TNFSF13B | 5 |
| Cellular Development | maturation | maturation of T lymphocytes | 1.09E-04 | CD247, ITK, LCK, PTPRC | 4 |
| Cellular Development | maturation | maturation of blood cells | 6.74E-04 | CD247, ITK, LCK, NFE2, PTPRC, TNFSF13B | 6 |
| Cellular Development | maturation | arrest in maturation of megakaryocytes | 5.54E-03 | NFE2 | 1 |
| Cellular Development | expansion | expansion of lymphocytes | 2.86E-04 | C5AR1, CD2, IL7R, PRF1, SRGN | 5 |
| Cellular Development | expansion | expansion of T lymphocytes | 1.43E-03 | C5AR1, CD2, PRF1, SRGN | 4 |
| Cellular Development | growth | growth of leukocytes | 3.65E-04 | C5AR1, CD2, IL7R, PF4, PRF1, SRGN | 6 |
| Cellular Development | differentiation | differentiation of gamma-delta T lymphocytes | 4.48E-04 | LCK, PTPRC | 2 |
| Cellular Development | differentiation | differentiation of mononuclear leukocytes | 4.98E-04 | CD2, CD8A, IL7R, ITK, LCK, PF4, PTPRC, TNFSF13B | 8 |
| Cellular Development | differentiation | differentiation of T lymphocytes | 1.02E-03 | CD2, CD8A, IL7R, ITK, LCK, PTPRC | 6 |
| Cellular Development | differentiation | differentiation of lymphocytes | 1.22E-03 | CD2, CD8A, IL7R, ITK, LCK, PTPRC, TNFSF13B | 7 |
| Cellular Development | differentiation | differentiation of blood cells | 1.70E-03 | CD2, CD8A, IL7R, ITK, LCK, NFE2, PF4, PTPRC, TNFSF13B | 9 |
| Cellular Development | differentiation | differentiation of transitional type 2 B lymphocytes | 5.54E-03 | TNFSF13B | 1 |
| Cellular Development | differentiation | differentiation of megakaryocytes | 7.72E-03 | NFE2, PF4 | 2 |
| Cellular Development | differentiation | arrest in differentiation of cord blood cells | 1.10E-02 | PTPRC | 1 |
| Cellular Development | differentiation | arrest in differentiation of erythroblasts | 1.10E-02 | PTPRC | 1 |
| Cellular Development | morphogenesis | morphogenesis of cells | 2.52E-03 | CD247, EPB42, FCGR2A, FGR, HCK, IL7R, IL8RB, LGALS3, SELL | 9 |
| Hematological Disease | apoptosis | apoptosis of leukocytes | 5.93E-07 | CD2, CD247, IL7R, ITK, LCK, LGALS3, NAMPT, PF4, PRF1, PTPRC, SATB1, TNFSF13B | 12 |
| Hematological Disease | apoptosis | apoptosis of mononuclear leukocytes | 2.41E-06 | CD2, CD247, IL7R, ITK, LCK, PF4, PRF1, PTPRC, SATB1, TNFSF13B | 10 |
| Hematological Disease | apoptosis | apoptosis of lymphocytes | 1.42E-05 | CD2, CD247, IL7R, ITK, LCK, PRF1, PTPRC, SATB1, TNFSF13B | 9 |
| Hematological Disease | apoptosis | apoptosis of T lymphocytes | 1.78E-05 | CD2, CD247, IL7R, ITK, LCK, PRF1, PTPRC, SATB1 | 8 |
| Hematological Disease | apoptosis | apoptosis of leukemia cell lines | 2.34E-05 | FGR, LCK, LGALS3, PRF1, PTPRC, RASSF5, S100A8, TNFSF13B | 8 |
| Hematological Disease | apoptosis | apoptosis of intraepithelial T lymphocytes | 5.54E-03 | PTPRC | 1 |
| Hematological Disease | apoptosis | apoptosis of hematopoietic progenitor cells | 7.72E-03 | PF4, PRF1 | 2 |
| Hematological Disease | apoptosis | apoptosis of B lymphocytes | 1.06E-02 | PRF1, PTPRC, TNFSF13B | 3 |
| Hematological Disease | apoptosis | apoptosis of naive T lymphocytes | 1.10E-02 | PRF1 | 1 |
| Hematological Disease | apoptosis | apoptosis of CD34+ cells | 1.65E-02 | PF4 | 1 |
| Hematological Disease | cytolysis | cytolysis of T lymphocytes | 6.51E-07 | LCK, PRF1, PTPRC | 3 |
| Hematological Disease | cytolysis | cytolysis of intraepithelial T lymphocytes | 3.03E-05 | LCK, PTPRC | 2 |
| Hematological Disease | cytolysis | cytolysis of myeloid cells | 5.54E-03 | PRF1 | 1 |
| Hematological Disease | cell death | cell death of leukemia cell lines | 8.69E-07 | FGR, GZMH, GZMK, LCK, LGALS3, PRF1, PTPRC, RASSF5, S100A8, TNFSF13B | 10 |
| Hematological Disease | hematological disorder | hematological disorder | 1.12E-06 | CD2, CD52, CD247, CX3CR1, EPB42, EPB49, FCGR2A, FCGR3B, HBD, IL7R, ITK, LCK, NFE2, PRF1, SATB1, SELL, SLC4A1, XK | 18 |
| Hematological Disease | hematological disorder | hematological disorder of mammalia | 5.99E-05 | CX3CR1, EPB49, FCGR2A, HBD, IL7R, NFE2, PRF1, SLC4A1, XK | 9 |
| Hematological Disease | hematological disorder | hematological disorder of mice | 2.01E-04 | CX3CR1, EPB49, FCGR2A, HBD, IL7R, NFE2, PRF1, SLC4A1 | 8 |
| Hematological Disease | spherocytosis | spherocytosis | 1.62E-06 | EPB42, EPB49, SLC4A1 | 3 |
| Hematological Disease | spherocytosis | spherocytosis of red blood cells | 3.03E-05 | EPB49, SLC4A1 | 2 |
| Hematological Disease | activation | activation of leukemia cell lines | 1.18E-05 | CD247, CD8A, FCGR2A, IL8RB | 4 |
| Hematological Disease | anemia | anemia of mice | 5.11E-05 | EPB49, FCGR2A, HBD, IL7R, SLC4A1 | 5 |
| Hematological Disease | anemia | anemia | 1.39E-04 | CX3CR1, EPB49, FCGR2A, HBD, IL7R, SLC4A1 | 6 |
| Hematological Disease | hereditary spherocytosis | hereditary spherocytosis | 9.05E-05 | EPB42, SLC4A1 | 2 |
| Hematological Disease | adhesion | adhesion of myeloid leukemia cells | 2.99E-04 | AMICA1, SELL | 2 |
| Hematological Disease | killing | killing of lymphocytes | 6.57E-04 | CD48, LGALS3, PRF1 | 3 |
| Hematological Disease | killing | killing of natural killer cells | 2.27E-03 | CD48, PRF1 | 2 |
| Hematological Disease | autoimmune hemolytic anemia | autoimmune hemolytic anemia of mice | 1.06E-03 | FCGR2A, IL7R | 2 |
| Hematological Disease | detachment | detachment of leukocytes | 1.06E-03 | PTPRC, SELL | 2 |
| Hematological Disease | detachment | detachment of macrophages | 1.10E-02 | PTPRC | 1 |
| Hematological Disease | hemolytic anemia | hemolytic anemia | 1.29E-03 | CX3CR1, FCGR2A, IL7R | 3 |
| Hematological Disease | cell movement | cell movement of leukemia cell lines | 2.36E-03 | FPR1, LCK, SELL | 3 |
| Hematological Disease | disease | disease of leukocytes | 3.14E-03 | FPR1, LTF, PF4 | 3 |
| Hematological Disease | disease | disease of mononuclear leukocytes | 1.19E-02 | FPR1, PF4 | 2 |
| Hematological Disease | binding | binding of leukemia cell lines | 3.48E-03 | IL8RB, LCK, SELL | 3 |
| Hematological Disease | lysis | lysis of red blood cells | 5.39E-03 | PRF1, SLC4A1 | 2 |
| Hematological Disease | clearance | clearance of red blood cells | 5.54E-03 | FCGR2A | 1 |
| Hematological Disease | immunosuppression | immunosuppression of T lymphocytes | 5.54E-03 | PF4 | 1 |

¬© 2000-2009 Ingenuity Systems, Inc. All rights reserved.

| Category | Function | Function Annotation | P-value | Molecules | # Molecules |
|---|---|---|---|---|---|
| Hematological Disease | hemorrhage | hemorrhage of mice | 7.72E-03 | FCGR2A, NFE2 | 2 |
| Hematological Disease | infection | infection of leukocytes | 9.03E-03 | FPR1, LTF | 2 |
| Hematological Disease | cytotoxic reaction | cytotoxic reaction of leukemia cell lines | 1.10E-02 | LCK | 1 |
| Hematological Disease | deletion | delay in initiation of deletion of T lymphocytes | 1.10E-02 | CD2 | 1 |
| Hematological Disease | pH | pH of leukemia cell lines | 1.10E-02 | LCK | 1 |
| Hematological Disease | recruitment | recruitment of leukemia cell lines | 1.10E-02 | IL8RB | 1 |
| Hematological Disease | activation-induced cell death | activation-induced cell death of T lymphocytes | 1.12E-02 | ITK, PRF1 | 2 |
| Hematological Disease | lymphocytic leukemia | lymphocytic leukemia | 1.27E-02 | CD2, CD52, CD247, LCK | 4 |
| Hematological Disease | hematologic cancer | hematologic cancer | 1.41E-02 | CD2, CD52, CD247, FGR, LCK, PF4 | 6 |
| Hematological Disease | Waldenstrom's macroglobulinemia | Waldenstrom's macroglobulinemia | 1.52E-02 | CD52, CD247, PRF1, SATB1 | 4 |
| Cancer | cell death | cell death of leukemia cell lines | 8.69E-07 | FGR, GZMH, GZMK, LCK, LGALS3, PRF1, PTPRC, RASSF5, S100A8, TNFSF13B | 10 |
| Cancer | cell death | cell death of tumor cell lines | 1.10E-02 | CD48, FGR, GZMH, GZMK, LCK, LGALS3, LTB, NAMPT, PRF1, PTPRC, RASSF5, S100A8, TNFSF13B | 13 |
| Cancer | cell death | cell death of lymphoma cell lines | 1.24E-02 | CD48, LGALS3, PTPRC, TNFSF13B | 4 |
| Cancer | activation | activation of leukemia cell lines | 1.18E-05 | CD247, CD8A, FCGR2A, IL8RB | 4 |
| Cancer | papillary thyroid carcinoma | papillary thyroid carcinoma | 1.81E-05 | CD48, CST7, LGALS3, LTB, PTPRC | 5 |
| Cancer | apoptosis | apoptosis of leukemia cell lines | 2.34E-05 | FGR, LCK, LGALS3, PRF1, PTPRC, RASSF5, S100A8, TNFSF13B | 8 |
| Cancer | binding | binding of tumor cell lines | 2.73E-04 | FCGR2A, IL8RB, LCK, LGALS3, LTF, SELL | 6 |
| Cancer | binding | binding of leukemia cell lines | 3.48E-03 | IL8RB, LCK, SELL | 3 |
| Cancer | adhesion | adhesion of myeloid leukemia cells | 2.99E-04 | AMICA1, SELL | 2 |
| Cancer | prostate cancer | prostate cancer | 1.15E-03 | CD52, CYTIP, IL7R, ITK, LCK, LTF, LYZ, PTPRC, S100A8 | 9 |
| Cancer | disease | disease of brain cancer cell lines | 1.32E-03 | FPR1, IL8RB | 2 |
| Cancer | cytolysis | cytolysis of tumor cells | 2.27E-03 | FCGR2A, PRF1 | 2 |
| Cancer | thymic lymphoma | thymic lymphoma | 2.27E-03 | LCK, PTPRC | 2 |
| Cancer | cell movement | cell movement of leukemia cell lines | 2.36E-03 | FPR1, LCK, SELL | 3 |
| Cancer | non-hodgkin lymphoma | non-hodgkin lymphoma | 4.31E-03 | CD2, CD52, CD247, LCK, PRF1 | 5 |
| Cancer | lymphoma | lymphoma | 4.54E-03 | CD2, CD52, CD247, LCK, PRF1, PTPRC | 6 |
| Cancer | size | size of glioma | 5.54E-03 | PF4 | 1 |
| Cancer | size | size of lymphoma cell lines | 5.54E-03 | PTPRC | 1 |
| Cancer | peripheral T-cell lymphoma | peripheral T-cell lymphoma | 9.71E-03 | CD2, CD52 | 2 |
| Cancer | cell cycle progression | delay in initiation of cell cycle progression of carcinoma cell lines | 1.10E-02 | RASSF5 | 1 |
| Cancer | cell cycle progression | delay in initiation of cell cycle progression of lung cancer cell lines | 1.10E-02 | RASSF5 | 1 |
| Cancer | cell rolling | cell rolling of lymphoma cell lines | 1.10E-02 | SELL | 1 |
| Cancer | cytotoxic reaction | cytotoxic reaction of leukemia cell lines | 1.10E-02 | LCK | 1 |
| Cancer | development | development of thymic lymphoma | 1.10E-02 | PTPRC | 1 |
| Cancer | growth | growth of murine Lewis lung carcinoma | 1.10E-02 | IL8RB | 1 |
| Cancer | lymphomagenesis | lymphomagenesis of natural killer cell lymphoma | 1.10E-02 | PRF1 | 1 |
| Cancer | pH | pH of leukemia cell lines | 1.10E-02 | LCK | 1 |
| Cancer | recruitment | recruitment of leukemia cell lines | 1.10E-02 | IL8RB | 1 |
| Cancer | lymphocytic leukemia | lymphocytic leukemia | 1.27E-02 | CD2, CD52, CD247, LCK | 4 |
| Cancer | hematologic cancer | hematologic cancer | 1.41E-02 | CD2, CD52, CD247, FGR, LCK, PF4 | 6 |
| Cancer | T-cell non-hodgkin lymphoma | T-cell non-hodgkin lymphoma | 1.44E-02 | CD2, CD52, PRF1 | 3 |
| Post-Translational Modification | tyrosine phosphorylation | tyrosine phosphorylation of protein | 1.53E-06 | CD2, CD48, CD247, CD8A, FCGR2A, HCK, LCK, PTPRC, SELL | 9 |
| Post-Translational Modification | phosphorylation | phosphorylation of protein | 4.95E-05 | CD2, CD48, CD247, CD8A, FCGR2A, HCK, LCK, LRRK2, PTPRC, SELL | 10 |
| Post-Translational Modification | moiety attachment | moiety attachment of protein | 3.46E-04 | CD2, CD48, CD247, CD8A, FCGR2A, HCK, LCK, LRRK2, PTPRC, SATB1, SELL | 11 |
| Post-Translational Modification | modification | modification of protein | 3.27E-03 | CD2, CD48, CD247, CD8A, FCGR2A, HCK, LCK, LRRK2, PTPRC, SATB1, SELL, SSH2 | 12 |
| Organismal Injury and Abnormalities | spherocytosis | spherocytosis | 1.62E-06 | EPB42, EPB49, SLC4A1 | 3 |
| Organismal Injury and Abnormalities | spherocytosis | spherocytosis of red blood cells | 3.03E-05 | EPB49, SLC4A1 | 2 |
| Organismal Injury and Abnormalities | hereditary spherocytosis | hereditary spherocytosis | 9.05E-05 | EPB42, SLC4A1 | 2 |
| Organismal Injury and Abnormalities | cytosis | cytosis | 3.77E-04 | EPB42, EPB49, LCK, NFE2, SELL, SLC4A1 | 6 |
| Organismal Injury and Abnormalities | damage | damage of organ | 4.50E-03 | FCGR2A, FGR, HCK, IL8RB, PRF1 | 5 |
| Organismal Injury and Abnormalities | edema | edema of joint | 5.54E-03 | TNFAIP6 | 1 |
| Organismal Injury and Abnormalities | necrosis | necrosis of brain | 5.54E-03 | PRF1 | 1 |
| Organismal Injury and Abnormalities | hemorrhage | hemorrhage of mice | 7.72E-03 | FCGR2A, NFE2 | 2 |

| | | | | ¬© 2000-2009 Ingenuity Systems, Inc. All rights reserved. | |
|---|---|---|---|---|---|
| **Category** | **Function** | **Function Annotation** | **P-value** | **Molecules** | **# Molecules** |
| Cellular Growth and Proliferation | proliferation | proliferation of blood cells | 1.72E-06 | CD2, CD48, CD247, CD8A, HCK, IL7R, ITK, LCK, NFE2, PF4, PIK3AP1, PTPRC, SATB1, SRGN, TNFSF13B | 15 |
| Cellular Growth and Proliferation | proliferation | proliferation of lymphocytes | 1.10E-05 | CD2, CD48, CD247, CD8A, HCK, IL7R, ITK, LCK, PIK3AP1, PTPRC, SATB1, SRGN, TNFSF13B | 13 |
| Cellular Growth and Proliferation | proliferation | proliferation of T lymphocytes | 2.86E-05 | CD2, CD48, CD247, CD8A, IL7R, ITK, LCK, PTPRC, SATB1, SRGN, TNFSF13B | 11 |
| Cellular Growth and Proliferation | proliferation | proliferation of normal cells | 2.27E-04 | CD2, CD48, CD247, CD8A, HCK, IL7R, IL8RB, ITK, LCK, LGALS3, NFE2, PF4, PIK3AP1, PROK2, PTPRC, SATB1, SRGN, TNFSF13B | 18 |
| Cellular Growth and Proliferation | proliferation | proliferation of hematopoietic progenitor cells | 4.83E-04 | IL7R, NFE2, PF4, PTPRC | 4 |
| Cellular Growth and Proliferation | proliferation | proliferation of B lymphocytes | 4.94E-04 | CD8A, HCK, IL7R, PIK3AP1, PTPRC, TNFSF13B | 6 |
| Cellular Growth and Proliferation | proliferation | proliferation of eukaryotic cells | 1.94E-03 | CD2, CD48, CD247, CD8A, HCK, IL7R, IL8RB, ITK, LCK, LGALS3, NFE2, PDZK1IP1, PF4, PIK3AP1, PROK2, PTPRC, S100A8, SATB1, SRGN, TNFSF13B | 20 |
| Cellular Growth and Proliferation | proliferation | proliferation of memory T lymphocytes | 5.94E-03 | CD2, IL7R | 2 |
| Cellular Growth and Proliferation | proliferation | proliferation of peripheral blood lymphocytes | 8.36E-03 | ITK, TNFSF13B | 2 |
| Cellular Growth and Proliferation | proliferation | proliferation of cells | 1.29E-02 | CD2, CD48, CD247, CD8A, HCK, IL7R, IL8RB, ITK, LCK, LGALS3, NAMPT, NFE2, PDZK1IP1, PF4, PIK3AP1, PROK2, PTPRC, S100A8, SATB1, SRGN, TNFSF13B | 21 |
| Cellular Growth and Proliferation | proliferation | proliferation of leukocyte cell lines | 1.64E-02 | CD8A, HCK, IL7R, PTPRC | 4 |
| Cellular Growth and Proliferation | stimulation | stimulation of eukaryotic cells | 1.50E-04 | C5AR1, CD2, CD8A, FGD3, PTPRC, TNFSF13B | 6 |
| Cellular Growth and Proliferation | stimulation | stimulation of normal cells | 7.90E-04 | C5AR1, CD2, FGD3, PTPRC, TNFSF13B | 5 |
| Cellular Growth and Proliferation | stimulation | stimulation of leukocytes | 1.31E-03 | C5AR1, CD2, PTPRC, TNFSF13B | 4 |
| Cellular Growth and Proliferation | stimulation | stimulation of lymphocytes | 2.97E-03 | CD2, PTPRC, TNFSF13B | 3 |
| Cellular Growth and Proliferation | expansion | expansion of lymphocytes | 2.86E-04 | C5AR1, CD2, IL7R, PRF1, SRGN | 5 |
| Cellular Growth and Proliferation | expansion | expansion of normal cells | 4.80E-04 | C5AR1, CD2, IL7R, PRF1, SRGN, TNFAIP6 | 6 |
| Cellular Growth and Proliferation | expansion | expansion of T lymphocytes | 1.43E-03 | C5AR1, CD2, PRF1, SRGN | 4 |
| Cellular Growth and Proliferation | growth | growth of leukocytes | 3.65E-04 | C5AR1, CD2, IL7R, PF4, PRF1, SRGN | 6 |
| Cellular Growth and Proliferation | growth | growth of normal cells | 1.36E-02 | C5AR1, CD2, IL7R, PF4, PRF1, SRGN, TNFAIP6 | 7 |
| Cellular Growth and Proliferation | assembly | assembly of endothelial cells | 5.54E-03 | PF4 | 1 |
| Cellular Growth and Proliferation | formation | formation of memory B cells | 5.54E-03 | LTB | 1 |
| Cellular Growth and Proliferation | formation | formation of lymphatic system cells | 1.35E-02 | LTB, NFE2 | 2 |
| Cellular Growth and Proliferation | generation | generation of pro-B lymphocytes | 5.54E-03 | IL7R | 1 |
| Cellular Growth and Proliferation | generation | generation of leukocytes | 7.99E-03 | IL7R, ITK, LTB | 3 |
| Cellular Growth and Proliferation | immunosuppression | immunosuppression of T lymphocytes | 5.54E-03 | PF4 | 1 |
| Inflammatory Disease | inflammatory disorder | inflammatory disorder | 2.06E-06 | AQP9, C5AR1, CD2, CD48, CD52, CD247, CD8A, CX3CR1, CYP4F3, FAM129A, FCGR2A, FCN1, HBD, HCK, IL7R, IL8RB, ITK, KCNJ15, LCK, LGALS3, LRRK2, LTB, LTF, LYZ, NAMPT, P2RY13, PIK3AP1, PRF1, PTPRC, S100A8, S100A12, SLC25A37, SORL1, TAGAP, TNFAIP6, TNFSF13B, VNN2 | 37 |
| Inflammatory Disease | inflammatory disorder | inflammatory disorder of mice | 4.53E-05 | C5AR1, CD2, FCGR2A, HCK, ITK, LGALS3, LTB, LTF, PRF1, PTPRC | 10 |
| Inflammatory Disease | inflammatory disorder | inflammatory disorder of organ | 7.85E-04 | CD8A, HCK, IL8RB, TNFSF13B | 4 |
| Inflammatory Disease | polyarticular juvenile rheumatoid arthritis | polyarticular juvenile rheumatoid arthritis | 7.32E-06 | P2RY13, PTPRC, S100A8, S100A12, SORL1, TNFSF13B | 6 |
| Inflammatory Disease | rheumatic disease | rheumatic disease | 1.68E-05 | AQP9, C5AR1, CYP4F3, FAM129A, FCGR2A, FCN1, HCK, IL7R, IL8RB, KCNJ15, LCK, LRRK2, LTB, LTF, LYZ, NAMPT, P2RY13, PIK3AP1, PTPRC, S100A8, S100A12, SORL1, TAGAP, TNFAIP6, TNFSF13B | 25 |
| Inflammatory Disease | arthritis | arthritis | 2.46E-05 | AQP9, C5AR1, CYP4F3, FAM129A, FCGR2A, FCN1, HCK, IL7R, IL8RB, KCNJ15, LRRK2, LTB, LTF, LYZ, NAMPT, P2RY13, PIK3AP1, PTPRC, S100A8, S100A12, SORL1, TAGAP, TNFAIP6, TNFSF13B | 24 |
| Inflammatory Disease | rheumatoid arthritis | rheumatoid arthritis | 3.81E-05 | AQP9, CYP4F3, FAM129A, FCN1, HCK, IL7R, IL8RB, KCNJ15, LRRK2, LTB, LTF, LYZ, NAMPT, P2RY13, PIK3AP1, PTPRC, S100A8, S100A12, SORL1, TAGAP, TNFAIP6, TNFSF13B | 22 |
| Inflammatory Disease | experimentally induced inflammation | experimentally induced inflammation of airway | 1.80E-04 | CD2, CD48 | 2 |
| Inflammatory Disease | emphysema | emphysema of lung | 1.93E-03 | CD8A, HCK | 2 |
| Inflammatory Disease | systemic lupus erythematosus | systemic lupus erythematosus | 2.20E-03 | FCGR2A, IL7R, PTPRC, TNFSF13B | 4 |
| Inflammatory Disease | systemic lupus erythematosus | systemic lupus erythematosus of animal | 8.36E-03 | PTPRC, TNFSF13B | 2 |
| Inflammatory Disease | airway hyperresponsiveness | airway hyperresponsiveness of mice | 3.14E-03 | C5AR1, ITK, LGALS3 | 3 |
| Inflammatory Disease | herpetic stromal keratitis | herpetic stromal keratitis of eye | 5.54E-03 | IL8RB | 1 |
| Inflammatory Disease | chronic bronchitis | chronic bronchitis | 1.04E-02 | LTF, LYZ | 2 |

¬© 2000-2009 Ingenuity Systems, Inc. All rights reserved.

| Category | Function | Function Annotation | P-value | Molecules | # Molecules |
|---|---|---|---|---|---|
| Infection Mechanism | replication | replication of HIV-1 | 4.69E-06 | HCK, LCK, PTPRC, S100A8, S100A12 | 5 |
| Infection Mechanism | replication | replication of Coxsackievirus B3 | 1.32E-03 | LCK, PTPRC | 2 |
| Infection Mechanism | replication | delay in initiation of replication of HIV-1 | 5.54E-03 | HCK | 1 |
| Infection Mechanism | quantity | quantity of Murine herpesvirus 4 | 5.54E-03 | CD8A | 1 |
| Infection Mechanism | quantity | quantity of human herpesvirus 1 | 1.10E-02 | CD8A | 1 |
| Infection Mechanism | uptake | uptake of Filoviridae | 5.54E-03 | LTF | 1 |
| Infection Mechanism | transmission | transmission of HIV | 1.10E-02 | LTF | 1 |
| Cellular Compromise | degranulation | degranulation of eukaryotic cells | 4.87E-06 | C5AR1, FCGR2A, FCGR3B, FPR1, HCK, ITK, PF4 | 7 |
| Cellular Compromise | degranulation | degranulation of granulocytes | 4.34E-04 | FCGR2A, FCGR3B, PF4 | 3 |
| Cellular Compromise | respiratory burst | respiratory burst | 2.13E-05 | FGR, FPR1, HCK, NCF2, PF4 | 5 |
| Cellular Compromise | deformability | deformability of plasma membrane | 5.54E-03 | SLC4A1 | 1 |
| Cellular Compromise | retraction | retraction of cells | 7.10E-03 | C5AR1, CD2 | 2 |
| Cellular Compromise | damage | damage of cellular membrane | 1.10E-02 | PRF1 | 1 |
| Cellular Compromise | deletion | delay in initiation of deletion of T lymphocytes | 1.10E-02 | CD2 | 1 |
| Cellular Compromise | lysis | lysis of liposome | 1.10E-02 | PRF1 | 1 |
| Cellular Compromise | perturbation | perturbation of mitochondria | 1.10E-02 | PRF1 | 1 |
| Connective Tissue Disorders | polyarticular juvenile rheumatoid arthritis | polyarticular juvenile rheumatoid arthritis | 7.32E-06 | P2RY13, PTPRC, S100A8, S100A12, SORL1, TNFSF13B | 6 |
| Connective Tissue Disorders | rheumatic disease | rheumatic disease | 1.68E-05 | AQP9, C5AR1, CYP4F3, FAM129A, FCGR2A, FCN1, HCK, IL7R, IL8RB, KCNJ15, LCK, LRRK2, LTB, LTF, LYZ, NAMPT, P2RY13, PIK3AP1, PTPRC, S100A8, S100A12, SORL1, TAGAP, TNFAIP6, TNFSF13B | 25 |
| Connective Tissue Disorders | arthritis | arthritis | 2.46E-05 | AQP9, C5AR1, CYP4F3, FAM129A, FCGR2A, FCN1, HCK, IL7R, IL8RB, KCNJ15, LRRK2, LTB, LTF, LYZ, NAMPT, P2RY13, PIK3AP1, PTPRC, S100A8, S100A12, SORL1, TAGAP, TNFAIP6, TNFSF13B | 24 |
| Connective Tissue Disorders | rheumatoid arthritis | rheumatoid arthritis | 3.81E-05 | AQP9, CYP4F3, FAM129A, FCN1, HCK, IL7R, IL8RB, KCNJ15, LRRK2, LTB, LTF, LYZ, NAMPT, P2RY13, PIK3AP1, PTPRC, S100A8, S100A12, SORL1, TAGAP, TNFAIP6, TNFSF13B | 22 |
| Connective Tissue Disorders | systemic lupus erythematosus | systemic lupus erythematosus | 2.20E-03 | FCGR2A, IL7R, PTPRC, TNFSF13B | 4 |
| Connective Tissue Disorders | systemic lupus erythematosus | systemic lupus erythematosus of animal | 8.36E-03 | PTPRC, TNFSF13B | 2 |
| Genetic Disorder | polyarticular juvenile rheumatoid arthritis | polyarticular juvenile rheumatoid arthritis | 7.32E-06 | P2RY13, PTPRC, S100A8, S100A12, SORL1, TNFSF13B | 6 |
| Genetic Disorder | rheumatoid arthritis | rheumatoid arthritis | 3.81E-05 | AQP9, CYP4F3, FAM129A, FCN1, HCK, IL7R, IL8RB, KCNJ15, LRRK2, LTB, LTF, LYZ, NAMPT, P2RY13, PIK3AP1, PTPRC, S100A8, S100A12, SORL1, TAGAP, TNFAIP6, TNFSF13B | 22 |
| Genetic Disorder | autoimmune disease | autoimmune disease | 5.48E-05 | AMICA1, AQP9, CD2, CD52, CX3CR1, CYP4F3, FAM129A, FCGR2A, FCN1, HCK, IL7R, IL8RB, KCNJ15, LRRK2, LTB, LTF, LYZ, NAMPT, P2RY13, PIK3AP1, PTPRC, S100A8, S100A12, SELL, SORL1, TAGAP, TNFAIP6, TNFSF13B | 28 |
| Genetic Disorder | severe combined immunodeficiency | severe combined immunodeficiency | 8.68E-05 | CD247, IL7R, PTPRC | 3 |
| Genetic Disorder | hereditary spherocytosis | hereditary spherocytosis | 9.05E-05 | EPB42, SLC4A1 | 2 |
| Genetic Disorder | autoimmune hemolytic anemia | autoimmune hemolytic anemia of mice | 1.06E-03 | FCGR2A, IL7R | 2 |
| Genetic Disorder | prostate cancer | prostate cancer | 1.15E-03 | CD52, CYTIP, IL7R, ITK, LCK, LTF, LYZ, PTPRC, S100A8 | 9 |
| Genetic Disorder | systemic lupus erythematosus | systemic lupus erythematosus | 2.20E-03 | FCGR2A, IL7R, PTPRC, TNFSF13B | 4 |
| Genetic Disorder | familial CD8 deficiency | familial CD8 deficiency | 5.54E-03 | CD8A | 1 |
| Genetic Disorder | ichthyosis hystrix, Curth Macklin type | ichthyosis hystrix, Curth Macklin type | 5.54E-03 | KRT1 | 1 |
| Genetic Disorder | familial hemophagocytic lymphohistiocytosis, type 2 | familial hemophagocytic lymphohistiocytosis, type 2 | 1.10E-02 | PRF1 | 1 |
| Skeletal and Muscular Disorders | polyarticular juvenile rheumatoid arthritis | polyarticular juvenile rheumatoid arthritis | 7.32E-06 | P2RY13, PTPRC, S100A8, S100A12, SORL1, TNFSF13B | 6 |
| Skeletal and Muscular Disorders | rheumatic disease | rheumatic disease | 1.68E-05 | AQP9, C5AR1, CYP4F3, FAM129A, FCGR2A, FCN1, HCK, IL7R, IL8RB, KCNJ15, LCK, LRRK2, LTB, LTF, LYZ, NAMPT, P2RY13, PIK3AP1, PTPRC, S100A8, S100A12, SORL1, TAGAP, TNFAIP6, TNFSF13B | 25 |
| Skeletal and Muscular Disorders | arthritis | arthritis | 2.46E-05 | AQP9, C5AR1, CYP4F3, FAM129A, FCGR2A, FCN1, HCK, IL7R, IL8RB, KCNJ15, LRRK2, LTB, LTF, LYZ, NAMPT, P2RY13, PIK3AP1, PTPRC, S100A8, S100A12, SORL1, TAGAP, TNFAIP6, TNFSF13B | 24 |
| Skeletal and Muscular Disorders | rheumatoid arthritis | rheumatoid arthritis | 3.81E-05 | AQP9, CYP4F3, FAM129A, FCN1, HCK, IL7R, IL8RB, KCNJ15, LRRK2, LTB, LTF, LYZ, NAMPT, P2RY13, PIK3AP1, PTPRC, S100A8, S100A12, SORL1, TAGAP, TNFAIP6, TNFSF13B | 22 |

¬© 2000-2009 Ingenuity Systems, Inc. All rights reserved.

| Category | Function | Function Annotation | P-value | Molecules | # Molecules |
|---|---|---|---|---|---|
| Skeletal and Muscular Disorders | systemic lupus erythematosus | systemic lupus erythematosus | 2.20E-03 | FCGR2A, IL7R, PTPRC, TNFSF13B | 4 |
| Skeletal and Muscular Disorders | systemic lupus erythematosus | systemic lupus erythematosus of animal | 8.36E-03 | PTPRC, TNFSF13B | 2 |
| Skeletal and Muscular Disorders | skeletal and muscular disorder | skeletal and muscular disorder | 2.24E-03 | AQP9, C5AR1, CYP4F3, EVI2B, FAM129A, FCGR2A, FCN1, FPR1, HCK, IL7R, IL8RB, KCNJ15, LCK, LRRK2, LTB, LTF, LYZ, MYL4, NAMPT, P2RY13, PIK3AP1, PTPRC, S100A8, S100A12, SLC25A37, SORL1, TAGAP, TNFAIP6, TNFSF13B, XK | 30 |
| Skeletal and Muscular Disorders | edema | edema of joint | 5.54E-03 | TNFAIP6 | 1 |
| Cellular Function and Maintenance | phagocytosis | phagocytosis of blood cells | 1.53E-05 | FCGR2A, FCGR3B, FGR, HCK, PF4 | 5 |
| Cellular Function and Maintenance | phagocytosis | phagocytosis of red blood cells | 2.02E-04 | FCGR2A, FGR, HCK | 3 |
| Cellular Function and Maintenance | phagocytosis | phagocytosis of leukocyte cell lines | 2.99E-04 | FCGR2A, FGR | 2 |
| Cellular Function and Maintenance | phagocytosis | phagocytosis of monocytes | 2.99E-04 | FCGR2A, PF4 | 2 |
| Cellular Function and Maintenance | phagocytosis | phagocytosis of leukocytes | 1.50E-03 | FCGR2A, FCGR3B, PF4 | 3 |
| Cellular Function and Maintenance | phagocytosis | phagocytosis of eosinophils | 5.54E-03 | FCGR2A | 1 |
| Cellular Function and Maintenance | phagocytosis | phagocytosis of gonadal cell lines | 5.54E-03 | FCGR2A | 1 |
| Cellular Function and Maintenance | phagocytosis | phagocytosis of neutrophils | 5.94E-03 | FCGR2A, FCGR3B | 2 |
| Cellular Function and Maintenance | contact growth inhibition | contact growth inhibition of T lymphocytes | 9.05E-05 | LCK, PTPRC | 2 |
| Cellular Function and Maintenance | respiratory burst | respiratory burst of leukocytes | 9.98E-05 | FGR, FPR1, HCK, PF4 | 4 |
| Cellular Function and Maintenance | respiratory burst | respiratory burst of neutrophils | 7.22E-04 | FGR, FPR1, HCK | 3 |
| Cellular Function and Maintenance | respiratory burst | respiratory burst of macrophages | 8.30E-04 | FGR, HCK | 2 |
| Cellular Function and Maintenance | presence | presence of neutrophils | 1.80E-04 | FGR, HCK | 2 |
| Cellular Function and Maintenance | presence | presence of blood cells | 2.67E-04 | FCGR2A, FGR, HCK | 3 |
| Cellular Function and Maintenance | function | function of cells | 6.16E-04 | FCGR2A, LTB, LTF, PRF1, TNFSF13B | 5 |
| Cellular Function and Maintenance | function | function of leukocytes | 7.85E-04 | FCGR2A, LTB, PRF1, TNFSF13B | 4 |
| Cellular Function and Maintenance | function | function of lymphocytes | 3.14E-03 | FCGR2A, PRF1, TNFSF13B | 3 |
| Cellular Function and Maintenance | function | function of antigen presenting cells | 9.03E-03 | LTB, TNFSF13B | 2 |
| Cellular Function and Maintenance | assembly | assembly of endothelial cells | 5.54E-03 | PF4 | 1 |
| Cellular Function and Maintenance | clearance | clearance of red blood cells | 5.54E-03 | FCGR2A | 1 |
| Cellular Function and Maintenance | co-localization | co-localization of red blood cells | 5.54E-03 | FCGR2A | 1 |
| Cellular Function and Maintenance | co-localization | co-localization of F-actin | 1.10E-02 | FCGR2A | 1 |
| Cellular Function and Maintenance | elimination | elimination of T lymphocytes | 1.10E-02 | PRF1 | 1 |
| Cell Morphology | cell spreading | cell spreading of blood platelets | 2.61E-05 | FCGR2A, FGR, HCK | 3 |
| Cell Morphology | cell spreading | cell spreading of blood cells | 9.11E-05 | CD247, FCGR2A, FGR, HCK | 4 |
| Cell Morphology | cell spreading | cell spreading of normal cells | 1.00E-04 | CD247, FCGR2A, FGR, HCK, IL8RB | 5 |
| Cell Morphology | cell spreading | cell spreading of leukocytes | 1.02E-03 | CD247, FGR, HCK | 3 |
| Cell Morphology | cell spreading | cell spreading of neutrophils | 1.93E-03 | FGR, HCK | 2 |
| Cell Morphology | shape change | shape change of blood cells | 4.47E-05 | CD247, FCGR2A, FGR, HCK, SELL | 5 |
| Cell Morphology | shape change | shape change of neutrophils | 4.49E-05 | FGR, HCK, SELL | 3 |
| Cell Morphology | shape change | shape change of leukocytes | 3.14E-04 | CD247, FGR, HCK, SELL | 4 |
| Cell Morphology | shape change | shape change of normal cells | 5.09E-04 | CD247, FCGR2A, FGR, HCK, IL8RB, SELL | 6 |
| Cell Morphology | transmembrane potential | transmembrane potential of mitochondria | 1.58E-03 | CD2, GZMK, LCK, PRF1, SRGN | 5 |
| Cell Morphology | morphology | morphology of red blood cells | 2.27E-03 | HBD, NFE2 | 2 |
| Cell Morphology | morphology | morphology of blood cells | 3.31E-03 | HBD, LGALS3, NFE2 | 3 |
| Cell Morphology | morphogenesis | morphogenesis of cells | 2.52E-03 | CD247, EPB42, FCGR2A, FGR, HCK, IL7R, IL8RB, LGALS3, SELL | 9 |
| Cell Morphology | area | area of endothelial cell lines | 5.54E-03 | C5AR1 | 1 |
| Cell Morphology | deformability | deformability of plasma membrane | 5.54E-03 | SLC4A1 | 1 |
| Cell Morphology | negatively-charged | negatively-charged of cell surface | 5.54E-03 | LTF | 1 |
| Cell Morphology | permeabilization | permeabilization of plasma membrane | 5.54E-03 | PRF1 | 1 |
| Cell Morphology | size | size of lymphoma cell lines | 5.54E-03 | PTPRC | 1 |
| Cell Morphology | structural integrity | structural integrity of mitochondria | 5.54E-03 | LGALS3 | 1 |
| Cell Morphology | remodeling | remodeling of cytoskeleton | 5.94E-03 | C5AR1, VNN2 | 2 |
| Cell Morphology | pH | pH of leukemia cell lines | 1.10E-02 | LCK | 1 |
| Cell Morphology | polarization | polarization of cytoskeleton | 1.10E-02 | CD2 | 1 |
| Free Radical Scavenging | release | release of superoxide | 2.78E-05 | FCGR2A, FGR, HCK, NCF2 | 4 |
| Free Radical Scavenging | production | production of superoxide | 8.94E-05 | FGR, FPR1, HCK, LTF, NCF2 | 5 |
| Free Radical Scavenging | production | production of reactive oxygen species | 1.40E-03 | CD52, GZMK, NCF2, PRF1, SESN3 | 5 |
| Hypersensitivity Response | cell movement | cell movement of eosinophils | 7.94E-05 | CD2, CD48, FPR1, ITK, LGALS3 | 5 |
| Hypersensitivity Response | infiltration | infiltration of eosinophils | 1.61E-03 | CD2, CD48, ITK | 3 |
| Hypersensitivity Response | quantity | quantity of eosinophils | 1.96E-03 | C5AR1, CD8A, LGALS3 | 3 |
| Hypersensitivity Response | influx | influx of eosinophils | 5.54E-03 | ITK | 1 |
| Hypersensitivity Response | phagocytosis | phagocytosis of eosinophils | 5.54E-03 | FCGR2A | 1 |

**Highly Expressed in MBC versus CVS** **Table S1F**

¬© 2000-2009 Ingenuity Systems, Inc. All rights reserved.

| Category | Function | Function Annotation | P-value | Molecules | # Molecules |
|---|---|---|---|---|---|
| Cellular Assembly and Organization | formation | formation of phagocytic cups | 9.05E-05 | FGR, HCK | 2 |
| Cellular Assembly and Organization | rearrangement | rearrangement of microtubule organizing centers | 1.80E-04 | CD247, LCK | 2 |
| Cellular Assembly and Organization | binding | binding of microvilli | 5.54E-03 | LCK | 1 |
| Cellular Assembly and Organization | binding | binding of actin cytoskeleton | 1.65E-02 | CD2 | 1 |
| Cellular Assembly and Organization | maturation | maturation of secretory granules | 5.54E-03 | SRGN | 1 |
| Cellular Assembly and Organization | recruitment | recruitment of membrane rafts | 5.54E-03 | CD48 | 1 |
| Cellular Assembly and Organization | structural integrity | structural integrity of mitochondria | 5.54E-03 | LGALS3 | 1 |
| Cellular Assembly and Organization | remodeling | remodeling of cytoskeleton | 5.94E-03 | C5AR1, VNN2 | 2 |
| Cellular Assembly and Organization | attachment | attachment of actin cytoskeleton | 1.10E-02 | EPB42 | 1 |
| Cellular Assembly and Organization | co-localization | co-localization of F-actin | 1.10E-02 | FCGR2A | 1 |
| Cellular Assembly and Organization | perturbation | perturbation of mitochondria | 1.10E-02 | PRF1 | 1 |
| Cellular Assembly and Organization | polarization | polarization of cytoskeleton | 1.10E-02 | CD2 | 1 |
| Cellular Assembly and Organization | polymerization | polymerization of cytoskeleton | 1.10E-02 | ITK | 1 |
| Cardiovascular System Development and Function | adhesion | adhesion of postcapillary venule | 1.80E-04 | FCGR2A, SELL | 2 |
| Cardiovascular System Development and Function | adhesion | adhesion of endothelial cell lines | 6.32E-03 | IL8RB, LGALS3, PF4 | 3 |
| Cardiovascular System Development and Function | adhesion | adhesion of endothelial cells | 9.89E-03 | AMICA1, SELL, SLC4A1 | 3 |
| Cardiovascular System Development and Function | adhesion | adhesion of vascular endothelial tissue | 1.10E-02 | SELL | 1 |
| Cardiovascular System Development and Function | adhesion | adhesion of high endothelial postcapillary venule | 1.65E-02 | SELL | 1 |
| Cardiovascular System Development and Function | binding | binding of endothelial cell lines | 2.75E-04 | KRT1, LGALS3, PF4, SELL | 4 |
| Cardiovascular System Development and Function | area | area of endothelial cell lines | 5.54E-03 | C5AR1 | 1 |
| Cardiovascular System Development and Function | assembly | assembly of endothelial cells | 5.54E-03 | PF4 | 1 |
| Cardiovascular System Development and Function | assembly | assembly of microvessel | 5.54E-03 | PF4 | 1 |
| Cardiovascular System Development and Function | chemotaxis | chemotaxis of endothelial cell lines | 5.94E-03 | IL8RB, LGALS3 | 2 |
| Cardiovascular System Development and Function | angiogenesis | angiogenesis of testis | 1.10E-02 | PROK2 | 1 |
| Cardiovascular System Development and Function | proliferation | proliferation of blood vessel | 1.10E-02 | PF4 | 1 |
| Lymphoid Tissue Structure and Development | presence | presence of neutrophils | 1.80E-04 | FGR, HCK | 2 |
| Lymphoid Tissue Structure and Development | development | development of lymphatic system component | 1.68E-03 | EPB42, HBD, IL7R, LTB | 4 |
| Lymphoid Tissue Structure and Development | development | development of peripheral T lymphocyte | 5.54E-03 | IL7R | 1 |
| Lymphoid Tissue Structure and Development | development | development of lymph node | 7.72E-03 | IL7R, LTB | 2 |
| Lymphoid Tissue Structure and Development | morphology | morphology of spleen | 3.03E-03 | LTB, TNFSF13B | 2 |
| Lymphoid Tissue Structure and Development | formation | formation of peripheral lymph node | 5.54E-03 | LTB | 1 |
| Lymphoid Tissue Structure and Development | formation | formation of lymphatic system cells | 1.35E-02 | LTB, NFE2 | 2 |
| Lymphoid Tissue Structure and Development | size | size of marginal zone of spleen | 5.54E-03 | TNFSF13B | 1 |
| Lymphoid Tissue Structure and Development | size | size of spleen | 9.03E-03 | HBD, SATB1 | 2 |
| Lymphoid Tissue Structure and Development | expansion | expansion of lymph node cells | 1.10E-02 | PRF1 | 1 |
| Lymphoid Tissue Structure and Development | quantity | quantity of isolated lymphoid follicle | 1.10E-02 | IL7R | 1 |

¬© 2000-2009 Ingenuity Systems, Inc. All rights reserved.

| Category | Function | Function Annotation | P-value | Molecules | # Molecules |
|---|---|---|---|---|---|
| Lymphoid Tissue Structure and Development | quantity | quantity of mesenteric lymph node | 1.10E-02 | IL7R | 1 |
| Tumor Morphology | adhesion | adhesion of myeloid leukemia cells | 2.99E-04 | AMICA1, SELL | 2 |
| Tumor Morphology | cytolysis | cytolysis of tumor cells | 2.27E-03 | FCGR2A, PRF1 | 2 |
| Tumor Morphology | size | size of glioma | 5.54E-03 | PF4 | 1 |
| Tumor Morphology | development | development of thymic lymphoma | 1.10E-02 | PTPRC | 1 |
| Tumor Morphology | growth | growth of murine Lewis lung carcinoma | 1.10E-02 | IL8RB | 1 |
| Small Molecule Biochemistry | release | release of 5-hydroxytryptamine | 6.57E-04 | CD247, FCGR2A, SIGLEC5 | 3 |
| Small Molecule Biochemistry | release | release of Ca2+ | 9.23E-03 | FCGR2A, IL8RB, ITK | 3 |
| Small Molecule Biochemistry | generation | generation of leukotriene C4 | 8.30E-04 | C5AR1, FCGR2A | 2 |
| Small Molecule Biochemistry | generation | generation of platelet activating factor | 5.54E-03 | FCGR2A | 1 |
| Small Molecule Biochemistry | secretion | secretion of nitric oxide | 2.27E-03 | CD8A, PTPRC | 2 |
| Small Molecule Biochemistry | hydroxylation | hydroxylation of leukotriene B4 | 5.54E-03 | CYP4F3 | 1 |
| Small Molecule Biochemistry | immobilization | immobilization of hyaluronic acid | 5.54E-03 | TNFAIP6 | 1 |
| Small Molecule Biochemistry | neutralization | neutralization of heparin | 5.54E-03 | PF4 | 1 |
| Small Molecule Biochemistry | transport | transport of amine | 5.54E-03 | AQP9 | 1 |
| Small Molecule Biochemistry | transport | transport of purine base | 5.54E-03 | AQP9 | 1 |
| Small Molecule Biochemistry | transport | transport of pyrimidine base | 1.10E-02 | AQP9 | 1 |
| Small Molecule Biochemistry | transport | transport of taurine | 1.10E-02 | SLC4A1 | 1 |
| Small Molecule Biochemistry | accumulation | accumulation of phosphatidylinositol 4,5-diphosphate | 1.10E-02 | FCGR2A | 1 |
| Small Molecule Biochemistry | concentration | concentration of bilirubin | 1.10E-02 | HBD | 1 |
| Small Molecule Biochemistry | omega-hydroxylation | omega-hydroxylation of very long chain fatty acid | 1.10E-02 | CYP4F3 | 1 |
| Small Molecule Biochemistry | phospholipid flip-flop | phospholipid flip-flop of phosphatidylserine | 1.12E-02 | CD2, CD48 | 2 |
| Antimicrobial Response | antibacterial response | antibacterial response of organism | 7.46E-04 | CFP, FGR, HCK, LYZ | 4 |
| Antimicrobial Response | clearance | clearance of Theiler's encephalomyelitis virus | 5.54E-03 | PRF1 | 1 |
| Lipid Metabolism | generation | generation of leukotriene C4 | 8.30E-04 | C5AR1, FCGR2A | 2 |
| Lipid Metabolism | generation | generation of platelet activating factor | 5.54E-03 | FCGR2A | 1 |
| Lipid Metabolism | hydroxylation | hydroxylation of leukotriene B4 | 5.54E-03 | CYP4F3 | 1 |
| Lipid Metabolism | accumulation | accumulation of phosphatidylinositol 4,5-diphosphate | 1.10E-02 | FCGR2A | 1 |
| Lipid Metabolism | omega-hydroxylation | omega-hydroxylation of very long chain fatty acid | 1.10E-02 | CYP4F3 | 1 |
| Lipid Metabolism | phospholipid flip-flop | phospholipid flip-flop of phosphatidylserine | 1.12E-02 | CD2, CD48 | 2 |
| Reproductive System Disease | prostate cancer | prostate cancer | 1.15E-03 | CD52, CYTIP, IL7R, ITK, LCK, LTF, LYZ, PTPRC, S100A8 | 9 |
| Neurological Disease | disease | disease of brain cancer cell lines | 1.32E-03 | FPR1, IL8RB | 2 |
| Neurological Disease | necrosis | necrosis of brain | 5.54E-03 | PRF1 | 1 |
| Neurological Disease | apoptosis | apoptosis of oligodendrocyte precursor cells | 1.10E-02 | IL8RB | 1 |
| Renal and Urological System Development and Function | binding | binding of kidney cell lines | 1.72E-03 | FCGR2A, SIGLEC5, SORL1 | 3 |
| Renal and Urological System Development and Function | infiltration | infiltration of kidney | 1.10E-02 | LTB | 1 |
| Reproductive System Development and Function | binding | binding of gonadal cell lines | 1.72E-03 | CD48, LTF, PF4 | 3 |
| Reproductive System Development and Function | angiogenesis | angiogenesis of testis | 1.10E-02 | PROK2 | 1 |
| Reproductive System Development and Function | expansion | expansion of cumulus oocyte complex | 1.10E-02 | TNFAIP6 | 1 |
| Renal and Urological Disease | renal and urological disorder | renal and urological disorder | 1.96E-03 | CD2, CD52, CX3CR1, IL8RB, LGALS3, PTPRC, SLC4A1, TNFSF13B | 8 |
| Renal and Urological Disease | renal and urological disorder | renal and urological disorder of mice | 1.32E-02 | CD2, LGALS3, PTPRC, TNFSF13B | 4 |
| Renal and Urological Disease | damage | damage of kidney | 2.36E-03 | FCGR2A, FGR, HCK | 3 |
| Gene Expression | activation | activation of Nfat binding site | 3.03E-03 | CD247, LCK | 2 |
| Gene Expression | activation | activation of RNA | 1.10E-02 | LGALS3 | 1 |
| Gene Expression | transactivation | transactivation of NF-E2 binding site | 1.10E-02 | NFE2 | 1 |
| Gene Expression | transcription | transcription of matrix-associated region | 1.10E-02 | SATB1 | 1 |
| Gene Expression | binding | binding of EBS/AP1 response element | 1.65E-02 | CD2 | 1 |
| Organ Morphology | morphology | morphology of spleen | 3.03E-03 | LTB, TNFSF13B | 2 |
| Organ Morphology | morphology | morphology of inner nuclear layer | 5.54E-03 | LCK | 1 |
| Organ Morphology | morphology | morphology of photoreceptor layer | 5.54E-03 | LCK | 1 |
| Organ Morphology | destruction | destruction of submandibular gland | 5.54E-03 | TNFSF13B | 1 |
| Organ Morphology | size | size of marginal zone of spleen | 5.54E-03 | TNFSF13B | 1 |

| | | | | ¬© 2000-2009 Ingenuity Systems, Inc. All rights reserved. | |
|---|---|---|---|---|---|
| **Category** | **Function** | **Function Annotation** | **P-value** | **Molecules** | **# Molecules** |
| Organ Morphology | size | size of spleen | 9.03E-03 | HBD, SATB1 | 2 |
| Organ Morphology | expansion | expansion of cumulus oocyte complex | 1.10E-02 | TNFAIP6 | 1 |
| Organ Morphology | quantity | quantity of mesenteric lymph node | 1.10E-02 | IL7R | 1 |
| Organ Morphology | structural integrity | structural integrity of spleen | 1.10E-02 | LTB | 1 |
| Organismal Development | secretion | secretion of mucus | 3.03E-03 | IL8RB, LGALS3 | 2 |
| Organismal Development | production | production of bodily fluid | 9.71E-03 | ITK, TNFSF13B | 2 |
| Dermatological Diseases and Conditions | dermatological disorder | dermatological disorder | 4.03E-03 | CD2, CD52, CYP4F3, FCGR2A, KRT1, LCK, LTB, S100A8, S100A12, SELL | 10 |
| Dermatological Diseases and Conditions | dermatological disorder | dermatological disorder of mice | 1.36E-02 | FCGR2A, LTB, SELL | 3 |
| Dermatological Diseases and Conditions | ichthyosis hystrix, Curth Macklin type | ichthyosis hystrix, Curth Macklin type | 5.54E-03 | KRT1 | 1 |
| Dermatological Diseases and Conditions | lymphadenopathy | lymphadenopathy of skin | 5.54E-03 | SELL | 1 |
| Carbohydrate Metabolism | activation | activation of glycogen | 5.54E-03 | C5AR1 | 1 |
| Carbohydrate Metabolism | generation | generation of platelet activating factor | 5.54E-03 | FCGR2A | 1 |
| Carbohydrate Metabolism | generation | generation of carbohydrate | 1.60E-02 | FCGR2A, PTPRC | 2 |
| Carbohydrate Metabolism | immobilization | immobilization of hyaluronic acid | 5.54E-03 | TNFAIP6 | 1 |
| Carbohydrate Metabolism | neutralization | neutralization of heparin | 5.54E-03 | PF4 | 1 |
| Carbohydrate Metabolism | accumulation | accumulation of phosphatidylinositol 4,5-diphosphate | 1.10E-02 | FCGR2A | 1 |
| Carbohydrate Metabolism | phospholipid flip-flop | phospholipid flip-flop of phosphatidylserine | 1.12E-02 | CD2, CD48 | 2 |
| Cell Cycle | cell cycle progression | entry into cell cycle progression of B lymphocytes | 5.54E-03 | TNFSF13B | 1 |
| Cell Cycle | cell cycle progression | delay in initiation of cell cycle progression of carcinoma cell lines | 1.10E-02 | RASSF5 | 1 |
| Cell Cycle | cell cycle progression | delay in initiation of cell cycle progression of lung cancer cell lines | 1.10E-02 | RASSF5 | 1 |
| Drug Metabolism | immobilization | immobilization of hyaluronic acid | 5.54E-03 | TNFAIP6 | 1 |
| Drug Metabolism | neutralization | neutralization of heparin | 5.54E-03 | PF4 | 1 |
| Gastrointestinal Disease | destruction | destruction of submandibular gland | 5.54E-03 | TNFSF13B | 1 |
| Gastrointestinal Disease | damage | damage of liver | 1.06E-02 | FGR, HCK, PRF1 | 3 |
| Gastrointestinal Disease | damage | damage of crypt | 1.10E-02 | IL7R | 1 |
| Gastrointestinal Disease | damage | damage of intestinal villus | 1.10E-02 | IL7R | 1 |
| Nervous System Development and Function | distribution | distribution of oligodendrocyte precursor cells | 5.54E-03 | IL8RB | 1 |
| Nervous System Development and Function | binding | binding of oligodendrocyte precursor cells | 1.10E-02 | IL8RB | 1 |
| Nervous System Development and Function | migration | arrest in migration of oligodendrocyte precursor cells | 1.10E-02 | IL8RB | 1 |
| Nucleic Acid Metabolism | transport | transport of purine base | 5.54E-03 | AQP9 | 1 |
| Nucleic Acid Metabolism | transport | transport of pyrimidine base | 1.10E-02 | AQP9 | 1 |
| Ophthalmic Disease | herpetic stromal keratitis | herpetic stromal keratitis of eye | 5.54E-03 | IL8RB | 1 |
| RNA Post-Transcriptional Modification | decapping | decapping of RNA | 5.54E-03 | PABPC1 | 1 |
| Visual System Development and Function | invagination | invagination of retina | 5.54E-03 | LCK | 1 |
| Visual System Development and Function | morphology | morphology of inner nuclear layer | 5.54E-03 | LCK | 1 |
| Visual System Development and Function | morphology | morphology of photoreceptor layer | 5.54E-03 | LCK | 1 |
| Organismal Survival | survival | survival of mice | 5.61E-03 | C5AR1, CD2, IL7R, IL8RB, ITK, PRF1, SLC4A1 | 7 |
| Hepatic System Disease | damage | damage of liver | 1.06E-02 | FGR, HCK, PRF1 | 3 |
| Hepatic System Disease | experimentally-induced hepatic fibrosis | experimentally-induced hepatic fibrosis of mice | 1.10E-02 | LGALS3 | 1 |
| Amino Acid Metabolism | transport | transport of taurine | 1.10E-02 | SLC4A1 | 1 |
| Connective Tissue Development and Function | activation | activation of adipocytes | 1.10E-02 | FFAR2 | 1 |
| Hair and Skin Development and Function | cohesiveness | cohesiveness of keratinocytes | 1.10E-02 | CD8A | 1 |
| Metabolic Disease | hereditary renal amyloidoses | hereditary renal amyloidoses | 1.10E-02 | LYZ | 1 |
| Organ Development | angiogenesis | angiogenesis of testis | 1.10E-02 | PROK2 | 1 |
| Organ Development | expansion | expansion of cumulus oocyte complex | 1.10E-02 | TNFAIP6 | 1 |
| Protein Trafficking | co-localization | co-localization of F-actin | 1.10E-02 | FCGR2A | 1 |

## Highly Expressed in MBC versus CVS

**Table S1F**

| | | | | ¬© 2000-2009 Ingenuity Systems, Inc. All rights reserved. | |
|---|---|---|---|---|---|
| **Category** | **Function** | **Function Annotation** | **P-value** | **Molecules** | **# Molecules** |
| Respiratory System Development and Function | binding | binding of lung cell lines | 1.10E-02 | PF4 | 1 |
| DNA Replication, Recombination, and Repair | degradation | degradation of DNA | 1.64E-02 | CD2, GZMH, PRF1, SRGN | 4 |
