# Supplementary material for: Structural and Regulatory Characterization of the Placental Epigenome at Its Maternal Interface
Source: PLoS One. 2011 Feb 23;6(2):e14723. doi: 10.1371/journal.pone.0014723 (PMC3044138; doi:10.1371/journal.pone.0014723)
Supplement: Table S7 — IPA biological network analysis of genes over-expressed in CVS versus MBC. (0.05 MB PDF) [file pone.0014723.s007.pdf]

Highly Expressed in CVS Versus MBC

Table S7

ID	Top Functions	Molecules in Network	Score	Focus Molecules
		¬© 2000-2009 Ingenuity Systems, Inc. All rights reserved.		
1	Cardiovascular System Development and Function, Cancer, Cellular Growth and Proliferation	C12ORF23,CDH5,CSH1,CSH2,DLK1,DPP4,DSP,EBI3,EPS8,ERK,FABP5,GCM1,GH1,GH2,GHR,GNAI1,Growth hormone,H19,Igfbp,IGFBP3,INSL4,Insulin,LDL,LIFR,LIPG,N-cor,NET1,PI3K,PITX2,PLOD2,Rac,Ras homolog,RND3,SNAI2,STAT5a/b	48	25
2	Organ Morphology, Reproductive System Development and Function, Cancer	ADAMTS1,CGA,COL15A1,Creb,CYP11A1,DSG2,DUSP9,EFEMP1,FBN1,FSH,GNGT1,hCG,HSPE1,Integrin alpha V beta 3,ITGAV,KISS1,LAMB1,LAMC1,Laminin,Laminin2,Mek,MFAP5,NFkB (complex),NID2,OLR1,PLK2,PPAP2B,Scf,SDC1,Secretase gamma,SERPINB2,SERPINF1,SKP2,TFPI2,Vegf	44	24
3	Cardiovascular System Development and Function, Cellular Movement, Cell-To-Cell Signaling and Interaction	Akt,ALP,ANGPT2,Ap1,CDO1,COL3A1,COL4A2,Collagen(s),CSRP2,DCN,F3,FAK,FERMT2,Fibrinogen,HAPLN1,HGF,HSPA2,IFN Beta,IKK (complex),IL1,Integrin,KDR,Mmp,p70 S6k,Pdgf,PDGF BB,PLA2G2A,RASA1,Shc,SNRPG,SPARC,SPP1,Tgf beta,VCAM1,YAP1	33	19
4	Cellular Assembly and Organization, Hair and Skin Development and Function, Hepatic System Development and Function	ASS1,Calcineurin protein(s),Caspase,CDH1,CDK7,COL4A1,Cyclin A,DKK1,GJA1,GMNN,Histone h3,Hsp70,HSPB1,IFI6,IgG,IL12 (complex),Interferon alpha,Jnk,KRT8,KRT18,MAP2K1/2,MAP4K3,NFE2L3,P38 MAPK,PKD2 (includes EG:5311),PLS3,Proteasome,PVRL3,Ras,Rb,RNA polymerase II,RNF128,Sapk,SLC27A2,TLR3	33	19
5	Amino Acid Metabolism, Post-Translational Modification, Small Molecule Biochemistry	AGL,AGTR1,ATP8A2,BAS,C8ORF4,CETN3,CLIC3,COL6A3,CRY1,ERK1/2,F Actin,FAM158A,GABRE,GCSH,GLDC,HNF4A,IGH-IA,IL15,KCTD3,LRP11,Mapk,MAPK15,NUDT6,OGN,Pka,Pkc(s),PPARG,PPIC,PPIL1,PSG1,RSPH3,SGCE,SMAD3,TTC35,ZNHIT6	26	16
6	Endocrine System Development and Function, Small Molecule Biochemistry, Drug Metabolism	ABCB10,ABCG2,BAZ1A,beta-estradiol,COLEC12,EGLN1,FAM162A,GCHFR,GMFB,GPX3,GSR,GSTT1,HIF1A,HSD11B2,HSD17B1,HSD17B2,HSD17B3,HSD17B7,KLK6,MAFF,MAOA,MATN2,MGP,OS9,PKIB,PNRC1,PSG9,RCN2,RHOBTB1,SLC16A4,SMAD2,SMS,SRM,TRPV5,VitaminD3-VDR-RXR	20	14
7	Cell Signaling, Connective Tissue Development and Function, Skeletal and Muscular System Development and Function	BMP1,BMP4,CCKAR,CDC42SE1,CHRD,CKS2,COL5A1,COL5A2,CRK/CRKL,ENPP1,ENPP2,ERBB2,FBXW5,GSTA2,HTRA1,IL1B,IL1F6,LUM,MAPK8,NRK,PELI3,PHYH,PPM1L,PROCR,PTPN18,RASA2,SH2D3A,SHC2,SPINT1,SRPX,STMN3,TWSG1,UAP1,WBP5,ZNF675	20	13
8	Cancer, Tumor Morphology, Cell Cycle	ANLN,ARL6IP1,c-Myc/N-Myc,CRIM1,EFEMP2,EGFL6,FAM115A,FBXW8,FRMD6,GART,GGH,KIAA0101,KIAA1524,LRDD,MYC,MYCN,NFYB,PEG10,PERP,PGM3,PLSCR4,PMP22,ROBO1,RPL41,RPS12 (includes EG:6206),RRM2B,SESN1,SGTB,SLC19A2,SLC25A19,SLIT3,TP53,UBE2S,YY2 (includes EG:404281),ZNF468	19	13
9	Carbohydrate Metabolism, Small Molecule Biochemistry, Cell Cycle	Actin,AIM2,ANXA8L2,BPI,CALML3,CDC42,CDC42SE1,CHST3,FETUB,FNBP1L,GPR21,GRHL1,HOXD1,HS3ST1,HS6ST1,HS6ST2,LYVE1,MIR124,MORC4,NDST1,NOS2,PCOLCE2 (includes EG:26577),PSG3,RACGAP1,RAI14,RELA,retinoic acid,ROR1,SKIL,STARD10,SVEP1,TCHH,TGFB1,Vacuolar H+ ATPase,VGLL3	16	11
10	Protein Degradation, Amino Acid Metabolism, Small Molecule Biochemistry	ADAMTS14,ADCYAP1,ASTL,BTBD3,Ca2+,CASP8,CBL,Ck2,CNN3,CPA5,CSHL1,CTRL,ENPEP,IMMP2L,LGMN,MEST,MIR199A1,MMP1B,NAALADL1,NFKBIA,NOTCH1,PEPC (includes EG:109616),peptidase,PIR,PRT5,PRT6,PSG2,RCN1,RNF150,SENP17,SENP5 (includes EG:303874),STS,TMPRSS11D,TWIST1,UPG2	16	11
11	Cell Cycle, Cell Death, Cell-mediated Immune Response	ARMCX1,STAT4	2	1
12	Lipid Metabolism, Small Molecule Biochemistry	LGALS13,Lysophospholipase	2	1
13		RDH,RDH14	2	1
14	Cardiac Arteriopathy, Cardiovascular Disease, Genetic Disorder	1-acylglycerol-3-phosphate O-acyltransferase,AGPAT5,Phospholipid:diacylglycerol acyltransferase	2	1
15	Embryonic Development, Organismal Development, Tissue Morphology	C4ORF14,HIRA,NFU1,NOVA1	2	1
