# Supplementary material for: Structural and Regulatory Characterization of the Placental Epigenome at Its Maternal Interface
Source: PLoS One. 2011 Feb 23;6(2):e14723. doi: 10.1371/journal.pone.0014723 (PMC3044138; doi:10.1371/journal.pone.0014723)
Supplement: Table S8 — IPA biological pathway analysis of genes over-expressed in CVS versus MBC. (0.33 MB PDF) [file pone.0014723.s008.pdf]

Highly Expressed in CVS Versus MBC

Table S8

				¬© 2000-2009 Ingenuity Systems, Inc. All rights reserved.	
**Category**	**Function**	**Function Annotation**	**P-Value**	**Molecules**	**# Molecules**
Cancer	neoplasia	neoplasia	4.40E-17	ABCG2, ADAMTS1, AGTR1, ANGPT2, ASS1, BEX4, CDH1, CDK7, CETN3, CKS2, COL15A1, COL3A1, COL4A1, COL4A2, COL6A3, COLEC12, CSRP2, DCN, DKK1, DLK1, DPP4, DSG2, EBI3, EFEMP1, ENPEP, ENPP2, EPS8, F3, FABP5, FBN1, GABRE, GGH, GH1, GHR, GJA1, GMNN, GPX3, H19, HAPLN1, HGF, HSD11B2, HSD17B2, HSPE1, HTRA1, IGFBP3, ITGAV, KDR, KISS1, KRT18, LIFR, MAOA, MEST, MFAP5, OLR1, PHF16, PITX2, PKD2 (includes EG:5311), PLA2G2A, PLOD2, PLSCR4, PPAP2B, PVRL3, RACGAP1, ROBO1, SDC1, SERPINB2, SERPINF1, SKP2, SLC16A4, SLC19A2, SLC27A2, SMS, SNAI2, SPARC, SPP1, SRPX, STS, TCEAL4, TFPI2, TLR3, TWIST1, VCAM1	82
Cancer	neoplasia	neoplasia of skin	3.70E-03	SERPINB2, SPP1	2
Cancer	neoplasia	neoplasia of organ	1.00E-02	HGF, SERPINB2, SPP1	3
Cancer	tumorigenesis	tumorigenesis	6.55E-17	ABCG2, ADAMTS1, AGTR1, ANGPT2, ASS1, BEX4, CDH1, CDK7, CETN3, CGA, CKS2, COL15A1, COL3A1, COL4A1, COL4A2, COL6A3, COLEC12, CSRP2, CYP11A1, DCN, DKK1, DLK1, DPP4, DSG2, EBI3, EFEMP1, ENPEP, ENPP2, EPS8, F3, FABP5, FBN1, GABRE, GGH, GH1, GHR, GJA1, GMNN, GPX3, H19, HAPLN1, HGF, HSD11B2, HSD17B2, HSPE1, HTRA1, IGFBP3, ITGAV, KDR, KISS1, KRT18, LIFR, MAOA, MEST, MFAP5, OLR1, PHF16, PITX2, PKD2 (includes EG:5311), PLA2G2A, PLOD2, PLSCR4, PPAP2B, PVRL3, RACGAP1, RASA1, ROBO1, SDC1, SERPINB2, SERPINF1, SKP2, SLC16A4, SLC19A2, SLC27A2, SMS, SNAI2, SPARC, SPP1, SRPX, STS, TCEAL4, TFPI2, TLR3, TWIST1, VCAM1	85
Cancer	tumorigenesis	tumorigenesis of cells	4.34E-04	AGTR1, CGA, DPP4, ENPP2, F3, FABP5, GHR, HGF, ITGAV, KISS1, TWIST1	11
Cancer	tumorigenesis	tumorigenesis of cell lines	1.79E-03	DPP4, ENPP2, F3, FABP5, HGF, KISS1, TWIST1	7
Cancer	tumorigenesis	tumorigenesis of tumor cell lines	1.83E-03	DPP4, F3, FABP5, HGF, KISS1, TWIST1	6
Cancer	tumorigenesis	tumorigenesis of melanoma cell lines	1.83E-03	DPP4, F3, KISS1	3
Cancer	tumorigenesis	tumorigenesis of eukaryotic cells	3.73E-03	AGTR1, CGA, DPP4, ENPP2, F3, FABP5, HGF, KISS1, TWIST1	9
Cancer	cancer	cancer	7.74E-16	ABCG2, ADAMTS1, AGTR1, ANGPT2, ASS1, BEX4, CDH1, CDK7, CETN3, CKS2, COL15A1, COL3A1, COL4A1, COL4A2, COL6A3, COLEC12, CSRP2, DCN, DKK1, DLK1, DPP4, DSG2, EBI3, EFEMP1, ENPEP, ENPP2, EPS8, F3, FBN1, GABRE, GGH, GH1, GHR, GJA1, GMNN, GPX3, H19, HAPLN1, HGF, HSD11B2, HSD17B2, HSPE1, HTRA1, IGFBP3, ITGAV, KDR, KRT18, LIFR, MAOA, MEST, MFAP5, OLR1, PHF16, PITX2, PLA2G2A, PLOD2, PLSCR4, PPAP2B, PVRL3, RACGAP1, ROBO1, SDC1, SERPINF1, SKP2, SLC16A4, SLC19A2, SLC27A2, SMS, SNAI2, SPARC, SPP1, SRPX, STS, TCEAL4, TFPI2, TLR3, TWIST1, VCAM1	78
Cancer	colorectal cancer	colorectal cancer	1.73E-14	ADAMTS1, BEX4, CDK7, COL3A1, COL4A1, COL4A2, COL6A3, COLEC12, DKK1, DSG2, EBI3, EFEMP1, ENPP2, EPS8, GGH, GJA1, GMNN, HSD11B2, HSD17B2, HSPE1, HTRA1, KDR, KRT18, MEST, PHF16, PITX2, PLA2G2A, PLOD2, PLSCR4, PPAP2B, PVRL3, SDC1, SLC27A2, SPP1, SRPX, TWIST1, VCAM1	37
Cancer	tumor	tumor	8.83E-10	ABCG2, ADAMTS1, AGTR1, ANGPT2, ASS1, CDH1, CDK7, CETN3, CGA, CKS2, COL15A1, COL3A1, COL4A1, COL4A2, COL6A3, CSRP2, DCN, DLK1, DPP4, EBI3, ENPEP, F3, FBN1, GABRE, GJA1, GMNN, GPX3, H19, HGF, HSPE1, IGFBP3, ITGAV, KDR, KRT18, LIFR, MAOA, MEST, MFAP5, OLR1, PKD2 (includes EG:5311), PLA2G2A, RACGAP1, SDC1, SERPINB2, SERPINF1, SKP2, SLC16A4, SLC19A2, SMS, SNAI2, SPARC, SPP1, STS, TCEAL4, TFPI2, TLR3, TWIST1	57
Cancer	migration	migration of tumor cells	2.95E-09	DPP4, ENPP2, F3, GH1, HGF, ITGAV, KDR, SERPINB2, SPARC, VCAM1	10
Cancer	migration	migration of tumor cell lines	2.03E-06	CDH1, CDK7, DPP4, ENPP2, GJA1, HGF, IGFBP3, ITGAV, KISS1, PITX2, PPIC, SDC1, SLC16A4, SPP1, TWIST1, VCAM1	16
Cancer	migration	migration of breast cancer cell lines	2.48E-06	GJA1, HGF, IGFBP3, ITGAV, KISS1, PPIC, SDC1, SLC16A4, SPP1	9
Cancer	migration	migration of cancer cells	1.56E-05	DPP4, HGF, ITGAV, KDR, SPARC, VCAM1	6
Cancer	migration	migration of prostate cancer cells	1.58E-05	DPP4, ITGAV, SPARC	3
Cancer	migration	migration of ovarian cancer cell lines	8.06E-04	CDK7, DPP4, HGF	3
Cancer	migration	migration of fibrosarcoma cell lines	2.40E-03	ITGAV, KISS1, SDC1	3
Cancer	migration	migration of thyroid tumor cell lines	3.70E-03	HGF, KISS1	2
Cancer	migration	migration of glioma cells	5.85E-03	HGF, ITGAV	2
Cancer	migration	migration of mammary tumor cells	5.85E-03	GH1, ITGAV	2
Cancer	migration	migration of squamous cell carcinoma cell lines	9.90E-03	HGF, ITGAV	2
Cancer	primary tumor	primary tumor	5.56E-09	ABCG2, ADAMTS1, AGTR1, ANGPT2, ASS1, CDH1, CDK7, CETN3, CKS2, COL15A1, COL3A1, COL4A1, COL4A2, COL6A3, CSRP2, DCN, DLK1, DPP4, EBI3, ENPEP, F3, FBN1, GABRE, GJA1, GMNN, GPX3, H19, HGF, HSPE1, IGFBP3, ITGAV, KDR, KRT18, LIFR, MAOA, MEST, MFAP5, PLA2G2A, RACGAP1, SERPINB2, SERPINF1, SKP2, SLC16A4, SLC19A2, SMS, SNAI2, SPARC, SPP1, STS, TCEAL4, TFPI2, TLR3, TWIST1	53
Cancer	malignant tumor	malignant tumor	1.63E-08	ABCG2, ADAMTS1, AGTR1, ANGPT2, ASS1, CDH1, CDK7, CETN3, CKS2, COL15A1, COL3A1, COL4A1, COL4A2, COL6A3, CSRP2, DCN, DLK1, DPP4, EBI3, ENPEP, F3, FBN1, GABRE, GJA1, GMNN, GPX3, H19, HGF, HSPE1, IGFBP3, ITGAV, KDR, KRT18, LIFR, MAOA, MEST, MFAP5, RACGAP1, SERPINF1, SKP2, SLC16A4, SLC19A2, SMS, SNAI2, SPARC, SPP1, STS, TCEAL4, TFPI2, TLR3, TWIST1	51
Cancer	invasion	invasion of melanoma cell lines	1.73E-06	ENPP2, HGF, ITGAV, RND3, SPARC, SPP1	6
Cancer	invasion	invasion of tumor cell lines	1.83E-05	CDH1, DPP4, ENPP2, FABP5, HGF, ITGAV, KISS1, PPIC, RND3, SKP2, SPARC, SPP1, TFPI2, TWIST1	14
Cancer	invasion	invasion of cells	7.39E-05	CDH1, DPP4, ENPP2, FABP5, GH1, HGF, ITGAV, KISS1, LGMN, PPIC, RND3, SKP2, SPARC, SPP1, TFPI2, TWIST1	16

				¬© 2000-2009 Ingenuity Systems, Inc. All rights reserved.	
**Category**	**Function**	**Function Annotation**	**P-Value**	**Molecules**	**# Molecules**
Cancer	invasion	invasion of tumor cells	9.58E-05	CDH1, DPP4, ENPP2, GH1, HGF, ITGAV	6
Cancer	invasion	invasion of eukaryotic cells	1.57E-04	CDH1, DPP4, ENPP2, FABP5, GH1, HGF, ITGAV, KISS1, PPIC, RND3, SKP2, SPARC, SPP1, TFPI2, TWIST1	15
Cancer	invasion	invasion of hepatoma cell lines	8.06E-04	HGF, SPP1, TFPI2	3
Cancer	invasion	invasion of cancer cells	1.11E-03	CDH1, DPP4, HGF, ITGAV	4
Cancer	invasion	invasion of breast cancer cell lines	1.66E-03	CDH1, HGF, PPIC, SPARC, SPP1, TWIST1	6
Cancer	invasion	invasion of squamous cell carcinoma cell lines	4.70E-03	HGF, ITGAV, SPP1	3
Cancer	invasion	invasion of melanoma cells	5.85E-03	CDH1, ITGAV	2
Cancer	invasion	invasion of prostate cancer cell lines	1.00E-02	CDH1, FABP5, SPP1	3
Cancer	carcinoma	carcinoma	2.02E-06	ADAMTS1, AGTR1, ANGPT2, ASS1, CDH1, CDK7, CKS2, COL15A1, COL6A3, CSRP2, DLK1, DPP4, EBI3, ENPEP, F3, GJA1, GMNN, GPX3, HGF, HSPE1, IGFBP3, ITGAV, KDR, KRT18, MFAP5, RACGAP1, SERPINF1, SKP2, SLC16A4, SLC19A2, SMS, SPP1, STS, TCEAL4, TFPI2, TLR3, TWIST1	37
Cancer	proliferation	proliferation of tumor cell lines	5.39E-06	CDH1, CKS2, CSH1, CSH2, DCN, DLK1, EPS8, FABP5, GH1, GH2, GHR, GJA1, HGF, HSD11B2, IGFBP3, ITGAV, KDR, KISS1, SERPINB2, SKP2, SPARC, SPP1, TFPI2	23
Cancer	proliferation	proliferation of lymphoma cell lines	6.95E-04	CSH1, CSH2, GH1, GH2, IGFBP3	5
Cancer	proliferation	proliferation of breast cancer cell lines	8.72E-03	CDH1, DCN, GH1, IGFBP3, KISS1, SKP2	6
Cancer	metastasis	metastasis of cells	8.88E-06	ENPP2, F3, FABP5, HGF, ITGAV, KISS1, TWIST1	7
Cancer	metastasis	metastasis of cell lines	9.07E-06	ENPP2, F3, FABP5, HGF, KISS1, TWIST1	6
Cancer	metastasis	metastasis of tumor cell lines	6.90E-05	F3, FABP5, HGF, KISS1, TWIST1	5
Cancer	metastasis	metastasis of breast cancer cell lines	3.28E-04	FABP5, HGF, TWIST1	3
Cancer	metastasis	metastasis	9.19E-04	ENPP2, F3, FABP5, HGF, ITGAV, KDR, KISS1, TWIST1	8
Cancer	metastasis	metastasis of melanoma cell lines	8.44E-03	F3, KISS1	2
Cancer	binding	binding of tumor cell lines	2.20E-05	DCN, DPP4, F3, FERMT2, HGF, IGFBP3, ITGAV, SERPINF1, SPP1, VCAM1	10
Cancer	binding	binding of tumor cells	2.10E-03	DKK1, ITGAV, VCAM1	3
Cancer	adhesion	adhesion of tumor cell lines	3.92E-05	CDH1, DCN, DSG2, HGF, ITGAV, PITX2, PVRL3, SDC1, SERPINB2, SPP1, VCAM1	11
Cancer	adhesion	adhesion of leukemia cell lines	3.73E-03	CDH1, SERPINB2, SPP1, VCAM1	4
Cancer	adhesion	adhesion of colon cancer cell lines	5.18E-03	CDH1, DSG2, HGF	3
Cancer	adhesion	adhesion of lymphoma cell lines	8.00E-03	HGF, SDC1, VCAM1	3
Cancer	adhesion	adhesion of tumor cells	8.00E-03	F3, ITGAV, VCAM1	3
Cancer	adhesion	adhesion of melanoma cell lines	8.44E-03	ITGAV, SPP1	2
Cancer	aggregation	aggregation of squamous cell carcinoma cell lines	1.38E-04	CDH1, ITGAV	2
Cancer	aggregation	aggregation of colon cancer cell lines	2.01E-03	CDH1, PSG2	2
Cancer	aggregation	aggregation of tumor cell lines	1.08E-02	CDH1, ITGAV, PSG2	3
Cancer	branching	branching of tumor cell lines	1.38E-04	CDH1, HGF	2
Cancer	cell-cell contact	cell-cell contact of tumor cells	1.38E-04	CDH1, GH1	2
Cancer	morphogenesis	morphogenesis of breast cancer cell lines	1.38E-04	CDH1, HGF	2
Cancer	colon cancer	colon cancer	1.69E-04	ADAMTS1, CDK7, COL3A1, EBI3, GGH, GJA1, GMNN, HSD11B2, HSD17B2, HSPE1, PLA2G2A, PVRL3, SLC27A2, SPP1	14
Cancer	growth	growth of cancer cells	2.87E-04	CDH5, DCN, HGF, ITGAV, KDR, PEG10, SKP2	7
Cancer	growth	growth of breast cancer cell lines	8.51E-04	CDH1, COL6A3, DCN, GJA1, HGF, IGFBP3, SKP2, SPP1	8
Cancer	growth	growth of tumor	3.31E-03	COL4A1, GJA1, HGF, ITGAV, KDR, OLR1, SPP1	7
Cancer	developmental process	developmental process of tumor	3.72E-04	CDH5, COL4A1, GJA1, HGF, ITGAV, KDR, OLR1, SDC1, SKP2, SNAI2, SPP1	11
Cancer	developmental process	developmental process of thyroid tumor cell lines	9.90E-03	HGF, KISS1	2
Cancer	serous ovarian carcinoma	serous ovarian carcinoma	5.18E-04	COL15A1, DPP4, ENPEP, GPX3, MFAP5, RACGAP1, TFPI2	7
Cancer	ovarian cancer	ovarian cancer	7.28E-04	CDH1, COL15A1, COL4A1, DPP4, ENPEP, GPX3, H19, KDR, MFAP5, RACGAP1, SPP1, TFPI2	12
Cancer	development	development of papilloma	8.17E-04	HGF, SPP1	2
Cancer	development	development of squamous-cell carcinoma	2.01E-03	HGF, SKP2	2
Cancer	development	development of tumor	2.79E-03	CDH5, COL4A1, HGF, SDC1, SKP2, SNAI2, SPP1	7
Cancer	development	development of tumor cell lines	3.03E-03	AGTR1, CDH1, GMNN, HGF	4
Cancer	multiple myeloma	multiple myeloma	1.00E-03	CDK7, DKK1, GH1, GHR, ITGAV, KDR, SDC1	7
Cancer	cell death	cell death of breast cancer cell lines	1.07E-03	ABCG2, CDH1, GH1, HGF, IGFBP3, ITGAV, KRT18, PLK2, RASA1, SDC1	10
Cancer	cell death	cell death of tumor cell lines	5.00E-03	ABCG2, ANGPT2, CDH1, DKK1, DPP4, DSG2, DUSP9, GH1, HGF, HSPB1, IFI6, IGFBP3, ITGAV, KRT18, MAOA, PLK2, PSG2, RASA1, SDC1, SKP2, SPARC, SPP1, SRPX, TWIST1	24
Cancer	cell death	cell death of tumor cells	5.24E-03	CDH1, DPP4, DUSP9, GH1, ITGAV, KDR, SNAI2, SPARC	8
Cancer	interphase	interphase of tumor cell lines	1.10E-03	CDH1, DCN, DPP4, DUSP9, GJA1, GMNN, HGF, ITGAV, PITX2, SKP2	10
Cancer	interphase	arrest in interphase of tumor cell lines	5.02E-03	DCN, DPP4, DUSP9, HGF, ITGAV, PITX2, SKP2	7
Cancer	cell division process	cell division process of tumor cell lines	1.20E-03	CDH1, CKS2, DCN, DPP4, DUSP9, GH1, GJA1, GMNN, GNAI1, HGF, IGFBP3, ITGAV, PITX2, SKP2	14

				¬© 2000-2009 Ingenuity Systems, Inc. All rights reserved.	
**Category**	**Function**	**Function Annotation**	**P-Value**	**Molecules**	**# Molecules**
Cancer	cell division process	cell division process of carcinoma cells	2.01E-03	DCN, GH1	2
Cancer	cell division process	arrest in cell division process of tumor cell lines	7.46E-03	CKS2, DCN, DPP4, DUSP9, HGF, ITGAV, PITX2, SKP2	8
Cancer	cell division process	cell division process of cancer cells	9.33E-03	DCN, DPP4, GH1	3
Cancer	hyperproliferation	hyperproliferation	1.25E-03	AGTR1, CDH1, CGA, CYP11A1, GH1, GHR, HSD11B2, RASA1, SDC1, SKP2, SPP1	11
Cancer	G1 phase	G1 phase of ovarian cancer cell lines	1.35E-03	ITGAV, SKP2	2
Cancer	chemotaxis	chemotaxis of rhabdomyosarcoma cell lines	1.35E-03	HGF, LIFR	2
Cancer	synovial sarcoma	synovial sarcoma	1.35E-03	CDH1, KDR	2
Cancer	apoptosis	apoptosis of breast cancer cell lines	1.54E-03	CDH1, GH1, HGF, IGFBP3, ITGAV, KRT18, PLK2, RASA1, SDC1	9
Cancer	apoptosis	apoptosis of tumor cell lines	3.21E-03	ABCG2, ANGPT2, CDH1, DKK1, DSG2, GH1, HGF, HSPB1, IFI6, IGFBP3, ITGAV, KRT18, MAOA, PLK2, PSG2, RASA1, SDC1, SKP2, SPARC, SPP1, SRPX, TWIST1	22
Cancer	apoptosis	apoptosis of colon cancer cell lines	3.78E-03	DSG2, IGFBP3, ITGAV, KRT18, PSG2, SPARC, SPP1	7
Cancer	apoptosis	apoptosis of cancer cells	5.88E-03	CDH1, DPP4, GH1, ITGAV, KDR, SPARC	6
Cancer	apoptosis	apoptosis of tumor cells	8.63E-03	CDH1, DPP4, GH1, ITGAV, KDR, SNAI2, SPARC	7
Cancer	cell movement	cell movement of colon cancer cell lines	1.83E-03	CDH1, EPS8, FERMT2	3
Cancer	cell movement	cell movement of glioma cells	2.79E-03	ITGAV, SPP1	2
Cancer	cell movement	cell movement of tumor cell lines	6.77E-03	CDH1, EPS8, FERMT2, HGF, ITGAV, LIFR, SPARC, SPP1	8
Cancer	cell movement	cell movement of tumor cells	8.65E-03	GH1, ITGAV, SPP1	3
Cancer	follicular thyroid carcinoma	follicular thyroid carcinoma	1.83E-03	ADAMTS1, ANGPT2, SPP1	3
Cancer	damage	damage of tumor cell lines	2.01E-03	GJA1, SPP1	2
Cancer	survival	survival of squamous cell carcinoma cell lines	2.01E-03	CDH1, HGF	2
Cancer	survival	survival of cancer cells	3.06E-03	ITGAV, SPARC, TWIST1	3
Cancer	cell stage	cell stage of tumor cell lines	2.24E-03	CDH1, DCN, DPP4, DUSP9, GJA1, GMNN, GNAI1, HGF, ITGAV, PITX2, SKP2	11
Cancer	genital tumor	genital tumor	2.36E-03	AGTR1, ASS1, CDK7, COL15A1, DPP4, ENPEP, GPX3, IGFBP3, ITGAV, KDR, MFAP5, RACGAP1, SMS, TFPI2	14
Cancer	anoikis	anoikis of colon cancer cell lines	2.79E-03	ITGAV, PSG2	2
Cancer	anoikis	anoikis of tumor cell lines	6.78E-03	HGF, ITGAV, PSG2	3
Cancer	mitogenesis	mitogenesis of bone cancer cell lines	2.79E-03	GH1, IGFBP3	2
Cancer	thyroid cancer	thyroid cancer	2.97E-03	ADAMTS1, ANGPT2, HGF, KDR, SPP1	5
Cancer	prostatic intraepithelial tumor	prostatic intraepithelial tumor	3.65E-03	AGTR1, ASS1, GPX3, IGFBP3, SMS	5
Cancer	peripheral T-cell lymphoma	peripheral T-cell lymphoma	3.83E-03	COL3A1, COL4A1, COL4A2	3
Cancer	T-cell non-hodgkin lymphoma	T-cell non-hodgkin lymphoma	4.63E-03	COL3A1, COL4A1, COL4A2, DCN, SKP2	5
Cancer	sarcoma	sarcoma	4.97E-03	CDH1, CDK7, CKS2, HGF, ITGAV, KDR, SPARC, SPP1	8
Cancer	acute lymphoblastic leukemia	acute lymphoblastic leukemia	5.13E-03	DPP4, F3, GH1, GHR	4
Cancer	digestive organ tumor	digestive organ tumor	5.17E-03	CDH1, CDK7, COL6A3, EBI3, GJA1, GMNN, HGF, HSPE1, IGFBP3, ITGAV, KDR, KRT18, SLC16A4, SPP1	14
Cancer	head and neck cancer	head and neck cancer	5.43E-03	ABCG2, ADAMTS1, ANGPT2, CDK7, COL6A3, ITGAV, KDR, KRT18, SPP1, TLR3, TWIST1	11
Cancer	ovarian tumor	ovarian tumor	5.82E-03	COL15A1, DPP4, ENPEP, GPX3, KDR, MFAP5, RACGAP1, TFPI2	8
Cancer	lymphoma	lymphoma	6.01E-03	CDK7, COL3A1, COL4A1, COL4A2, DCN, DPP4, ITGAV, KDR, SKP2	9
Cancer	cardiovascular process	cardiovascular process of tumor	6.12E-03	CDH5, COL4A1, HGF, KDR	4
Cancer	head and neck tumor	head and neck tumor	6.43E-03	ABCG2, ADAMTS1, ANGPT2, CDK7, COL6A3, ITGAV, KDR, KRT18, SPP1, TWIST1	10
Cancer	uterine tumor	uterine tumor	6.96E-03	CDH1, CDK7, CETN3, COL6A3, FBN1, H19, KDR, LIFR, MAOA, MEST	10
Cancer	non-hodgkin lymphoma	non-hodgkin lymphoma	7.96E-03	CDK7, COL3A1, COL4A1, COL4A2, DCN, DPP4, SKP2	7
Cancer	leiomyosarcoma	leiomyosarcoma	8.00E-03	CKS2, KDR, SPP1	3
Cancer	cell-cell adhesion	cell-cell adhesion of tumor cell lines	8.44E-03	CDH1, HGF	2
Cancer	myosarcoma	myosarcoma	8.46E-03	CKS2, HGF, KDR, SPP1	4
Cancer	hematologic cancer	hematologic cancer	9.83E-03	CDK7, DKK1, DPP4, F3, GH1, GHR, ITGAV, KDR, SDC1, SNAI2	10
Cancer	prostate cancer	prostate cancer	1.02E-02	AGTR1, ASS1, CDH1, CDK7, FBN1, GPX3, HAPLN1, IGFBP3, ITGAV, KDR, SMS, SPP1	12
Cancer	thyroid gland tumor	thyroid gland tumor	1.03E-02	ADAMTS1, ANGPT2, KDR, SPP1	4
Cancer	morphology	morphology of prostate cancer cell lines	1.15E-02	CDH1, TWIST1	2
Cancer	bone tumor	bone tumor	1.17E-02	CDK7, ITGAV, KDR, SPARC, SPP1	5
Cancer	accumulation	accumulation of prostate cancer cell lines	1.18E-02	HGF	1
Gastrointestinal Disease	colorectal cancer	colorectal cancer	1.73E-14	ADAMTS1, BEX4, CDK7, COL3A1, COL4A1, COL4A2, COL6A3, COLEC12, DKK1, DSG2, EBI3, EFEMP1, ENPP2, EPS8, GGH, GJA1, GMNN, HSD11B2, HSD17B2, HSPE1, HTRA1, KDR, KRT18, MEST, PHF16, PITX2, PLA2G2A, PLOD2, PLSCR4, PPAP2B, PVRL3, SDC1, SLC27A2, SPP1, SRPX, TWIST1, VCAM1	37
Gastrointestinal Disease	colon cancer	colon cancer	1.69E-04	ADAMTS1, CDK7, COL3A1, EBI3, GGH, GJA1, GMNN, HSD11B2, HSD17B2, HSPE1, PLA2G2A, PVRL3, SLC27A2, SPP1	14
Gastrointestinal Disease	short bowel syndrome	short bowel syndrome	4.12E-04	GH1, GHR	2
Gastrointestinal Disease	cell movement	cell movement of colon cancer cell lines	1.83E-03	CDH1, EPS8, FERMT2	3

				¬© 2000-2009 Ingenuity Systems, Inc. All rights reserved.	
**Category**	**Function**	**Function Annotation**	**P-Value**	**Molecules**	**# Molecules**
Gastrointestinal Disease	aggregation	aggregation of colon cancer cell lines	2.01E-03	CDH1, PSG2	2
Gastrointestinal Disease	anoikis	anoikis of colon cancer cell lines	2.79E-03	ITGAV, PSG2	2
Gastrointestinal Disease	apoptosis	apoptosis of colon cancer cell lines	3.78E-03	DSG2, IGFBP3, ITGAV, KRT18, PSG2, SPARC, SPP1	7
Gastrointestinal Disease	digestive organ tumor	digestive organ tumor	5.17E-03	CDH1, CDK7, COL6A3, EBI3, GJA1, GMNN, HGF, HSPE1, IGFBP3, ITGAV, KDR, KRT18, SLC16A4, SPP1	14
Gastrointestinal Disease	adhesion	adhesion of colon cancer cell lines	5.18E-03	CDH1, DSG2, HGF	3
Cardiovascular System Development and Function	development	development of blood vessel	1.01E-10	ADAMTS1, AGTR1, ANGPT2, CDH5, COL3A1, COL4A1, COL4A2, COL5A1, CSH1, ENPEP, F3, GH1, GH2, GJA1, HGF, ITGAV, KDR, LAMC1, PITX2, PPAP2B, RASA1, SERPINF1, SPARC, VCAM1, YAP1	25
Cardiovascular System Development and Function	development	development of capillary vessel	6.30E-05	CSH1, GH1, GH2, KDR	4
Cardiovascular System Development and Function	development	development of heart	4.71E-03	COL3A1, COL5A1, DSP, FBN1, GJA1, PITX2, PKD2 (includes EG:5311), VCAM1	8
Cardiovascular System Development and Function	angiogenesis	angiogenesis	2.61E-07	ADAMTS1, AGTR1, ANGPT2, CDH5, COL4A1, COL4A2, CSH1, ENPEP, GH1, GH2, HGF, ITGAV, KDR, LAMC1, PITX2, SERPINF1, SPARC, VCAM1	18
Cardiovascular System Development and Function	angiogenesis	angiogenesis of organism	7.40E-05	ANGPT2, CDH5, HGF, ITGAV, KDR, LAMC1, SERPINF1	7
Cardiovascular System Development and Function	angiogenesis	angiogenesis of capillary vessel	8.64E-05	CSH1, GH1, GH2	3
Cardiovascular System Development and Function	angiogenesis	angiogenesis of mammalia	1.24E-04	ANGPT2, CDH5, HGF, ITGAV, KDR, SERPINF1	6
Cardiovascular System Development and Function	angiogenesis	angiogenesis of tissue	6.43E-04	ADAMTS1, ANGPT2, HGF, SERPINF1, SPARC	5
Cardiovascular System Development and Function	angiogenesis	angiogenesis of mice	8.08E-04	ANGPT2, CDH5, HGF, ITGAV, KDR	5
Cardiovascular System Development and Function	angiogenesis	angiogenesis of cornea	4.70E-03	CDH5, HGF, VCAM1	3
Cardiovascular System Development and Function	angiogenesis	angiogenesis of skin	7.09E-03	HGF, KDR	2
Cardiovascular System Development and Function	formation	formation of blood vessel	3.52E-06	ADAMTS1, CDH5, COL4A2, ENPP2, HGF, KDR, OLR1	7
Cardiovascular System Development and Function	formation	formation of capillary vessel	3.83E-03	ADAMTS1, KDR, OLR1	3
Cardiovascular System Development and Function	binding	binding of endothelial cell lines	4.50E-06	DCN, F3, HGF, ITGAV, LIPG, SPARC, VCAM1	7
Cardiovascular System Development and Function	survival	survival of endothelial cells	1.41E-05	CDH5, HGF, ITGAV, KDR, OLR1	5
Cardiovascular System Development and Function	cardiovascular process	cardiovascular process of organism	1.58E-05	AGTR1, ANGPT2, CDH5, HGF, ITGAV, KDR, LAMC1, SERPINF1, VCAM1	9
Cardiovascular System Development and Function	cardiovascular process	cardiovascular process of mammalia	2.02E-05	AGTR1, ANGPT2, CDH5, HGF, ITGAV, KDR, SERPINF1, VCAM1	8
Cardiovascular System Development and Function	cardiovascular process	cardiovascular process of blood vessel	3.33E-05	AGTR1, ANGPT2, COL4A2, CSH1, GH1, GH2, HGF, KDR, SERPINF1, SPARC	10
Cardiovascular System Development and Function	cardiovascular process	cardiovascular process of mice	9.18E-05	AGTR1, ANGPT2, CDH5, HGF, ITGAV, KDR, VCAM1	7
Cardiovascular System Development and Function	cardiovascular process	cardiovascular process of tissue	1.21E-04	ADAMTS1, ANGPT2, HGF, SERPINF1, SPARC, SPP1, YAP1	7
Cardiovascular System Development and Function	cardiovascular process	cardiovascular process of cornea	1.99E-04	ADAMTS1, CDH5, HGF, SERPINF1, VCAM1	5
Cardiovascular System Development and Function	electrical resistance	electrical resistance of endothelial cells	1.70E-05	CDH5, HGF, PROCR, SERPINF1	4
Cardiovascular System Development and Function	stroke volume index	stroke volume index of rats	1.38E-04	GH1, HGF	2
Cardiovascular System Development and Function	adhesion	adhesion of endothelial cell lines	1.47E-04	ANGPT2, HGF, ITGAV, KDR, SPP1, VCAM1	6
Cardiovascular System Development and Function	adhesion	adhesion of endothelial cells	3.59E-04	HGF, ITGAV, OLR1, PPAP2B, SPP1, VCAM1	6
Cardiovascular System Development and Function	vascularization	vascularization	1.52E-04	ADAMTS1, CDH5, CGA, HGF, IGFBP3, KDR, SERPINF1, VCAM1	8
Cardiovascular System Development and Function	vascularization	vascularization of cornea	3.06E-03	ADAMTS1, HGF, SERPINF1	3

Highly Expressed in CVS Versus MBC

Table S1H

				¬© 2000-2009 Ingenuity Systems, Inc. All rights reserved.	
**Category**	**Function**	**Function Annotation**	**P-Value**	**Molecules**	**# Molecules**
Cardiovascular System Development and Function	attachment	attachment of endothelial cells	1.82E-04	DCN, TFPI2, VCAM1	3
Cardiovascular System Development and Function	maturation	maturation of blood vessel	2.48E-04	ANGPT2, CDH5, ENPP2	3
Cardiovascular System Development and Function	neovascularization	neovascularization	2.72E-04	CDH5, HGF, IGFBP3, KDR, SERPINF1, VCAM1	6
Cardiovascular System Development and Function	neovascularization	neovascularization of mammalia	8.17E-04	SERPINF1, VCAM1	2
Cardiovascular System Development and Function	neovascularization	neovascularization of retina	1.58E-03	IGFBP3, KDR, SERPINF1	3
Cardiovascular System Development and Function	tubulation	tubulation of endothelial cells	3.06E-04	HGF, IGFBP3, OLR1, SERPINF1	4
Cardiovascular System Development and Function	tubulation	tubulation of endothelial progenitor cells	4.12E-04	IGFBP3, OLR1	2
Cardiovascular System Development and Function	tubulation	tubulation of endothelial cell lines	9.33E-03	CDH5, HGF, ROBO1	3
Cardiovascular System Development and Function	total peripheral resistance	total peripheral resistance of rats	4.12E-04	GH1, HGF	2
Cardiovascular System Development and Function	differentiation	differentiation of endothelial cells	6.57E-04	CDH5, DCN, IGFBP3, KDR	4
Cardiovascular System Development and Function	sprouting	sprouting of blood vessel	6.61E-04	ANGPT2, KDR, OLR1	3
Cardiovascular System Development and Function	sprouting	sprouting of endothelial cells	2.72E-03	ANGPT2, DCN, SPP1	3
Cardiovascular System Development and Function	vasculogenesis	vasculogenesis of mice	1.35E-03	ITGAV, KDR	2
Cardiovascular System Development and Function	vasculogenesis	vasculogenesis	3.49E-03	ITGAV, KDR, RASA1, YAP1	4
Cardiovascular System Development and Function	organization	organization of endothelial cells	2.01E-03	CDH5, KDR	2
Cardiovascular System Development and Function	density	density of microvessel	2.79E-03	HGF, KDR	2
Cardiovascular System Development and Function	morphogenesis	morphogenesis of heart	3.73E-03	COL5A1, DSP, GJA1, PKD2 (includes EG:5311)	4
Cardiovascular System Development and Function	morphogenesis	morphogenesis of blood vessel	8.00E-03	ANGPT2, GJA1, KDR	3
Cardiovascular System Development and Function	proliferation	proliferation of lymphatic endothelial cells	7.09E-03	HGF, KDR	2
Cardiovascular System Development and Function	proliferation	proliferation of endothelial cells	7.20E-03	ADAMTS1, ANGPT2, COL4A1, COL4A2, HGF, KDR	6
Cardiovascular System Development and Function	tubulogenesis	tubulogenesis of endothelial cell lines	8.44E-03	HGF, ITGAV	2
Cardiovascular System Development and Function	tubulogenesis	tubulogenesis of endothelial cells	8.44E-03	HGF, KDR	2
Cardiovascular System Development and Function	thickness	thickness of blood vessel	1.15E-02	AGTR1, SPP1	2
Organismal Development	developmental process	developmental process of mammalia	1.75E-09	ADAMTS1, ANGPT2, CDH5, CGA, COL4A1, COL4A2, DLK1, F3, GCM1, GH1, GHR, H19, HGF, HSD17B2, IGFBP3, ITGAV, KDR, KISS1, PKD2 (includes EG:5311), PLK2, SERPINF1, SNAI2, TWSG1, VCAM1, YAP1	25
Organismal Development	developmental process	developmental process of animal	2.12E-09	ADAMTS1, ANGPT2, CDH5, CGA, COL4A1, COL4A2, DKK1, DLK1, F3, GCM1, GH1, GHR, GJA1, H19, HGF, HSD17B2, IGFBP3, ITGAV, KDR, KISS1, MAFF, PGM3, PKD2 (includes EG:5311), PLK2, RACGAP1, RASA1, SERPINF1, SNAI2, TWIST1, TWSG1, VCAM1, YAP1	32
Organismal Development	developmental process	developmental process of organism	1.72E-08	ADAMTS1, ANGPT2, CDH5, CGA, COL4A1, COL4A2, DKK1, DLK1, F3, GCM1, GH1, GHR, GJA1, H19, HGF, HSD17B2, IGFBP3, INSL4, ITGAV, KDR, KISS1, KRT8, LAMC1, MAFF, PGM3, PKD2 (includes EG:5311), PLK2, RACGAP1, RASA1, SERPINF1, SNAI2, TWIST1, TWSG1, VCAM1, YAP1	35
Organismal Development	developmental process	developmental process of mice	3.07E-08	ADAMTS1, ANGPT2, CDH5, CGA, COL4A1, COL4A2, DLK1, F3, GCM1, GH1, GHR, HGF, HSD17B2, IGFBP3, ITGAV, KDR, KISS1, PKD2 (includes EG:5311), PLK2, SNAI2, TWSG1, VCAM1, YAP1	23
Organismal Development	growth	growth of mammalia	6.54E-06	ADAMTS1, COL4A1, COL4A2, DLK1, F3, GH1, GHR, H19, HSD17B2, IGFBP3, PLK2, TWSG1	12
Organismal Development	growth	growth of mice	2.62E-05	ADAMTS1, COL4A1, COL4A2, DLK1, F3, GH1, GHR, HSD17B2, IGFBP3, PLK2, TWSG1	11
Organismal Development	development	development of organism	9.00E-06	ANGPT2, CDH5, CGA, DKK1, DLK1, GCM1, GJA1, H19, HGF, HSD17B2, INSL4, ITGAV, KDR, KISS1, KRT8, LAMC1, MAFF, PGM3, PKD2 (includes EG:5311), RACGAP1, RASA1, SERPINF1, SNAI2, TWIST1, TWSG1, YAP1	26

				¬© 2000-2009 Ingenuity Systems, Inc. All rights reserved.	
**Category**	**Function**	**Function Annotation**	**P-Value**	**Molecules**	**# Molecules**
Organismal Development	development	development of animal	9.64E-06	ANGPT2, CDH5, CGA, DKK1, GCM1, GJA1, H19, HGF, HSD17B2, ITGAV, KDR, KISS1, MAFF, PGM3, PKD2 (includes EG:5311), RACGAP1, RASA1, SERPINF1, SNAI2, TWIST1, TWSG1, YAP1	22
Organismal Development	development	development of mammalia	3.78E-05	ANGPT2, CDH5, CGA, GCM1, H19, HGF, HSD17B2, ITGAV, KDR, KISS1, PKD2 (includes EG:5311), SERPINF1, SNAI2, YAP1	14
Organismal Development	development	development of mice	4.76E-04	ANGPT2, CDH5, CGA, GCM1, HGF, HSD17B2, ITGAV, KDR, KISS1, PKD2 (includes EG:5311), SNAI2, YAP1	12
Organismal Development	angiogenesis	angiogenesis of organism	7.40E-05	ANGPT2, CDH5, HGF, ITGAV, KDR, LAMC1, SERPINF1	7
Organismal Development	angiogenesis	angiogenesis of mammalia	1.24E-04	ANGPT2, CDH5, HGF, ITGAV, KDR, SERPINF1	6
Organismal Development	angiogenesis	angiogenesis of mice	8.08E-04	ANGPT2, CDH5, HGF, ITGAV, KDR	5
Organismal Development	vascularization	vascularization	1.52E-04	ADAMTS1, CDH5, CGA, HGF, IGFBP3, KDR, SERPINF1, VCAM1	8
Organismal Development	neovascularization	neovascularization	2.72E-04	CDH5, HGF, IGFBP3, KDR, SERPINF1, VCAM1	6
Organismal Development	neovascularization	neovascularization of mammalia	8.17E-04	SERPINF1, VCAM1	2
Organismal Development	vasculogenesis	vasculogenesis of mice	1.35E-03	ITGAV, KDR	2
Organismal Development	length	length of rodents	2.79E-03	GH1, IGFBP3	2
Organismal Development	fibrillogenesis	fibrillogenesis	5.85E-03	LUM, OGN	2
Cellular Movement	migration	migration of tumor cells	2.95E-09	DPP4, ENPP2, F3, GH1, HGF, ITGAV, KDR, SERPINB2, SPARC, VCAM1	10
Cellular Movement	migration	migration of cells	9.92E-08	ANGPT2, CDH1, CDH5, CDK7, COL4A1, COL4A2, CSH1, DCN, DPP4, ENPP2, F3, GH1, GJA1, HGF, IGFBP3, ITGAV, KDR, KISS1, LAMB1, LAMC1, LGMN, OLR1, PITX2, PMP22, PPAP2B, PPIC, PROCR, ROBO1, SDC1, SERPINB2, SLC16A4, SNAI2, SPARC, SPP1, TWIST1, VCAM1	36
Cellular Movement	migration	migration of eukaryotic cells	4.79E-07	ANGPT2, CDH1, CDH5, CDK7, COL4A1, COL4A2, CSH1, DCN, DPP4, ENPP2, F3, GH1, GJA1, HGF, IGFBP3, ITGAV, KDR, KISS1, LAMC1, OLR1, PITX2, PMP22, PPAP2B, PPIC, PROCR, ROBO1, SDC1, SERPINB2, SLC16A4, SPARC, SPP1, TWIST1, VCAM1	33
Cellular Movement	migration	migration of endothelial cells	8.99E-07	ANGPT2, CDH5, COL4A1, COL4A2, DCN, HGF, IGFBP3, ITGAV, KDR, OLR1, SPP1, VCAM1	12
Cellular Movement	migration	migration of tumor cell lines	2.03E-06	CDH1, CDK7, DPP4, ENPP2, GJA1, HGF, IGFBP3, ITGAV, KISS1, PITX2, PPIC, SDC1, SLC16A4, SPP1, TWIST1, VCAM1	16
Cellular Movement	migration	migration of breast cancer cell lines	2.48E-06	GJA1, HGF, IGFBP3, ITGAV, KISS1, PPIC, SDC1, SLC16A4, SPP1	9
Cellular Movement	migration	migration of cell lines	2.64E-06	CDH1, CDH5, CDK7, CSH1, DPP4, ENPP2, GJA1, HGF, IGFBP3, ITGAV, KDR, KISS1, PITX2, PPIC, ROBO1, SDC1, SLC16A4, SPP1, TWIST1, VCAM1	20
Cellular Movement	migration	migration of cancer cells	1.56E-05	DPP4, HGF, ITGAV, KDR, SPARC, VCAM1	6
Cellular Movement	migration	migration of prostate cancer cells	1.58E-05	DPP4, ITGAV, SPARC	3
Cellular Movement	migration	migration of endothelial progenitor cells	4.12E-04	IGFBP3, OLR1	2
Cellular Movement	migration	migration of ovarian cancer cell lines	8.06E-04	CDK7, DPP4, HGF	3
Cellular Movement	migration	migration of normal cells	8.75E-04	ANGPT2, CDH1, CDH5, COL4A1, COL4A2, DCN, F3, GJA1, HGF, IGFBP3, ITGAV, KDR, LAMC1, OLR1, PMP22, PPAP2B, PROCR, SDC1, SPP1, TWIST1, VCAM1	21
Cellular Movement	migration	migration of monocytes	1.23E-03	COL4A1, HGF, ITGAV, SPP1, VCAM1	5
Cellular Movement	migration	migration of fibrosarcoma cell lines	2.40E-03	ITGAV, KISS1, SDC1	3
Cellular Movement	migration	migration of thyroid tumor cell lines	3.70E-03	HGF, KISS1	2
Cellular Movement	migration	migration of glioma cells	5.85E-03	HGF, ITGAV	2
Cellular Movement	migration	migration of lymphatic endothelial cells	5.85E-03	HGF, KDR	2
Cellular Movement	migration	migration of mammary tumor cells	5.85E-03	GH1, ITGAV	2
Cellular Movement	migration	migration of endothelial cell lines	7.44E-03	CDH5, HGF, ITGAV, KDR, ROBO1	5
Cellular Movement	migration	migration of epithelial cells	9.36E-03	CDH1, HGF, ITGAV, PMP22	4
Cellular Movement	migration	migration of squamous cell carcinoma cell lines	9.90E-03	HGF, ITGAV	2
Cellular Movement	invasion	invasion of melanoma cell lines	1.73E-06	ENPP2, HGF, ITGAV, RND3, SPARC, SPP1	6
Cellular Movement	invasion	invasion of tumor cell lines	1.83E-05	CDH1, DPP4, ENPP2, FABP5, HGF, ITGAV, KISS1, PPIC, RND3, SKP2, SPARC, SPP1, TFPI2, TWIST1	14
Cellular Movement	invasion	invasion of cells	7.39E-05	CDH1, DPP4, ENPP2, FABP5, GH1, HGF, ITGAV, KISS1, LGMN, PPIC, RND3, SKP2, SPARC, SPP1, TFPI2, TWIST1	16
Cellular Movement	invasion	invasion of tumor cells	9.58E-05	CDH1, DPP4, ENPP2, GH1, HGF, ITGAV	6
Cellular Movement	invasion	invasion of eukaryotic cells	1.57E-04	CDH1, DPP4, ENPP2, FABP5, GH1, HGF, ITGAV, KISS1, PPIC, RND3, SKP2, SPARC, SPP1, TFPI2, TWIST1	15
Cellular Movement	invasion	invasion of hepatoma cell lines	8.06E-04	HGF, SPP1, TFPI2	3
Cellular Movement	invasion	invasion of cancer cells	1.11E-03	CDH1, DPP4, HGF, ITGAV	4
Cellular Movement	invasion	invasion of breast cancer cell lines	1.66E-03	CDH1, HGF, PPIC, SPARC, SPP1, TWIST1	6
Cellular Movement	invasion	invasion of squamous cell carcinoma cell lines	4.70E-03	HGF, ITGAV, SPP1	3
Cellular Movement	invasion	invasion of melanoma cells	5.85E-03	CDH1, ITGAV	2
Cellular Movement	invasion	invasion of prostate cancer cell lines	1.00E-02	CDH1, FABP5, SPP1	3
Cellular Movement	cell movement	cell movement of osteoclasts	2.48E-04	HGF, ITGAV, SPP1	3
Cellular Movement	cell movement	cell movement of connective tissue cells	1.59E-03	ANGPT2, ENPP2, HGF, ITGAV, SPP1	5
Cellular Movement	cell movement	cell movement of colon cancer cell lines	1.83E-03	CDH1, EPS8, FERMT2	3

				¬© 2000-2009 Ingenuity Systems, Inc. All rights reserved.	
**Category**	**Function**	**Function Annotation**	**P-Value**	**Molecules**	**# Molecules**
Cellular Movement	cell movement	cell movement	1.96E-03	AGTR1, ANGPT2, CDH1, COL4A1, ENPP2, EPS8, F3, FERMT2, GH1, HGF, HSPB1, ITGAV, KDR, KISS1, LIFR, LYVE1, ROBO1, SPARC, SPP1, TLR3, TWIST1, VCAM1	22
Cellular Movement	cell movement	cell movement of cell lines	2.75E-03	AGTR1, CDH1, ENPP2, EPS8, FERMT2, HGF, ITGAV, KISS1, LIFR, SPARC, SPP1	11
Cellular Movement	cell movement	cell movement of glioma cells	2.79E-03	ITGAV, SPP1	2
Cellular Movement	cell movement	cell movement of epidermal cells	5.85E-03	HGF, SPARC	2
Cellular Movement	cell movement	cell movement of tumor cell lines	6.77E-03	CDH1, EPS8, FERMT2, HGF, ITGAV, LIFR, SPARC, SPP1	8
Cellular Movement	cell movement	cell movement of eukaryotic cells	7.70E-03	AGTR1, ANGPT2, CDH1, COL4A1, ENPP2, EPS8, FERMT2, GH1, HGF, ITGAV, KDR, KISS1, LIFR, ROBO1, SPARC, SPP1, TLR3, VCAM1	18
Cellular Movement	cell movement	cell movement of tumor cells	8.65E-03	GH1, ITGAV, SPP1	3
Cellular Movement	cell movement	cell movement of breast cell lines	1.15E-02	HGF, SPP1	2
Cellular Movement	emigration	emigration of Langerhans cells	4.12E-04	ITGAV, SPP1	2
Cellular Movement	emigration	emigration of leukocytes	5.68E-03	ITGAV, SPP1, VCAM1	3
Cellular Movement	infiltration	infiltration of macrophages	5.05E-04	ANGPT2, HGF, KDR, SPP1, TLR3	5
Cellular Movement	chemotaxis	chemotaxis of rhabdomyosarcoma cell lines	1.35E-03	HGF, LIFR	2
Cellular Movement	homing	homing of eukaryotic cells	1.02E-02	AGTR1, ANGPT2, ENPP2, HGF, ITGAV, KDR, KISS1, LIFR, ROBO1, SPP1, VCAM1	11
Reproductive System Development and Function	gestation	gestation	1.42E-08	CSH1, CSH2, GHR, INSL4, PSG1, PSG3, PSG6, PSG7, PSG9	9
Reproductive System Development and Function	size	size of testis	1.16E-04	CGA, CYP11A1, GJA1, KISS1	4
Reproductive System Development and Function	size	size of pituitary gland	2.01E-03	GHR, PITX2	2
Reproductive System Development and Function	size	size of ovary	2.79E-03	GJA1, KISS1	2
Reproductive System Development and Function	binding	binding of gonadal cell lines	1.44E-04	F3, FERMT2, OLR1, SDC1, VCAM1	5
Reproductive System Development and Function	mass	mass of seminal vesicle	3.28E-04	CGA, GH1, GHR	3
Reproductive System Development and Function	mass	mass of uterus	7.09E-03	CGA, KISS1	2
Reproductive System Development and Function	mass	mass of gonad	1.00E-02	CGA, GHR, KISS1	3
Reproductive System Development and Function	morphogenesis	morphogenesis of mammary alveolus	4.12E-04	CDH1, DSG2	2
Reproductive System Development and Function	proliferation	proliferation of breast cell lines	5.85E-04	CDH1, CSH1, IGFBP3, YAP1	4
Reproductive System Development and Function	development	development of mammary alveolus	1.15E-03	CDH1, DSG2, GH1	3
Reproductive System Development and Function	development	development of uterus	3.70E-03	ADAMTS1, KISS1	2
Reproductive System Development and Function	development	development of ovarian follicle	4.83E-03	CGA, GJA1, KDR, KISS1	4
Reproductive System Development and Function	development	development of gonad	6.48E-03	ADAMTS1, CGA, KDR, SNAI2	4
Reproductive System Development and Function	opening	opening of vagina	2.01E-03	GHR, KISS1	2
Reproductive System Development and Function	reproductive process	reproductive process of mice	7.68E-03	CGA, GHR, HSD17B2, KDR, KISS1, SPP1	6
Reproductive System Development and Function	quantity	quantity of ovarian follicle	1.08E-02	ADAMTS1, CGA, GHR	3
Reproductive System Development and Function	cell movement	cell movement of breast cell lines	1.15E-02	HGF, SPP1	2
Cellular Growth and Proliferation	proliferation	proliferation of cells	9.23E-08	ADAMTS1, AGTR1, ANGPT2, CDH1, CDH5, CDK7, CGA, CKS2, COL4A1, COL4A2, CSH1, CSH2, DCN, DKK1, DLK1, DPP4, EBI3, ENPEP, ENPP2, EPS8, F3, FABP5, GH1, GH2, GHR, GJA1, H19, HGF, HS6ST2, HSD11B2, IGFBP3, INSL4, ITGAV, KDR, KISS1, KRT8, LAMB1, LAMC1, LIFR, LIPG, PEG10, PITX2, PLA2G2A, PMP22, SERPINB2, SERPINF1, SKP2, SNAI2, SPARC, SPP1, STS, TFPI2, TLR3, VCAM1, YAP1	55
Cellular Growth and Proliferation	proliferation	proliferation of tumor cell lines	5.39E-06	CDH1, CKS2, CSH1, CSH2, DCN, DLK1, EPS8, FABP5, GH1, GH2, GHR, GJA1, HGF, HSD11B2, IGFBP3, ITGAV, KDR, KISS1, SERPINB2, SKP2, SPARC, SPP1, TFPI2	23

				¬© 2000-2009 Ingenuity Systems, Inc. All rights reserved.	
**Category**	**Function**	**Function Annotation**	**P-Value**	**Molecules**	**# Molecules**
Cellular Growth and Proliferation	proliferation	proliferation of eukaryotic cells	5.62E-06	ADAMTS1, AGTR1, ANGPT2, CDH1, CGA, CKS2, COL4A1, COL4A2, CSH1, CSH2, DCN, DKK1, DLK1, DPP4, EBI3, EPS8, F3, FABP5, GH1, GH2, GHR, GJA1, HGF, HS6ST2, HSD11B2, IGFBP3, ITGAV, KDR, KISS1, LAMB1, LAMC1, LIFR, PEG10, PLA2G2A, SERPINB2, SKP2, SNAI2, SPARC, SPP1, TFPI2, TLR3, VCAM1, YAP1	43
Cellular Growth and Proliferation	proliferation	proliferation of cell lines	1.19E-04	AGTR1, CDH1, CKS2, CSH1, CSH2, DCN, DLK1, EPS8, FABP5, GH1, GH2, GHR, GJA1, HGF, HSD11B2, IGFBP3, ITGAV, KDR, KISS1, LIFR, SERPINB2, SKP2, SPARC, SPP1, TFPI2, YAP1	26
Cellular Growth and Proliferation	proliferation	proliferation of beta islet cells	1.95E-04	CSH1, GH1, HGF, IGFBP3	4
Cellular Growth and Proliferation	proliferation	proliferation of connective tissue cells	2.61E-04	CGA, DCN, GHR, GJA1, HGF, HS6ST2, IGFBP3, ITGAV, SKP2, SPARC, SPP1	11
Cellular Growth and Proliferation	proliferation	proliferation of breast cell lines	5.85E-04	CDH1, CSH1, IGFBP3, YAP1	4
Cellular Growth and Proliferation	proliferation	proliferation of endocrine cells	5.94E-04	ANGPT2, CSH1, GH1, HGF, IGFBP3	5
Cellular Growth and Proliferation	proliferation	proliferation of normal cells	6.57E-04	ADAMTS1, ANGPT2, CGA, COL4A1, COL4A2, CSH1, CSH2, DCN, DKK1, DPP4, EBI3, F3, GH1, GHR, GJA1, HGF, HS6ST2, IGFBP3, ITGAV, KDR, LAMB1, LAMC1, PLA2G2A, SKP2, SNAI2, SPARC, SPP1, TLR3, VCAM1	29
Cellular Growth and Proliferation	proliferation	proliferation of lymphoma cell lines	6.95E-04	CSH1, CSH2, GH1, GH2, IGFBP3	5
Cellular Growth and Proliferation	proliferation	proliferation of epithelial cells	1.79E-03	DKK1, HGF, KDR, LAMB1, LAMC1, PLA2G2A, SKP2, SNAI2, SPARC	9
Cellular Growth and Proliferation	proliferation	proliferation of stromal cells	2.10E-03	CGA, HGF, IGFBP3	3
Cellular Growth and Proliferation	proliferation	proliferation of mesenchymal stem cells	2.79E-03	DKK1, HGF	2
Cellular Growth and Proliferation	proliferation	proliferation of tubular cells	2.79E-03	HGF, SKP2	2
Cellular Growth and Proliferation	proliferation	proliferation of lymphatic endothelial cells	7.09E-03	HGF, KDR	2
Cellular Growth and Proliferation	proliferation	proliferation of endothelial cells	7.20E-03	ADAMTS1, ANGPT2, COL4A1, COL4A2, HGF, KDR	6
Cellular Growth and Proliferation	proliferation	proliferation of smooth muscle cells	7.68E-03	F3, HGF, IGFBP3, SKP2, SPARC, SPP1	6
Cellular Growth and Proliferation	proliferation	proliferation of osteoclasts	8.44E-03	HGF, ITGAV	2
Cellular Growth and Proliferation	proliferation	proliferation of bone marrow cells	8.46E-03	DKK1, HGF, IGFBP3, SPP1	4
Cellular Growth and Proliferation	proliferation	proliferation of kidney cells	8.65E-03	HGF, SKP2, SPARC	3
Cellular Growth and Proliferation	proliferation	proliferation of breast cancer cell lines	8.72E-03	CDH1, DCN, GH1, IGFBP3, KISS1, SKP2	6
Cellular Growth and Proliferation	proliferation	proliferation of hepatic stellate cells	1.15E-02	ITGAV, SPP1	2
Cellular Growth and Proliferation	growth	growth of cells	1.75E-04	AGTR1, CDH1, CDH5, CDK7, COL6A3, DCN, DLK1, DPP4, DSP, DUSP9, EBI3, FBN1, GH1, GHR, GJA1, GPX3, HGF, HSD11B2, HTRA1, IGFBP3, ITGAV, KDR, KISS1, LAMB1, LAMC1, MAFF, MEST, PEG10, PLA2G2A, PLK2, PMP22, RASA1, SERPINF1, SKP2, SPARC, SPP1, TLR3, YAP1	38
Cellular Growth and Proliferation	growth	growth of cancer cells	2.87E-04	CDH5, DCN, HGF, ITGAV, KDR, PEG10, SKP2	7
Cellular Growth and Proliferation	growth	growth of breast cancer cell lines	8.51E-04	CDH1, COL6A3, DCN, GJA1, HGF, IGFBP3, SKP2, SPP1	8
Cellular Growth and Proliferation	hypertrophy	hypertrophy of neuroendocrine cells	1.35E-03	CGA, SKP2	2
Cellular Growth and Proliferation	formation	formation of connective tissue cells	2.82E-03	DKK1, DLK1, HGF, LGMN, SPARC	5
Cellular Growth and Proliferation	formation	formation of adipocytes	3.70E-03	DLK1, SPARC	2
Cellular Growth and Proliferation	formation	formation of eukaryotic cells	5.61E-03	ANLN, CDH1, DKK1, DLK1, ENPP2, HGF, LAMC1, LGMN, SPARC	9
Cellular Growth and Proliferation	formation	formation of gonadal cells	7.09E-03	CDH1, HGF	2
Dermatological Diseases and Conditions	burn	burn	1.31E-07	COL15A1, COL21A1, COL3A1, COL4A1, COL4A2, COL5A1, COL5A2, COL6A3	8
Dermatological Diseases and Conditions	Ehlers-Danlos syndrome, type I	Ehlers-Danlos syndrome, type I	4.12E-04	COL5A1, COL5A2	2
Dermatological Diseases and Conditions	Ehlers-Danlos syndrome	Ehlers-Danlos syndrome	4.22E-04	COL3A1, COL5A1, COL5A2	3
Dermatological Diseases and Conditions	dermatological disorder	dermatological disorder	2.67E-03	CDH1, COL15A1, COL21A1, COL3A1, COL4A1, COL4A2, COL5A1, COL5A2, COL6A3, DSP, ENPP2, GJA1, HGF, KDR, KRT8, SNAI2, TWIST1	17
Dermatological Diseases and Conditions	neoplasia	neoplasia of skin	3.70E-03	SERPINB2, SPP1	2
Dermatological Diseases and Conditions	cell death	cell death of epithelial cell lines	4.11E-03	ABCG2, CDH1, HSPB1, IGFBP3, ITGAV, PMP22, SERPINF1, SPP1	8
Dermatological Diseases and Conditions	disease	disease of skin	4.85E-03	CDH1, HGF, KDR, SERPINB2, SPP1	5
Dermatological Diseases and Conditions	invasion	invasion of melanoma cells	5.85E-03	CDH1, ITGAV	2
Dermatological Diseases and Conditions	Ehlers-Danlos Syndrome Type IV	Ehlers-Danlos Syndrome Type IV	1.18E-02	COL3A1	1
Dermatological Diseases and Conditions	Ehlers-Danlos syndrome, type II	Ehlers-Danlos syndrome, type II	1.18E-02	COL5A1	1
Connective Tissue Disorders	Dupuytren contracture	Dupuytren contracture	1.41E-07	ADAMTS1, COL15A1, COL21A1, COL3A1, COL4A1, COL4A2, COL5A1, COL5A2, COL6A3	9
Connective Tissue Disorders	Ehlers-Danlos syndrome, type I	Ehlers-Danlos syndrome, type I	4.12E-04	COL5A1, COL5A2	2
Connective Tissue Disorders	Ehlers-Danlos syndrome	Ehlers-Danlos syndrome	4.22E-04	COL3A1, COL5A1, COL5A2	3
Connective Tissue Disorders	Bruck syndrome	Bruck syndrome	1.18E-02	PLOD2	1

¬© 2000-2009 Ingenuity Systems, Inc. All rights reserved.

Category	Function	Function Annotation	P-Value	Molecules	# Molecules
Connective Tissue Disorders	Ehlers-Danlos Syndrome Type IV	Ehlers-Danlos Syndrome Type IV	1.18E-02	COL3A1	1
Connective Tissue Disorders	Ehlers-Danlos syndrome, type II	Ehlers-Danlos syndrome, type II	1.18E-02	COL5A1	1
Genetic Disorder	Dupuytren contracture	Dupuytren contracture	1.41E-07	ADAMTS1, COL15A1, COL21A1, COL3A1, COL4A1, COL4A2, COL5A1, COL5A2, COL6A3	9
Genetic Disorder	colon cancer	colon cancer	1.69E-04	ADAMTS1, CDK7, COL3A1, EBI3, GGH, GJA1, GMNN, HSD11B2, HSD17B2, HSPE1, PLA2G2A, PVRL3, SLC27A2, SPP1	14
Genetic Disorder	Ehlers-Danlos syndrome, type I	Ehlers-Danlos syndrome, type I	4.12E-04	COL5A1, COL5A2	2
Genetic Disorder	glycine encephalopathy	glycine encephalopathy	4.12E-04	GCSH, GLDC	2
Genetic Disorder	Ehlers-Danlos syndrome	Ehlers-Danlos syndrome	4.22E-04	COL3A1, COL5A1, COL5A2	3
Genetic Disorder	Marfan's syndrome	Marfan's syndrome	8.17E-04	AGTR1, FBN1	2
Genetic Disorder	age-related macular degeneration-wet form	age-related macular degeneration-wet form	1.35E-03	HTRA1, KDR	2
Genetic Disorder	Budd-Chiari syndrome	Budd-Chiari syndrome	1.83E-03	ANGPT2, COL4A1, F3	3
Genetic Disorder	gonadal dysgenesis	gonadal dysgenesis	2.79E-03	GH1, GHR	2
Genetic Disorder	arrhythmogenic right ventricular dysplasia	arrhythmogenic right ventricular dysplasia	3.70E-03	DSG2, DSP	2
Genetic Disorder	genetic disorder	genetic disorder of mice	5.71E-03	DLK1, DPP4, EBI3, GCM1, GH1, GJA1, H19, HSD11B2, IGFBP3, KRT8, PKD2 (includes EG:5311), SDC1, SNAI2, SPP1, TWSG1	15
Genetic Disorder	diabetic nephropathy	diabetic nephropathy	7.09E-03	AGTR1, SPP1	2
Genetic Disorder	prostate cancer	prostate cancer	1.02E-02	AGTR1, ASS1, CDH1, CDK7, FBN1, GPX3, HAPLN1, IGFBP3, ITGAV, KDR, SMS, SPP1	12
Genetic Disorder	Bruck syndrome	Bruck syndrome	1.18E-02	PLOD2	1
Genetic Disorder	Ehlers-Danlos Syndrome Type IV	Ehlers-Danlos Syndrome Type IV	1.18E-02	COL3A1	1
Genetic Disorder	Ehlers-Danlos syndrome, type II	Ehlers-Danlos syndrome, type II	1.18E-02	COL5A1	1
Genetic Disorder	Rieger syndrome	Rieger syndrome	1.18E-02	PITX2	1
Organismal Survival	death	death of mice	2.21E-07	AGTR1, ANGPT2, CDH1, CDH5, CKS2, COL4A1, COL4A2, COL5A1, CYP11A1, DCN, DSP, ENPP2, F3, FBN1, GH1, GJA1, HAPLN1, HGF, HSD11B2, HSPE1, ITGAV, KDR, KRT8, LIFR, MAFF, PITX2, RASA1, ROBO1, TLR3, TWSG1, VCAM1	31
Organismal Survival	survival	survival of mice	2.18E-03	AGTR1, COL3A1, DKK1, DSP, F3, HGF, KDR, KRT18, LAMC1, PKD2 (includes EG:5311), SKP2, TLR3	12
Tissue Development	formation	formation of tissue	4.49E-07	ANGPT2, CDH1, COL4A1, COL4A2, DCN, DKK1, DLK1, GH1, HGF, HSD11B2, IGFBP3, KDR, SPARC, SPP1, TWSG1, VCAM1	16
Tissue Development	formation	formation of bone	5.47E-05	DCN, DLK1, GH1, HSD11B2, IGFBP3, SPARC, SPP1	7
Tissue Development	formation	formation of trabecular bone	4.71E-03	IGFBP3, SPP1	2
Tissue Development	adhesion	adhesion of cells	1.60E-06	ANGPT2, CDH1, CDH5, COL5A1, DCN, DSG2, F3, GH1, HGF, ITGAV, KDR, LAMB1, LAMC1, LIPG, NID2, OLR1, PITX2, PPAP2B, PVRL3, RASA1, RND3, ROBO1, SDC1, SERPINB2, SPP1, TWIST1, VCAM1	27
Tissue Development	adhesion	adhesion of eukaryotic cells	5.28E-05	ANGPT2, CDH1, DCN, DSG2, F3, GH1, HGF, ITGAV, KDR, LIPG, OLR1, PITX2, PPAP2B, PVRL3, SDC1, SERPINB2, SPP1, VCAM1	18
Tissue Development	adhesion	adhesion of endothelial cells	3.59E-04	HGF, ITGAV, OLR1, PPAP2B, SPP1, VCAM1	6
Tissue Development	adhesion	adhesion of extracellular matrix	6.61E-04	CDH1, ITGAV, SPP1	3
Tissue Development	developmental process	developmental process of tissue	1.03E-04	ADAMTS1, ANGPT2, CDH1, COL6A3, ENPP2, GH1, GHR, GJA1, HGF, LAMB1, LAMC1, LGMN, LUM, MEST, PITX2, ROBO1, SERPINF1, SGCE, SNAI2, SPARC, SPP1, TWIST1, TWSG1, VCAM1, YAP1	25
Tissue Development	developmental process	developmental process of connective tissue	1.01E-03	ADAMTS1, GH1, GHR, GJA1, LGMN, PITX2, SPARC, SPP1, TWSG1, VCAM1	10
Tissue Development	developmental process	developmental process of bone	4.46E-03	GH1, GHR, GJA1, LGMN, PITX2, SPARC, SPP1, TWSG1	8
Tissue Development	developmental process	developmental process of muscle	6.01E-03	CDH1, COL6A3, GJA1, HGF, PITX2, SGCE, SNAI2, TWIST1, VCAM1	9
Tissue Development	developmental process	developmental process of epithelial tissue	9.04E-03	CDH1, LAMB1, PITX2, SPP1, YAP1	5
Tissue Development	angiogenesis	angiogenesis of tissue	6.43E-04	ADAMTS1, ANGPT2, HGF, SERPINF1, SPARC	5
Tissue Development	development	development of tissue	9.41E-04	ADAMTS1, ANGPT2, COL6A3, ENPP2, GJA1, HGF, LAMB1, LAMC1, LUM, MEST, PITX2, ROBO1, SERPINF1, SGCE, SPARC, SPP1, TWIST1, TWSG1, VCAM1, YAP1	20
Tissue Development	development	development of extraembryonic tissue	6.85E-03	ADAMTS1, ENPP2, HGF, YAP1	4
Tissue Development	development	development of fetal membranes	7.37E-03	ADAMTS1, ENPP2, HGF	3
Tissue Development	accumulation	accumulation of monocytes	2.01E-03	RASA1, VCAM1	2
Tissue Development	accumulation	accumulation of cells	6.28E-03	CGA, GH1, HGF, KDR, KRT18, RASA1, TLR3, VCAM1	8
Tissue Development	growth	growth of connective tissue	3.83E-03	GH1, GHR, GJA1	3
Tissue Development	deposition	deposition of connective tissue	4.71E-03	GJA1, SPARC	2
Cell-To-Cell Signaling and Interaction	binding	binding of connective tissue cells	7.47E-07	DCN, DKK1, IGFBP3, ITGAV, SPARC, VCAM1	6
Cell-To-Cell Signaling and Interaction	binding	binding of endothelial cell lines	4.50E-06	DCN, F3, HGF, ITGAV, LIPG, SPARC, VCAM1	7
Cell-To-Cell Signaling and Interaction	binding	binding of cell lines	5.79E-06	DCN, DPP4, F3, FERMT2, HGF, IGFBP3, ITGAV, LIPG, OLR1, SDC1, SERPINF1, SPARC, SPP1, VCAM1	14

¬© 2000-2009 Ingenuity Systems, Inc. All rights reserved.

Category	Function	Function Annotation	P-Value	Molecules	# Molecules
Cell-To-Cell Signaling and Interaction	binding	binding of tumor cell lines	2.20E-05	DCN, DPP4, F3, FERMT2, HGF, IGFBP3, ITGAV, SERPINF1, SPP1, VCAM1	10
Cell-To-Cell Signaling and Interaction	binding	binding of cells	6.14E-05	CDH5, DCN, DKK1, DPP4, F3, FERMT2, HGF, IGFBP3, ITGAV, LIPG, OLR1, RASA1, SDC1, SERPINF1, SPARC, SPP1, VCAM1	17
Cell-To-Cell Signaling and Interaction	binding	binding of osteoblasts	8.64E-05	DKK1, IGFBP3, VCAM1	3
Cell-To-Cell Signaling and Interaction	binding	binding of gonadal cell lines	1.44E-04	F3, FERMT2, OLR1, SDC1, VCAM1	5
Cell-To-Cell Signaling and Interaction	binding	binding of eukaryotic cells	3.35E-04	DCN, DKK1, DPP4, F3, FERMT2, HGF, IGFBP3, ITGAV, LIPG, OLR1, SDC1, SERPINF1, SPARC, SPP1, VCAM1	15
Cell-To-Cell Signaling and Interaction	binding	binding of fibroblasts	8.06E-04	IGFBP3, ITGAV, SPARC	3
Cell-To-Cell Signaling and Interaction	binding	binding of tumor cells	2.10E-03	DKK1, ITGAV, VCAM1	3
Cell-To-Cell Signaling and Interaction	binding	binding of normal cells	1.02E-02	DCN, DKK1, F3, HGF, IGFBP3, ITGAV, LIPG, SPARC, VCAM1	9
Cell-To-Cell Signaling and Interaction	adhesion	adhesion of cells	1.60E-06	ANGPT2, CDH1, CDH5, COL5A1, DCN, DSG2, F3, GH1, HGF, ITGAV, KDR, LAMB1, LAMC1, LIPG, NID2, OLR1, PITX2, PPAP2B, PVRL3, RASA1, RND3, ROBO1, SDC1, SERPINB2, SPP1, TWIST1, VCAM1	27
Cell-To-Cell Signaling and Interaction	adhesion	adhesion of cell-associated matrix	3.57E-05	COL3A1, LYVE1, NID2, PKD2 (includes EG:5311), RASA1, SGCE	6
Cell-To-Cell Signaling and Interaction	adhesion	adhesion of tumor cell lines	3.92E-05	CDH1, DCN, DSG2, HGF, ITGAV, PITX2, PVRL3, SDC1, SERPINB2, SPP1, VCAM1	11
Cell-To-Cell Signaling and Interaction	adhesion	adhesion of cell lines	4.91E-05	ANGPT2, CDH1, DCN, DSG2, HGF, ITGAV, KDR, PITX2, PVRL3, SDC1, SERPINB2, SPP1, VCAM1	13
Cell-To-Cell Signaling and Interaction	adhesion	adhesion of eukaryotic cells	5.28E-05	ANGPT2, CDH1, DCN, DSG2, F3, GH1, HGF, ITGAV, KDR, LIPG, OLR1, PITX2, PPAP2B, PVRL3, SDC1, SERPINB2, SPP1, VCAM1	18
Cell-To-Cell Signaling and Interaction	adhesion	adhesion of endothelial cell lines	1.47E-04	ANGPT2, HGF, ITGAV, KDR, SPP1, VCAM1	6
Cell-To-Cell Signaling and Interaction	adhesion	adhesion of endothelial cells	3.59E-04	HGF, ITGAV, OLR1, PPAP2B, SPP1, VCAM1	6
Cell-To-Cell Signaling and Interaction	adhesion	adhesion of stem cells	2.01E-03	CDH1, ITGAV	2
Cell-To-Cell Signaling and Interaction	adhesion	adhesion of connective tissue cells	2.42E-03	CDH1, HGF, ITGAV, VCAM1	4
Cell-To-Cell Signaling and Interaction	adhesion	adhesion of leukemia cell lines	3.73E-03	CDH1, SERPINB2, SPP1, VCAM1	4
Cell-To-Cell Signaling and Interaction	adhesion	adhesion of colon cancer cell lines	5.18E-03	CDH1, DSG2, HGF	3
Cell-To-Cell Signaling and Interaction	adhesion	adhesion of lymphoma cell lines	8.00E-03	HGF, SDC1, VCAM1	3
Cell-To-Cell Signaling and Interaction	adhesion	adhesion of tumor cells	8.00E-03	F3, ITGAV, VCAM1	3
Cell-To-Cell Signaling and Interaction	adhesion	adhesion of embryonic cells	8.44E-03	CDH1, ITGAV	2
Cell-To-Cell Signaling and Interaction	adhesion	adhesion of melanoma cell lines	8.44E-03	ITGAV, SPP1	2
Cell-To-Cell Signaling and Interaction	adhesion	adhesion of normal cells	9.65E-03	CDH1, F3, GH1, HGF, ITGAV, LIPG, OLR1, PPAP2B, SPP1, VCAM1	10
Cell-To-Cell Signaling and Interaction	adhesion	adhesion of kidney cell lines	1.00E-02	CDH1, HGF, ITGAV	3
Cell-To-Cell Signaling and Interaction	cell-cell contact	cell-cell contact of tumor cells	1.38E-04	CDH1, GH1	2
Cell-To-Cell Signaling and Interaction	cell-cell contact	cell-cell contact of eukaryotic cells	4.58E-04	CDH1, CDH5, GH1, HGF	4
Cell-To-Cell Signaling and Interaction	cell-cell contact	cell-cell contact of epithelial cells	2.01E-03	CDH1, HGF	2
Cell-To-Cell Signaling and Interaction	cell-cell contact	cell-cell contact of normal cells	2.10E-03	CDH1, CDH5, HGF	3
Cell-To-Cell Signaling and Interaction	cell-cell contact	cell-cell contact	5.07E-03	CDH1, CDH5, GH1, HGF, PVRL3	5

				¬© 2000-2009 Ingenuity Systems, Inc. All rights reserved.	
**Category**	**Function**	**Function Annotation**	**P-Value**	**Molecules**	**# Molecules**
Cell-To-Cell Signaling and Interaction	cell-cell contact	cell-cell contact of cell lines	8.44E-03	CDH1, CDH5	2
Cell-To-Cell Signaling and Interaction	attachment	attachment of endothelial cells	1.82E-04	DCN, TFPI2, VCAM1	3
Cell-To-Cell Signaling and Interaction	attachment	attachment of normal cells	3.49E-03	DCN, ITGAV, TFPI2, VCAM1	4
Cell-To-Cell Signaling and Interaction	attachment	attachment of cells	5.54E-03	DCN, ITGAV, SPP1, TFPI2, VCAM1	5
Cell-To-Cell Signaling and Interaction	quantity	quantity of adherens junctions	4.12E-04	CDH1, DSP	2
Cell-To-Cell Signaling and Interaction	quantity	quantity of intercellular junctions	2.40E-03	CDH1, DSP, GJA1	3
Cell-To-Cell Signaling and Interaction	quantity	quantity of focal adhesions	4.25E-03	CDH5, SPARC, SPP1	3
Cell-To-Cell Signaling and Interaction	activation	activation of cell lines	4.92E-04	CDH1, ENPEP, F3, HGF, KRT8, KRT18, TLR3	7
Cell-To-Cell Signaling and Interaction	activation	activation of fibroblast cell lines	3.70E-03	KRT8, KRT18	2
Cell-To-Cell Signaling and Interaction	activation	activation of kidney cell lines	3.70E-03	HGF, TLR3	2
Cell-To-Cell Signaling and Interaction	disruption	disruption of adherens junctions	2.79E-03	CDH1, HGF	2
Cell-To-Cell Signaling and Interaction	association	association of eukaryotic cells	4.71E-03	GHR, IGFBP3	2
Cell-To-Cell Signaling and Interaction	formation	formation of focal adhesions	7.14E-03	FERMT2, HGF, KISS1, NET1, RND3	5
Cell-To-Cell Signaling and Interaction	cell-cell adhesion	cell-cell adhesion of tumor cell lines	8.44E-03	CDH1, HGF	2
Reproductive System Disease	migration	migration of breast cancer cell lines	2.48E-06	GJA1, HGF, IGFBP3, ITGAV, KISS1, PPIC, SDC1, SLC16A4, SPP1	9
Reproductive System Disease	migration	migration of prostate cancer cells	1.58E-05	DPP4, ITGAV, SPARC	3
Reproductive System Disease	migration	migration of ovarian cancer cell lines	8.06E-04	CDK7, DPP4, HGF	3
Reproductive System Disease	migration	migration of mammary tumor cells	5.85E-03	GH1, ITGAV	2
Reproductive System Disease	reproductive system disorder	reproductive system disorder	3.55E-06	ANGPT2, CYP11A1, DCN, DKK1, F3, FBN1, GH1, GHR, GPX3, HGF, HSD17B2, HSPB1, KDR, KRT8, LGMN, MAOA, MEST, PKD2 (includes EG:5311), PLK2, PPAP2B, SPP1, STS	22
Reproductive System Disease	endometriosis	endometriosis	1.30E-05	ANGPT2, DCN, DKK1, FBN1, GPX3, HGF, HSD17B2, HSPB1, KDR, KRT8, MAOA, MEST, PKD2 (includes EG:5311), PLK2, PPAP2B, SPP1, STS	17
Reproductive System Disease	morphogenesis	morphogenesis of breast cancer cell lines	1.38E-04	CDH1, HGF	2
Reproductive System Disease	metastasis	metastasis of breast cancer cell lines	3.28E-04	FABP5, HGF, TWIST1	3
Reproductive System Disease	serous ovarian carcinoma	serous ovarian carcinoma	5.18E-04	COL15A1, DPP4, ENPEP, GPX3, MFAP5, RACGAP1, TFPI2	7
Reproductive System Disease	ovarian cancer	ovarian cancer	7.28E-04	CDH1, COL15A1, COL4A1, DPP4, ENPEP, GPX3, H19, KDR, MFAP5, RACGAP1, SPP1, TFPI2	12
Reproductive System Disease	degeneration	degeneration of seminiferous tubules	8.17E-04	CGA, KISS1	2
Reproductive System Disease	growth	growth of breast cancer cell lines	8.51E-04	CDH1, COL6A3, DCN, GJA1, HGF, IGFBP3, SKP2, SPP1	8
Reproductive System Disease	cell death	cell death of breast cancer cell lines	1.07E-03	ABCG2, CDH1, GH1, HGF, IGFBP3, ITGAV, KRT18, PLK2, RASA1, SDC1	10
Reproductive System Disease	G1 phase	G1 phase of ovarian cancer cell lines	1.35E-03	ITGAV, SKP2	2
Reproductive System Disease	apoptosis	apoptosis of breast cancer cell lines	1.54E-03	CDH1, GH1, HGF, IGFBP3, ITGAV, KRT18, PLK2, RASA1, SDC1	9
Reproductive System Disease	invasion	invasion of breast cancer cell lines	1.66E-03	CDH1, HGF, PPIC, SPARC, SPP1, TWIST1	6
Reproductive System Disease	invasion	invasion of prostate cancer cell lines	1.00E-02	CDH1, FABP5, SPP1	3
Reproductive System Disease	genital tumor	genital tumor	2.36E-03	AGTR1, ASS1, CDK7, COL15A1, DPP4, ENPEP, GPX3, IGFBP3, ITGAV, KDR, MFAP5, RACGAP1, SMS, TFPI2	14
Reproductive System Disease	gonadal dysgenesis	gonadal dysgenesis	2.79E-03	GH1, GHR	2
Reproductive System Disease	prostatic intraepithelial tumor	prostatic intraepithelial tumor	3.65E-03	AGTR1, ASS1, GPX3, IGFBP3, SMS	5
Reproductive System Disease	ovarian tumor	ovarian tumor	5.82E-03	COL15A1, DPP4, ENPEP, GPX3, KDR, MFAP5, RACGAP1, TFPI2	8
Reproductive System Disease	uterine tumor	uterine tumor	6.96E-03	CDH1, CDK7, CETN3, COL6A3, FBN1, H19, KDR, LIFR, MAOA, MEST	10
Reproductive System Disease	proliferation	proliferation of breast cancer cell lines	8.72E-03	CDH1, DCN, GH1, IGFBP3, KISS1, SKP2	6
Reproductive System Disease	prostate cancer	prostate cancer	1.02E-02	AGTR1, ASS1, CDH1, CDK7, FBN1, GPX3, HAPLN1, IGFBP3, ITGAV, KDR, SMS, SPP1	12
Reproductive System Disease	morphology	morphology of prostate cancer cell lines	1.15E-02	CDH1, TWIST1	2
Reproductive System Disease	abortion	abortion of mice	1.18E-02	F3	1
Reproductive System Disease	accumulation	accumulation of prostate cancer cell lines	1.18E-02	HGF	1
Cell Morphology	electrical resistance	electrical resistance of cells	7.61E-06	CDH5, HGF, PMP22, PROCR, SERPINF1	5

Highly Expressed in CVS Versus MBC

Table S1H

				¬© 2000-2009 Ingenuity Systems, Inc. All rights reserved.	
**Category**	**Function**	**Function Annotation**	**P-Value**	**Molecules**	**# Molecules**
Cell Morphology	electrical resistance	electrical resistance of endothelial cells	1.70E-05	CDH5, HGF, PROCR, SERPINF1	4
Cell Morphology	morphogenesis	morphogenesis of cells	1.53E-05	ANGPT2, CDH1, DCN, FBN1, HGF, HSPB1, ITGAV, KDR, KRT8, PITX2, PMP22, RASA1, RND3, ROBO1, SDC1, SNAI2, SPARC, SPP1, VCAM1	19
Cell Morphology	morphogenesis	morphogenesis of breast cancer cell lines	1.38E-04	CDH1, HGF	2
Cell Morphology	morphogenesis	morphogenesis of normal cells	1.00E-02	CDH1, HGF, VCAM1	3
Cell Morphology	shape change	shape change of eukaryotic cells	4.45E-05	ANGPT2, DCN, FBN1, HGF, ITGAV, KDR, PITX2, RND3, ROBO1, SDC1, SNAI2, SPARC, SPP1, VCAM1	14
Cell Morphology	shape change	shape change	5.68E-05	ANGPT2, DCN, FBN1, HGF, HSPB1, ITGAV, KDR, OLR1, PITX2, PMP22, RASA1, RND3, ROBO1, SDC1, SNAI2, SPARC, SPP1, VCAM1	18
Cell Morphology	shape change	shape change of normal cells	1.42E-03	ANGPT2, DCN, FBN1, HGF, ITGAV, ROBO1, SPARC, SPP1	8
Cell Morphology	shape change	shape change of cell lines	2.65E-03	ITGAV, KDR, PITX2, RND3, SDC1, SNAI2, SPP1, VCAM1	8
Cell Morphology	tubulation	tubulation of eukaryotic cells	7.98E-05	CDH5, HGF, IGFBP3, OLR1, ROBO1, SERPINF1	6
Cell Morphology	tubulation	tubulation of endothelial cells	3.06E-04	HGF, IGFBP3, OLR1, SERPINF1	4
Cell Morphology	tubulation	tubulation of endothelial progenitor cells	4.12E-04	IGFBP3, OLR1	2
Cell Morphology	tubulation	tubulation of endothelial cell lines	9.33E-03	CDH5, HGF, ROBO1	3
Cell Morphology	morphology	morphology of eukaryotic cells	1.35E-04	CDH1, CDH5, COL15A1, DPP4, GH1, GJA1, HGF, IGFBP3, KRT8, KRT18, PEG10, PMP22, RASA1, SPARC, TWIST1	15
Cell Morphology	morphology	morphology of cells	1.46E-04	CDH1, CDH5, COL15A1, DPP4, FERMT2, GH1, GJA1, HGF, IGFBP3, KRT8, KRT18, PEG10, PMP22, RASA1, SPARC, TWIST1	16
Cell Morphology	morphology	morphology of filaments	4.22E-04	COL5A1, EPS8, LUM	3
Cell Morphology	morphology	morphology of fibrils	8.17E-04	COL5A1, LUM	2
Cell Morphology	morphology	morphology of cell lines	2.06E-03	CDH1, DPP4, HGF, IGFBP3, KRT8, KRT18, PEG10, PMP22, TWIST1	9
Cell Morphology	morphology	morphology of kidney cell lines	3.06E-03	HGF, PEG10, PMP22	3
Cell Morphology	morphology	morphology of normal cells	4.59E-03	CDH5, COL15A1, GH1, GJA1, HGF, PMP22, RASA1, SPARC	8
Cell Morphology	morphology	morphology of epithelial cells	1.08E-02	HGF, RASA1, SPARC	3
Cell Morphology	morphology	morphology of prostate cancer cell lines	1.15E-02	CDH1, TWIST1	2
Cell Morphology	branching	branching of tumor cell lines	1.38E-04	CDH1, HGF	2
Cell Morphology	branching	branching of cell lines	1.58E-03	CDH1, HGF, RND3	3
Cell Morphology	branching	branching of eukaryotic cells	2.62E-03	CDH1, HGF, LIFR, RND3	4
Cell Morphology	cell spreading	cell spreading	5.10E-04	FBN1, HGF, ITGAV, PITX2, PMP22, SDC1, SNAI2, SPP1, VCAM1	9
Cell Morphology	cell spreading	cell spreading of eukaryotic cells	7.87E-04	FBN1, HGF, ITGAV, PITX2, SDC1, SNAI2, SPP1, VCAM1	8
Cell Morphology	cell spreading	cell spreading of cell lines	1.30E-03	ITGAV, PITX2, SDC1, SNAI2, SPP1, VCAM1	6
Cell Morphology	cell spreading	cell spreading of epithelial cells	5.85E-03	FBN1, HGF	2
Cell Morphology	permeability	permeability of normal cells	2.72E-03	ANGPT2, CDH5, KDR	3
Cell Morphology	sprouting	sprouting of endothelial cells	2.72E-03	ANGPT2, DCN, SPP1	3
Cell Morphology	cell rounding	cell rounding	6.12E-03	HGF, ITGAV, RASA1, SPARC	4
Cell Morphology	vacuolation	vacuolation of eukaryotic cells	1.15E-02	RACGAP1, SPARC	2
Tumor Morphology	metastasis	metastasis of cells	8.88E-06	ENPP2, F3, FABP5, HGF, ITGAV, KISS1, TWIST1	7
Tumor Morphology	metastasis	metastasis of cell lines	9.07E-06	ENPP2, F3, FABP5, HGF, KISS1, TWIST1	6
Tumor Morphology	metastasis	metastasis of tumor cell lines	6.90E-05	F3, FABP5, HGF, KISS1, TWIST1	5
Tumor Morphology	metastasis	metastasis of breast cancer cell lines	3.28E-04	FABP5, HGF, TWIST1	3
Tumor Morphology	metastasis	metastasis of melanoma cell lines	8.44E-03	F3, KISS1	2
Tumor Morphology	invasion	invasion of tumor cells	9.58E-05	CDH1, DPP4, ENPP2, GH1, HGF, ITGAV	6
Tumor Morphology	invasion	invasion of cancer cells	1.11E-03	CDH1, DPP4, HGF, ITGAV	4
Tumor Morphology	invasion	invasion of melanoma cells	5.85E-03	CDH1, ITGAV	2
Tumor Morphology	development	development of papilloma	8.17E-04	HGF, SPP1	2
Tumor Morphology	development	development of squamous-cell carcinoma	2.01E-03	HGF, SKP2	2
Tumor Morphology	development	development of tumor	2.79E-03	CDH5, COL4A1, HGF, SDC1, SKP2, SNAI2, SPP1	7
Tumor Morphology	growth	growth of tumor	3.31E-03	COL4A1, GJA1, HGF, ITGAV, KDR, OLR1, SPP1	7
Tumor Morphology	adhesion	adhesion of tumor cells	8.00E-03	F3, ITGAV, VCAM1	3
Cell Death	apoptosis	apoptosis of eukaryotic cells	8.96E-06	ABCG2, AGTR1, ANGPT2, CDH1, CDH5, CGA, COL4A2, DCN, DKK1, DPP4, DSG2, DSP, F3, GH1, GHR, HGF, HSD11B2, HSPA2, HSPB1, HSPE1, IFI6, IGFBP3, ITGAV, KDR, KRT8, KRT18, MAOA, OLR1, PLK2, PROCR, PSG2, RASA1, RND3, SDC1, SERPINF1, SKP2, SNAI2, SPARC, SPP1, SRPX, TWIST1, YAP1	42
Cell Death	apoptosis	apoptosis of normal cells	3.16E-05	AGTR1, ANGPT2, CDH1, CDH5, CGA, COL4A2, DCN, DKK1, DSP, GH1, HGF, HSD11B2, HSPA2, HSPB1, HSPE1, IGFBP3, ITGAV, KDR, KRT8, MAOA, OLR1, RASA1, RND3, SERPINF1, SKP2, SPARC, SPP1, TWIST1	28
Cell Death	apoptosis	apoptosis of endothelial cells	9.85E-05	ANGPT2, CDH5, COL4A2, HGF, KDR, OLR1, SERPINF1	7

				¬© 2000-2009 Ingenuity Systems, Inc. All rights reserved.	
**Category**	**Function**	**Function Annotation**	**P-Value**	**Molecules**	**# Molecules**
Cell Death	apoptosis	apoptosis	1.58E-04	ABCG2, AGTR1, ANGPT2, CDH1, CDH5, CGA, COL4A2, DCN, DKK1, DPP4, DSG2, DSP, F3, FBN1, GH1, GHR, GJA1, HGF, HSD11B2, HSPA2, HSPB1, HSPE1, IFI6, IGFBP3, ITGAV, KDR, KRT8, KRT18, MAOA, OLR1, PLK2, PROCR, PSG2, RASA1, RND3, SDC1, SERPINF1, SKP2, SNAI2, SPARC, SPP1, SRPX, TLR3, TWIST1, YAP1	45
Cell Death	apoptosis	apoptosis of breast cancer cell lines	1.54E-03	CDH1, GH1, HGF, IGFBP3, ITGAV, KRT18, PLK2, RASA1, SDC1	9
Cell Death	apoptosis	apoptosis of cell lines	2.04E-03	ABCG2, ANGPT2, CDH1, DKK1, DSG2, F3, GH1, GHR, HGF, HSPB1, IFI6, IGFBP3, ITGAV, KRT18, MAOA, PLK2, PROCR, PSG2, RASA1, SDC1, SERPINF1, SKP2, SPARC, SPP1, SRPX, TWIST1, YAP1	27
Cell Death	apoptosis	apoptosis of tumor cell lines	3.21E-03	ABCG2, ANGPT2, CDH1, DKK1, DSG2, GH1, HGF, HSPB1, IFI6, IGFBP3, ITGAV, KRT18, MAOA, PLK2, PSG2, RASA1, SDC1, SKP2, SPARC, SPP1, SRPX, TWIST1	22
Cell Death	apoptosis	apoptosis of colon cancer cell lines	3.78E-03	DSG2, IGFBP3, ITGAV, KRT18, PSG2, SPARC, SPP1	7
Cell Death	apoptosis	apoptosis of cancer cells	5.88E-03	CDH1, DPP4, GH1, ITGAV, KDR, SPARC	6
Cell Death	apoptosis	apoptosis of tumor cells	8.63E-03	CDH1, DPP4, GH1, ITGAV, KDR, SNAI2, SPARC	7
Cell Death	apoptosis	apoptosis of endothelial cell lines	9.36E-03	HGF, PROCR, SPP1, YAP1	4
Cell Death	survival	survival of endothelial cells	1.41E-05	CDH5, HGF, ITGAV, KDR, OLR1	5
Cell Death	survival	survival of skin cell lines	8.17E-04	ITGAV, SPP1	2
Cell Death	survival	survival of eukaryotic cells	1.69E-03	ABCG2, CDH1, CDH5, DLK1, DPP4, GHR, HGF, HSPB1, IGFBP3, ITGAV, KDR, OLR1, PLK2, RASA1, SERPINB2, SERPINF1, SNAI2, SPARC, SPP1, TWIST1	20
Cell Death	survival	survival of squamous cell carcinoma cell lines	2.01E-03	CDH1, HGF	2
Cell Death	survival	survival of cancer cells	3.06E-03	ITGAV, SPARC, TWIST1	3
Cell Death	survival	survival of normal cells	6.18E-03	CDH5, DLK1, HGF, HSPB1, ITGAV, KDR, OLR1, RASA1, SERPINB2, SERPINF1, SNAI2, SPARC	12
Cell Death	survival	survival of cell lines	9.48E-03	ABCG2, CDH1, DPP4, GHR, HGF, HSPB1, IGFBP3, ITGAV, PLK2, RASA1, SERPINB2, SPP1, TWIST1	13
Cell Death	survival	survival of macrophages	1.15E-02	RASA1, SERPINB2	2
Cell Death	cell death	cell death of eukaryotic cells	2.95E-05	ABCG2, AGTR1, ANGPT2, CDH1, CDH5, CGA, COL4A2, CSH1, DCN, DKK1, DPP4, DSG2, DSP, DUSP9, F3, GH1, GHR, HGF, HSD11B2, HSPA2, HSPB1, HSPE1, IFI6, IGFBP3, ITGAV, KDR, KRT8, KRT18, MAOA, OLR1, PLK2, PMP22, PROCR, PSG2, RASA1, RND3, SDC1, SERPINB2, SERPINF1, SKP2, SNAI2, SPARC, SPP1, SRPX, TWIST1, YAP1	46
Cell Death	cell death	cell death	1.21E-04	ABCG2, AGTR1, ANGPT2, CDH1, CDH5, CGA, COL4A2, CSH1, DCN, DKK1, DPP4, DSG2, DSP, DUSP9, F3, FBN1, GH1, GHR, GJA1, GMNN, HGF, HSD11B2, HSPA2, HSPB1, HSPE1, IFI6, IGFBP3, ITGAV, KDR, KRT8, KRT18, MAOA, OLR1, PEG10, PLK2, PMP22, PROCR, PSG2, RASA1, RND3, SDC1, SERPINB2, SERPINF1, SKP2, SNAI2, SPARC, SPP1, SRPX, TLR3, TWIST1, YAP1	51
Cell Death	cell death	cell death of normal cells	1.84E-04	AGTR1, ANGPT2, CDH1, CDH5, CGA, COL4A2, CSH1, DCN, DKK1, DSP, GH1, HGF, HSD11B2, HSPA2, HSPB1, HSPE1, IGFBP3, ITGAV, KDR, KRT8, KRT18, MAOA, OLR1, RASA1, RND3, SERPINF1, SKP2, SPARC, SPP1, TWIST1	30
Cell Death	cell death	cell death of cell lines	3.11E-04	ABCG2, ANGPT2, CDH1, COL4A2, DKK1, DPP4, DSG2, DUSP9, F3, GH1, GHR, HGF, HSPB1, IFI6, IGFBP3, ITGAV, KRT8, KRT18, MAOA, PLK2, PMP22, PROCR, PSG2, RASA1, SDC1, SERPINB2, SERPINF1, SKP2, SPARC, SPP1, SRPX, TWIST1, YAP1	33
Cell Death	cell death	cell death of breast cancer cell lines	1.07E-03	ABCG2, CDH1, GH1, HGF, IGFBP3, ITGAV, KRT18, PLK2, RASA1, SDC1	10
Cell Death	cell death	cell death of endothelial cell lines	2.02E-03	COL4A2, HGF, PROCR, SPP1, YAP1	5
Cell Death	cell death	cell death of epithelial cell lines	4.11E-03	ABCG2, CDH1, HSPB1, IGFBP3, ITGAV, PMP22, SERPINF1, SPP1	8
Cell Death	cell death	cell death of tumor cell lines	5.00E-03	ABCG2, ANGPT2, CDH1, DKK1, DPP4, DSG2, DUSP9, GH1, HGF, HSPB1, IFI6, IGFBP3, ITGAV, KRT18, MAOA, PLK2, PSG2, RASA1, SDC1, SKP2, SPARC, SPP1, SRPX, TWIST1	24
Cell Death	cell death	cell death of tumor cells	5.24E-03	CDH1, DPP4, DUSP9, GH1, ITGAV, KDR, SNAI2, SPARC	8
Cell Death	cell death	cell death of kidney cell lines	5.97E-03	ABCG2, F3, HGF, HSPB1, IGFBP3, ITGAV, PMP22, SPP1	8
Cell Death	cell death	cell death of epithelial cells	6.19E-03	CDH1, DCN, HGF, KRT8, KRT18, RND3, SPARC	7
Cell Death	anoikis	anoikis of colon cancer cell lines	2.79E-03	ITGAV, PSG2	2
Cell Death	anoikis	anoikis of tumor cell lines	6.78E-03	HGF, ITGAV, PSG2	3
Cell Death	anoikis	anoikis of eukaryotic cells	8.46E-03	CDH1, HGF, ITGAV, PSG2	4
Organ Morphology	size	size of organ	9.98E-06	CGA, CYP11A1, GH1, GHR, GJA1, H19, IGFBP3, KISS1, PITX2, VCAM1	10
Organ Morphology	size	size of testis	1.16E-04	CGA, CYP11A1, GJA1, KISS1	4
Organ Morphology	size	size of pituitary gland	2.01E-03	GHR, PITX2	2
Organ Morphology	size	size of ovary	2.79E-03	GJA1, KISS1	2
Organ Morphology	mass	mass of seminal vesicle	3.28E-04	CGA, GH1, GHR	3
Organ Morphology	mass	mass of uterus	7.09E-03	CGA, KISS1	2
Organ Morphology	mass	mass of gonad	1.00E-02	CGA, GHR, KISS1	3
Organ Morphology	modification	modification of organ	3.28E-04	GHR, HSD17B2, KISS1	3
Organ Morphology	modification	delay in initiation of modification of organ	4.12E-04	GHR, HSD17B2	2
Organ Morphology	morphology	morphology of organ	3.59E-04	AGTR1, CYP11A1, GHR, HGF, LIFR, PKD2 (includes EG:5311), ROBO1	7
Organ Morphology	morphogenesis	morphogenesis of mammary alveolus	4.12E-04	CDH1, DSG2	2
Organ Morphology	morphogenesis	morphogenesis of heart	3.73E-03	COL5A1, DSP, GJA1, PKD2 (includes EG:5311)	4

¬© 2000-2009 Ingenuity Systems, Inc. All rights reserved.

Category	Function	Function Annotation	P-Value	Molecules	# Molecules
Organ Morphology	opening	opening of vagina	2.01E-03	GHR, KISS1	2
Organ Morphology	quantity	quantity of ovarian follicle	1.08E-02	ADAMTS1, CGA, GHR	3
Cellular Development	morphogenesis	morphogenesis of cells	1.53E-05	ANGPT2, CDH1, DCN, FBN1, HGF, HSPB1, ITGAV, KDR, KRT8, PITX2, PMP22, RASA1, RND3, ROBO1, SDC1, SNAI2, SPARC, SPP1, VCAM1	19
Cellular Development	developmental process	developmental process of endothelial cells	6.41E-05	CDH5, DCN, HGF, IGFBP3, KDR, OLR1, SERPINF1, VCAM1	8
Cellular Development	developmental process	developmental process of lymphatic endothelial cells	8.17E-04	HGF, KDR	2
Cellular Development	developmental process	developmental process of epithelial cells	1.38E-03	CDH1, COL4A1, DSP, GH1, GJA1, HGF, KRT8, MAFF, SNAI2, TWIST1	10
Cellular Development	developmental process	developmental process of endothelial cell lines	4.22E-03	CDH5, HGF, ITGAV, KDR, ROBO1	5
Cellular Development	developmental process	developmental process of macrophages	6.04E-03	HGF, ITGAV, LIFR, RACGAP1, TLR3	5
Cellular Development	differentiation	differentiation of cells	1.84E-04	CDH1, CDH5, CDO1, COL4A1, DCN, DKK1, DLK1, DPP4, DSP, GCM1, GH1, GHR, HGF, HSPA2, IGFBP3, ITGAV, KDR, KRT8, LAMC1, LIFR, MAFF, NID2, PITX2, PMP22, RACGAP1, RASA1, SERPINB2, SERPINF1, SPARC, SPP1, TLR3, TWIST1, TWSG1	33
Cellular Development	differentiation	differentiation of endothelial cells	6.57E-04	CDH5, DCN, IGFBP3, KDR	4
Cellular Development	differentiation	differentiation of epidermal cells	4.85E-03	CDH1, DSP, GH1, HGF, MAFF	5
Cellular Development	differentiation	differentiation of muscle cell lines	5.79E-03	CDH1, CDO1, DCN, HGF, PITX2	5
Cellular Development	differentiation	differentiation of epithelial cells	5.84E-03	CDH1, COL4A1, DSP, GH1, HGF, KRT8, MAFF	7
Cellular Development	differentiation	differentiation of central nervous system cells	8.90E-03	GCM1, GH1, LIFR, SERPINF1	4
Cellular Development	differentiation	differentiation of exocrine cells	9.90E-03	DKK1, GH1	2
Cellular Development	development	development of cells	2.58E-04	AGTR1, ANGPT2, CDH1, DCN, DKK1, FBN1, GHR, GMNN, GNGT1, HGF, HSPA2, HSPB1, ITGAV, KDR, KRT8, PGM3, PITX2, PMP22, RASA1, RND3, ROBO1, SDC1, SNAI2, SPARC, SPP1, TLR3, TWSG1, VCAM1	28
Cellular Development	tubulation	tubulation of endothelial cells	3.06E-04	HGF, IGFBP3, OLR1, SERPINF1	4
Cellular Development	tubulation	tubulation of endothelial progenitor cells	4.12E-04	IGFBP3, OLR1	2
Cellular Development	tubulation	tubulation of endothelial cell lines	9.33E-03	CDH5, HGF, ROBO1	3
Cellular Development	epithelial-mesenchymal transition	epithelial-mesenchymal transition of epithelial cells	4.22E-04	CDH1, SNAI2, TWIST1	3
Cellular Development	hypertrophy	hypertrophy of neuroendocrine cells	1.35E-03	CGA, SKP2	2
Cellular Development	tubulogenesis	tubulogenesis of cells	6.22E-03	HGF, ITGAV, KDR	3
Cellular Development	tubulogenesis	tubulogenesis of endothelial cell lines	8.44E-03	HGF, ITGAV	2
Cellular Development	tubulogenesis	tubulogenesis of endothelial cells	8.44E-03	HGF, KDR	2
Small Molecule Biochemistry	release	release of hormone	1.70E-05	CGA, CSH1, CSH2, GHR	4
Small Molecule Biochemistry	release	release of testosterone	1.35E-03	CGA, GHR	2
Small Molecule Biochemistry	release	release of progesterone	2.01E-03	CSH1, CSH2	2
Small Molecule Biochemistry	metabolism	metabolism of sulfur amino acid	2.30E-05	CDO1, GCSH, GLDC, SMS	4
Small Molecule Biochemistry	metabolism	metabolism of alpha-amino acid	5.13E-03	CDO1, GCSH, GLDC, SMS	4
Small Molecule Biochemistry	catabolism	catabolism of sulfur amino acid	3.14E-05	CDO1, GCSH, GLDC	3
Small Molecule Biochemistry	catabolism	catabolism of serine family amino acid	5.45E-05	CDO1, GCSH, GLDC	3
Small Molecule Biochemistry	catabolism	catabolism of glycine	4.12E-04	GCSH, GLDC	2
Small Molecule Biochemistry	quantity	quantity of hormone	3.36E-05	CDH5, CGA, CSH1, CSH2, GH1, GHR, KISS1	7
Small Molecule Biochemistry	quantity	quantity of beta-estradiol	2.28E-04	CDH5, CGA, GH1, KISS1	4
Small Molecule Biochemistry	quantity	quantity of progesterone	1.01E-03	CDH5, CGA, CSH1, CSH2	4
Small Molecule Biochemistry	quantity	quantity of cholesterol	3.93E-03	DLK1, GH1, HGF, LIPG, OLR1, PLA2G2A	6
Small Molecule Biochemistry	quantity	quantity of bilirubin	4.71E-03	GH1, HGF	2
Small Molecule Biochemistry	quantity	quantity of lysophosphatidic acid	9.90E-03	ENPP2, LIPG	2
Small Molecule Biochemistry	decarboxylation	decarboxylation of glycine	1.38E-04	GCSH, GLDC	2
Small Molecule Biochemistry	biosynthesis	biosynthesis of UDP-N-acetylglucosamine	4.12E-04	PGM3, UAP1	2
Small Molecule Biochemistry	incorporation	incorporation of L-amino acid	2.40E-03	GH1, IGFBP3, RASA1	3
Small Molecule Biochemistry	conversion	conversion of pregnenolone	3.70E-03	CYP11A1, STS	2
Small Molecule Biochemistry	uptake	uptake of palmitic acid	3.70E-03	GH1, OLR1	2
Small Molecule Biochemistry	uptake	uptake of fatty acid	4.25E-03	GH1, OLR1, SLC27A2	3
Small Molecule Biochemistry	production	production of aldosterone	4.71E-03	AGTR1, ANGPT2	2
Small Molecule Biochemistry	modification	modification of serine family amino acid	6.22E-03	CDK7, GCSH, GLDC	3
Small Molecule Biochemistry	accumulation	accumulation of 5-hydroxytryptamine	1.18E-02	MAOA	1
Amino Acid Metabolism	metabolism	metabolism of sulfur amino acid	2.30E-05	CDO1, GCSH, GLDC, SMS	4
Amino Acid Metabolism	metabolism	metabolism of alpha-amino acid	5.13E-03	CDO1, GCSH, GLDC, SMS	4
Amino Acid Metabolism	catabolism	catabolism of sulfur amino acid	3.14E-05	CDO1, GCSH, GLDC	3
Amino Acid Metabolism	catabolism	catabolism of serine family amino acid	5.45E-05	CDO1, GCSH, GLDC	3

				¬© 2000-2009 Ingenuity Systems, Inc. All rights reserved.	
**Category**	**Function**	**Function Annotation**	**P-Value**	**Molecules**	**# Molecules**
Amino Acid Metabolism	catabolism	catabolism of glycine	4.12E-04	GCSH, GLDC	2
Amino Acid Metabolism	decarboxylation	decarboxylation of glycine	1.38E-04	GCSH, GLDC	2
Amino Acid Metabolism	incorporation	incorporation of L-amino acid	2.40E-03	GH1, IGFBP3, RASA1	3
Amino Acid Metabolism	modification	modification of serine family amino acid	6.22E-03	CDK7, GCSH, GLDC	3
Endocrine System Development and Function	quantity	quantity of hormone	3.36E-05	CDH5, CGA, CSH1, CSH2, GH1, GHR, KISS1	7
Endocrine System Development and Function	quantity	quantity of beta-estradiol	2.28E-04	CDH5, CGA, GH1, KISS1	4
Endocrine System Development and Function	quantity	quantity of progesterone	1.01E-03	CDH5, CGA, CSH1, CSH2	4
Endocrine System Development and Function	quantity	quantity of lactotropes	2.79E-03	CGA, GH1	2
Endocrine System Development and Function	proliferation	proliferation of beta islet cells	1.95E-04	CSH1, GH1, HGF, IGFBP3	4
Endocrine System Development and Function	release	release of testosterone	1.35E-03	CGA, GHR	2
Endocrine System Development and Function	release	release of progesterone	2.01E-03	CSH1, CSH2	2
Endocrine System Development and Function	size	size of pituitary gland	2.01E-03	GHR, PITX2	2
Endocrine System Development and Function	conversion	conversion of pregnenolone	3.70E-03	CYP11A1, STS	2
Endocrine System Development and Function	production	production of aldosterone	4.71E-03	AGTR1, ANGPT2	2
Molecular Transport	quantity	quantity of hormone	3.36E-05	CDH5, CGA, CSH1, CSH2, GH1, GHR, KISS1	7
Molecular Transport	quantity	quantity of beta-estradiol	2.28E-04	CDH5, CGA, GH1, KISS1	4
Molecular Transport	quantity	quantity of progesterone	1.01E-03	CDH5, CGA, CSH1, CSH2	4
Molecular Transport	quantity	quantity of cholesterol	3.93E-03	DLK1, GH1, HGF, LIPG, OLR1, PLA2G2A	6
Molecular Transport	quantity	quantity of bilirubin	4.71E-03	GH1, HGF	2
Molecular Transport	quantity	quantity of Ca2+	7.50E-03	AGTR1, ANGPT2, DCN, GH1, HGF, ITGAV, KDR, KISS1, PKD2 (includes EG:5311)	9
Molecular Transport	quantity	quantity of lysophosphatidic acid	9.90E-03	ENPP2, LIPG	2
Molecular Transport	localization	localization of green fluorescent	1.35E-03	IGFBP3, ITGAV	2
Molecular Transport	release	release of testosterone	1.35E-03	CGA, GHR	2
Molecular Transport	release	release of progesterone	2.01E-03	CSH1, CSH2	2
Molecular Transport	uptake	uptake of palmitic acid	3.70E-03	GH1, OLR1	2
Molecular Transport	uptake	uptake of fatty acid	4.25E-03	GH1, OLR1, SLC27A2	3
Molecular Transport	accumulation	accumulation of 5-hydroxytryptamine	1.18E-02	MAOA	1
Cellular Assembly and Organization	adhesion	adhesion of cell-associated matrix	3.57E-05	COL3A1, LYVE1, NID2, PKD2 (includes EG:5311), RASA1, SGCE	6
Cellular Assembly and Organization	formation	formation of Mallory bodies	1.38E-04	KRT8, KRT18	2
Cellular Assembly and Organization	formation	formation of actin stress fibers	2.33E-03	GH1, HGF, KISS1, NET1, RND3, SPARC, VCAM1	7
Cellular Assembly and Organization	formation	formation of actin filaments	2.42E-03	GH1, HGF, KISS1, NET1, RASA1, RND3, SPARC, VCAM1	8
Cellular Assembly and Organization	formation	formation of filaments	2.51E-03	COL5A1, GH1, HGF, KISS1, KRT18, NET1, RASA1, RND3, SPARC, VCAM1	10
Cellular Assembly and Organization	formation	formation of focal adhesions	7.14E-03	FERMT2, HGF, KISS1, NET1, RND3	5
Cellular Assembly and Organization	stabilization	stabilization of keratin filaments	1.38E-04	KRT8, KRT18	2
Cellular Assembly and Organization	quantity	quantity of adherens junctions	4.12E-04	CDH1, DSP	2
Cellular Assembly and Organization	quantity	quantity of intermediate filaments	8.17E-04	DSP, KRT18	2
Cellular Assembly and Organization	quantity	quantity of intercellular junctions	2.40E-03	CDH1, DSP, GJA1	3
Cellular Assembly and Organization	quantity	quantity of focal adhesions	4.25E-03	CDH5, SPARC, SPP1	3

¬© 2000-2009 Ingenuity Systems, Inc. All rights reserved.

Category	Function	Function Annotation	P-Value	Molecules	# Molecules
Cellular Assembly and Organization	quantity	quantity of filaments	5.30E-03	COL5A1, DSP, KRT18, RND3, SPP1	5
Cellular Assembly and Organization	morphology	morphology of filaments	4.22E-04	COL5A1, EPS8, LUM	3
Cellular Assembly and Organization	morphology	morphology of fibrils	8.17E-04	COL5A1, LUM	2
Cellular Assembly and Organization	rearrangement	rearrangement of actin stress fibers	2.01E-03	CDH5, VCAM1	2
Cellular Assembly and Organization	rearrangement	rearrangement of cytoskeleton	3.73E-03	CDH1, GH1, ITGAV, SPP1	4
Cellular Assembly and Organization	organization	organization of collagen fibrils	2.10E-03	COL3A1, COL5A1, COL5A2	3
Cellular Assembly and Organization	organization	organization of filaments	4.24E-03	COL3A1, COL5A1, COL5A2, GHR, KRT18, TCHH	6
Cellular Assembly and Organization	organization	organization of intermediate filaments	7.09E-03	KRT18, TCHH	2
Cellular Assembly and Organization	disruption	disruption of adherens junctions	2.79E-03	CDH1, HGF	2
Cellular Assembly and Organization	disruption	disruption of microtubules	9.90E-03	DUSP9, KRT18	2
Cellular Assembly and Organization	myelination	myelination of axons	7.09E-03	LAMC1, PMP22	2
Cellular Assembly and Organization	accumulation	accumulation of lipid droplets	9.90E-03	DSP, HGF	2
Cellular Assembly and Organization	development	development of neurites	1.14E-02	GJA1, KDR, LAMB1, LAMC1, LIFR, PMP22, ROBO1	7
Cellular Function and Maintenance	organization	organization of eukaryotic cells	3.94E-05	CDH1, CDH5, DSG2, KDR	4
Cellular Function and Maintenance	organization	organization of normal cells	8.06E-04	CDH5, DSG2, KDR	3
Cellular Function and Maintenance	organization	organization of endothelial cells	2.01E-03	CDH5, KDR	2
Cellular Function and Maintenance	organization	organization of collagen fibrils	2.10E-03	COL3A1, COL5A1, COL5A2	3
Cellular Function and Maintenance	organization	organization of filaments	4.24E-03	COL3A1, COL5A1, COL5A2, GHR, KRT18, TCHH	6
Cellular Function and Maintenance	organization	organization of intermediate filaments	7.09E-03	KRT18, TCHH	2
Tissue Morphology	size	size of tissue	4.90E-05	GH1, GHR, H19, PITX2, SPARC, SPP1	6
Tissue Morphology	size	size of fat pad	5.45E-05	GH1, GHR, SPARC	3
Tissue Morphology	size	size of connective tissue	7.80E-05	GH1, GHR, PITX2, SPARC	4
Tissue Morphology	structural integrity	structural integrity of Reichert's membrane	1.38E-04	COL4A1, COL4A2	2
Tissue Morphology	structural integrity	structural integrity of basement membrane	4.12E-04	COL4A1, COL4A2	2
Tissue Morphology	volume	volume of tissue	3.26E-04	AGTR1, CGA, GH1, LIFR, SPP1	5
Tissue Morphology	volume	volume of bone	1.47E-03	AGTR1, CGA, LIFR, SPP1	4
Tissue Morphology	quantity	quantity of osteoblasts	2.72E-03	DCN, DKK1, SPARC	3
Tissue Morphology	quantity	quantity of lactotropes	2.79E-03	CGA, GH1	2
Tissue Morphology	quantity	quantity of connective tissue cells	3.09E-03	DCN, DKK1, GH1, IGFBP3, SPARC, SPP1	6
Tissue Morphology	quantity	quantity of cells	5.00E-03	ABCG2, ADAMTS1, AGTR1, ANGPT2, CGA, DCN, DKK1, EBI3, GH1, GHR, GJA1, HGF, IGFBP3, KDR, KRT8, LAMC1, LIFR, LIPG, PITX2, SKP2, SPARC, SPP1, TLR3, VCAM1	24
Tissue Morphology	quantity	quantity of osteocytes	6.48E-03	DCN, DKK1, SPARC, SPP1	4
Tissue Morphology	density	density of microvessel	2.79E-03	HGF, KDR	2
Tissue Morphology	fusion	fusion of allantois	4.71E-03	VCAM1, YAP1	2
Tissue Morphology	fusion	fusion of chorion	4.71E-03	VCAM1, YAP1	2
Tissue Morphology	morphology	morphology of tissue	1.09E-02	CDH1, GJA1, HAPLN1, HGF, LIFR	5
Tissue Morphology	thickness	thickness of blood vessel	1.15E-02	AGTR1, SPP1	2

				¬© 2000-2009 Ingenuity Systems, Inc. All rights reserved.	
**Category**	**Function**	**Function Annotation**	**P-Value**	**Molecules**	**# Molecules**
Connective Tissue Development and Function	size	size of fat pad	5.45E-05	GH1, GHR, SPARC	3
Connective Tissue Development and Function	size	size of connective tissue	7.80E-05	GH1, GHR, PITX2, SPARC	4
Connective Tissue Development and Function	formation	formation of bone	5.47E-05	DCN, DLK1, GH1, HSD11B2, IGFBP3, SPARC, SPP1	7
Connective Tissue Development and Function	formation	formation of adipocytes	3.70E-03	DLK1, SPARC	2
Connective Tissue Development and Function	formation	formation of trabecular bone	4.71E-03	IGFBP3, SPP1	2
Connective Tissue Development and Function	binding	binding of osteoblasts	8.64E-05	DKK1, IGFBP3, VCAM1	3
Connective Tissue Development and Function	binding	binding of fibroblasts	8.06E-04	IGFBP3, ITGAV, SPARC	3
Connective Tissue Development and Function	cell movement	cell movement of osteoclasts	2.48E-04	HGF, ITGAV, SPP1	3
Connective Tissue Development and Function	bone mineral density	bone mineral density of bone	8.19E-04	CGA, DKK1, GH1, SPP1	4
Connective Tissue Development and Function	volume	volume of bone	1.47E-03	AGTR1, CGA, LIFR, SPP1	4
Connective Tissue Development and Function	proliferation	proliferation of stromal cells	2.10E-03	CGA, HGF, IGFBP3	3
Connective Tissue Development and Function	proliferation	proliferation of osteoclasts	8.44E-03	HGF, ITGAV	2
Connective Tissue Development and Function	proliferation	proliferation of hepatic stellate cells	1.15E-02	ITGAV, SPP1	2
Connective Tissue Development and Function	quantity	quantity of osteoblasts	2.72E-03	DCN, DKK1, SPARC	3
Connective Tissue Development and Function	quantity	quantity of connective tissue cells	3.09E-03	DCN, DKK1, GH1, IGFBP3, SPARC, SPP1	6
Connective Tissue Development and Function	quantity	quantity of osteocytes	6.48E-03	DCN, DKK1, SPARC, SPP1	4
Connective Tissue Development and Function	activation	activation of fibroblast cell lines	3.70E-03	KRT8, KRT18	2
Connective Tissue Development and Function	deposition	deposition of connective tissue	4.71E-03	GJA1, SPARC	2
Skeletal and Muscular System Development and Function	formation	formation of bone	5.47E-05	DCN, DLK1, GH1, HSD11B2, IGFBP3, SPARC, SPP1	7
Skeletal and Muscular System Development and Function	formation	formation of trabecular bone	4.71E-03	IGFBP3, SPP1	2
Skeletal and Muscular System Development and Function	binding	binding of osteoblasts	8.64E-05	DKK1, IGFBP3, VCAM1	3
Skeletal and Muscular System Development and Function	cell movement	cell movement of osteoclasts	2.48E-04	HGF, ITGAV, SPP1	3
Skeletal and Muscular System Development and Function	bone mineral density	bone mineral density of bone	8.19E-04	CGA, DKK1, GH1, SPP1	4
Skeletal and Muscular System Development and Function	development	development of skeleton	1.02E-03	COL5A2, DLK1, FBN1, GHR, GJA1, HSD11B2, PLK2, SPARC, SPP1, TWIST1, TWSG1	11
Skeletal and Muscular System Development and Function	volume	volume of bone	1.47E-03	AGTR1, CGA, LIFR, SPP1	4
Skeletal and Muscular System Development and Function	quantity	quantity of osteoblasts	2.72E-03	DCN, DKK1, SPARC	3
Skeletal and Muscular System Development and Function	quantity	quantity of osteocytes	6.48E-03	DCN, DKK1, SPARC, SPP1	4
Skeletal and Muscular System Development and Function	differentiation	differentiation of muscle cell lines	5.79E-03	CDH1, CDO1, DCN, HGF, PITX2	5
Skeletal and Muscular System Development and Function	proliferation	proliferation of smooth muscle cells	7.68E-03	F3, HGF, IGFBP3, SKP2, SPARC, SPP1	6
Skeletal and Muscular System Development and Function	proliferation	proliferation of osteoclasts	8.44E-03	HGF, ITGAV	2
Cardiovascular Disease	apoptosis	apoptosis of endothelial cells	9.85E-05	ANGPT2, CDH5, COL4A2, HGF, KDR, OLR1, SERPINF1	7

				¬© 2000-2009 Ingenuity Systems, Inc. All rights reserved.	
**Category**	**Function**	**Function Annotation**	**P-Value**	**Molecules**	**# Molecules**
Cardiovascular Disease	apoptosis	apoptosis of endothelial cell lines	9.36E-03	HGF, PROCR, SPP1, YAP1	4
Cardiovascular Disease	cardiac fibrosis	cardiac fibrosis	1.22E-03	AGTR1, ANGPT2, F3, PLA2G2A	4
Cardiovascular Disease	vascular lesion	vascular lesion	1.37E-03	ANGPT2, F3, FBN1, PLA2G2A, SPP1, VCAM1	6
Cardiovascular Disease	Budd-Chiari syndrome	Budd-Chiari syndrome	1.83E-03	ANGPT2, COL4A1, F3	3
Cardiovascular Disease	cell death	cell death of endothelial cell lines	2.02E-03	COL4A2, HGF, PROCR, SPP1, YAP1	5
Cardiovascular Disease	chronic heart failure	chronic heart failure	2.10E-03	AGTR1, GH1, GHR	3
Cardiovascular Disease	arrhythmogenic right ventricular dysplasia	arrhythmogenic right ventricular dysplasia	3.70E-03	DSG2, DSP	2
Cardiovascular Disease	cardiovascular disorder	cardiovascular disorder	8.16E-03	AGPAT5, AGTR1, ANGPT2, COL15A1, COL3A1, COL4A1, COL4A2, COL5A1, COL5A2, COLEC12, DLK1, DPP4, DSG2, DSP, ENPEP, F3, GABRE, GH1, GHR, GJA1, GLDC, GRHL1, HGF, HSD11B2, ITGAV, KDR, LGMN, LIFR, LIN28B, LIPG, LRP11, MAOA, MAP4K3, MATN2, OLR1, PLA2G2A, PLSCR4, PROCR, RASA1, ROBO1, SPARC, SPP1, VCAM1, YAP1	44
Cardiovascular Disease	cardiovascular disorder	cardiovascular disorder of left ventricle	9.90E-03	AGTR1, ANGPT2	2
Cardiovascular Disease	aneurysm	aneurysm	9.90E-03	ANGPT2, FBN1	2
Cardiovascular Disease	arteriosclerosis	arteriosclerosis	9.90E-03	SPARC, SPP1	2
Embryonic Development	structural integrity	structural integrity of Reichert's membrane	1.38E-04	COL4A1, COL4A2	2
Embryonic Development	fusion	fusion of allantois	4.71E-03	VCAM1, YAP1	2
Embryonic Development	fusion	fusion of chorion	4.71E-03	VCAM1, YAP1	2
Embryonic Development	tubulogenesis	tubulogenesis of cells	6.22E-03	HGF, ITGAV, KDR	3
Embryonic Development	development	development of extraembryonic tissue	6.85E-03	ADAMTS1, ENPP2, HGF, YAP1	4
Embryonic Development	development	development of fetal membranes	7.37E-03	ADAMTS1, ENPP2, HGF	3
Embryonic Development	adhesion	adhesion of embryonic cells	8.44E-03	CDH1, ITGAV	2
Hair and Skin Development and Function	fragility	fragility of skin	1.38E-04	DCN, LUM	2
Hair and Skin Development and Function	stabilization	stabilization of keratin filaments	1.38E-04	KRT8, KRT18	2
Hair and Skin Development and Function	development	development of skin	1.61E-04	COL3A1, COL5A1, COL5A2, HGF, KDR	5
Hair and Skin Development and Function	tensile strength	tensile strength of skin	4.12E-04	COL5A1, DCN	2
Hair and Skin Development and Function	survival	survival of skin cell lines	8.17E-04	ITGAV, SPP1	2
Hair and Skin Development and Function	differentiation	differentiation of epidermal cells	4.85E-03	CDH1, DSP, GH1, HGF, MAFF	5
Hair and Skin Development and Function	angiogenesis	angiogenesis of skin	7.09E-03	HGF, KDR	2
Hepatic System Development and Function	formation	formation of Mallory bodies	1.38E-04	KRT8, KRT18	2
Hepatic System Development and Function	proliferation	proliferation of hepatic stellate cells	1.15E-02	ITGAV, SPP1	2
Post-Translational Modification	decarboxylation	decarboxylation of glycine	1.38E-04	GCSH, GLDC	2
Post-Translational Modification	modification	modification of serine family amino acid	6.22E-03	CDK7, GCSH, GLDC	3
Organ Development	development	development of skin	1.61E-04	COL3A1, COL5A1, COL5A2, HGF, KDR	5
Organ Development	development	development of organ	4.17E-04	ADAMTS1, AGTR1, CGA, COL3A1, COL5A1, COL5A2, CYP11A1, DKK1, DSP, FBN1, GJA1, HGF, KDR, KISS1, LIFR, PEG10, PITX2, PKD2 (includes EG:5311), SNAI2, SPARC, TWSG1, VCAM1	22
Organ Development	development	development of mammary alveolus	1.15E-03	CDH1, DSG2, GH1	3
Organ Development	development	development of uterus	3.70E-03	ADAMTS1, KISS1	2
Organ Development	development	development of heart	4.71E-03	COL3A1, COL5A1, DSP, FBN1, GJA1, PITX2, PKD2 (includes EG:5311), VCAM1	8
Organ Development	development	development of ovarian follicle	4.83E-03	CGA, GJA1, KDR, KISS1	4
Organ Development	development	development of gonad	6.48E-03	ADAMTS1, CGA, KDR, SNAI2	4
Organ Development	organogenesis	organogenesis	2.41E-04	ADAMTS1, CGA, COL3A1, COL5A1, COL5A2, DCN, DSP, FBN1, GJA1, GMNN, KDR, LAMB1, PITX2, PKD2 (includes EG:5311), SERPINF1, SNAI2, TWSG1, VCAM1	18
Organ Development	morphogenesis	morphogenesis of mammary alveolus	4.12E-04	CDH1, DSG2	2
Organ Development	morphogenesis	morphogenesis of heart	3.73E-03	COL5A1, DSP, GJA1, PKD2 (includes EG:5311)	4
Organ Development	neovascularization	neovascularization of retina	1.58E-03	IGFBP3, KDR, SERPINF1	3
Organ Development	vascularization	vascularization of cornea	3.06E-03	ADAMTS1, HGF, SERPINF1	3
Organ Development	angiogenesis	angiogenesis of cornea	4.70E-03	CDH5, HGF, VCAM1	3
Organ Development	angiogenesis	angiogenesis of skin	7.09E-03	HGF, KDR	2
Organ Development	formation	formation of organ	5.79E-03	COL5A1, COL5A2, ITGAV, SERPINF1, TWSG1	5

¬© 2000-2009 Ingenuity Systems, Inc. All rights reserved.

Category	Function	Function Annotation	P-Value	Molecules	# Molecules
Organismal Injury and Abnormalities	fibrosis	fibrosis	1.73E-04	AGTR1, ANGPT2, DSP, HGF, IFI6, KDR, KRT8, LYVE1, PLA2G2A, SKP2, SPP1	11
Organismal Injury and Abnormalities	fibrosis	fibrosis of kidney	1.15E-03	AGTR1, HGF, SKP2	3
Organismal Injury and Abnormalities	quantity	quantity of nodule	3.28E-04	GJA1, IGFBP3, SPARC	3
Organismal Injury and Abnormalities	nodule	nodule	9.10E-04	GJA1, IGFBP3, SPARC, SPP1	4
Organismal Injury and Abnormalities	edema	edema	1.02E-02	ANGPT2, F3, HGF, ITGAV, KDR, PROCR	6
Endocrine System Disorders	diabetic retinopathy	diabetic retinopathy	1.82E-04	HGF, SERPINF1, VCAM1	3
Endocrine System Disorders	hypertrophy	hypertrophy of neuroendocrine cells	1.35E-03	CGA, SKP2	2
Endocrine System Disorders	migration	migration of thyroid tumor cell lines	3.70E-03	HGF, KISS1	2
Endocrine System Disorders	diabetic nephropathy	diabetic nephropathy	7.09E-03	AGTR1, SPP1	2
Endocrine System Disorders	developmental process	developmental process of thyroid tumor cell lines	9.90E-03	HGF, KISS1	2
Metabolic Disease	diabetic retinopathy	diabetic retinopathy	1.82E-04	HGF, SERPINF1, VCAM1	3
Metabolic Disease	glycine encephalopathy	glycine encephalopathy	4.12E-04	GCSH, GLDC	2
Metabolic Disease	diabetic nephropathy	diabetic nephropathy	7.09E-03	AGTR1, SPP1	2
Metabolic Disease	hypoinsulinemia	hypoinsulinemia of mice	1.15E-02	GH1, SKP2	2
Neurological Disease	diabetic retinopathy	diabetic retinopathy	1.82E-04	HGF, SERPINF1, VCAM1	3
Neurological Disease	chronic fatigue syndrome	chronic fatigue syndrome	1.22E-03	ENPP2, SERPINF1, SPP1, TLR3	4
Neurological Disease	cell movement	cell movement of glioma cells	2.79E-03	ITGAV, SPP1	2
Neurological Disease	migration	migration of glioma cells	5.85E-03	HGF, ITGAV	2
Ophthalmic Disease	diabetic retinopathy	diabetic retinopathy	1.82E-04	HGF, SERPINF1, VCAM1	3
Ophthalmic Disease	age-related macular degeneration-wet form	age-related macular degeneration-wet form	1.35E-03	HTRA1, KDR	2
Ophthalmic Disease	opthalmic disorder	opthalmic disorder	1.76E-03	DCN, DLK1, EFEMP1, FBN1, HGF, HTRA1, KDR, MAOA, PITX2, PVRL3, SERPINF1, VCAM1	12
Renal and Urological Disease	disease	disease of kidney	2.02E-04	AGTR1, H19, HGF, LAMC1, SKP2, TLR3	6
Renal and Urological Disease	end stage renal disease	end stage renal disease	3.06E-04	GH1, GHR, HGF, SKP2	4
Renal and Urological Disease	glomerulosclerosis	glomerulosclerosis of mice	8.06E-04	AGTR1, GH1, GHR	3
Renal and Urological Disease	fibrosis	fibrosis of kidney	1.15E-03	AGTR1, HGF, SKP2	3
Renal and Urological Disease	cell death	cell death of kidney cell lines	5.97E-03	ABCG2, F3, HGF, HSPB1, IGFBP3, ITGAV, PMP22, SPP1	8
Renal and Urological Disease	diabetic nephropathy	diabetic nephropathy	7.09E-03	AGTR1, SPP1	2
Renal and Urological Disease	experimental crescentic glomerulonephritis	experimental crescentic glomerulonephritis	7.09E-03	SPP1, VCAM1	2
Renal and Urological Disease	renal and urological disorder	renal and urological disorder	9.71E-03	AGTR1, GH1, GHR, HGF, HSD11B2, KDR, PKD2 (includes EG:5311), SKP2, SPP1, TLR3, VCAM1	11
Drug Metabolism	quantity	quantity of beta-estradiol	2.28E-04	CDH5, CGA, GH1, KISS1	4
Drug Metabolism	quantity	quantity of progesterone	1.01E-03	CDH5, CGA, CSH1, CSH2	4
Drug Metabolism	release	release of testosterone	1.35E-03	CGA, GHR	2
Drug Metabolism	release	release of progesterone	2.01E-03	CSH1, CSH2	2
Lipid Metabolism	quantity	quantity of beta-estradiol	2.28E-04	CDH5, CGA, GH1, KISS1	4
Lipid Metabolism	quantity	quantity of progesterone	1.01E-03	CDH5, CGA, CSH1, CSH2	4
Lipid Metabolism	quantity	quantity of cholesterol	3.93E-03	DLK1, GH1, HGF, LIPG, OLR1, PLA2G2A	6
Lipid Metabolism	quantity	quantity of lysophosphatidic acid	9.90E-03	ENPP2, LIPG	2
Lipid Metabolism	release	release of testosterone	1.35E-03	CGA, GHR	2
Lipid Metabolism	release	release of progesterone	2.01E-03	CSH1, CSH2	2
Lipid Metabolism	conversion	conversion of pregnenolone	3.70E-03	CYP11A1, STS	2
Lipid Metabolism	uptake	uptake of palmitic acid	3.70E-03	GH1, OLR1	2
Lipid Metabolism	uptake	uptake of fatty acid	4.25E-03	GH1, OLR1, SLC27A2	3
Lipid Metabolism	production	production of aldosterone	4.71E-03	AGTR1, ANGPT2	2
Carbohydrate Metabolism	biosynthesis	biosynthesis of UDP-N-acetylglucosamine	4.12E-04	PGM3, UAP1	2
Cell Cycle	replication	replication of B lymphocytes	4.12E-04	CSH1, CSH2	2
Cell Cycle	interphase	interphase of tumor cell lines	1.10E-03	CDH1, DCN, DPP4, DUSP9, GJA1, GMNN, HGF, ITGAV, PITX2, SKP2	10
Cell Cycle	interphase	interphase of cell lines	1.76E-03	CDH1, CKS2, DCN, DPP4, DUSP9, GHR, GJA1, GMNN, HGF, ITGAV, PITX2, SKP2	12
Cell Cycle	interphase	interphase of eukaryotic cells	2.88E-03	CDH1, CKS2, DCN, DPP4, DUSP9, GHR, GJA1, GMNN, HGF, ITGAV, PITX2, PLK2, SKP2	13
Cell Cycle	interphase	arrest in interphase of cell lines	3.28E-03	CKS2, DCN, DPP4, DUSP9, GHR, HGF, ITGAV, PITX2, SKP2	9

				¬© 2000-2009 Ingenuity Systems, Inc. All rights reserved.	
**Category**	**Function**	**Function Annotation**	**P-Value**	**Molecules**	**# Molecules**
Cell Cycle	interphase	arrest in interphase of tumor cell lines	5.02E-03	DCN, DPP4, DUSP9, HGF, ITGAV, PITX2, SKP2	7
Cell Cycle	cell division process	cell division process of tumor cell lines	1.20E-03	CDH1, CKS2, DCN, DPP4, DUSP9, GH1, GJA1, GMNN, GNAI1, HGF, IGFBP3, ITGAV, PITX2, SKP2	14
Cell Cycle	cell division process	cell division process of carcinoma cells	2.01E-03	DCN, GH1	2
Cell Cycle	cell division process	cell division process of cell lines	2.83E-03	CDH1, CKS2, DCN, DPP4, DUSP9, GH1, GHR, GJA1, GMNN, GNAI1, HGF, IGFBP3, ITGAV, PITX2, SKP2, SPARC, SPP1	17
Cell Cycle	cell division process	cell division process of cells	3.65E-03	ANLN, CDH1, CKS2, DCN, DPP4, DUSP9, GH1, GHR, GJA1, GMNN, GNAI1, H19, HGF, HSPA2, IGFBP3, ITGAV, KDR, PITX2, PKD2 (includes EG:5311), PLK2, RACGAP1, SKP2, SPARC, SPP1	24
Cell Cycle	cell division process	cell division process of eukaryotic cells	3.84E-03	CDH1, CKS2, DCN, DPP4, DUSP9, GH1, GHR, GJA1, GMNN, GNAI1, HGF, HSPA2, IGFBP3, ITGAV, KDR, PITX2, PLK2, SKP2, SPARC, SPP1	20
Cell Cycle	cell division process	arrest in cell division process of tumor cell lines	7.46E-03	CKS2, DCN, DPP4, DUSP9, HGF, ITGAV, PITX2, SKP2	8
Cell Cycle	cell division process	cell division process of cancer cells	9.33E-03	DCN, DPP4, GH1	3
Cell Cycle	G1 phase	G1 phase of ovarian cancer cell lines	1.35E-03	ITGAV, SKP2	2
Cell Cycle	cell stage	cell stage of tumor cell lines	2.24E-03	CDH1, DCN, DPP4, DUSP9, GJA1, GMNN, GNAI1, HGF, ITGAV, PITX2, SKP2	11
Cell Cycle	cell stage	cell stage of cells	2.77E-03	ANLN, CDH1, CKS2, DCN, DPP4, DUSP9, GHR, GJA1, GMNN, GNAI1, H19, HGF, HSPA2, ITGAV, KDR, PITX2, PLK2, RACGAP1, SKP2	19
Cell Cycle	cell stage	cell stage of cell lines	3.72E-03	CDH1, CKS2, DCN, DPP4, DUSP9, GHR, GJA1, GMNN, GNAI1, HGF, ITGAV, PITX2, SKP2	13
Cell Cycle	cell stage	cell stage of eukaryotic cells	5.63E-03	CDH1, CKS2, DCN, DPP4, DUSP9, GHR, GJA1, GMNN, GNAI1, HGF, HSPA2, ITGAV, PITX2, PLK2, SKP2	15
Cell Cycle	mitogenesis	mitogenesis of bone cancer cell lines	2.79E-03	GH1, IGFBP3	2
Cell Cycle	mitogenesis	mitogenesis of eukaryotic cells	6.97E-03	GH1, HGF, IGFBP3, KDR, SPARC, SPP1	6
Cell Cycle	mitogenesis	mitogenesis of cell lines	7.14E-03	GH1, HGF, IGFBP3, SPARC, SPP1	5
Hematological System Development and Function	replication	replication of B lymphocytes	4.12E-04	CSH1, CSH2	2
Hematological System Development and Function	infiltration	infiltration of macrophages	5.05E-04	ANGPT2, HGF, KDR, SPP1, TLR3	5
Hematological System Development and Function	accumulation	accumulation of monocytes	2.01E-03	RASA1, VCAM1	2
Hematological System Development and Function	survival	survival of macrophages	1.15E-02	RASA1, SERPINB2	2
Humoral Immune Response	replication	replication of B lymphocytes	4.12E-04	CSH1, CSH2	2
Humoral Immune Response	immune response	immune response of organism	6.29E-03	ASS1, COL3A1, COLEC12, EBI3, GH1, KRT8, LYVE1, PSG3, TLR3	9
Immune Cell Trafficking	emigration	emigration of Langerhans cells	4.12E-04	ITGAV, SPP1	2
Immune Cell Trafficking	emigration	emigration of leukocytes	5.68E-03	ITGAV, SPP1, VCAM1	3
Immune Cell Trafficking	infiltration	infiltration of macrophages	5.05E-04	ANGPT2, HGF, KDR, SPP1, TLR3	5
Immune Cell Trafficking	migration	migration of monocytes	1.23E-03	COL4A1, HGF, ITGAV, SPP1, VCAM1	5
Immune Cell Trafficking	accumulation	accumulation of monocytes	2.01E-03	RASA1, VCAM1	2
Nucleic Acid Metabolism	biosynthesis	biosynthesis of UDP-N-acetylglucosamine	4.12E-04	PGM3, UAP1	2
Skeletal and Muscular Disorders	Ehlers-Danlos syndrome, type I	Ehlers-Danlos syndrome, type I	4.12E-04	COL5A1, COL5A2	2
Skeletal and Muscular Disorders	Ehlers-Danlos syndrome	Ehlers-Danlos syndrome	4.22E-04	COL3A1, COL5A1, COL5A2	3
Skeletal and Muscular Disorders	chemotaxis	chemotaxis of rhabdomyosarcoma cell lines	1.35E-03	HGF, LIFR	2
Skeletal and Muscular Disorders	mitogenesis	mitogenesis of bone cancer cell lines	2.79E-03	GH1, IGFBP3	2
Skeletal and Muscular Disorders	leiomyosarcoma	leiomyosarcoma	8.00E-03	CKS2, KDR, SPP1	3
Skeletal and Muscular Disorders	myosarcoma	myosarcoma	8.46E-03	CKS2, HGF, KDR, SPP1	4
Skeletal and Muscular Disorders	bone tumor	bone tumor	1.17E-02	CDK7, ITGAV, KDR, SPARC, SPP1	5
Skeletal and Muscular Disorders	Ehlers-Danlos Syndrome Type IV	Ehlers-Danlos Syndrome Type IV	1.18E-02	COL3A1	1
Skeletal and Muscular Disorders	Ehlers-Danlos syndrome, type II	Ehlers-Danlos syndrome, type II	1.18E-02	COL5A1	1
Antigen Presentation	infiltration	infiltration of macrophages	5.05E-04	ANGPT2, HGF, KDR, SPP1, TLR3	5
Antigen Presentation	immune response	immune response of organism	6.29E-03	ASS1, COL3A1, COLEC12, EBI3, GH1, KRT8, LYVE1, PSG3, TLR3	9
Inflammatory Response	infiltration	infiltration of macrophages	5.05E-04	ANGPT2, HGF, KDR, SPP1, TLR3	5
Inflammatory Response	immune response	immune response of organism	6.29E-03	ASS1, COL3A1, COLEC12, EBI3, GH1, KRT8, LYVE1, PSG3, TLR3	9
Developmental Disorder	developmental disorder	developmental disorder	5.37E-04	AGTR1, ANGPT2, CGA, CYP11A1, DLK1, DPP4, DSP, FBN1, GABRE, GCM1, GH1, GHR, GJA1, GPX3, H19, HSD11B2, KRT8, LAMC1, PITX2, PVRL3, SKP2, SNAI2, TWSG1	23
Developmental Disorder	developmental disorder	developmental disorder of rodents	1.58E-03	CYP11A1, DLK1, GCM1, GH1, H19, PVRL3, SNAI2, TWSG1	8
Developmental Disorder	developmental disorder	developmental disorder of mice	5.67E-03	CYP11A1, DLK1, GCM1, H19, PVRL3, SNAI2, TWSG1	7
Developmental Disorder	Marfan's syndrome	Marfan's syndrome	8.17E-04	AGTR1, FBN1	2
Developmental Disorder	gonadal dysgenesis	gonadal dysgenesis	2.79E-03	GH1, GHR	2

Highly Expressed in CVS Versus MBC

Table S1H

				¬© 2000-2009 Ingenuity Systems, Inc. All rights reserved.	
**Category**	**Function**	**Function Annotation**	**P-Value**	**Molecules**	**# Molecules**
Developmental Disorder	growth failure	growth failure	2.79E-03	GH1, GHR	2
Developmental Disorder	Rieger syndrome	Rieger syndrome	1.18E-02	PITX2	1
DNA Replication, Recombination, and Repair	synthesis	synthesis of DNA	5.58E-04	AGTR1, ANGPT2, EFEMP1, GH1, GJA1, GNAI1, HGF, IGFBP3, KDR, SKP2, SPARC, SPP1, TFPI2	13
Immunological Disease	proliferation	proliferation of lymphoma cell lines	6.95E-04	CSH1, CSH2, GH1, GH2, IGFBP3	5
Immunological Disease	adhesion	adhesion of lymphoma cell lines	8.00E-03	HGF, SDC1, VCAM1	3
Immunological Disease	leukopenia	leukopenia of mice	9.90E-03	PGM3, TWSG1	2
Hepatic System Disease	invasion	invasion of hepatoma cell lines	8.06E-04	HGF, SPP1, TFPI2	3
Visual System Development and Function	opacity	opacity of cornea	8.17E-04	LUM, PITX2	2
Visual System Development and Function	neovascularization	neovascularization of retina	1.58E-03	IGFBP3, KDR, SERPINF1	3
Visual System Development and Function	vascularization	vascularization of cornea	3.06E-03	ADAMTS1, HGF, SERPINF1	3
Visual System Development and Function	angiogenesis	angiogenesis of cornea	4.70E-03	CDH5, HGF, VCAM1	3
Hematological Disease	multiple myeloma	multiple myeloma	1.00E-03	CDK7, DKK1, GH1, GHR, ITGAV, KDR, SDC1	7
Hematological Disease	Budd-Chiari syndrome	Budd-Chiari syndrome	1.83E-03	ANGPT2, COL4A1, F3	3
Hematological Disease	adhesion	adhesion of leukemia cell lines	3.73E-03	CDH1, SERPINB2, SPP1, VCAM1	4
Hematological Disease	acute lymphoblastic leukemia	acute lymphoblastic leukemia	5.13E-03	DPP4, F3, GH1, GHR	4
Hematological Disease	hematologic cancer	hematologic cancer	9.83E-03	CDK7, DKK1, DPP4, F3, GH1, GHR, ITGAV, KDR, SDC1, SNAI2	10
Hematological Disease	leukopenia	leukopenia of mice	9.90E-03	PGM3, TWSG1	2
Free Radical Scavenging	generation	generation of reactive oxygen species	1.31E-03	HGF, HSPB1, OLR1, SERPINF1, VCAM1	5
Protein Trafficking	localization	localization of green fluorescent	1.35E-03	IGFBP3, ITGAV	2
Cellular Compromise	disruption	disruption of adherens junctions	2.79E-03	CDH1, HGF	2
Cellular Compromise	disruption	disruption of microtubules	9.90E-03	DUSP9, KRT18	2
Cellular Compromise	dysfunction	dysfunction of cells	4.25E-03	AGTR1, ANGPT2, MAFF	3
Cellular Compromise	adhesion	adhesion of tumor cells	8.00E-03	F3, ITGAV, VCAM1	3
Cellular Compromise	endoplasmic reticulum stress response	endoplasmic reticulum stress response of cells	9.33E-03	HSPA2, HSPB1, HSPE1	3
Nervous System Development and Function	quantity	quantity of lactotropes	2.79E-03	CGA, GH1	2
Nervous System Development and Function	myelination	myelination of axons	7.09E-03	LAMC1, PMP22	2
Nervous System Development and Function	differentiation	differentiation of central nervous system cells	8.90E-03	GCM1, GH1, LIFR, SERPINF1	4
Nervous System Development and Function	development	development of neurites	1.14E-02	GJA1, KDR, LAMB1, LAMC1, LIFR, PMP22, ROBO1	7
Renal and Urological System Development and Function	proliferation	proliferation of tubular cells	2.79E-03	HGF, SKP2	2
Renal and Urological System Development and Function	proliferation	proliferation of kidney cells	8.65E-03	HGF, SKP2, SPARC	3
Renal and Urological System Development and Function	morphology	morphology of kidney cell lines	3.06E-03	HGF, PEG10, PMP22	3
Renal and Urological System Development and Function	activation	activation of kidney cell lines	3.70E-03	HGF, TLR3	2
Renal and Urological System Development and Function	adhesion	adhesion of kidney cell lines	1.00E-02	CDH1, HGF, ITGAV	3
Nutritional Disease	nutritional disorder	nutritional disorder of mice	4.59E-03	CRY1, DLK1, DPP4, GH1, IGFBP3, SDC1, SPP1, TWIST1	8
Nutritional Disease	nutritional disorder	nutritional disorder	5.03E-03	AGTR1, CRY1, DLK1, DPP4, GABRE, GH1, IGFBP3, MAOA, SDC1, SPP1, TWIST1	11
Inflammatory Disease	acute respiratory distress syndrome	acute respiratory distress syndrome	5.18E-03	F3, IGFBP3, PLA2G2A	3
Inflammatory Disease	experimental crescentic glomerulonephritis	experimental crescentic glomerulonephritis	7.09E-03	SPP1, VCAM1	2
Respiratory Disease	acute respiratory distress syndrome	acute respiratory distress syndrome	5.18E-03	F3, IGFBP3, PLA2G2A	3
Organismal Functions	healing	healing	5.30E-03	COL3A1, COL5A1, GJA1, SERPINB2, SPP1	5
Organismal Functions	healing	healing of wound	5.78E-03	COL3A1, COL5A1, GJA1, SERPINB2	4

Highly Expressed in CVS Versus MBC

Table S1H

				¬© 2000-2009 Ingenuity Systems, Inc. All rights reserved.	
**Category**	**Function**	**Function Annotation**	**P-Value**	**Molecules**	**# Molecules**
Cell-mediated Immune Response	immune response	immune response of organism	6.29E-03	ASS1, COL3A1, COLEC12, EBI3, GH1, KRT8, LYVE1, PSG3, TLR3	9
Lymphoid Tissue Structure and Development	proliferation	proliferation of lymphatic endothelial cells	7.09E-03	HGF, KDR	2
Cell Signaling	quantity	quantity of Ca2+	7.50E-03	AGTR1, ANGPT2, DCN, GH1, HGF, ITGAV, KDR, KISS1, PKD2 (includes EG:5311)	9
Vitamin and Mineral Metabolism	quantity	quantity of Ca2+	7.50E-03	AGTR1, ANGPT2, DCN, GH1, HGF, ITGAV, KDR, KISS1, PKD2 (includes EG:5311)	9
