# Supplementary material for: Structural and Regulatory Characterization of the Placental Epigenome at Its Maternal Interface
Source: PLoS One. 2011 Feb 23;6(2):e14723. doi: 10.1371/journal.pone.0014723 (PMC3044138; doi:10.1371/journal.pone.0014723)
Supplement: Table S9 — List of genes that are differentially expressed between CVS and MBC. (0.04 MB PDF) [file pone.0014723.s009.pdf]

# Table S9 CH3 Paper

| Overexpressed in CVS versus MBC | Overexpressed in MBC versus CVS |
|---|---|
| ABCB1 /// ABCB4 | AMICA1 |
| ABCG2 | AQP9 |
| ADAMTS1 | C19orf59 |
| AGL | C1orf38 |
| AGPAT5 | C5AR1 |
| AGTR1 | CD2 |
| ANGPT2 | CD247 |
| ANLN | CD48 |
| ARMCX1 | CD52 |
| ASS1 | CD8A |
| BEX4 | CFP |
| BTBD3 | CMTM2 |
| C12orf23 | CST7 |
| CDH1 | CX3CR1 |
| CDH5 | CYP4F3 |
| CDK7 | CYTIP |
| CDO1 | DEFA1 /// DEFA3 /// LOC728358 |
| CETN3 | EPB42 |
| CGA | EPB49 |
| CGB /// CGB5 /// CGB7 | EVI2B |
| CKS2 | FAM129A |
| CLIC3 | FCGR2A |
| CNN3 | FCGR3A /// FCGR3B |
| COL15A1 | FCGR3B |
| COL21A1 | FCN1 |
| COL3A1 | FFAR2 |
| COL4A1 | FGD3 |
| COL4A2 | FGR |
| COL5A1 | FPR1 |
| COL5A2 | GALNAC4S-6ST |
| COL6A3 | GMPR |
| COLEC12 | GZMH |
| CRIM1 | GZMK |
| CRY1 | HBD |
| CSH1 | HBM |
| CSH1 /// CSH2 /// CSHL1 /// GH1 /// GH2 | HBQ1 |
| CSH2 | HCK |
| CSHL1 | HLA-DRB1 /// HLA-DRB4 /// HLA-DRB5 |
| CSRP2 | IGK@ /// IGKC |
| CTHRC1 | IL7R |
| CYP11A1 | IL8RB |
| DCN | ITK |
| DKK1 | KCNJ15 |

| Overexpressed in CVS versus MBC | Overexpressed in MBC versus CVS |
|---|---|
| DLK1 | KRT1 |
| DPP4 | LCK |
| DSG2 | LGALS3 |
| DSP | LITAF |
| DUSP9 | LOC100131993 |
| EBI3 | LOC642103 /// MGAM |
| EFEMP1 | LOC727820 |
| EFHA2 | LRRK2 |
| EGFL6 | LTB |
| ENPEP | LTF |
| ENPP2 | LYZ |
| EPS8 | MNDA |
| F3 | MPZL3 |
| FABP5 | MYL4 |
| FAM115A | NAMPT |
| FBLN1 /// LOC100133843 | NCF2 |
| FBN1 | NFE2 |
| FERMT2 | NIACR2 |
| FNBP1L | OR2W3 |
| FRMD6 | P2RY13 |
| GABARAPL1 /// GABARAPL3 | P2RY8 |
| GABRE | PABPC1 |
| GCM1 | PDZK1IP1 |
| GCSH | PF4 |
| GGH | PIK3AP1 |
| GH1 | PRF1 |
| GH2 | PROK2 |
| GHR | PTPRC |
| GJA1 | RASSF5 |
| GLDC | RGS18 |
| GMNN | S100A12 |
| GNAI1 | S100A8 |
| GNGT1 | SATB1 |
| GPX3 | SELL |
| GRHL1 | SESN3 |
| H19 | SIGLEC5 |
| HAPLN1 | SLC25A37 |
| HGF | SLC25A39 |
| HS6ST2 | SLC4A1 |
| HSD11B2 | SORL1 |
| HSD17B2 | SRGN |
| HSPA2 | SSH2 |

| Overexpressed in CVS versus MBC | Overexpressed in MBC versus CVS |
|---|---|
| HSPB1 | TAGAP |
| HSPE1 | TMEM71 |
| HTRA1 | TNFAIP6 |
| IFI6 | TNFSF13B |
| IGFBP3 | TRA@ /// TRAC |
| INSL4 | TRBC1 |
| ITGAV | TRBC1 /// TRBC2 |
| KCTD3 | TRIM58 |
| KDELC2 | VNN2 |
| KDR | XK |
| KISS1 | |
| KRT18 | |
| KRT8 | |
| LAMB1 | |
| LAMC1 | |
| LGALS13 | |
| LGALS14 | |
| LGMN | |
| LIFR | |
| LIN28B | |
| LIPG | |
| LRP11 | |
| LUM | |
| LYVE1 | |
| MAFF | |
| MAGEA8 | |
| MAOA | |
| MAP4K3 | |
| MATN2 | |
| MEST | |
| MFAP5 | |
| MORC4 | |
| MRC1 /// MRC1L1 | |
| NET1 | |
| NFE2L3 | |
| NFU1 | |
| NID2 | |
| NRK | |
| OGN | |
| OLR1 | |
| P11 | |
| PABPC4L | |

| Overexpressed in CVS versus MBC | Overexpressed in MBC versus CVS |
| --- | --- |
| PAGE4 | |
| PCOLCE2 | |
| PEG10 | |
| PEG3 /// ZIM2 | |
| PGM3 | |
| PHF16 | |
| PHYH | |
| PIR | |
| PITX2 | |
| PKD2 | |
| PKIB | |
| PLA2G2A | |
| PLEKHH1 | |
| PLK2 | |
| PLOD2 | |
| PLS3 | |
| PLSCR4 | |
| PMP22 | |
| PP14571 | |
| PPAP2B | |
| PPIC | |
| PROCR | |
| PSG1 | |
| PSG2 | |
| PSG3 | |
| PSG6 | |
| PSG7 | |
| PSG9 | |
| PVRL3 | |
| RACGAP1 | |
| RAI14 | |
| RASA1 | |
| RCN1 | |
| RCN2 | |
| RDH14 | |
| RHOBTB1 | |
| RND3 | |
| RNF128 | |
| ROBO1 | |
| SDC1 | |
| SERPINB2 | |
| SERPINF1 | |

## Table S2 CH3 Paper

| Overexpressed in CVS versus MBC | Overexpressed in MBC versus CVS |
|---|---|
| SGCE | |
| SKP2 | |
| SLC16A4 | |
| SLC19A2 | |
| SLC27A2 | |
| SMS | |
| SNAI2 | |
| SNRPG | |
| SPARC | |
| SPESP1 | |
| SPP1 | |
| SRPX | |
| STS | |
| TCEAL4 | |
| TCHH | |
| TFPI2 | |
| TLR3 | |
| TTC35 | |
| TWIST1 | |
| TWSG1 | |
| UAP1 | |
| VCAM1 | |
| VGLL3 | |
| WBP5 | |
| YAP1 | |
| ZNF117 | |
| ZNF468 | |
